# Genomic Structural Equation Modeling Combined With Post‐GWAS Analysis Identifies Two Risk Gene Loci and Functionally Sensitive Genes Associated With Cardiac Conduction Block

**DOI:** 10.1155/genr/1063531

**Published:** 2026-01-14

**Authors:** Tongyu Wang, Xinge Miao, Yunlong Xia

**Affiliations:** ^1^ Department of Cardiology, The First Affiliated Hospital of Dalian Medical University, Dalian, Liaoning, China, dlmedu.edu.cn; ^2^ Department of Cancer Medicine, The First Affiliated Hospital of Dalian Medical University, Dalian, Liaoning, China, dlmedu.edu.cn

**Keywords:** biomarkers, cardiac conduction disorders, genomic structural equation

## Abstract

**Background:**

Cardiac conduction disorders (CCDs) represent a broad spectrum of severe cardiovascular conditions associated with syncope and sudden cardiac death. Therefore, identification of reliable biomarkers is necessary to significantly improve the diagnostic accuracy and therapeutic outcomes of CCDs. This study analyzed GWAS summary datasets using a genomic structural equation model (Genomic‐SEM), fine mapping, linkage disequilibrium score regression (LDSC), and two‐sample Mendelian randomization (TSMR) analyses to identify genetic loci and genes associated with CCDs.

**Methods:**

GWAS summary datasets of European subjects were obtained from the GWAS Catalog and FinnGen databases. The GenomicSEM R package was used to construct a structural equation model to identify common latent factors influencing CCD progression. The Functional Mapping and Annotation of Genome‐Wide Association Studies (FUMA) platform was used to annotate the lead SNPs and candidate genes. Fine‐mapping tools, such as SuSiE and FINEMAP, and Phenome‐Wide Association Study (PheWAS) analysis were used to identify causal SNPs associated with CCDs. Transcriptome‐Wide Association Study (TWAS) and Functional Summary Statistics (FOCUS) analyses were performed to identify CCD susceptibility genes. LDSC and TSMR were performed to determine causal relationships between the candidate risk genes and specific CCDs.

**Results:**

Newly explored CCD‐associated leading SNPs (rs71208329 and rs112720315) were generated from genomic SEM and FUMA analyses. Fine‐mapping and PheWAS analysis confirmed that rs112720315 was linked to nonischemic cardiomyopathy. TWAS, FUMA, and FOCUS analyses showed that five genes (*CCDC141*, *SCN10A*, *SH3PXD2A*, *FKBP7*, and *ESR2*) were associated with CCDs. The *APOL1* gene is associated with the risk of CCDs in African ancestry. TSMR and LDSC analyses further demonstrated that these genes were significantly associated with CCDs and were potential prediction biomarkers for CCDs.

**Conclusion:**

The novel genetic locus rs112720315 is significantly associated with the occurrence of CCDs. Biomarkers such as *CCDC141*, *SCN10A*, *ESR2*, *FKBP7*, *and SH3PXD2A* can predict a wide spectrum of CCDs. The *APOL1* gene is a specific marker for CCDs in African ancestry.

## 1. Background

Cardiac conduction disorders (CCDs) are a group of clinical conditions characterized by disruptions in the normal cardiac depolarization, leading to bradycardia [1]. Clinical presentation of CCDs varies widely, from asymptomatic cases detected incidentally on electrocardiograms (ECGs) to severe cases causing syncope, heart failure, or sudden cardiac death [2]. Clinically important CCDs include sick sinus syndrome (SSS), atrioventricular block (AVB), left bundle branch block (BBB) (LBBB), and right BBB (RBBB). Each type of conduction disorder results from different levels of damage to the conduction system. For example, AVB derives from a block in the His bundle or AV node. LBBB and RBBB derive from damage to the distal His bundle area. Epidemiological studies have demonstrated that prolonged PR intervals and RBBB are significantly associated with an increased risk of cardiovascular disease (CVD) mortality [3, 4]. Twenty percent of individuals in a recent multicenter cohort study involving 189,163 samples were diagnosed with a conduction disorder at baseline, and over 6% of the study sample developed a new conduction disorder over 10 years of follow‐up [5].

The pathogenesis of CCDs involves the loss of nonrenewable cardiomyocytes during the aging process [6, 7]. Moreover, animal studies have demonstrated that dysregulation of calcium‐handling proteins impairs atrioventricular node conduction by disrupting Ca^2+^ homeostasis in the endoplasmic reticulum [8, 9]. Mutations in the connexin 45 gene cause progressive AVB [10]. Purkinje cell (PC) dysfunction also contributes to intraventricular conduction disorders and heart failure. Nav1.5 α‐subunit (*SCN5A*) gene mutation is related to His‐Purkinje conduction disease and cellular hyperexcitability [11]. *Cx43* polymorphism is associated with increased risk of developing LBBB [12]. Epigenetic markers also contribute to the pathogenesis of sinoatrial node (SAN) dysfunction. The expression of miR‐486‐3p is significantly lower in normal SAN tissue than in nonpacemaker atrial muscle tissue, and miR‐486‐3p is involved in the regulation of HCN4 expression [13]. Human HF hearts exhibit elevated miR‐486‐3p expression in the SAN [14].

Currently, specific circulating biomarkers to definitively predict the risk of CCDs and guide intervention strategies are unavailable. Furthermore, different types of conduction disorders can be explained by common mechanisms, such as conduction system dysfunction. The genomic structural equation model (Genomic‐SEM) uses published CCD GWAS data to create a new GWAS summary dataset. This helps explain latent confounding factors for diseases and speeds up the discovery of new genetic loci for CCDs. Unlike traditional GWAS meta‐analysis, Genomic‐SEM considers causal relationships and correlations among multiple traits. MTAG, however, only enhances the significance of single genetic loci via correlation between two traits and cannot handle complex frameworks [15]. We used multiple methods, including fine mapping, Phenome‐Wide Association Study (PheWAS), Transcriptome‐Wide Association Study (TWAS), Functional Mapping and Annotation of Genome‐Wide Association Studies (FUMA), and Functional Summary Statistics (FOCUS), to determine the associations between CCDs and genetic markers. Linkage disequilibrium (LD) score regression (LDSC) and two‐sample Mendelian randomization (TSMR) were performed to determine causal relationships between the candidate risk genes and specific CCDs. The overall aim was to discover potential clinical biomarkers as therapeutic targets and identify biomarkers that predict broad‐spectrum CCD risk.

## 2. Methods

### 2.1. Source and Genomic Parameters of GWAS Summary Data

We analyzed GWAS summary‐level data for CCDs in a European ancestry population. The database construction methods and quality control parameters are shown in Table 1.

**Table 1:** Details of GWAS summary datasets used for the structural genomic equation analysis of European ancestry data.

Accession no.	Ancestry	Cases	Controls	Sample size	Ratio	Imputation panel	Source	λGC	NSNP	Definition
GCST90475955	European	2012	447,369	449,381	0.0044	1000 Genomes Project	GWAS Catalog	1.0288	1,173,070	Second‐degree AV block
GCST90477911	European	6274	443,107	449,381	0.0139	1000 Genomes Project	GWAS Catalog	1.0606	1,173,072	Atrioventricular block, complete
GCST90475980	European	6431	439,404	445,835	0.0144	1000 Genomes Project	GWAS Catalog	1.0767	1,173,071	Sinoatrial node dysfunction (bradycardia)
GCST90475954	European	6072	435,543	441,615	0.0137	1000 Genomes Project	GWAS Catalog	1.0784	1,173,067	First‐degree AV block
I9_LBBB	European	2766	375,343	378,109	0.0073	SISu v4.2	FinnGen	1.059	1,159,470	Left bundle branch block
I9_RBBB	European	1347	375,343	376,690	0.0035	SISu v4.2	FinnGen	1.0291	1,159,466	Right bundle branch block

### 2.2. Genomic Structural Equation Modeling Analysis

The GenomicSEM R package (v.0.0.5) was used to construct a Genomic‐SEM to investigate the broad genetic susceptibility underlying CCDs such as SSS, IAVB, IIAVB, IIIAVB, LBBB, and RBBB. Genomic‐SEM is a multivariate method that evaluates genetic relationships between multiple traits simultaneously and demonstrates robustness against biases due to sample overlap or unequal sample sizes. It also distinguishes genetic variants affecting specific trait subsets from those influencing cross‐trait susceptibility. This analysis used a two‐stage approach, wherein the empirical genetic covariance matrix and its sampling covariance matrix were first analyzed, followed by model fitting. We constructed the model based on a hypothesis that conduction disorders can be explained by one common latent factor.

A multivariate cross‐trait LD score regression analysis was performed, and an empirical genetic covariance matrix for six traits was generated as inputs for the SEM common factor model. Discrepancy between the empirical matrix and the hypothesized covariance structure was minimized using structural equation modeling. This study specifically investigated the genetic architecture underlying the six CCD traits and evaluated a single‐factor model. Subsequently, we evaluated the model fit using the standardized root‐mean‐square residual (SRMR), χ^2^ statistic, Akaike information criterion (AIC), and comparative fit index (CFI). The χ^2^ statistic assessed the discrepancy between the genetic covariance matrix and the empirical covariance matrix. The AIC balanced the model fit and complexity for model selection and was used to compare models. SRMR quantified the standardized residuals between predicted and observed values, with values below 0.08 suggesting a good fit. The CFI compared the model to a perfect fit, with values closer to 1 indicating a better fit.

### 2.3. Identifying Genomic Loci and Novel Variants Associated With CCDs

The FUMA platform is a valuable tool for identifying genomic loci and lead single‐nucleotide polymorphisms (SNPs) linked with the CCDs [16]. Lead SNPs were defined as variants with a genome‐wide significance of *p* < 5 × 10^−8^ and a low LD of *r* ^2^ < 0.1. Genomic‐SEM was used to assess the strength of association of potential risk SNPs with CCDs by integrating summary statistics from GWAS. These lead SNPs and loci were then compared against those identified in the original univariate GWAS. SNPs demonstrating greater significance in the Genomic‐SEM than in the individual univariate GWAS analysis were considered novel genetic loci. Furthermore, MAGMA (v1.08) functions in the FUMA website were also used to expand genes associated with conduction disorders, and genes with adjusted Bonferroni *p*‐value < 0.05 were selected.

### 2.4. Fine Mapping to Identify Causal SNPs

Fine‐mapping methods, such as Sum of Single Effects (SuSIE) and FINEMAP, from the R package echolocatoR (v. 2.0.3), were used to identify the most probable causal variants in the GWAS analysis. A probability threshold of 0.95 was used to identify credible sets of potential causal variants. Both SuSIE and FINEMAP are statistical fine‐mapping methods used to identify specific genetic variants that are most likely responsible for the observed phenotype [17]. A 250‐kb region around each lead SNP was selected to capture potentially associated variants. Then, the posterior inclusion probability (PIP) was calculated for all the SNPs within these intervals. Genetic variants with PIP > 0.95 were classified as probable causal variants.

### 2.5. TWAS

After identifying potential causal variants, a TWAS was performed to detect genes associated with CCDs based on their expression–phenotype relationships. The FOCUS (v0.802) method was used to assess causal gene–phenotype relationships by calculating PIP values. The FUSION method was used to evaluate these associations using precomputed mixed tissue panels’ expression quantitative trait loci (eQTL) weights from GTEx v8, which included 37,920 gene–tissue pairs [18]. Weight panels are developed based on expression–phenotype associations across multiple genes and types of tissue mixtures and were used for the exploratory identification of associated genes. Moreover, to confirm the results from the mixture panels, we also used left ventricle and atrial appendage weights from GTEx v8.

Weight panels can be found at the following website: https://gusevlab.org/projects/fusion/#gtex-cross-tissue-scca-expression.

### 2.6. LDSC Analysis

LDSC is used to estimate genetic correlations from GWAS summary statistics by quantifying conserved genetic effects between quantitative traits. The LDSC manual outlines intercept and heritability as key GWAS quality check metrics. Intercept, aiming for a value of 1, detects potential confounding factors. Heritability (h2) quantifies the proportion of trait variation explained by common genetic variants. Genetic correlation (*r*_*g*_) was calculated using the R package “ldscr (v0.1.0)” [19].

### 2.7. TSMR Analysis

The causal relationship between genetic markers and abnormal conduction on an ECG was explored through TSMR analysis. eQTL data were derived from the eQTLGen database and included results of testing blood samples from 31,684 individuals, encompassing 16,989 gene eQTL loci. This approach ensured compliance with the following core assumptions of Mendelian randomization analysis: (i) SNPs have a strong correlation; (ii) SNPs are independent of confounders; and (iii) SNPs have no effect on outcome [20]. We filtered exposure datasets by retaining variants with a *p*‐value of < 5*e* − 8 and *F* < 10, removing SNPs showing LD using clumping parameters of 10,000 kb window size and r^2^threshold of 0.6. For the outcome datasets, SNPs with *p* < 5*e* − 8 were excluded to minimize direct effects on the outcomes. Egger’s regression was used to determine whether the instrumental variable had horizontal pleiotropy. At the core, we evaluated whether the intercept significantly deviated from zero using regression analysis. We used the Steiger directionality test to verify the directionality of causal relationships. The basic logic involves determining the causal direction by comparing the correlation between SNPs and exposures, as well as between the instrumental variable and the outcome. The Mendelian randomization analysis was conducted using the “TwoSampleMR (v 0.6.2)” package in R, and both random‐effects inverse‐variance weighted (IVW) and Wald ratio methods were used to estimate causal effects between exposures and outcomes. Regarding the median analysis, the median effect was evaluated using the “RMediation (v 1.2.2)” package using the “medic” function.

## 3. Results

### 3.1. Quality Control Parameters for GWAS Summary Data and the Genomic‐SEM

Based on the LDSC analysis, the heritability estimates (*h* ^2^) for the six GWAS traits were as follows: SSS (0.006 ± 0.0012), IAVB (0.0059 ± 0.0012), IIAVB (0.0019 ± 0.0011), IIIAVB (0.0061 ± 0.001), LBBB (0.006 ± 0.0014), and RBBB (0.0027 ± 0.0014). Genetic covariance values between traits are shown in Figure 1(a). Our data showed positive genetic correlations between AVBs and infra‐His bundle conduction disorders, such as LBBB and RBBB. SSS exhibits a positive correlation with AVB and a negative correlation with BBBs. BBBs demonstrate negative correlations with AVBs. Subsequently, a Genomic‐SEM was constructed using the configuration shown in Figure 1(b).

Figure 1(a) Correlation plot of the LD score regression analysis shows associations between distinct types of conduction disorders. (b) The genomic structural equation model for calculating genetic correlations between distinct types of conduction disorders. SSS, sick sinus syndrome; LBBB, left branch bundle block; RBBB, right branch bundle block; IAVB, first‐degree atrioventricular block; IIAVB, second‐degree atrioventricular block; IIIAVB, third‐degree atrioventricular block.

**Figure:**
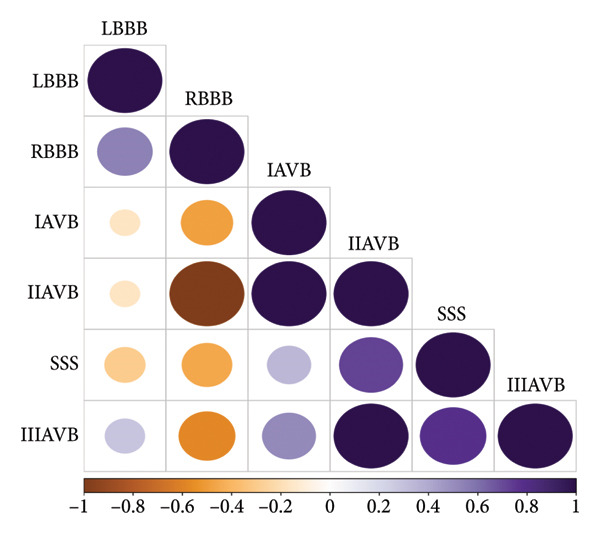
(a)

**Figure:**
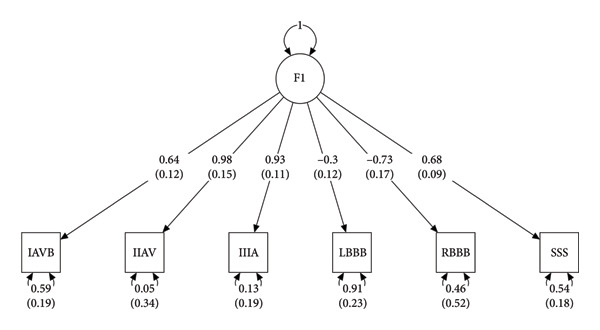
(b)

Before constructing the model, the genetic relationships between the six GWAS traits were analyzed using GenomicSEM to identify the effectiveness of the model. Regarding the chi‐square test, the *χ* ^2^ was 8.2165 and the *p*‐value was 0.5124, indicating that the model showed no difference between the genetic covariance matrix and the empirical covariance matrix. The CFI value of 1.00 in this study indicates that the model fits very well. The AIC value of this model was 32.2165. Although our SRMR value (0.0984) was slightly above 0.08, it is still within an acceptable range, indicating that the model performs well in fitting the data. Table 2 shows the latent factor derived from genome structural equation modeling and the univariate SEM parameter associations. As the table indicates, different degrees of AVBs and SSS are significantly positively associated with the common latent factor, whereas intraventricular conduction block is significantly negatively associated with the common latent factor. These findings support the presence of a conserved genetic architecture among the CCD traits evaluated in this study. In‐depth molecular mechanisms may differ between the two categories of CCDs (AVB and BBBs).

**Table 2:** Genome structural equation for identifying the latent factor (F1) and univariate SEM parameters.

Lhs	Op	Rhs	Unstandardized_estimate	Unstandardized_SE	Standardized_Est	Standardized_SE	*p*_value
F1	=∼	SSS	0.17	0.023	0.68	0.092	2.18*e* − 13
F1	=∼	IAVB	0.16	0.03	0.63	0.11	2.78*e* − 08
F1	=∼	IIAVB	0.27	0.04	0.97	0.14	3.64*e* − 11
F1	=∼	IIIAVB	0.25	0.029	0.93	0.10	2.44*e* − 18
F1	=∼	LBBB	−0.09	0.037	−0.29	0.11	0.0098
F1	=∼	RBBB	−0.21	0.049	−0.73	0.16	1.19*e* − 05
SSS	∼∼	SSS	0.03	0.011	0.53	0.17	0.0021
IAVB	∼∼	IAVB	0.040	0.013	0.59	0.18	0.0017
IIAVB	∼∼	IIAVB	0.003	0.026	0.045	0.33	0.89
IIIAVB	∼∼	IIIAVB	0.01	0.014	0.13	0.18	0.47
LBBB	∼∼	LBBB	0.097	0.025	0.91	0.23	0.00010
RBBB	∼∼	RBBB	0.039	0.044	0.46	0.51	0.37

*Note:* This table shows the correlation between each conduction disorder and the common latent factor (F1), as well as the residual covariance that cannot be explained by the latent factor.

### 3.2. Quality Control and Statistical Genetic Analysis Report for the GenomicSEM Identified a Latent Factor

Quality analysis of latent factor GWAS summary datasets using methodological parameter controls resulted in the exclusion of 6,401,566 SNPs and retention of 1,117,809 effective variants. The characteristics of the retained SNPs were as follows: mean *X* ^2^ = 1.1023; genomic control lambda (λGC) = 1.1491; max *X* ^2^ = 33.324; contribution ratio of genetic component to environment = 0.1066 (0.0623); and *h* ^2^ = 0.0699 (0.0083).

### 3.3. Identification of CCD‐Related Genetic Loci and Genes Based on Structural Equation Modeling and FUMA Software Analysis of GWAS Data

We identified four risk loci (rs71208329, rs13031826, rs2634071, and rs112720315; Table S1) with parameter *p*‐value < 5*e − *8 and LD *R* ^2^ < 0.1 using the FUMA website. Among these, rs71208329 and rs112720315 were significant SNPs in the common latent factor GWAS summary dataset; these loci had smaller *p*‐values than others in each type of conduction disorder. Furthermore, we identified four genes significantly associated (FDR < 0.05) with the CCD traits (*CCDC141*, *SCN10A*, *SH3PXD2A*, and *ESR2*) by analyzing GWAS data (Figure 2).

Figure 2Manhattan plots from the FUMA software analysis. (a) Annotation of lead SNPs using the FUMA software with *p*‐value < 5 × 10^−8^ and *R* ^2^ < 0.1 set as threshold parameters. (b) Identification of CCD‐associated genes (*CCDC141, SCN10A, SH3PXD2A, and ESR2*) with significant adjusted *p*‐values based on the FUMA software analysis.

**Figure:**
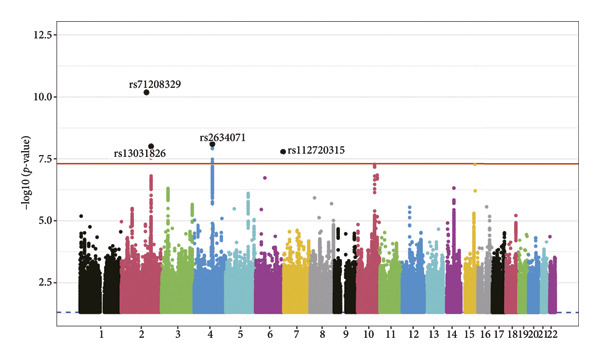
(a)

**Figure:**
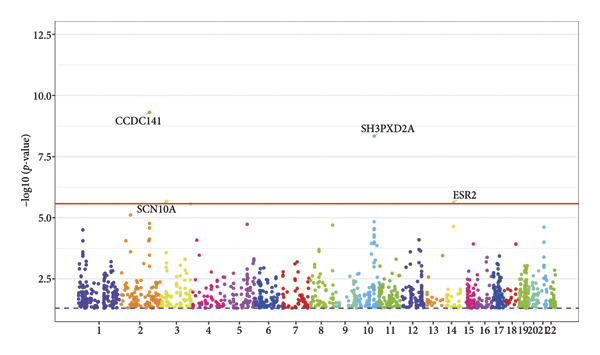
(b)

### 3.4. Fine‐Mapping Analysis Identifies Latent Factor as the Causal SNP

A fine‐mapping analysis demonstrated strong associations between multiple genomic loci and the CCDs, especially rs71208329, a variant within the AC023469.1 region on Chromosome 2, and rs112720315, a variant located in the TCP10L2 locus on Chromosome 6. SuSiE indicated that rs71208329 has a posterior probability of 1, and FINEMAP also indicated a posterior probability of 1. SuSiE and FINEMAP showed that rs112720315 has a posterior probability of 0.99 and 0.99, respectively. Thus, both methods support the causal effect of the variants. Regional association plots displayed distinct peaks at these locations in Chromosomes 2 and 6, and these associations were further confirmed by their identification as credible set variants (Figure 3 and Figure S1).

Figure 3The LocusZoom plot shows the fine‐mapping analysis results. The plots show results from two methods (SuSiE and FINEMAP) of fine‐mapping analysis. The lowest panel shows the final results of the fine‐mapping analysis. (a) A fine‐mapping plot showing a PP of > 0.95 for rs112720315 with both the SuSiE and FINEMAP fine‐mapping methods, thereby indicating its causal role in the pathogenesis of CCDs. (b) A fine‐mapping plot demonstrating a PP of > 0.95 for rs71208329 with SuSiE and FINEMAP methods, thereby indicating its causal role in the pathogenesis of CCDs.

**Figure:**
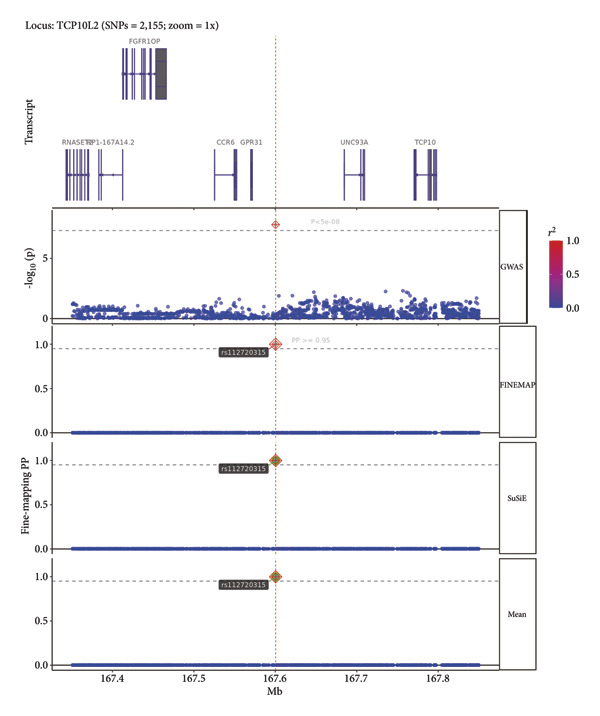
(a)

**Figure:**
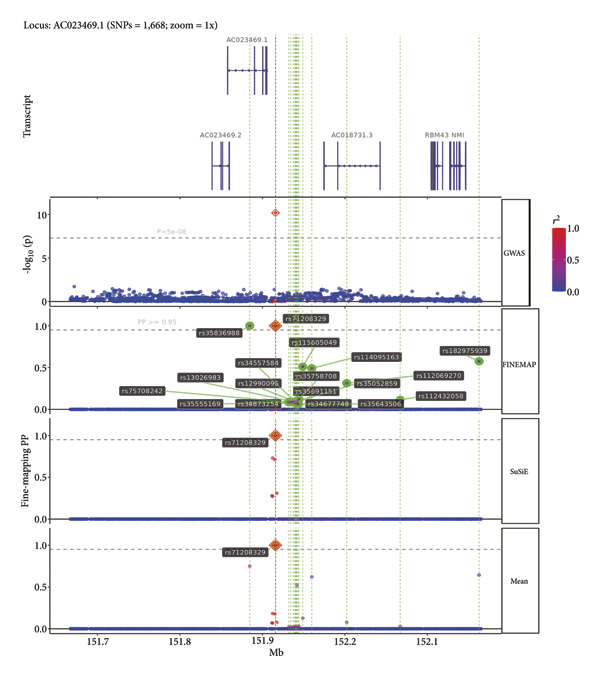
(b)

### 3.5. PheWAS Analysis Confirms a Significant Association Between rs112720315 and Nonischemic Cardiomyopathy

**Figure 4:**
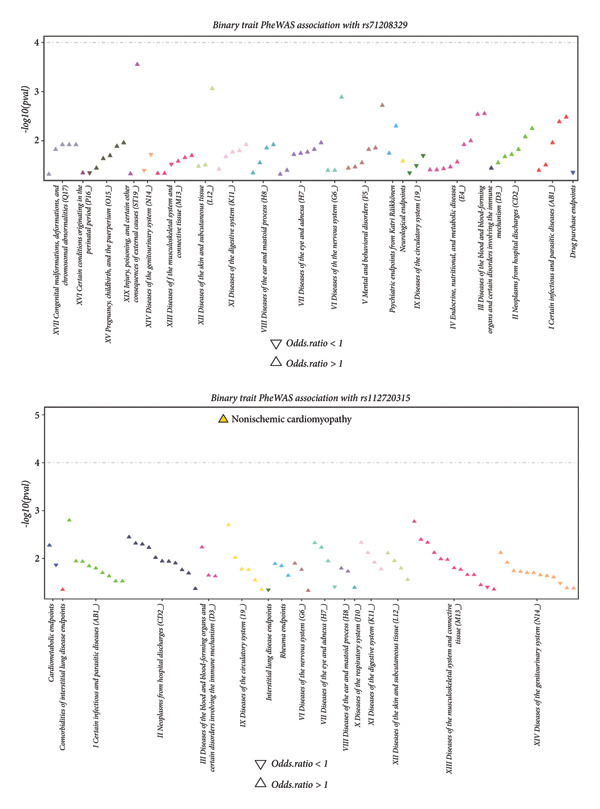
PheWAS analysis based on the FinnGen database. The association between significant SNPs identified by fine‐mapping analysis and CCD‐related traits is illustrated. As shown, only nonischemic cardiomyopathy is correlated with rs112720315 (odds ratio > 1).

We then assessed the association between the two significant SNPs (rs71208329 and rs112720315) and the CCD‐associated traits from the FinnGen database. We found a significant association between nonischemic cardiomyopathy and rs112720315 (OR > 1) using a *p*‐value threshold of 1 × 10^−4^ (Figure 4). This suggested a potential link between CCDs and cardiomyopathy.

### 3.6. Transcriptome‐Wide Association Analysis Demonstrates a Significant Causal Association Between *FKBP7* and CCDs

**Figure 5:**
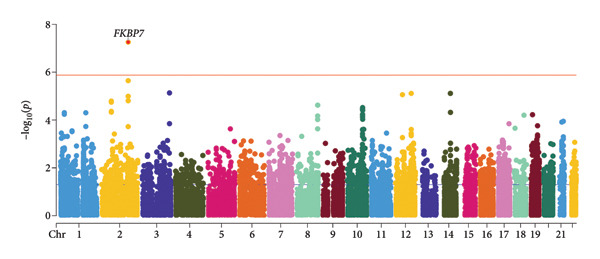
A Manhattan plot of the FUSION TWAS results. As shown, only the *FKBP7* gene is significantly associated with CCDs based on the adjusted *p*‐value.

Subsequently, we performed TWAS using the FUSION software based on three types of tissue mixture panels (sCCA1, sCCA2, and sCCA3) to identify genes significantly associated with CCDs. Among 37,640 genes identified by TWAS (Table S2 indicated those with TWAS *p*‐value < 0.05), only FKBP7 in the sCCA2 panel reached TWAS significance (*p-value < 0.05/37,640*) (Figure 5) and exhibited a positive relationship with the SEM‐identified factor. Subsequently, we conducted a FOCUS fine‐mapping analysis of the genomic structural equation modeling data and intersected the result with the significant genes in the TWAS analysis (Table S3). We found that *FKBP7* (PIP > 0.9 in the FOCUS analysis) demonstrated significant causal relationships with CCDs.

To further confirm the role of *FKBP7* in cardiac‐specific tissues, we repeated the TWAS and FOCUS analyses on the left ventricle and atrial appendage panels. We found that *FKBP7* still exhibited a positive correlation and a causal relationship (PIP > 0.7 in FOCUS test) with the CCD latent factor in the left ventricle panel (Table 3).

**Table 3:** Validation of TWAS and FOCUS results of FKBP7 in heart‐related tissues.

Panel	ID	CHR	P0	P1	Best.GWAS.ID	eQTL.ID	eQTL.R2	eQTL.Z	eQTL.GWAS.Z	NSNP	TWAS.Z	TWAS.P	FDR	Pips
Heart_Atrial_Appendage	FKBP7	2	178,478,599	178,478,600	rs13019119	rs13411588	0.12	6.7	3.55	464	3.4	0.000473	0.17	0.71
Heart_Left_Ventricle	FKBP7	2	178,478,599	178,478,600	rs13019119	rs13411588	0.13	6.85	3.55	463	3.5	0.000378	0.15	0.052

### 3.7. LDSC and TSMR Demonstrate a Causal Association Between the Identified Genes and CCDs

We used a MAGMA analysis to further annotate genes associated with the latent factor F1. However, MAGMA cannot verify causal effects. We identified four genes (*CCDC141, SCN10A, SH3PXD2A,* and *ESR2*) with an adjusted *p*‐value < 0.05. To further determine whether the identified genes can predict the occurrence of different types of CCDs, we analyzed eQTL data from the eQTLGen cohort for the identified genes.

For the GWAS dataset parameters (in Table S4), we found that *SH3PXD2A’s* intercept (12.20) was significantly larger than 1, indicating population stratification. Although *CCDC141* (intercept: 0.1014) and *ESR2* genes had intercepts significantly lower than 1, this suggests that the trait was affected by some confounding factors. Moreover, regarding heritability, we found that the CCDs exhibited significantly lower *h* ^2^ (< 0.01), which was due to the relative size or depth of the included GWAS summary data.

As shown in Figure 6(a), *CCDC141* demonstrated a positive correlation with RBBB and negative correlations with SSS, IIIAVB, IIAVB, and IAVB. Furthermore, *CCDC141* demonstrated a strong association with the latent factor identified from the GenomicSEM analysis. *ESR2* demonstrated a significant positive correlation with SSS, IIIAVB, IIAVB, and IAVB and a negative association with RBBB. *FKBP7* demonstrated significant positive correlations with F1, SSS, IIIAVB, IIAVB, LBBB, and IAVB and a negative association with RBBB. *SH3PXD2A* demonstrated significant negative correlations with F1, IIIAVB, IIAVB, and IAVB.

Figure 6(a) A correlation heat map shows the LDSC analysis results of the association between four specific genes (*CCDC141*, *ESP2*, *FKBP7*, and *SH3PXD2A*) and multiple CCDs. The red color indicates positive correlations with the CCD traits. (b) A heat map shows the results of the two‐sample Mendelian randomization analysis of the causal relationships between four specific genes (*CCDC141*, *ESP2*, *FKBP7*, and *SH3PXD2A*) and multiple CCDs.

**Figure:**
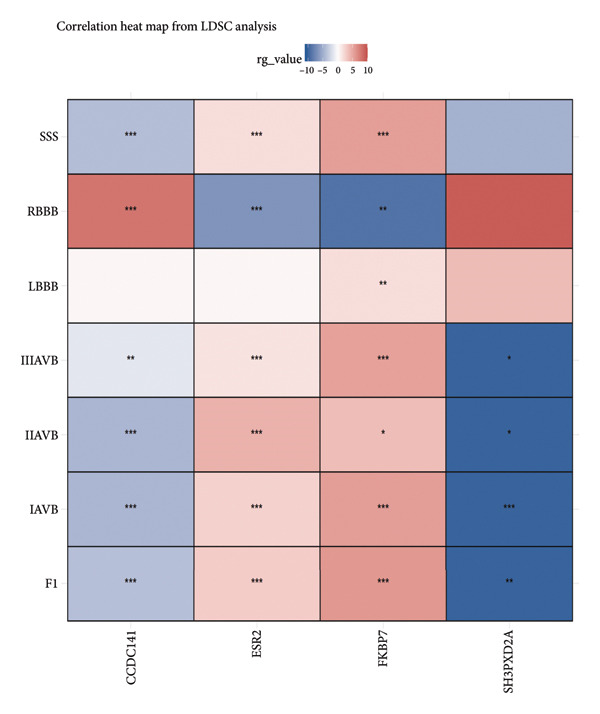
(a)

**Figure:**
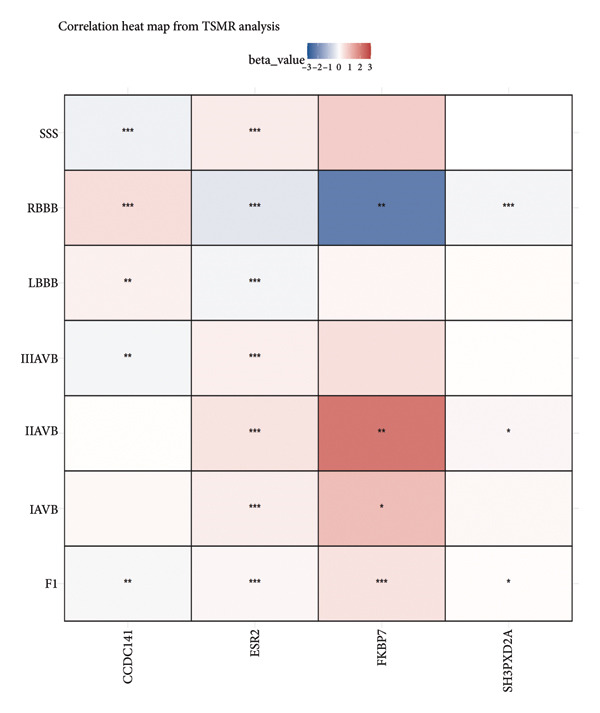
(b)

The TSMR analysis results (Figure 6(b)) demonstrated a positive causal relationship of *CCDC141* with RBBB and LBBB. Furthermore, *CCDC141* showed negative causal relationships with IIIAVB and F1. *ESR2* demonstrated significant positive correlations with SSS, IIIAVB, IIAVB, and IAVB and negative correlations with RBBB and LBBB. *FKBP7* demonstrated significant positive correlations with F1, IIAVB, and IAVB and a negative association with RBBB. *SH3PXD2A* showed a negative correlation with RBBB and positive correlations with IIAVB and F1. The Steiger test and pleiotropy tests demonstrated that the TSMR results were stable and reliable (Table S5).

In summary, we identified genetic biomarkers for accurately predicting a wide spectrum of CCDs. This includes *CCDC141,* which increases the risk of SSS, RBBB, IIAVB, and latent factor; *ESR2,* which increases the risk of SSS, IIIAVB, IIAVB, IAVB, and RBBB; and *FKBP7*, which increases the risk of IAVB, RBBB, and IIAVB.

### 3.8. Cross‐Ancestry Validation of the Efficiency of the Above Model

In the TWAS section, we found that the *APOL1* gene was significantly associated with a latent factor in two reference panels (sCCA1 and sCCA2). *APOL1* was previously reported to be associated with kidney diseases in individuals of African ancestry [21, 22]. Therefore, we tested the structural model among individuals of African ancestry. The included datasets are shown in Table 4. The *χ* ^2^ was 3.29 (*p*‐value: 0.95), indicating that the model showed no difference between the genetic covariance matrix and the empirical covariance matrix. The CFI value of 1.00 in this study indicates that the model fits very well. The AIC value in this model was 27.29, indicating a relatively better fit than that of those of European ancestry. However, the SRMR value of the model was 0.219, which is higher than that of the European model, indicating that the model may not fit African ancestry data. Regarding the relationship between each type of CCD and the common latent factor (F1), we found no significant association between F1 and IIAVB, LBBB, and SSS (*p*‐value > 0.05; Table 5).

**Table 4:** Details of GWAS summary datasets used for the structural genomic equation analysis of African ancestry data.

Accession no.	Ancestry	Cases	Controls	Sample size	Ratio	Imputation panel	Source	λGC	NSNP	Definition
GCST90477908	African	1415	118,304	119,719	0.011	1000 Genomes Project	GWAS Catalog	1.0269	1,159,809	IAVB
GCST90477909	African	536	120,832	121,368	0.004	1000 Genomes Project	GWAS Catalog	1.0155	1,159,804	IIAVB
GCST90477910	African	856	120,644	121,500	0.007	1000 Genomes Project	GWAS Catalog	1.0033	1,159,807	IIIAVB
GCST90477917	African	879	119,623	120,502	0.007	1000 Genomes Project	GWAS Catalog	0.9972	1,159,800	LBBB
GCST90477916	African	1024	119,414	120,438	0.008	1000 Genomes Project	GWAS Catalog	1.0098	1,159,793	RBBB
GCST90477950	African	695	120,312	121,007	0.005	1000 Genomes Project	GWAS Catalog	1.0051	1,159,801	SSS

**Table 5:** Genome structural equation for identifying the latent factor (F1) and univariate SEM parameters in African ancestry data.

Lhs	Op	Rhs	Unstandardized_estimate	Unstandardized_SE	Standardized_est	Standardized_SE	*p*_value
F1	=∼	IAVB	0.20	0.08	0.71	0.28	0.012
F1	=∼	IIAVB	0.16	0.10	0.37	0.23	0.11
F1	=∼	IIIAVB	0.24	0.09	0.93	0.35	0.009
F1	=∼	LBBB	−0.043	0.08	−0.68	1.30	0.60
F1	=∼	RBBB	0.34	0.13	1.07	0.40	0.008
F1	=∼	SSS	0.052	0.08	0.2	0.46	0.54
IAVB	∼∼	IAVB	0.039	0.04	0.49	0.50	0.33
IIAVB	∼∼	IIAVB	0.17	0.061	0.86	0.33	0.009
IIIAVB	∼∼	IIIAVB	0.009	0.06	0.13	0.94	0.88
LBBB	∼∼	LBBB	0.002	0.06	0.53	15.22	0.97
RBBB	∼∼	RBBB	−0.016	0.09	−0.15	0.88	0.85
SSS	∼∼	SSS	0.031	0.06	0.91	1.82	0.61

*Note:* This table shows the correlation between each conduction disorder and the common latent factor (F1), as well as the residual covariance that cannot be explained by the latent factor.

### 3.9. In‐Depth Exploration of the Effect of the *APOL1* Gene on CCDs

As previously reported, chronic kidney disease (CKD) is independently associated with AVB (HR: 1.83; 95% CI: 1.73–1.93) [23]. Additionally, CKD is associated with mortality in patients with LBBB (HR = 2.48, 95% CI = 1.71–3.59, *p* < 0.001) [24]. Furthermore, *APOL1* is strongly associated with CKD in persons of African ancestry. In this study, the TWAS analysis revealed a potential association between CCDs and the APOL1 gene in individuals of European ancestry. We evaluated the relationships among the *APOL1* gene, different stages of CKD (Table 6), and CCDs, using Mendelian randomization to identify causal relationships.

**Table 6:** Information for GWAS datasets of chronic kidney diseases in different stages from African ancestry.

Phenotype	Accession number	Number of cases	Number of controls	Ancestry
Stage I ∼ II CKD	GCST90478531	5454	112,018	African
Stage III CKD	GCST90476127	13,744	104,364	African
Stage IV CKD	GCST90476129	4501	116,141	African
End‐stage renal disease	GCST90476125	4335	116,698	African

**Figure 7:**
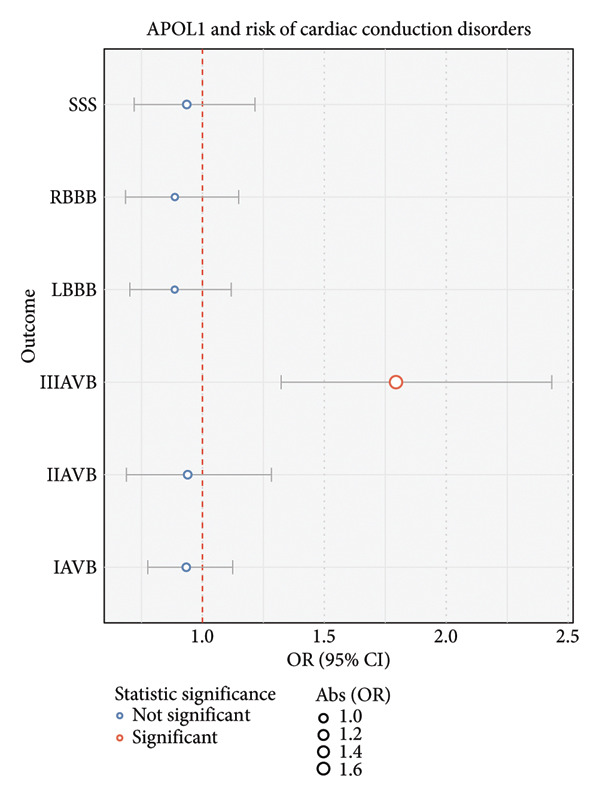
A forest plot reveals the causal relationships between the *APOL1* gene expression and the risk of each type of cardiac conduction disorder in African ancestry data.

**Figure 8:**
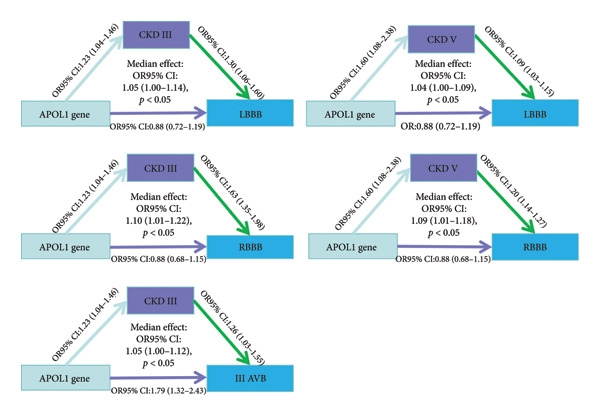
Median analysis of the relationships among the *APOL1* gene, chronic kidney diseases, and each type of cardiac conduction disorder.

The MR analysis (Figure 7) between APOL1 and CCDs among persons of African ancestry indicated that *APOL1* is a direct causal factor for third‐degree AVB (OR: 1.79, 1.32–2.43; *p*‐value < 0.01), and the result of the sensitivity analysis is shown in Table S6. Additionally, *APOL1* increased the risk of Stage III CKD (OR: 1.23, 1.04–1.46; *p*‐value < 0.05) and Stage V CKD (OR: 1.60, 1.08–2.38; *p*‐value < 0.05), and these CKD stages mediated the increased risk of RBBB, LBBB, and IIIAVB (Figure 8). Stage III CKD is a mediator for *APOL1*‐related LBBB, RBBB, and IIIAVB. Stage V CKD is a mediator for *APOL1*‐related LBBB and RBBB. The sensitivity analysis of the median MR analysis is shown in Tables S7–S8. Thus, we found novel connections between CKD, the *APOL1* gene, and CCDs among persons of African ancestry, which would benefit the management of cardiac arrhythmia in patients with CKD.

## 4. Discussion

CCDs are associated with life‐threatening cardiovascular events related to syncope and sudden cardiac death [25]. Therefore, an in‐depth understanding of the genetic basis of various CCDs is beneficial for their management. In this study, we performed an integrated investigation of CCD traits to identify new genetic markers that can accurately predict CCDs. By offering a fresh theoretical framework, the study advances the identification of CCD‐linked genetic loci to inform precision medicine strategies and public health initiatives.

A Genomic‐SEM analysis uncovered shared genetic variants across SSS, BBBs, and all grades of AVB. Genetically, AVB and BBB are strongly positively correlated, whereas SSS is negatively correlated with BBB but positively correlated with AVB; accordingly, AVB and BBB show a distinct genetic architecture. Genomic‐SEM revealed a latent factor that drives CCDs and highlighted previously unreported risk loci. Fine mapping prioritized rs112720315 as a novel CCD‐associated variant, and PheWAS further linked this allele to nonischemic cardiomyopathy. As previously reported, conduction disorders such as LBBB result from NICM [26]. However, previous studies have not analyzed the genetic links between BBBs, especially LBBB, and NICM or heart failure. Our study provides insights into the underlying genetic mechanisms. Furthermore, we performed TWAS, FOCUS, and FUMA analyses and identified causal gene markers related to the risk of conduction disorders. Subsequent LDSC and MR analyses demonstrated the clinical potential of these gene markers in predicting a wide spectrum of conduction disorders.

GWAS data have shown that *FKBP7* is a marker of increased risk and a therapeutic target of atrial fibrillation (AF) [27–29]. Our data exhibited a positive correlation between different degrees of AVB and *FKBP7* gene expression levels, as determined by the TSMR method. First‐degree AVB and AF were reported to have a common underlying mechanism [30]. Our data suggest that *FKBP7* may play a key role in AVB and AF. Moreover, FKBP7 can bind to Ca^2+^ in the endoplasmic reticulum and helps control cellular calcium ion concentrations [31]. An enhanced binding of Ca^2+^ in the endoplasmic reticulum reduces cardiac conduction velocity because of a reduced Ca^2+^ transient [32]. Previous GWASs have reported that *CCDC141* was associated with SAN dysfunction, distal conduction disorders, and pacemaker implantation [33]. Our study confirms the association between *CCDC141* and distal conduction disorders. Our data indicate that, in contrast to *CCDC141*, *ESR2* is related to SSS and AVB, revealing an opposite genetic effect on CCDs. *ESR2*, the principal nuclear receptor for estrogen, downregulates *GLUT4* and accelerates collagen deposition and cardiac fibrosis [34, 35]. Previous studies have shown that AVB and early AF share pathophysiological features, most notably pronounced atrial fibrosis [36, 37]. Thus, *ESR2* is a promising therapeutic target that drives atrial fibrosis and, consequently, CCDs. A previous study reported that *SH3PXD2A* increased the risk of AF [38]. However, LDSC and TSMR analyses in our study did not demonstrate that *SH3PXD2A* was a significant predictor of conduction disorders.

Regarding the transancestry applicability of our model, we tested the model among individuals of African ancestry because the TWAS analysis among persons of European ancestry revealed that the *APOL1* gene showed a significant positive correlation with CCDs, and the *APOL1* gene is related to kidney diseases in people of African ancestry [39]. The common factor model demonstrated better fitting effectiveness among individuals of African ancestry than among those of European ancestry. However, the associations between the common factor and each type of CCD were not significant, indicating that the model should be cautiously applied across ancestry. Moreover, CKD is related to the severity of BBBs [24] and the occurrence of third‐degree AVBs [23]. Our TSMR results demonstrate a novel connection between *APOL1* and IIIAVB. Moreover, Stage 3 and 5 CKDs are mediators of *APOL1*‐related conduction disorders. However, current studies primarily focus on the variants of *APOL1* associated with CVDs, with a limited number of studies examining arrhythmia. Therefore, we provided new insights into the *APOL1* gene’s role in arrhythmia and CKD.

Overall, our study demonstrates genetic correlations between distinct types of CCDs. We also detected a novel locus of a latent factor that modulates several CCDs. SNP rs112720315 on Chromosome 6 shows a significant causal relationship with NICM, a disease closely linked with CCDs. Therefore, this genetic locus has significant clinical value in CCD therapy. Furthermore, we identified multiple genes as potential prediction biomarkers for CCDs.

## 5. Limitations

Although this study revealed genetic correlations between CCDs, the heterogeneity of genetic associations and differences in data format and annotation in different sources may limit the accuracy and interpretability of the model estimates. However, only GWAS summaries are retrieved from the database; specific individual data were not extracted, making it impossible to prevent sample overlap. The GenomicSEM method calculates genetic correlation between traits before formal calculation, and this correlation matrix was considered to minimize the overlap. SCN10A was undetected due to a lack of eQTL data. Markers identified in the European GWAS may not be applicable to other populations, necessitating validation in diverse groups. Fine‐mapping and transcriptomics identified CCD loci, but their biological effects are unknown because of a lack of functional validation. Polygenic factors significantly impact CCDs, but environmental factors are also crucial. However, this method detected markers rarely reported in experimental studies. Genomic SEM identified a latent CCD factor, partially addressing this limitation [40].

### 5.1. Conclusion

This study performed a Genomic‐SEM using CCD GWAS summary data and identified a latent factor that influences CCDs. Based on the results from the SEM and fine‐mapping analyses, we identified a novel lead gene locus associated with the latent factor and nonischemic cardiomyopathy. Furthermore, TWAS, FUMA, FOCUS, LDSC, and TSMR analyses identified four gene markers (*CCDC141, FKBP7, SH3PXD2A, and ESR2*) that are significantly associated with the latent factor. We tested the structural model using African ancestry data and found that *APOL1* was associated with the occurrence of IIIAVB and caused CCDs through CKD.

**Nomenclature**
CCDs: Cardiac conduction disorders
GWAS: Genome‐wide association study
TSMR: Two‐sample Mendelian randomization
LDSC: Linkage disequilibrium score regression
SRMR: Standardized root‐mean‐square residual
TWAS: Transcriptome‐wide association study
FUMA: Functional Mapping and Annotation of Genetic Associations
SSS: Sick sinus syndrome
AVB: Atrioventricular block
LBBB: Left bundle branch block
RBBB: Right bundle branch block
NICM: Nonischemic cardiomyopathy

## Ethics Statement

The authors have nothing to report.

## Consent

The authors have nothing to report.

## Disclosure

All the authors read and approved the final manuscript.

## Conflicts of Interest

The authors declare no conflicts of interest.

## Author Contributions

Yunlong Xia designed the entire study. Tongyu Wang and Xinge Miao performed data collection and analysis. Tongyu Wang and Xinge Miao performed data visualization. Yunlong Xia performed language editing. Tongyu Wang and Xinge Miao wrote the original draft. Tongyu Wang and Xinge Miao contributed equally to this work.

## Funding

This study was supported by funds from the Liaoning Revitalization Talents Program (Grant No. XLYC2002096) and National Key Research and Development Program of China (Grant No. 2022YFC2405002).

## Supporting Information

Figure S1: Regional association plots and LD patterns for rs71208329 and rs112720315. LocusZoom plots of the top lead SNPs identified by GWAS. A. Chromosome 2 locus (lead SNP: rs71208329; position 151.7–152.2 Mb). B. Chromosome 6 locus (lead SNP: rs112720315; position 167.4–167.8 Mb). For each panel, ‐log (*p*) is plotted on the *y*‐axis and genomic position (hg19) on the *x*‐axis. Color coding indicates LD *R* ^2^.

Table S1. Detailed information of the FUMA analysis with annotated leading SNPs, including the position, nearest gene, alleles, effect parameters, and MAF values.

Table S2. TWAS analysis results. This table indicates the correlation between the gene in the GTEx reference panel and the latent factor of interest.

Table S3. FOCUS analysis results. A Bayes colocalization analysis showing the causal genes associated with the latent factor (F1) of interest.

Table S4. This table presents key quality control metrics estimated using LDSC to evaluate the validity of the genome‐wide association study (GWAS) summary statistics before downstream analyses.

Table S5. Results of directionality and pleiotropy tests for gene and cardiac conduction disorders: Mendelian randomization analyses. A. Pleiotropy test results for the Mendelian randomization analysis to detect horizontal pleiotropy in the TSMR analysis; the MR‐Egger intercept test evaluates the presence of directional horizontal pleiotropy. A *p*‐value of < 0.05 indicates significant pleiotropic bias. B. Results of the MR‐Steiger test to identify the direction of MR results; the Steiger test was used to assess the correctness of the assumed causal direction between exposure and outcome. A *p*‐value of < 0.05 suggests that the direction of the MR analysis is correct.

Table S6. Results of the directionality and pleiotropy tests in Mendelian randomization analyses of the association between the *APOL1* gene and cardiac conduction disorders in African ancestry data. A. Pleiotropy test results from the Mendelian randomization analysis to assess horizontal pleiotropy in the two‐sample MR (TSMR) framework; the MR‐Egger intercept test was used to evaluate the presence of directional horizontal pleiotropy. A *p*‐value of < 0.05 indicates significant pleiotropic bias. B. Results of the MR‐Steiger test to determine the causal directionality of the MR estimates; the Steiger test assesses the validity of the assumed causal direction between exposure and outcome. A *p*‐value of < 0.05 supports the correctness of the specified direction in the MR analysis.

Table S7. Results and sensitivity analysis of the Mendelian randomization analysis of the association between the *APOL1* gene and different stages of chronic kidney disease in African ancestry data. A. Mendelian randomization results for the causal relationship between APOL1 and chronic kidney diseases in African ancestry data using the “IVW” method. B. Results of the pleiotropy test of the Mendelian randomization analysis to assess horizontal pleiotropy in the two‐sample MR (TSMR) framework; the MR‐Egger intercept test was used to evaluate the presence of directional horizontal pleiotropy. A *p*‐value of < 0.05 indicates significant pleiotropic bias. C. Results of the MR‐Steiger test to determine the causal directionality of the MR estimates; the Steiger test assesses the validity of the assumed causal direction between exposure and outcome. A *p*‐value of < 0.05 supports the correctness of the specified direction in the MR analysis.

Table S8. Results and sensitivity analysis of Mendelian randomization between different stages of chronic kidney disease and cardiac conduction disorders in African ancestry data. A. Mendelian randomization results for the causal relationship between chronic kidney diseases and cardiac conduction disorders in African ancestry data using the “IVW” method. B. Pleiotropy test results from the Mendelian randomization analysis to assess horizontal pleiotropy in the two‐sample MR (TSMR) framework; the MR‐Egger intercept test was used to evaluate the presence of directional horizontal pleiotropy. A *p*‐value of < 0.05 indicates significant pleiotropic bias. C. Results of the MR‐Steiger test to determine the causal directionality of the MR estimates; the Steiger test assesses the validity of the assumed causal direction between exposure and outcome. A *p*‐value of < 0.05 supports the correctness of the specified direction in the MR analysis.

## Supporting information


**Supporting Information** Additional supporting information can be found online in the Supporting Information section.

## Data Availability

The data that support the findings of this study are available in GWAS Catalog at https://www.ebi.ac.uk/gwas/downloads/summary-statistics, Reference Numbers GCST90475955, GCST90477911, GCST90475980, GCST90475954, GCST90477908, GCST90477909, GCST90477910, GCST90477917, GCST90477916, and GCST90477950. LBBB and RBBB GWAS data were also derived from the following resource available in the public domain: FinnGen, https://r12.finngen.fi/.

## References

[1] Cristina B. , Luca C. , Marco Z. et al., Cardiac Conduction Disorders Due to Acquired or Genetic Causes in Young Adults: A Review of the Current Literature, Journal of the American Heart Association. (2025) 14, no. 9, 10.1161/jaha.124.040274. PMC12184228 40314370

[2] Bingxun L. , Hongxuan X. , and Lin W. , Genetic Insights Into Cardiac Conduction Disorders From Genome-Wide Association Studies, Human Genomics. (2025) 19, no. 1, 10.1186/s40246-025-00732-x. PMC11871809 40022259

[3] Petri H. , Ismo A. , Kjell N. et al., Prognostic Implications of Intraventricular Conduction Delays in a General Population: The Health 2000 Survey, Annals of Medicine. (2015) 47, no. 1, 74–80, 10.3109/07853890.2014.985704, 2-s2.0-84923287275. 25613171

[4] Wenli O. , Peipei L. , Naihui Z. et al., Association Between Cumulative Body Mass Index Exposure and the Risk of Incident Cardiac Conduction Block, Journal of the American Heart Association. (2025) 14, no. 8, 10.1161/jaha.124.039522. PMC12132829 40207481

[5] Julian S H. , Paolo D. A. , Victor N. et al., Frequency of electrocardiogram-defined Cardiac Conduction Disorders in a Multi-Institutional Primary Care Cohort, JACC Advances. (2024) 3, no. 7, 10.1016/j.jacadv.2024.101004. PMC11312782 39130046

[6] Stefan v. D. , Christopher P N. , Zahra R.-E. et al., Leucocyte Telomere Length and Conduction System Ageing, Heart. (2024) 111, no. 7, 314–320, 10.1136/heartjnl-2024-324875. PMC12015050 39689933

[7] Judy R S. , Hector M.-N. , Xin S. et al., Cardiac Conduction System Regeneration Prevents Arrhythmias After Myocardial Infarction, Nature Cardiovascular Research. (2025) 4, no. 2, 163–179, 10.1038/s44161-024-00586-x. PMC11825367 39753976

[8] Hongwei C. , Godfrey L S. , Jules C H. , and Clive H O. , Inhibition of Spontaneous Activity of Rabbit Atrioventricular Node Cells by kb-r7943 and Inhibitors of Sarcoplasmic Reticulum ca(2+) ATPase, Cell Calcium. (2010) 49, 56–65, 10.1016/j.ceca.2010.11.008, 2-s2.0-79951551951. 21163524 PMC3048929

[9] Yuan C. , Hongfei W. , Baijun X. et al., Calmodulin Kinase Ii Inhibition Suppresses Atrioventricular Conduction by Regulating Intracellular ca(2+) Homeostasis, Heart Rhythm. (2024) 22, 1089–1102, 10.1016/j.hrthm.2024.10.022. 39427687

[10] Akiko S. , Taisuke I. , Xavier D. et al., Progressive Atrial Conduction Defects Associated With Bone Malformation Caused by a Connexin-45 Mutation, Journal of the American College of Cardiology. (2017) 70, no. 3, 358–370, 10.1016/j.jacc.2017.05.039, 2-s2.0-85028589417. 28705318

[11] Beixin Julie H. , Penelope B. , and Melvin S. , Ventricular Arrhythmias Involving the his-purkinje System in the Structurally Abnormal Heart, Pacing and Clinical Electrophysiology. (2018) 41, no. 9, 1051–1059, 10.1111/pace.13465, 2-s2.0-85052845496. 30084120 PMC6168393

[12] Per L. , Björn A. , Mikael D. et al., Genetic Variation at the Human Connexin 43 Locus But Not at the Connexin 40 Locus is Associated With Left Bundle Branch Block, Open Heart. (2015) 2, no. 1, 10.1136/openhrt-2014-000187. PMC4395834 25893100

[13] Maria P. , Andrew J A. , Joseph Y. et al., Identification of Key Small Non-Coding MicroRNAs Controlling Pacemaker Mechanisms in the Human Sinus Node, Journal of the American Heart Association. (2020) 9, no. 20, 10.1161/jaha.120.016590. PMC7763385 33059532

[14] Ning L. , Esthela A. , Anuradha K. et al., Altered MicroRNA and mRNA Profiles During Heart Failure in the Human Sinoatrial Node, Scientific Reports. (2021) 11, no. 1, 10.1038/s41598-021-98580-x. PMC8481550 34588502

[15] Patrick T. , Raymond K W. , Omeed M. et al., Multi-Trait Analysis of Genome-Wide Association Summary Statistics Using MTAG, Nature Genetics. (2018) 50, no. 2, 229–237, 10.1038/s41588-017-0009-4, 2-s2.0-85039801269. 29292387 PMC5805593

[16] Kyoko W. , Erdogan T. , Arjen v. B. , and Danielle P. , Functional Mapping and Annotation of Genetic Associations With FUMA, Nature Communications. (2017) 8, no. 1, 10.1038/s41467-017-01261-5, 2-s2.0-85036569680. PMC5705698 29184056

[17] Christian B. , Chris C A S. , Aki S H. , Veikko S. , Samuli R. , and Matti P. , FINEMAP: Efficient Variable Selection Using Summary Data From Genome-Wide Association Studies, Bioinformatics. (2016) 32, no. 10, 1493–1501, 10.1093/bioinformatics/btw018, 2-s2.0-84970015769. 26773131 PMC4866522

[18] Alexander G. , Arthur K. , Huwenbo S. et al., Integrative Approaches for Large-Scale Transcriptome-Wide Association Studies, Nature Genetics. (2016) 48, no. 3, 245–252, 10.1038/ng.3506, 2-s2.0-84959547986. 26854917 PMC4767558

[19] Brendan B.-S. , Hilary F. , Verneri A. et al., An Atlas of Genetic Correlations Across Human Diseases and Traits, Nature Genetics. (2015) 47, no. 11, 1236–1241, 10.1038/ng.3406, 2-s2.0-85000692305. 26414676 PMC4797329

[20] Kevin N. and Braxton M. , A Guide to Understanding Mendelian Randomization Studies, Arthritis Care & Research. (2024) 76, no. 11, 1451–1460, 10.1002/acr.25400. 39030941 PMC11833605

[21] Rasheed A G. , Ifeoma U. , Samuel A. et al., Apol1 Bi- and Monoallelic Variants and Chronic Kidney Disease in West Africans, New England Journal of Medicine. (2024) 392, 10.1056/NEJMoa2404211.

[22] Constance B H. , The Mislaid Clue to apol1 Kidney Disease Prevention in Blacks, Journal of Human Hypertension. (2025) 39, no. 6, 389–391, 10.1038/s41371-025-01024-6. 40335636

[23] Hannah K W.-K. , Anne Storgaard N. , Nicholas C. et al., The Association Between Chronic Kidney Disease and Third-Degree Atrioventricular Block: A Danish Nationwide Study, JACC: Clinical Electrophysiology. (2024) 11, 10.1016/j.jacep.2024.10.007. 39708039

[24] Hui-Chun H. , Chun-Kai C. , Yen-Bin L. , Chien-Hua H. , and Kuo-Liong C. , Risk of Mortality Associated to Chronic Kidney Disease in Patients With Complete Left Bundle Branch Block, Scientific Reports. (2024) 14, no. 1, 10.1038/s41598-024-68826-5. PMC11297155 39095533

[25] Fred M K. , Mark H S. , Coletta B. et al., ACC/AHA/HRS Guideline on the Evaluation and Management of Patients With Bradycardia and Cardiac Conduction Delay: A Report of the American College of Cardiology/American Heart Association Task Force on Clinical Practice Guidelines and the Heart Rhythm Society, Circulation. (2018) 140, e382–e482. 30586772 10.1161/CIR.0000000000000628

[26] Giuseppe D S. , Annamaria D. B. , Massimo Z. et al., Prevalence, Clinical and Instrumental Features of Left Bundle Branch Block-Induced Cardiomyopathy: The Climb Registry, ESC Heart Fail. (2021) 8, no. 6, 5589–5593, 10.1002/ehf2.13568. 34510787 PMC8712772

[27] Yalin Y. , Xin Z. , Wenjing Z. , Zhaoyu R. , and Bin L. , A Cross-Tissue Transcriptome-Wide Association Study Identifies Novel Susceptibility Genes for Atrial Fibrillation, Journal of Arrhythmia. (2025) 41, no. 3, 10.1002/joa3.70097. PMC12099065 40416952

[28] Zuodong N. , Yunying H. , Haocheng L. et al., Novel Drug Targets for Atrial Fibrillation Identified Through Mendelian Randomization Analysis of the Blood Proteome, Cardiovascular Drugs and Therapy. (2023) 38, no. 6, 1215–1222, 10.1007/s10557-023-07467-8. 37212950

[29] Jeffrey H. , Shamone G.-P. , Gregory T. et al., Genetic Control of Left Atrial Gene Expression Yields Insights Into the Genetic Susceptibility for Atrial Fibrillation, Circulation: Genomic and Precision Medicine. (2018) 11, no. 3, 10.1161/circgen.118.002107, 2-s2.0-85049127836. PMC5858469 29545482

[30] Florian S. , Sven K. , Ivan Z. et al., First-Degree Atrioventricular Block in Patients With Atrial Fibrillation and Atrial Flutter: The Prevalence of Intra-Atrial Conduction Delay, Journal of Interventional Cardiac Electrophysiology. (2020) 61, no. 2, 421–425, 10.1007/s10840-020-00838-3. 32734408 PMC8324594

[31] Chen C. , Hui M. , Ying W. , and Huaifeng M. , Binding of fkbp23 to BiP in ER Shown by Gel Filtration Chromatography, Zeitschrift Fuer Naturforschung, C: Journal of Biosciences. (2007) 62, no. 1-2, 133–137, 10.1515/znc-2007-1-222, 2-s2.0-34248363175. 17425118

[32] Carlos H P. , Hiroki K. , Edward J O. V. et al., Colitis Induced Ventricular Alternans Increases the Risk for Ventricular Arrhythmia, Journal of Molecular and Cellular Cardiology. (2025) 204, 68–78, 10.1016/j.yjmcc.2025.05.004. 40409406 PMC12355935

[33] Lu-Chen W. , Joel T R. , Sean J J. et al., The Impact of Common and Rare Genetic Variants on Bradyarrhythmia Development, Nature Genetics. (2025) 57, no. 1, 53–64, 10.1038/s41588-024-01978-2. 39747593 PMC11735381

[34] Yining D. , Rumeng C. , Zhiwei Z. et al., Identification of Novel Therapeutic Targets for Atrial Fibrillation Through Mendelian Randomization Analysis of Druggable Genes, Experimental Gerontology. (2025) 207, 10.1016/j.exger.2025.112797. 40451550

[35] Mahdi F. , Ramin A. S. , Vahid S. S. , and Pouran K. , Cardiac Fibrosis and down Regulation of glut4 in Experimental Diabetic Cardiomyopathy are Ameliorated by Chronic Exposures to Intermittent Altitude, Journal of Cardiovascular and Thoracic Research. (2016) 8, no. 1, 26–33, 10.15171/jcvtr.2016.05. 27069564 PMC4827136

[36] Zain S A. , Abdullah B. , Purav V. et al., PR Prolongation as a Predictor of Atrial Fibrillation Onset: A State-of-the-Art Review, Current Problems in Cardiology. (2024) 49, no. 4, 10.1016/j.cpcardiol.2024.102469. 38369207

[37] Ryuichi K. , Ai G. , Akira T. et al., Characterization of Remodeling Processes in the Atria of Atrioventricular Block Dogs: Utility as an Early-Stage Atrial Fibrillation Model, Journal of Pharmacological Sciences. (2024) 156, no. 1, 19–29, 10.1016/j.jphs.2024.06.004. 39068031

[38] Shih-Yin C. , Yu-Chia C. , Ting-Yuan L. et al., Novel Genes Associated With Atrial Fibrillation and the Predictive Models for AF Incorporating Polygenic Risk Score and PheWAS-Derived Risk Factors, Canadian Journal of Cardiology. (2024) 40, no. 11, 2117–2127, 10.1016/j.cjca.2024.07.029. 39142603

[39] Akinlolu O O. , Dwomoa A. , Kate B. et al., Apol1 Kidney Disease: Conclusions From a Kidney Disease: Improving Global Outcomes (KDIGO) Controversies Conference, Kidney International. (2025) 108, no. 5, 763–779, 10.1016/j.kint.2025.05.017. 40582702 PMC13266834

[40] Wang T. , Miao X. , and Xia Y. , Integration of Genomic Structural Equation and Post-GWAS Analysis Reveal the Risk Gene Loci and Sensitive Genes for Cardiac Conduction Block Risk, Research Square. (2025) https://www.researchsquare.com/article/rs-6928678/v1. 10.1155/genr/1063531 PMC12801132 41541644

