# Supplementary material for: Genomic Structural Equation Modeling Combined With Post‐GWAS Analysis Identifies Two Risk Gene Loci and Functionally Sensitive Genes Associated With Cardiac Conduction Block
Source: Genet Res (Camb). 2026 Jan 14;2026:1063531. doi: 10.1155/genr/1063531 (PMC12801132; doi:10.1155/genr/1063531)
Supplement: Supplementary file 1 — Supporting Information Additional supporting information can be found online in the Supporting Information section. [file GENR-2026-1063531-s001.zip › Table S1.docx]

| rsID | CHR | BP | other_allele | effect_allele | MAF | p | beta | se | nearestGene | distance | function |
| --- | --- | --- | --- | --- | --- | --- | --- | --- | --- | --- | --- |
| rs71208329 | 2 | 151915828 | T | C | 0.03 | 6.51e-11 | 0.04 | 0.007 | AC023469.1 | 10539 | intergenic |
| rs13031826 | 2 | 179756602 | T | A | 0.12 | 9.73e-09 | -0.05 | 0.009 | CCDC141 | 0 | intronic |
| rs2634071 | 4 | 111669220 | C | T | 0.21 | 7.79e-09 | 0.06 | 0.011 | RP11-777N19.1 | 46338 | intergenic |
| rs112720315 | 6 | 167600420 | C | T | 0.02 | 1.62e-08 | -0.05 | 0.009 | TCP10L2 | 0 | intronic |
