# Supplementary material for: Genomic Structural Equation Modeling Combined With Post‐GWAS Analysis Identifies Two Risk Gene Loci and Functionally Sensitive Genes Associated With Cardiac Conduction Block
Source: Genet Res (Camb). 2026 Jan 14;2026:1063531. doi: 10.1155/genr/1063531 (PMC12801132; doi:10.1155/genr/1063531)
Supplement: Supplementary file 1 — Supporting Information Additional supporting information can be found online in the Supporting Information section. [file GENR-2026-1063531-s001.zip › Table S2.docx]

| ID | PANEL | NSNP | NWGT | MODEL | MODELCV.R2 | MODELCV.PV | TWAS.Z | TWAS.P | FDR |
| --- | --- | --- | --- | --- | --- | --- | --- | --- | --- |
| FKBP7 | sCCA2 | 463 | 43 | enet | 0.29 | 2.6e-24 | 5.43 | 5.52e-08 | 0 |
| FKBP7 | sCCA3 | 463 | 34 | enet | 0.48 | 3.9e-45 | -4.73 | 2.26e-06 | 0.04 |
| SYNE2 | sCCA2 | 363 | 12 | lasso | 0.086 | 1.2e-07 | 4.47 | 7.71e-06 | 0.05 |
| FKBP7 | sCCA1 | 463 | 29 | enet | 0.48 | 6.8e-45 | -4.41 | 1.02e-05 | 0.05 |
| WASHC4 | sCCA3 | 647 | 27 | enet | 0.15 | 1.9e-12 | 4.47 | 7.73e-06 | 0.05 |
| SENP2 | sCCA1 | 354 | 38 | enet | 0.23 | 3.8e-19 | 4.48 | 7.37e-06 | 0.05 |
| RP11-474P2.6 | sCCA3 | 344 | 26 | enet | 0.062 | 6.5e-06 | -4.45 | 8.73e-06 | 0.05 |
| CEP68 | sCCA1 | 427 | 5 | lasso | 0.7 | 2.7e-82 | -4.31 | 1.62e-05 | 0.07 |
| CEP68 | sCCA2 | 427 | 6 | lasso | 0.58 | 5e-59 | 4.29 | 1.81e-05 | 0.07 |
| PRKRA | sCCA2 | 467 | 7 | lasso | 0.03 | 0.0014 | 4.32 | 1.57e-05 | 0.07 |
| PTK2 | sCCA1 | 408 | 1 | top1 | 0.12 | 1.9e-10 | 4.22 | 2.39e-05 | 0.08 |
| CEP68 | sCCA3 | 427 | 6 | lasso | 0.47 | 5.9e-44 | 4.07 | 4.71e-05 | 0.09 |
| PSD | sCCA2 | 344 | 8 | lasso | 0.037 | 0.00046 | 4.13 | 3.65e-05 | 0.09 |
| NT5C2 | sCCA1 | 350 | 7 | lasso | 0.22 | 1.2e-18 | -4.17 | 3.02e-05 | 0.09 |
| SH3PXD2A | sCCA3 | 423 | 1 | top1 | 0.048 | 6.5e-05 | 4.11 | 3.88e-05 | 0.09 |
| PRRX1 | sCCA3 | 450 | 38 | enet | 0.071 | 1.5e-06 | -4.06 | 4.96e-05 | 0.09 |
| RAB1A | sCCA1 | 437 | 7 | lasso | 0.1 | 7.9e-09 | 4.09 | 4.32e-05 | 0.09 |
| CNKSR1 | sCCA1 | 439 | 50 | enet | 0.71 | 1.2e-83 | -4.06 | 4.92e-05 | 0.09 |
| CNKSR1 | sCCA3 | 439 | 46 | enet | 0.7 | 6e-81 | 4.02 | 5.75e-05 | 0.09 |
| BORCS7 | sCCA1 | 344 | 18 | enet | 0.76 | 4.3e-97 | 4.01 | 6.11e-05 | 0.09 |
| BCL2 | sCCA2 | 499 | 1 | top1 | 0.13 | 2.9e-11 | -4 | 6.28e-05 | 0.09 |
| ZNF69 | sCCA3 | 318 | 19 | enet | 0.0015 | 0.23 | 4.01 | 5.98e-05 | 0.09 |
| AS3MT | sCCA3 | 344 | 35 | enet | 0.85 | 8e-127 | 4.15 | 3.29e-05 | 0.09 |
| AS3MT | sCCA2 | 344 | 30 | enet | 0.72 | 1.6e-86 | -4.12 | 3.84e-05 | 0.09 |
| AS3MT | sCCA1 | 344 | 13 | lasso | 0.85 | 1.1e-126 | 4.02 | 5.77e-05 | 0.09 |
| CTD-2555O16.4 | sCCA3 | 492 | 1 | top1 | 0.069 | 1.9e-06 | -4.07 | 4.8e-05 | 0.09 |
| ERICD | sCCA1 | 369 | 14 | enet | 0.2 | 7.1e-17 | -3.98 | 6.76e-05 | 0.09 |
| CNNM2 | sCCA1 | 342 | 7 | lasso | 0.21 | 2.2e-17 | -3.96 | 7.4e-05 | 0.1 |
| BORCS7 | sCCA2 | 344 | 5 | lasso | 0.63 | 2e-67 | -3.96 | 7.47e-05 | 0.1 |
| NEURL1 | sCCA3 | 367 | 31 | enet | 0.018 | 0.011 | 3.91 | 9.32e-05 | 0.12 |
| CTA-204B4.2 | sCCA1 | 386 | 1 | top1 | 0.04 | 0.00026 | 3.9 | 9.55e-05 | 0.12 |
| BRWD1 | sCCA1 | 673 | 56 | enet | 0.1 | 8.5e-09 | 3.86 | 0.000113 | 0.13 |
| MAP3K7CL | sCCA1 | 426 | 6 | lasso | 0.13 | 3.3e-11 | 3.84 | 0.000122 | 0.14 |
| SENP2 | sCCA3 | 354 | 1 | top1 | 0.08 | 3.3e-07 | -3.8 | 0.000142 | 0.15 |
| GPRC5C | sCCA1 | 505 | 18 | enet | 0.31 | 4.6e-26 | -3.8 | 0.000143 | 0.15 |
| DMWD | sCCA2 | 394 | 6 | lasso | 0.16 | 9.4e-14 | 3.75 | 0.000174 | 0.18 |
| PRRX1 | sCCA1 | 450 | 18 | enet | 0.17 | 6.1e-14 | 3.74 | 0.000187 | 0.19 |
| DFNB59 | sCCA2 | 467 | 17 | enet | 0.043 | 0.00016 | 3.73 | 0.000189 | 0.19 |
| MYL12A | sCCA2 | 489 | 47 | enet | 0.17 | 2.1e-14 | -3.69 | 0.000221 | 0.21 |
| SMC3 | sCCA1 | 433 | 15 | enet | 0.021 | 0.0059 | -3.67 | 0.000246 | 0.21 |
| APPL2 | sCCA2 | 546 | 5 | lasso | 0.11 | 2.1e-09 | 3.66 | 0.000248 | 0.21 |
| PPARGC1B | sCCA2 | 617 | 31 | enet | 0.063 | 5.5e-06 | 3.68 | 0.000235 | 0.21 |
| BORCS7 | sCCA3 | 344 | 49 | enet | 0.69 | 1.1e-78 | 3.66 | 0.000251 | 0.21 |
| ERICD | sCCA2 | 369 | 1 | top1 | 0.071 | 1.4e-06 | -3.68 | 0.000234 | 0.21 |
| TRIM8 | sCCA2 | 362 | 10 | lasso | 0.068 | 2.2e-06 | 3.65 | 0.000259 | 0.22 |
| FUBP1 | sCCA3 | 332 | 29 | enet | 0.21 | 1.1e-17 | 3.63 | 0.000284 | 0.23 |
| FUBP1 | sCCA1 | 332 | 34 | enet | 0.3 | 6.5e-26 | 3.61 | 0.000307 | 0.25 |
| SUFU | sCCA1 | 333 | 9 | lasso | 0.14 | 3.9e-12 | -3.55 | 0.000378 | 0.27 |
| ALDH1L2 | sCCA3 | 680 | 12 | lasso | 0.23 | 2e-19 | -3.56 | 0.000366 | 0.27 |
| ALDH1L2 | sCCA1 | 680 | 32 | enet | 0.21 | 3.2e-17 | 3.55 | 0.000378 | 0.27 |
| YAP1 | sCCA1 | 432 | 1 | top1 | 0.094 | 2.8e-08 | 3.57 | 0.000354 | 0.27 |
| RNF207 | sCCA2 | 450 | 6 | lasso | 0.33 | 4.7e-28 | 3.57 | 0.000353 | 0.27 |
| TMEM150A | sCCA3 | 475 | 1 | top1 | 0.018 | 0.011 | 3.55 | 0.000385 | 0.27 |
| POMGNT1 | sCCA3 | 343 | 1 | top1 | 0.05 | 5e-05 | -3.49 | 0.000486 | 0.3 |
| RNF122 | sCCA2 | 261 | 11 | enet | 0.038 | 0.00039 | -3.49 | 0.000482 | 0.3 |
| CYP17A1 | sCCA3 | 345 | 1 | top1 | 0.076 | 5.8e-07 | 3.49 | 0.000482 | 0.3 |
| CD52 | sCCA2 | 395 | 11 | lasso | 0.19 | 1.6e-15 | -3.48 | 0.000494 | 0.3 |
| DMWD | sCCA1 | 394 | 10 | lasso | 0.28 | 6.9e-24 | 3.52 | 0.000425 | 0.3 |
| DMWD | sCCA3 | 394 | 9 | lasso | 0.25 | 2.4e-21 | -3.48 | 0.000494 | 0.3 |
| C12orf75 | sCCA3 | 548 | 25 | enet | 0.17 | 6.8e-14 | -3.51 | 0.000451 | 0.3 |
| STAG3L2 | sCCA3 | 97 | 5 | lasso | 0.2 | 2.1e-16 | 3.51 | 0.000447 | 0.3 |
| BRWD1-AS2 | sCCA3 | 675 | 54 | enet | 0.47 | 3.3e-44 | 3.48 | 0.000504 | 0.31 |
| RP11-474P2.6 | sCCA2 | 344 | 17 | enet | 0.028 | 0.002 | 3.46 | 0.000542 | 0.32 |
| CROT | sCCA2 | 428 | 4 | lasso | 0.023 | 0.0043 | 3.24 | 0.001185 | 0.34 |
| CROT | sCCA3 | 428 | 1 | top1 | 0.066 | 3.1e-06 | -3.15 | 0.001616 | 0.34 |
| GIPR | sCCA2 | 374 | 28 | enet | 0.019 | 0.0087 | -3.29 | 0.000996 | 0.34 |
| GIPR | sCCA3 | 374 | 24 | enet | 0.012 | 0.03 | -3.15 | 0.00163 | 0.34 |
| SYNE2 | sCCA1 | 363 | 24 | enet | 0.25 | 1.5e-20 | 3.13 | 0.00175 | 0.34 |
| TMEM206 | sCCA2 | 467 | 7 | lasso | 0.25 | 3.3e-21 | 3.28 | 0.001037 | 0.34 |
| PLPP1 | sCCA3 | 330 | 13 | enet | 0.036 | 0.00049 | 3.18 | 0.001493 | 0.34 |
| IFT80 | sCCA2 | 374 | 6 | lasso | 0.061 | 7.6e-06 | -3.2 | 0.001379 | 0.34 |
| RAB27A | sCCA2 | 444 | 1 | top1 | 0.14 | 4.5e-12 | 3.15 | 0.00163 | 0.34 |
| HLTF | sCCA2 | 566 | 17 | lasso | 0.18 | 8.3e-15 | 3.12 | 0.001824 | 0.34 |
| LIMS2 | sCCA3 | 364 | 43 | enet | 0.095 | 2.2e-08 | 3.28 | 0.00104 | 0.34 |
| TMEM38A | sCCA3 | 379 | 21 | enet | 0.09 | 5.9e-08 | 3.31 | 0.000934 | 0.34 |
| FNDC3B | sCCA2 | 460 | 36 | enet | 0.014 | 0.023 | -3.38 | 0.000718 | 0.34 |
| FNDC3B | sCCA1 | 460 | 17 | enet | 0.054 | 2.4e-05 | -3.15 | 0.00161 | 0.34 |
| BLVRB | sCCA3 | 377 | 20 | enet | 0.057 | 1.6e-05 | 3.41 | 0.000656 | 0.34 |
| MTHFD1 | sCCA1 | 498 | 1 | top1 | 0.61 | 4.4e-64 | -3.14 | 0.00171 | 0.34 |
| MTHFD1 | sCCA2 | 498 | 1 | top1 | 0.36 | 7.2e-32 | -3.14 | 0.00171 | 0.34 |
| MTHFD1 | sCCA3 | 498 | 1 | top1 | 0.6 | 7.8e-63 | 3.14 | 0.00171 | 0.34 |
| NDRG4 | sCCA3 | 479 | 44 | enet | 0.15 | 1.1e-12 | -3.14 | 0.00166 | 0.34 |
| DMPK | sCCA1 | 386 | 6 | lasso | 0.42 | 1.6e-37 | 3.2 | 0.001389 | 0.34 |
| PRX | sCCA1 | 375 | 20 | enet | 0.19 | 1.9e-15 | 3.39 | 0.000696 | 0.34 |
| PRX | sCCA2 | 375 | 1 | top1 | 0.18 | 3.7e-15 | -3.32 | 0.000902 | 0.34 |
| CDK6 | sCCA1 | 319 | 1 | top1 | 0.087 | 9.6e-08 | 3.2 | 0.00135 | 0.34 |
| GBF1 | sCCA1 | 298 | 11 | enet | 0.0041 | 0.13 | -3.28 | 0.00104 | 0.34 |
| PSMD9 | sCCA3 | 346 | 11 | enet | 0.075 | 7.3e-07 | 3.13 | 0.00174 | 0.34 |
| COQ5 | sCCA2 | 406 | 5 | lasso | 0.56 | 4.7e-56 | 3.31 | 0.00092 | 0.34 |
| COQ5 | sCCA1 | 406 | 1 | top1 | 0.76 | 4.9e-95 | 3.29 | 0.001016 | 0.34 |
| COQ5 | sCCA3 | 406 | 1 | top1 | 0.75 | 1.1e-93 | -3.29 | 0.00102 | 0.34 |
| MAN2A1 | sCCA3 | 427 | 27 | enet | 0.017 | 0.012 | 3.12 | 0.001799 | 0.34 |
| WIPF1 | sCCA2 | 330 | 10 | lasso | 0.25 | 6e-21 | -3.18 | 0.00146 | 0.34 |
| ASH1L | sCCA1 | 223 | 1 | top1 | 0.092 | 3.7e-08 | -3.22 | 0.0013 | 0.34 |
| PLEKHM2 | sCCA1 | 482 | 6 | lasso | 0.26 | 3.5e-22 | 3.16 | 0.00157 | 0.34 |
| PRRC2C | sCCA1 | 591 | 8 | lasso | 0.23 | 7.8e-19 | -3.19 | 0.00141 | 0.34 |
| STMN1 | sCCA3 | 423 | 1 | top1 | 0.043 | 0.00015 | -3.13 | 0.00177 | 0.34 |
| GPAM | sCCA3 | 467 | 30 | enet | 0.042 | 2e-04 | 3.42 | 0.000621 | 0.34 |
| EPC1 | sCCA2 | 310 | 33 | enet | 0.029 | 0.0016 | -3.12 | 0.00183 | 0.34 |
| STAMBP | sCCA2 | 370 | 25 | enet | 0.2 | 5.5e-17 | -3.12 | 0.00183 | 0.34 |
| MSTO1 | sCCA1 | 219 | 4 | lasso | 0.13 | 3.6e-11 | 3.25 | 0.00117 | 0.34 |
| GPR108 | sCCA1 | 473 | 6 | lasso | 0.69 | 7.7e-79 | -3.25 | 0.00117 | 0.34 |
| GPR108 | sCCA2 | 473 | 32 | enet | 0.58 | 6.5e-59 | 3.22 | 0.001292 | 0.34 |
| GPR108 | sCCA3 | 473 | 5 | lasso | 0.54 | 7.8e-54 | -3.12 | 0.0018 | 0.34 |
| CAPNS1 | sCCA1 | 332 | 2 | lasso | 0.09 | 5.4e-08 | -3.19 | 0.001402 | 0.34 |
| HSPA2 | sCCA2 | 497 | 26 | enet | 0.057 | 1.6e-05 | -3.31 | 0.000934 | 0.34 |
| CALU | sCCA2 | 352 | 7 | lasso | 0.095 | 2.2e-08 | 3.38 | 0.000714 | 0.34 |
| MYO1B | sCCA1 | 383 | 20 | enet | 0.17 | 1.6e-14 | 3.29 | 0.00101 | 0.34 |
| CEP85 | sCCA1 | 427 | 35 | enet | 0.59 | 1.5e-61 | -3.18 | 0.00147 | 0.34 |
| CEP85 | sCCA3 | 427 | 30 | enet | 0.53 | 1.8e-52 | 3.14 | 0.0017 | 0.34 |
| RRP8 | sCCA3 | 632 | 6 | lasso | 0.2 | 2.1e-16 | -3.27 | 0.00107 | 0.34 |
| RRP8 | sCCA2 | 632 | 4 | lasso | 0.33 | 3.3e-28 | -3.24 | 0.001187 | 0.34 |
| RRP8 | sCCA1 | 632 | 3 | lasso | 0.41 | 4.6e-37 | 3.22 | 0.001287 | 0.34 |
| DAP3 | sCCA1 | 221 | 5 | lasso | 0.082 | 2.2e-07 | 3.27 | 0.00106 | 0.34 |
| VAV3 | sCCA1 | 465 | 7 | lasso | 0.37 | 9.4e-33 | -3.23 | 0.00122 | 0.34 |
| IL6ST | sCCA2 | 416 | 1 | top1 | 0.036 | 0.00054 | 3.13 | 0.00172 | 0.34 |
| WDR33 | sCCA1 | 372 | 6 | lasso | 0.13 | 2.6e-11 | 3.17 | 0.00153 | 0.34 |
| DENND4C | sCCA2 | 621 | 47 | enet | 0.15 | 3e-12 | 3.31 | 0.000946 | 0.34 |
| AOX1 | sCCA3 | 477 | 7 | lasso | 0.025 | 0.0032 | 3.21 | 0.00135 | 0.34 |
| TMTC3 | sCCA1 | 290 | 48 | enet | 0.33 | 4.8e-28 | 3.17 | 0.001521 | 0.34 |
| SH3BGRL3 | sCCA1 | 415 | 4 | lasso | 0.29 | 8.5e-25 | -3.17 | 0.00152 | 0.34 |
| XPR1 | sCCA3 | 440 | 1 | top1 | 0.059 | 1.1e-05 | -3.43 | 0.000611 | 0.34 |
| DUSP10 | sCCA2 | 474 | 29 | enet | 0.044 | 0.00014 | -3.18 | 0.001475 | 0.34 |
| TRPC1 | sCCA3 | 382 | 1 | top1 | 0.033 | 0.00086 | -3.18 | 0.00147 | 0.34 |
| DOK3 | sCCA1 | 260 | 1 | top1 | 0.38 | 3.2e-33 | 3.36 | 0.000787 | 0.34 |
| DOK3 | sCCA3 | 260 | 1 | top1 | 0.25 | 2.7e-21 | 3.36 | 0.000787 | 0.34 |
| TIAL1 | sCCA2 | 471 | 18 | enet | 0.025 | 0.003 | -3.17 | 0.00151 | 0.34 |
| RWDD2B | sCCA1 | 428 | 9 | lasso | 0.47 | 1.7e-43 | 3.33 | 0.000871 | 0.34 |
| RWDD2B | sCCA3 | 428 | 8 | enet | 0.21 | 4.6e-17 | 3.3 | 0.000968 | 0.34 |
| RWDD2B | sCCA2 | 428 | 9 | lasso | 0.35 | 1.2e-30 | 3.28 | 0.00104 | 0.34 |
| MAP3K7CL | sCCA3 | 426 | 1 | top1 | 0.054 | 2.6e-05 | -3.36 | 0.000766 | 0.34 |
| BACH1 | sCCA3 | 450 | 36 | enet | 0.14 | 5.3e-12 | 3.22 | 0.001279 | 0.34 |
| BACH1 | sCCA1 | 450 | 34 | enet | 0.13 | 2.2e-11 | 3.14 | 0.001676 | 0.34 |
| GPR153 | sCCA2 | 447 | 1 | top1 | 0.12 | 1.3e-10 | 3.44 | 0.000581 | 0.34 |
| PEA15 | sCCA3 | 513 | 30 | enet | 0.026 | 0.0028 | 3.14 | 0.0017 | 0.34 |
| MAPKAPK2 | sCCA1 | 381 | 1 | top1 | 0.12 | 3.1e-10 | 3.21 | 0.00131 | 0.34 |
| FBXO41 | sCCA2 | 292 | 9 | lasso | 0.21 | 1.7e-17 | -3.37 | 0.000751 | 0.34 |
| FBXO41 | sCCA1 | 292 | 16 | enet | 0.17 | 1.5e-14 | -3.15 | 0.00164 | 0.34 |
| PCOLCE2 | sCCA3 | 480 | 1 | top1 | 0.11 | 8.8e-10 | -3.18 | 0.00147 | 0.34 |
| RHOBTB3 | sCCA2 | 402 | 45 | enet | 0.014 | 0.024 | 3.17 | 0.001547 | 0.34 |
| SUN1 | sCCA3 | 314 | 4 | lasso | 0.18 | 3.8e-15 | -3.34 | 0.000847 | 0.34 |
| DPCD | sCCA1 | 307 | 12 | lasso | 0.13 | 3.2e-11 | 3.17 | 0.0015 | 0.34 |
| NOLC1 | sCCA2 | 275 | 4 | lasso | 0.021 | 0.0061 | -3.18 | 0.00146 | 0.34 |
| FAM227B | sCCA3 | 367 | 24 | enet | 0.18 | 7.9e-15 | 3.21 | 0.00135 | 0.34 |
| MAP1A | sCCA3 | 266 | 9 | lasso | 0.067 | 2.8e-06 | 3.17 | 0.00153 | 0.34 |
| TMEM182 | sCCA2 | 418 | 5 | lasso | 0.037 | 0.00046 | -3.2 | 0.00138 | 0.34 |
| TRIAP1 | sCCA3 | 390 | 1 | top1 | 0.076 | 6.2e-07 | 3.28 | 0.00102 | 0.34 |
| TRIAP1 | sCCA1 | 390 | 1 | top1 | 0.18 | 6.1e-15 | 3.28 | 0.001023 | 0.34 |
| TRMT112 | sCCA1 | 359 | 24 | enet | 0.55 | 9e-55 | 3.22 | 0.001283 | 0.34 |
| TRMT112 | sCCA3 | 359 | 33 | enet | 0.48 | 1.9e-45 | 3.22 | 0.00129 | 0.34 |
| TRMT112 | sCCA2 | 359 | 10 | lasso | 0.32 | 1.4e-27 | -3.14 | 0.001707 | 0.34 |
| CHST2 | sCCA1 | 547 | 1 | top1 | 0.22 | 6.6e-18 | -3.31 | 0.000931 | 0.34 |
| RPL23P2 | sCCA3 | 426 | 34 | enet | 0.39 | 4.3e-35 | 3.16 | 0.00157 | 0.34 |
| RPL23P2 | sCCA2 | 426 | 34 | enet | 0.41 | 1.7e-36 | 3.15 | 0.00163 | 0.34 |
| EVI2B | sCCA2 | 368 | 1 | top1 | 0.038 | 0.00035 | -3.39 | 0.000689 | 0.34 |
| SULF2 | sCCA2 | 527 | 11 | lasso | 0.15 | 1.8e-12 | 3.3 | 0.000972 | 0.34 |
| WDR5B | sCCA2 | 460 | 39 | enet | 0.31 | 1e-26 | 3.2 | 0.00137 | 0.34 |
| MSTO2P | sCCA1 | 236 | 4 | lasso | 0.36 | 2.8e-31 | 3.39 | 0.000692 | 0.34 |
| MSTO2P | sCCA3 | 236 | 3 | lasso | 0.34 | 7.1e-30 | -3.39 | 0.000705 | 0.34 |
| MSTO2P | sCCA2 | 236 | 22 | enet | 0.12 | 2e-10 | -3.36 | 0.000773 | 0.34 |
| HMGN1 | sCCA1 | 668 | 1 | top1 | 0.076 | 6e-07 | -3.24 | 0.001201 | 0.34 |
| IGHA1 | sCCA1 | 71 | 5 | lasso | 0.035 | 0.00059 | 3.15 | 0.00165 | 0.34 |
| RP1-257A7.4 | sCCA1 | 523 | 36 | enet | 0.44 | 9.8e-41 | 3.27 | 0.00109 | 0.34 |
| CCDC7 | sCCA2 | 317 | 52 | enet | 0.43 | 2e-39 | -3.12 | 0.00179 | 0.34 |
| VAMP2 | sCCA3 | 439 | 30 | enet | 0.12 | 1.6e-10 | 3.21 | 0.001345 | 0.34 |
| RPL4P6 | sCCA2 | 268 | 9 | enet | 0.056 | 1.9e-05 | 3.33 | 0.000853 | 0.34 |
| AF129075.5 | sCCA1 | 427 | 5 | lasso | 0.021 | 0.0063 | 3.28 | 0.00104 | 0.34 |
| C12orf75 | sCCA2 | 548 | 13 | lasso | 0.26 | 1.3e-21 | 3.24 | 0.001177 | 0.34 |
| ARPIN | sCCA2 | 413 | 21 | enet | 0.35 | 2.6e-30 | 3.25 | 0.00117 | 0.34 |
| ZNF585B | sCCA3 | 277 | 54 | enet | 0.45 | 6.1e-41 | 3.17 | 0.00154 | 0.34 |
| ZNF585B | sCCA1 | 277 | 47 | enet | 0.44 | 5.6e-40 | 3.15 | 0.001614 | 0.34 |
| MGC16275 | sCCA3 | 559 | 1 | top1 | 0.005 | 0.11 | -3.18 | 0.001469 | 0.34 |
| NRAV | sCCA1 | 397 | 1 | top1 | 0.15 | 1.5e-12 | -3.28 | 0.001023 | 0.34 |
| SMURF2P1 | sCCA2 | 292 | 1 | top1 | 0.055 | 2e-05 | 3.39 | 0.000689 | 0.34 |
| RP11-1334A24.6 | sCCA1 | 263 | 1 | top1 | 0.18 | 4e-15 | 3.36 | 0.000787 | 0.34 |
| CTD-2010I16.1 | sCCA2 | 650 | 10 | lasso | 0.39 | 1.6e-34 | -3.38 | 0.000716 | 0.34 |
| AP001372.2 | sCCA3 | 413 | 5 | lasso | 0.12 | 4.9e-10 | 3.21 | 0.00133 | 0.34 |
| BRWD1-AS2 | sCCA1 | 675 | 57 | enet | 0.5 | 3.3e-48 | -3.42 | 0.000618 | 0.34 |
| BRWD1-AS2 | sCCA2 | 675 | 1 | top1 | 0.18 | 6.7e-15 | -3.25 | 0.00117 | 0.34 |
| HMBS | sCCA2 | 376 | 12 | lasso | 0.54 | 1.5e-53 | -3.16 | 0.001598 | 0.34 |
| SUZ12P1 | sCCA3 | 287 | 35 | enet | 0.8 | 2.3e-107 | -3.34 | 0.000835 | 0.34 |
| SUZ12P1 | sCCA2 | 287 | 37 | enet | 0.68 | 2.7e-77 | -3.33 | 0.00087 | 0.34 |
| SUZ12P1 | sCCA1 | 287 | 44 | enet | 0.81 | 5.6e-111 | -3.26 | 0.001098 | 0.34 |
| SH3GL1P2 | sCCA1 | 291 | 9 | enet | 0.05 | 4.7e-05 | 3.2 | 0.001363 | 0.34 |
| HMGN3-AS1 | sCCA2 | 430 | 18 | enet | 0.18 | 4e-15 | 3.37 | 0.000763 | 0.34 |
| CTB-75G16.1 | sCCA1 | 328 | 13 | enet | 0.061 | 7.5e-06 | -3.16 | 0.001572 | 0.34 |
| RP1-278C19.8 | sCCA2 | 375 | 1 | top1 | 0.037 | 0.00045 | -3.28 | 0.001023 | 0.34 |
| STAG3L2 | sCCA2 | 97 | 5 | lasso | 0.19 | 3.8e-16 | -3.21 | 0.001343 | 0.34 |
| RP11-93B14.9 | sCCA1 | 536 | 6 | lasso | 0.17 | 6.9e-14 | 3.28 | 0.00106 | 0.34 |
| MYO19 | sCCA1 | 334 | 29 | enet | 0.52 | 1.6e-50 | -3.35 | 0.000806 | 0.34 |
| MYO19 | sCCA3 | 334 | 30 | enet | 0.52 | 1e-50 | -3.35 | 0.000813 | 0.34 |
| GGNBP2 | sCCA3 | 334 | 12 | lasso | 0.56 | 2.8e-56 | 3.23 | 0.001216 | 0.34 |
| GGNBP2 | sCCA1 | 334 | 12 | lasso | 0.58 | 4.4e-59 | 3.19 | 0.0014 | 0.34 |
| GGNBP2 | sCCA2 | 334 | 8 | lasso | 0.21 | 2e-17 | -3.16 | 0.001553 | 0.34 |
| DHRS11 | sCCA1 | 323 | 1 | top1 | 0.34 | 1.1e-29 | 3.29 | 0.001001 | 0.34 |
| DHRS11 | sCCA2 | 323 | 1 | top1 | 0.22 | 4.2e-18 | 3.29 | 0.001001 | 0.34 |
| DHRS11 | sCCA3 | 323 | 12 | enet | 0.13 | 2.5e-11 | -3.2 | 0.001374 | 0.34 |
| RP11-640N20.4 | sCCA3 | 288 | 11 | enet | 0.022 | 0.0054 | -3.31 | 0.00094 | 0.34 |
| RP11-299G20.5 | sCCA2 | 604 | 6 | lasso | 0.085 | 1.3e-07 | -3.18 | 0.00149 | 0.34 |
| AL022393.9 | sCCA3 | 461 | 14 | enet | 0.084 | 1.6e-07 | 3.37 | 0.000749 | 0.34 |
| EP300 | sCCA2 | 270 | 1 | top1 | 0.093 | 3.5e-08 | -3.09 | 0.001977 | 0.35 |
| B3GAT2 | sCCA3 | 415 | 8 | lasso | 0.16 | 2.1e-13 | 3.09 | 0.00201 | 0.35 |
| TNFSF10 | sCCA2 | 475 | 53 | enet | 0.014 | 0.022 | -3.1 | 0.001959 | 0.35 |
| UBL3 | sCCA3 | 580 | 46 | enet | 0.0024 | 0.19 | 3.09 | 0.002 | 0.35 |
| ECSIT | sCCA1 | 384 | 1 | top1 | 0.16 | 2e-13 | 3.09 | 0.002006 | 0.35 |
| NAA35 | sCCA2 | 309 | 29 | enet | 0.02 | 0.0073 | 3.1 | 0.001944 | 0.35 |
| CDCA7L | sCCA3 | 753 | 41 | enet | 0.19 | 4.8e-16 | -3.11 | 0.001874 | 0.35 |
| RPL23P2 | sCCA1 | 426 | 34 | enet | 0.41 | 2.1e-36 | -3.1 | 0.00194 | 0.35 |
| PSMG1 | sCCA2 | 637 | 10 | lasso | 0.49 | 2e-46 | -3.09 | 0.00201 | 0.35 |
| ASB13 | sCCA3 | 670 | 31 | enet | 0.15 | 6.6e-13 | 3.11 | 0.00185 | 0.35 |
| ZNF569 | sCCA2 | 278 | 16 | enet | 0.12 | 6.9e-10 | -3.1 | 0.001926 | 0.35 |
| MGEA5 | sCCA2 | 259 | 4 | enet | 0.015 | 0.017 | 3.11 | 0.0019 | 0.35 |
| C6orf89 | sCCA3 | 540 | 6 | lasso | 0.14 | 5.7e-12 | 3.09 | 0.00198 | 0.35 |
| CTD-2010I16.1 | sCCA1 | 650 | 31 | enet | 0.5 | 3.4e-48 | -3.1 | 0.00194 | 0.35 |
| TBC1D23 | sCCA3 | 346 | 1 | top1 | 0.54 | 2e-53 | -3.06 | 0.002188 | 0.36 |
| TBC1D23 | sCCA1 | 346 | 1 | top1 | 0.55 | 7.2e-55 | 3.06 | 0.00219 | 0.36 |
| TRIP13 | sCCA3 | 372 | 9 | enet | 0.056 | 1.8e-05 | -3.06 | 0.002228 | 0.36 |
| ANKRD13A | sCCA2 | 299 | 34 | enet | 0.041 | 0.00024 | 3.08 | 0.002102 | 0.36 |
| AP4S1 | sCCA3 | 336 | 11 | enet | 0.063 | 5.2e-06 | -3.06 | 0.002218 | 0.36 |
| PRDM2 | sCCA2 | 512 | 15 | enet | 0.066 | 3e-06 | -3.07 | 0.002151 | 0.36 |
| MSTO1 | sCCA3 | 219 | 9 | enet | 0.094 | 2.8e-08 | 3.06 | 0.00223 | 0.36 |
| TIMM10B | sCCA1 | 654 | 9 | lasso | 0.29 | 5.5e-25 | 3.06 | 0.002191 | 0.36 |
| GAS2 | sCCA2 | 396 | 11 | enet | 0.17 | 1.6e-14 | 3.06 | 0.0022 | 0.36 |
| FAM227B | sCCA2 | 367 | 6 | lasso | 0.19 | 3.9e-16 | -3.08 | 0.00204 | 0.36 |
| PIP4K2C | sCCA1 | 337 | 7 | lasso | 0.11 | 2.6e-09 | 3.06 | 0.002188 | 0.36 |
| HSPA4 | sCCA1 | 408 | 42 | enet | 0.26 | 5.1e-22 | -3.06 | 0.002216 | 0.36 |
| GBA | sCCA3 | 314 | 22 | enet | 0.1 | 8.8e-09 | 3.06 | 0.00224 | 0.36 |
| YOD1 | sCCA3 | 414 | 19 | enet | 0.049 | 6.1e-05 | 3.06 | 0.00222 | 0.36 |
| TET3 | sCCA1 | 371 | 5 | lasso | 0.052 | 3.7e-05 | -3.07 | 0.00212 | 0.36 |
| ARID2 | sCCA2 | 389 | 22 | enet | 0.1 | 6.7e-09 | 3.07 | 0.002164 | 0.36 |
| ZNF525 | sCCA3 | 480 | 11 | lasso | 0.25 | 1.3e-20 | 3.06 | 0.00225 | 0.36 |
| AC010976.2 | sCCA1 | 420 | 1 | top1 | 0.053 | 2.7e-05 | 3.08 | 0.00207 | 0.36 |
| RP11-288I21.1 | sCCA1 | 444 | 1 | top1 | 0.21 | 1.8e-17 | 3.08 | 0.00204 | 0.36 |
| ARPIN | sCCA3 | 413 | 4 | lasso | 0.14 | 1.3e-11 | 3.06 | 0.00221 | 0.36 |
| PLPP1 | sCCA1 | 330 | 6 | lasso | 0.09 | 5.7e-08 | 3.01 | 0.002603 | 0.37 |
| TMEM38A | sCCA1 | 379 | 8 | lasso | 0.26 | 5.9e-22 | 3.03 | 0.002444 | 0.37 |
| DMPK | sCCA2 | 386 | 1 | top1 | 0.21 | 7.6e-18 | -3.03 | 0.002423 | 0.37 |
| ABHD11 | sCCA3 | 279 | 5 | lasso | 0.42 | 3.4e-38 | 3.03 | 0.002475 | 0.37 |
| ABHD11 | sCCA2 | 279 | 1 | top1 | 0.083 | 1.9e-07 | 3.02 | 0.002562 | 0.37 |
| SNX8 | sCCA3 | 414 | 4 | lasso | 0.12 | 7.1e-10 | -3.01 | 0.002584 | 0.37 |
| MEGF9 | sCCA1 | 341 | 1 | top1 | 0.45 | 1.5e-41 | 3.02 | 0.00256 | 0.37 |
| OAS2 | sCCA1 | 422 | 10 | lasso | 0.22 | 5.3e-18 | -3.01 | 0.002622 | 0.37 |
| ESYT2 | sCCA3 | 407 | 13 | lasso | 0.21 | 1.4e-17 | 3 | 0.002657 | 0.37 |
| ARHGAP9 | sCCA2 | 334 | 22 | enet | 0.074 | 7.9e-07 | -3 | 0.002657 | 0.37 |
| AGO2 | sCCA1 | 369 | 23 | enet | 0.12 | 1.5e-10 | 3.03 | 0.00242 | 0.37 |
| TST | sCCA3 | 698 | 27 | enet | 0.032 | 0.001 | 3.03 | 0.00245 | 0.37 |
| TTBK2 | sCCA1 | 282 | 1 | top1 | 0.21 | 4.2e-17 | -3 | 0.00267 | 0.37 |
| POMP | sCCA1 | 470 | 1 | top1 | 0.015 | 0.018 | -3.01 | 0.00264 | 0.37 |
| RRAS2 | sCCA1 | 376 | 41 | enet | 0.39 | 8.3e-35 | 3.03 | 0.00245 | 0.37 |
| RRAS2 | sCCA2 | 376 | 39 | enet | 0.39 | 1.4e-34 | -3 | 0.002701 | 0.37 |
| WASHC4 | sCCA1 | 647 | 11 | lasso | 0.52 | 2.7e-50 | -3.03 | 0.002485 | 0.37 |
| RIT1 | sCCA2 | 281 | 1 | top1 | 0.23 | 2.2e-19 | -3.01 | 0.002653 | 0.37 |
| TMEM168 | sCCA1 | 359 | 13 | enet | 0.066 | 3.4e-06 | -3 | 0.00272 | 0.37 |
| CYP17A1 | sCCA1 | 345 | 22 | enet | 0.083 | 1.9e-07 | -3.02 | 0.00251 | 0.37 |
| DPY19L4 | sCCA1 | 444 | 5 | lasso | 0.23 | 2.7e-19 | -3.02 | 0.00255 | 0.37 |
| UBR1 | sCCA3 | 299 | 1 | top1 | 0.016 | 0.015 | 3 | 0.00267 | 0.37 |
| AP2M1 | sCCA1 | 488 | 6 | lasso | 0.2 | 5.7e-17 | 3.03 | 0.00242 | 0.37 |
| 8-Mar | sCCA2 | 263 | 6 | lasso | 0.29 | 1.1e-24 | -3.04 | 0.00236 | 0.37 |
| 8-Mar | sCCA3 | 263 | 51 | enet | 0.36 | 1.2e-31 | -3.01 | 0.00262 | 0.37 |
| PHB | sCCA2 | 350 | 26 | enet | 0.026 | 0.0028 | -3.01 | 0.002633 | 0.37 |
| PSMG1 | sCCA3 | 637 | 9 | lasso | 0.74 | 5.6e-91 | 3.04 | 0.002361 | 0.37 |
| CARD9 | sCCA2 | 370 | 8 | lasso | 0.17 | 2.3e-14 | -3.03 | 0.00245 | 0.37 |
| ZNF569 | sCCA3 | 278 | 30 | enet | 0.098 | 1.4e-08 | -3.01 | 0.00258 | 0.37 |
| C6orf89 | sCCA2 | 540 | 25 | enet | 0.14 | 6.1e-12 | 3.03 | 0.00246 | 0.37 |
| PDCD4-AS1 | sCCA2 | 519 | 12 | enet | 0.003 | 0.17 | -3.01 | 0.00263 | 0.37 |
| HCP5 | sCCA2 | 272 | 46 | enet | 0.086 | 1e-07 | -3 | 0.00271 | 0.37 |
| VAMP2 | sCCA1 | 439 | 1 | top1 | 0.19 | 4.4e-16 | 3.03 | 0.002471 | 0.37 |
| RP11-263K19.4 | sCCA1 | 336 | 30 | enet | 0.12 | 3.3e-10 | -3.02 | 0.00256 | 0.37 |
| RP3-355L5.4 | sCCA1 | 395 | 29 | enet | 0.17 | 2.2e-14 | -3.01 | 0.0026 | 0.37 |
| RP11-263K19.6 | sCCA3 | 332 | 16 | enet | 0.055 | 2e-05 | 3.05 | 0.00229 | 0.37 |
| ZNF585B | sCCA2 | 277 | 43 | enet | 0.35 | 4.7e-30 | -3.02 | 0.002496 | 0.37 |
| RP11-29H23.5 | sCCA2 | 222 | 1 | top1 | 0.014 | 0.023 | -3.01 | 0.002653 | 0.37 |
| CLDN23 | sCCA2 | 628 | 6 | lasso | 0.45 | 8.9e-42 | -3.01 | 0.002653 | 0.37 |
| CTD-2010I16.1 | sCCA3 | 650 | 34 | enet | 0.51 | 9.6e-49 | -3.04 | 0.00235 | 0.37 |
| AC007191.4 | sCCA1 | 369 | 1 | top1 | 0.059 | 9.8e-06 | 3.03 | 0.002423 | 0.37 |
| ACAA1 | sCCA1 | 425 | 50 | enet | 0.17 | 1.6e-14 | 2.97 | 0.00293 | 0.38 |
| NSF | sCCA3 | 146 | 9 | enet | 0.067 | 2.8e-06 | -2.98 | 0.002837 | 0.38 |
| ITPKC | sCCA2 | 429 | 17 | enet | 0.35 | 1.2e-30 | -2.97 | 0.003026 | 0.38 |
| ZNF821 | sCCA1 | 401 | 22 | enet | -0.00099 | 0.4 | -2.98 | 0.00288 | 0.38 |
| SH3PXD2A | sCCA2 | 423 | 1 | top1 | 0.016 | 0.016 | -2.97 | 0.00297 | 0.38 |
| COQ8B | sCCA3 | 429 | 11 | lasso | 0.18 | 9.2e-15 | -2.97 | 0.00293 | 0.38 |
| ECSIT | sCCA2 | 384 | 1 | top1 | 0.11 | 4.1e-09 | 2.97 | 0.002959 | 0.38 |
| PPIL4 | sCCA3 | 425 | 1 | top1 | 0.048 | 6.6e-05 | -2.98 | 0.00287 | 0.38 |
| SELENOS | sCCA3 | 614 | 26 | enet | 0.018 | 0.01 | 2.96 | 0.00309 | 0.38 |
| PCED1A | sCCA2 | 527 | 17 | enet | 0.019 | 0.0093 | 2.96 | 0.00303 | 0.38 |
| VAV3 | sCCA3 | 465 | 6 | lasso | 0.044 | 0.00013 | 2.96 | 0.00311 | 0.38 |
| TMEM243 | sCCA2 | 452 | 4 | lasso | 0.034 | 0.00071 | 2.99 | 0.002782 | 0.38 |
| TUBGCP4 | sCCA3 | 269 | 43 | enet | 0.081 | 2.5e-07 | 2.95 | 0.00315 | 0.38 |
| CDAN1 | sCCA2 | 278 | 24 | enet | 0.07 | 1.7e-06 | -2.96 | 0.00308 | 0.38 |
| RIT1 | sCCA1 | 281 | 8 | lasso | 0.56 | 1.3e-56 | 2.98 | 0.00292 | 0.38 |
| SFXN2 | sCCA3 | 365 | 1 | top1 | 0.14 | 1.6e-11 | -2.95 | 0.00314 | 0.38 |
| KAT6B | sCCA1 | 275 | 26 | enet | 0.082 | 2.3e-07 | 2.98 | 0.00287 | 0.38 |
| ZNF222 | sCCA2 | 499 | 45 | enet | 0.016 | 0.015 | 2.95 | 0.003129 | 0.38 |
| FBXO41 | sCCA3 | 292 | 10 | enet | 0.1 | 8.5e-09 | -2.96 | 0.00304 | 0.38 |
| TBC1D2B | sCCA3 | 415 | 20 | enet | 0.35 | 1.1e-30 | -2.97 | 0.00301 | 0.38 |
| TMUB2 | sCCA3 | 351 | 9 | enet | 0.082 | 2.2e-07 | 2.98 | 0.002879 | 0.38 |
| NMNAT1 | sCCA2 | 298 | 14 | lasso | 0.073 | 9.3e-07 | -2.96 | 0.003029 | 0.38 |
| UCP3 | sCCA1 | 378 | 23 | enet | 0.03 | 0.0014 | 2.99 | 0.00283 | 0.38 |
| AKAP5 | sCCA3 | 497 | 1 | top1 | 0.039 | 0.00031 | -2.96 | 0.00307 | 0.38 |
| RWDD4 | sCCA3 | 443 | 1 | top1 | 0.07 | 1.7e-06 | -2.96 | 0.00304 | 0.38 |
| PSMG1 | sCCA1 | 637 | 9 | lasso | 0.75 | 1.3e-92 | 2.97 | 0.002993 | 0.38 |
| LDB1 | sCCA3 | 272 | 1 | top1 | 0.12 | 3.2e-10 | 2.99 | 0.00281 | 0.38 |
| AC009961.3 | sCCA3 | 469 | 38 | enet | 0.22 | 2.6e-18 | 2.98 | 0.00293 | 0.38 |
| CLDN23 | sCCA1 | 628 | 1 | top1 | 0.53 | 8.2e-52 | 2.96 | 0.00311 | 0.38 |
| CLDN23 | sCCA3 | 628 | 1 | top1 | 0.62 | 1.5e-66 | 2.96 | 0.00311 | 0.38 |
| RP11-539G18.3 | sCCA1 | 351 | 5 | lasso | 0.14 | 4.1e-12 | -2.99 | 0.00277 | 0.38 |
| GTF2I | sCCA1 | 155 | 12 | lasso | 0.72 | 5.5e-86 | -2.99 | 0.0028 | 0.38 |
| XXbac-BPG299F13.17 | sCCA1 | 173 | 105 | enet | 0.73 | 6.8e-88 | -2.97 | 0.00301 | 0.38 |
| XXbac-BPG299F13.17 | sCCA2 | 173 | 27 | enet | 0.68 | 9.1e-78 | -2.96 | 0.00309 | 0.38 |
| IKBKAP | sCCA1 | 609 | 29 | enet | 0.38 | 2.4e-33 | 2.93 | 0.00339 | 0.39 |
| MLH1 | sCCA1 | 407 | 1 | top1 | 0.11 | 2e-09 | -2.94 | 0.00331 | 0.39 |
| CRYM | sCCA2 | 251 | 1 | top1 | 0.064 | 4.9e-06 | -2.92 | 0.00345 | 0.39 |
| ZFP30 | sCCA2 | 292 | 8 | lasso | 0.26 | 5.8e-22 | -2.93 | 0.003383 | 0.39 |
| NCAPH | sCCA2 | 162 | 20 | enet | 0.038 | 0.00039 | -2.93 | 0.00342 | 0.39 |
| COQ8B | sCCA2 | 429 | 1 | top1 | 0.18 | 4.6e-15 | -2.93 | 0.003442 | 0.39 |
| FOSB | sCCA1 | 372 | 1 | top1 | 0.016 | 0.017 | 2.93 | 0.003339 | 0.39 |
| ADRM1 | sCCA3 | 506 | 6 | lasso | -0.0015 | 0.46 | -2.94 | 0.00323 | 0.39 |
| ANKHD1 | sCCA3 | 311 | 9 | enet | -0.00089 | 0.39 | 2.94 | 0.003249 | 0.39 |
| ZBED3 | sCCA2 | 587 | 31 | enet | 0.1 | 4.6e-09 | 2.92 | 0.003467 | 0.39 |
| YME1L1 | sCCA2 | 454 | 23 | enet | 0.067 | 2.9e-06 | -2.93 | 0.00339 | 0.39 |
| MAGOH | sCCA3 | 498 | 6 | lasso | 0.054 | 2.4e-05 | -2.93 | 0.00334 | 0.39 |
| TSACC | sCCA1 | 406 | 5 | lasso | -0.00055 | 0.36 | 2.94 | 0.00325 | 0.39 |
| AMOTL1 | sCCA1 | 480 | 1 | top1 | 0.066 | 3.1e-06 | -2.95 | 0.003206 | 0.39 |
| PBX3 | sCCA3 | 382 | 18 | enet | 0.03 | 0.0013 | 2.93 | 0.00339 | 0.39 |
| DTX3 | sCCA1 | 332 | 7 | lasso | 0.044 | 0.00013 | 2.93 | 0.003431 | 0.39 |
| LMNTD2 | sCCA3 | 435 | 6 | lasso | 0.0052 | 0.11 | -2.92 | 0.00347 | 0.39 |
| QRFP | sCCA2 | 472 | 3 | lasso | 0.061 | 7e-06 | 2.93 | 0.003367 | 0.39 |
| WDR5B | sCCA3 | 460 | 59 | enet | 0.65 | 3.7e-71 | 2.95 | 0.003187 | 0.39 |
| RP1-257A7.4 | sCCA3 | 523 | 40 | enet | 0.28 | 8.5e-24 | -2.94 | 0.00329 | 0.39 |
| ZNF192P1 | sCCA1 | 464 | 13 | lasso | 0.31 | 4.7e-26 | 2.94 | 0.00331 | 0.39 |
| RP1-151F17.1 | sCCA1 | 550 | 50 | enet | 0.046 | 1e-04 | 2.95 | 0.0032 | 0.39 |
| RP11-29H23.5 | sCCA1 | 222 | 11 | enet | 0.029 | 0.0016 | 2.92 | 0.00346 | 0.39 |
| EIF3D | sCCA2 | 652 | 1 | top1 | 0.12 | 2.1e-10 | 2.91 | 0.003604 | 0.4 |
| ASH1L | sCCA3 | 223 | 1 | top1 | 0.037 | 0.00043 | -2.91 | 0.00357 | 0.4 |
| RIT1 | sCCA3 | 281 | 5 | lasso | 0.42 | 2.4e-38 | -2.91 | 0.00358 | 0.4 |
| DPCD | sCCA2 | 307 | 25 | enet | 0.076 | 6.5e-07 | -2.92 | 0.00354 | 0.4 |
| WDR5B | sCCA1 | 460 | 54 | enet | 0.65 | 1.6e-71 | 2.91 | 0.00363 | 0.4 |
| LTN1 | sCCA3 | 426 | 21 | enet | 0.12 | 3.4e-10 | 2.91 | 0.003611 | 0.4 |
| RP11-571M6.17 | sCCA1 | 350 | 25 | enet | 0.16 | 3.5e-13 | -2.91 | 0.003559 | 0.4 |
| ABCB1 | sCCA1 | 403 | 44 | enet | 0.046 | 9.2e-05 | -2.88 | 0.00398 | 0.41 |
| SNAP23 | sCCA2 | 366 | 1 | top1 | 0.13 | 4.1e-11 | 2.9 | 0.00373 | 0.41 |
| ACIN1 | sCCA1 | 484 | 7 | lasso | 0.019 | 0.0094 | 2.9 | 0.00369 | 0.41 |
| LAPTM4B | sCCA1 | 510 | 39 | enet | 0.38 | 2.1e-33 | -2.88 | 0.00398 | 0.41 |
| STN1 | sCCA1 | 428 | 24 | enet | 0.14 | 1.7e-11 | -2.91 | 0.00366 | 0.41 |
| WNT3 | sCCA2 | 255 | 27 | enet | 0.29 | 1.1e-24 | -2.9 | 0.003769 | 0.41 |
| MLEC | sCCA2 | 442 | 10 | lasso | 0.18 | 6.1e-15 | 2.9 | 0.003682 | 0.41 |
| SRSF9 | sCCA1 | 395 | 4 | lasso | 0.082 | 2.2e-07 | 2.89 | 0.003822 | 0.41 |
| CHMP3 | sCCA1 | 334 | 42 | enet | 0.58 | 6.3e-60 | 2.89 | 0.00388 | 0.41 |
| PLEKHA3 | sCCA1 | 464 | 22 | enet | 0.18 | 2.9e-15 | 2.88 | 0.00399 | 0.41 |
| ATP6V1F | sCCA2 | 337 | 28 | enet | 0.0077 | 0.068 | -2.89 | 0.003899 | 0.41 |
| THAP8 | sCCA1 | 338 | 1 | top1 | 0.33 | 3.7e-28 | -2.88 | 0.003919 | 0.41 |
| THAP8 | sCCA3 | 338 | 1 | top1 | 0.3 | 7.6e-26 | 2.88 | 0.00392 | 0.41 |
| YY1AP1 | sCCA2 | 220 | 1 | top1 | 0.029 | 0.0015 | -2.89 | 0.003875 | 0.41 |
| 8-Mar | sCCA1 | 263 | 50 | enet | 0.35 | 4.9e-30 | -2.89 | 0.00388 | 0.41 |
| TBC1D2B | sCCA1 | 415 | 36 | enet | 0.37 | 1.3e-32 | 2.9 | 0.00375 | 0.41 |
| RNF26 | sCCA3 | 412 | 16 | enet | 0.047 | 7.5e-05 | -2.89 | 0.00389 | 0.41 |
| TMEM107 | sCCA1 | 432 | 5 | lasso | 0.071 | 1.3e-06 | -2.89 | 0.003883 | 0.41 |
| ZNF607 | sCCA2 | 298 | 7 | lasso | 0.15 | 2.9e-12 | -2.89 | 0.003794 | 0.41 |
| CCDC7 | sCCA1 | 317 | 69 | enet | 0.54 | 5.4e-53 | -2.89 | 0.0038 | 0.41 |
| RP11-541N10.3 | sCCA1 | 420 | 4 | lasso | 0.093 | 3.2e-08 | 2.9 | 0.00378 | 0.41 |
| MYL12BP1 | sCCA1 | 324 | 6 | lasso | 0.16 | 1e-13 | -2.9 | 0.00378 | 0.41 |
| RP11-669E14.6 | sCCA3 | 29 | 4 | lasso | 0.11 | 2.3e-09 | 2.88 | 0.003989 | 0.41 |
| MYO19 | sCCA2 | 334 | 1 | top1 | 0.42 | 2.6e-38 | -2.88 | 0.003931 | 0.41 |
| RAB18 | sCCA3 | 441 | 33 | enet | 0.078 | 4.4e-07 | 2.87 | 0.00412 | 0.42 |
| ZNF106 | sCCA3 | 366 | 1 | top1 | 0.026 | 0.0028 | 2.87 | 0.00413 | 0.42 |
| KANSL3 | sCCA3 | 203 | 1 | top1 | 0.071 | 1.4e-06 | -2.87 | 0.00407 | 0.42 |
| CHMP3 | sCCA3 | 334 | 41 | enet | 0.59 | 1.9e-60 | 2.87 | 0.00408 | 0.42 |
| RTN2 | sCCA3 | 372 | 6 | lasso | 0.022 | 0.0056 | 2.87 | 0.00413 | 0.42 |
| ENTPD1 | sCCA2 | 510 | 5 | lasso | 0.16 | 2.8e-13 | -2.87 | 0.00406 | 0.42 |
| UBR1 | sCCA2 | 299 | 1 | top1 | 0.065 | 3.8e-06 | 2.87 | 0.00414 | 0.42 |
| GEM | sCCA3 | 454 | 1 | top1 | 0.064 | 4.5e-06 | 2.88 | 0.00404 | 0.42 |
| CARD9 | sCCA3 | 370 | 31 | enet | 0.12 | 6.9e-10 | 2.87 | 0.00409 | 0.42 |
| RPS3AP47 | sCCA1 | 296 | 1 | top1 | 0.046 | 9.8e-05 | -2.87 | 0.00414 | 0.42 |
| TMEM62 | sCCA1 | 297 | 1 | top1 | 0.086 | 1e-07 | 2.85 | 0.00432 | 0.43 |
| CLK2 | sCCA2 | 295 | 1 | top1 | -0.002 | 0.53 | -2.86 | 0.004226 | 0.43 |
| NTAN1P2 | sCCA3 | 445 | 7 | lasso | 0.085 | 1.4e-07 | 2.86 | 0.00427 | 0.43 |
| SKIV2L2 | sCCA1 | 386 | 7 | lasso | 0.18 | 4.5e-15 | 2.83 | 0.004603 | 0.44 |
| TELO2 | sCCA3 | 491 | 33 | enet | 0.52 | 7e-51 | -2.85 | 0.00443 | 0.44 |
| SLC38A7 | sCCA3 | 472 | 7 | lasso | 0.057 | 1.5e-05 | 2.83 | 0.00461 | 0.44 |
| C5 | sCCA3 | 374 | 1 | top1 | 0.14 | 5.8e-12 | 2.84 | 0.00453 | 0.44 |
| ENPP5 | sCCA2 | 514 | 5 | lasso | 0.12 | 1.7e-10 | 2.84 | 0.004518 | 0.44 |
| CHST10 | sCCA3 | 485 | 3 | lasso | 0.082 | 2.2e-07 | -2.85 | 0.00443 | 0.44 |
| ESYT2 | sCCA2 | 407 | 25 | enet | 0.24 | 1.1e-19 | 2.85 | 0.004371 | 0.44 |
| PGAP2 | sCCA1 | 355 | 6 | lasso | 0.11 | 1e-09 | 2.85 | 0.00442 | 0.44 |
| PIP4K2C | sCCA3 | 337 | 21 | enet | 0.1 | 6.3e-09 | -2.85 | 0.00439 | 0.44 |
| ANAPC7 | sCCA2 | 226 | 23 | enet | 0.075 | 6.9e-07 | 2.84 | 0.00455 | 0.44 |
| DDI2 | sCCA1 | 478 | 8 | lasso | 0.24 | 5.1e-20 | -2.84 | 0.00445 | 0.44 |
| TAS2R15P | sCCA3 | 381 | 19 | enet | 0.22 | 5.3e-18 | 2.84 | 0.00456 | 0.44 |
| HMBS | sCCA3 | 376 | 48 | enet | 0.59 | 3.9e-60 | 2.85 | 0.00442 | 0.44 |
| ZNF790-AS1 | sCCA3 | 276 | 6 | lasso | 0.0099 | 0.045 | -2.84 | 0.00448 | 0.44 |
| RP11-1099M24.6 | sCCA1 | 507 | 1 | top1 | 0.4 | 3.6e-36 | -2.84 | 0.004532 | 0.44 |
| BRD9 | sCCA1 | 372 | 1 | top1 | 0.32 | 9.4e-28 | -2.75 | 0.006008 | 0.45 |
| BRD9 | sCCA2 | 372 | 1 | top1 | 0.11 | 1.2e-09 | 2.75 | 0.006008 | 0.45 |
| HOMER3 | sCCA1 | 332 | 12 | enet | 0.61 | 2.2e-64 | -2.78 | 0.005441 | 0.45 |
| FOXC1 | sCCA2 | 662 | 6 | lasso | 0.1 | 6.5e-09 | 2.76 | 0.005711 | 0.45 |
| SCARF1 | sCCA3 | 429 | 1 | top1 | 0.036 | 0.00053 | -2.78 | 0.005434 | 0.45 |
| ZNF416 | sCCA1 | 478 | 1 | top1 | 0.0049 | 0.12 | -2.83 | 0.004727 | 0.45 |
| CHMP5 | sCCA3 | 461 | 45 | enet | 0.0089 | 0.054 | -2.75 | 0.00605 | 0.45 |
| PSME2 | sCCA3 | 509 | 1 | top1 | 0.027 | 0.0022 | 2.83 | 0.004658 | 0.45 |
| ADNP | sCCA1 | 476 | 5 | lasso | 0.051 | 4.4e-05 | -2.77 | 0.00568 | 0.45 |
| ACBD5 | sCCA2 | 457 | 12 | enet | 0.097 | 1.7e-08 | 2.77 | 0.0056 | 0.45 |
| SLC38A1 | sCCA2 | 312 | 24 | enet | 0.075 | 7.4e-07 | -2.83 | 0.004663 | 0.45 |
| B3GAT2 | sCCA1 | 415 | 7 | lasso | 0.5 | 7.6e-48 | 2.74 | 0.00605 | 0.45 |
| PADI2 | sCCA2 | 384 | 1 | top1 | 0.056 | 1.7e-05 | -2.77 | 0.005547 | 0.45 |
| PRRC2C | sCCA2 | 591 | 34 | enet | 0.099 | 1.1e-08 | 2.81 | 0.004942 | 0.45 |
| CTNNAL1 | sCCA1 | 622 | 6 | lasso | 0.61 | 1e-64 | -2.8 | 0.00505 | 0.45 |
| WBP4 | sCCA2 | 305 | 22 | enet | 0.083 | 1.9e-07 | 2.8 | 0.00519 | 0.45 |
| ZFP30 | sCCA3 | 292 | 1 | top1 | 0.13 | 9.9e-11 | 2.76 | 0.0058 | 0.45 |
| GLT8D2 | sCCA2 | 704 | 20 | enet | 0.0037 | 0.14 | -2.79 | 0.005263 | 0.45 |
| HERPUD2 | sCCA2 | 542 | 53 | enet | 0.089 | 6.9e-08 | 2.79 | 0.005292 | 0.45 |
| IFT81 | sCCA2 | 261 | 19 | enet | 0.072 | 1.2e-06 | -2.75 | 0.005878 | 0.45 |
| WWP1 | sCCA1 | 403 | 12 | lasso | 0.32 | 5e-27 | -2.8 | 0.00511 | 0.45 |
| G0S2 | sCCA2 | 496 | 22 | enet | 0.038 | 0.00034 | -2.76 | 0.005726 | 0.45 |
| PMS2P5 | sCCA3 | 97 | 3 | lasso | 0.2 | 8.9e-17 | 2.75 | 0.005935 | 0.45 |
| CAPNS1 | sCCA3 | 332 | 26 | enet | 0.0095 | 0.049 | -2.83 | 0.00468 | 0.45 |
| BECN1 | sCCA1 | 233 | 36 | enet | 0.22 | 5.7e-18 | -2.8 | 0.005154 | 0.45 |
| CALU | sCCA1 | 352 | 9 | lasso | 0.33 | 4.2e-28 | 2.79 | 0.00533 | 0.45 |
| KIAA1468 | sCCA2 | 508 | 15 | lasso | 0.17 | 7.4e-14 | 2.82 | 0.00487 | 0.45 |
| KIAA1468 | sCCA3 | 508 | 12 | lasso | 0.37 | 5.2e-32 | 2.78 | 0.00538 | 0.45 |
| GTF2B | sCCA1 | 353 | 24 | enet | 0.1 | 7.3e-09 | -2.76 | 0.00576 | 0.45 |
| ACTR1A | sCCA1 | 333 | 1 | top1 | 0.11 | 1.6e-09 | -2.74 | 0.00609 | 0.45 |
| WDR12 | sCCA1 | 260 | 4 | lasso | 0.029 | 0.0017 | 2.82 | 0.0048 | 0.45 |
| PPA2 | sCCA2 | 403 | 84 | enet | 0.35 | 2e-30 | 2.8 | 0.00512 | 0.45 |
| RNF185 | sCCA3 | 364 | 26 | enet | 0.2 | 2.9e-16 | -2.78 | 0.00541 | 0.45 |
| IMPA2 | sCCA2 | 449 | 21 | enet | 0.24 | 2.6e-20 | -2.82 | 0.00482 | 0.45 |
| EFHD2 | sCCA2 | 519 | 7 | lasso | 0.085 | 1.4e-07 | 2.77 | 0.00564 | 0.45 |
| RPL11 | sCCA2 | 339 | 11 | enet | 0.029 | 0.0017 | -2.76 | 0.005722 | 0.45 |
| MOB3C | sCCA2 | 362 | 56 | enet | 0.14 | 4e-12 | 2.8 | 0.005125 | 0.45 |
| ADAM15 | sCCA1 | 377 | 33 | enet | 0.25 | 1.5e-20 | 2.81 | 0.00489 | 0.45 |
| EFNA3 | sCCA1 | 371 | 36 | enet | 0.074 | 8.3e-07 | 2.76 | 0.00581 | 0.45 |
| HCN3 | sCCA1 | 296 | 1 | top1 | 0.29 | 5.3e-25 | 2.8 | 0.00516 | 0.45 |
| HCN3 | sCCA3 | 296 | 1 | top1 | -0.0024 | 0.61 | 2.78 | 0.00548 | 0.45 |
| HCN3 | sCCA2 | 296 | 1 | top1 | 0.042 | 0.00018 | 2.78 | 0.005492 | 0.45 |
| CENPC | sCCA1 | 407 | 9 | lasso | 0.46 | 4.6e-43 | -2.81 | 0.00489 | 0.45 |
| IRAK1BP1 | sCCA2 | 375 | 1 | top1 | 0.49 | 6.9e-46 | -2.75 | 0.005972 | 0.45 |
| PHIP | sCCA1 | 410 | 1 | top1 | 0.24 | 4.2e-20 | -2.82 | 0.00476 | 0.45 |
| PHIP | sCCA3 | 410 | 1 | top1 | 0.11 | 2.7e-09 | 2.82 | 0.00476 | 0.45 |
| ANKRD1 | sCCA1 | 440 | 10 | lasso | 0.13 | 3.7e-11 | 2.78 | 0.00543 | 0.45 |
| FAM151B | sCCA2 | 439 | 9 | enet | 0.011 | 0.035 | 2.78 | 0.00551 | 0.45 |
| ABCA10 | sCCA1 | 422 | 44 | enet | 0.2 | 8.2e-17 | 2.74 | 0.00608 | 0.45 |
| EEF1A1 | sCCA2 | 390 | 1 | top1 | 0.062 | 5.9e-06 | -2.8 | 0.005127 | 0.45 |
| SASS6 | sCCA1 | 450 | 28 | enet | 0.3 | 3.4e-25 | 2.81 | 0.00502 | 0.45 |
| ZC3H18 | sCCA1 | 373 | 16 | enet | 0.15 | 2e-12 | -2.74 | 0.00612 | 0.45 |
| EFCAB14 | sCCA3 | 376 | 14 | enet | 0.21 | 2.2e-17 | 2.78 | 0.00549 | 0.45 |
| VPS11 | sCCA1 | 380 | 19 | enet | 0.59 | 3.3e-61 | 2.75 | 0.005882 | 0.45 |
| FDPS | sCCA1 | 272 | 1 | top1 | 0.19 | 9.5e-16 | -2.8 | 0.00516 | 0.45 |
| KIAA1841 | sCCA2 | 322 | 29 | enet | 0.03 | 0.0015 | 2.81 | 0.00495 | 0.45 |
| YY1AP1 | sCCA1 | 220 | 1 | top1 | 0.074 | 8.8e-07 | 2.78 | 0.00549 | 0.45 |
| MB21D1 | sCCA3 | 377 | 1 | top1 | 0.043 | 0.00015 | 2.8 | 0.00513 | 0.45 |
| PPP1R36 | sCCA1 | 495 | 14 | lasso | 0.052 | 3.2e-05 | -2.77 | 0.00557 | 0.45 |
| XRRA1 | sCCA3 | 456 | 5 | lasso | 0.86 | 2.4e-130 | 2.74 | 0.00614 | 0.45 |
| POP5 | sCCA2 | 410 | 3 | lasso | 0.22 | 1.9e-18 | 2.76 | 0.005764 | 0.45 |
| GATAD2A | sCCA3 | 342 | 5 | lasso | 0.051 | 4e-05 | 2.76 | 0.00575 | 0.45 |
| PROSER3 | sCCA2 | 426 | 9 | lasso | 0.34 | 2.1e-29 | -2.79 | 0.005347 | 0.45 |
| PROSER3 | sCCA1 | 426 | 9 | lasso | 0.5 | 1.4e-47 | 2.78 | 0.005441 | 0.45 |
| FILIP1L | sCCA1 | 364 | 28 | enet | 0.2 | 1.2e-16 | 2.77 | 0.00552 | 0.45 |
| ATG4B | sCCA1 | 277 | 22 | enet | 0.35 | 3.2e-30 | -2.76 | 0.00574 | 0.45 |
| ATG4B | sCCA3 | 277 | 12 | enet | 0.33 | 1.4e-28 | 2.75 | 0.00595 | 0.45 |
| FAXDC2 | sCCA3 | 446 | 32 | enet | 0.082 | 2.2e-07 | -2.76 | 0.005762 | 0.45 |
| GPRC5C | sCCA3 | 505 | 33 | enet | 0.043 | 0.00015 | -2.81 | 0.004884 | 0.45 |
| SLFN11 | sCCA1 | 369 | 9 | lasso | 0.27 | 1.5e-22 | -2.76 | 0.005836 | 0.45 |
| HECTD4 | sCCA3 | 276 | 21 | enet | 0.031 | 0.0011 | 2.82 | 0.00488 | 0.45 |
| CD34 | sCCA1 | 520 | 41 | enet | 0.058 | 1.2e-05 | -2.82 | 0.00482 | 0.45 |
| THAP4 | sCCA1 | 277 | 1 | top1 | 0.17 | 6.9e-14 | -2.81 | 0.0049 | 0.45 |
| GBA | sCCA1 | 314 | 23 | enet | 0.45 | 2.2e-41 | 2.8 | 0.0051 | 0.45 |
| LDLRAD3 | sCCA2 | 618 | 3 | lasso | 0.016 | 0.015 | 2.77 | 0.005675 | 0.45 |
| PACS2 | sCCA2 | 193 | 1 | top1 | 0.047 | 8.2e-05 | -2.81 | 0.00491 | 0.45 |
| ZNF571 | sCCA2 | 290 | 12 | enet | 0.11 | 1.3e-09 | -2.82 | 0.004823 | 0.45 |
| CMTR2 | sCCA2 | 272 | 1 | top1 | 0.18 | 7.4e-15 | 2.79 | 0.0052 | 0.45 |
| RNF135 | sCCA2 | 285 | 6 | lasso | 0.064 | 4.5e-06 | -2.75 | 0.005938 | 0.45 |
| MTA1 | sCCA1 | 167 | 1 | top1 | 0.072 | 1.2e-06 | 2.74 | 0.00609 | 0.45 |
| VPS33B | sCCA3 | 496 | 9 | lasso | 0.21 | 3.4e-17 | 2.82 | 0.00475 | 0.45 |
| BRF1 | sCCA3 | 193 | 1 | top1 | 0.15 | 1.4e-12 | -2.74 | 0.006093 | 0.45 |
| ZNF749 | sCCA3 | 489 | 1 | top1 | 0.69 | 2.5e-80 | -2.77 | 0.00555 | 0.45 |
| ZNF749 | sCCA1 | 489 | 8 | lasso | 0.81 | 8.5e-113 | 2.77 | 0.00562 | 0.45 |
| ZSCAN30 | sCCA2 | 406 | 30 | enet | 0.21 | 2.8e-17 | 2.77 | 0.00561 | 0.45 |
| WDR86 | sCCA3 | 523 | 50 | enet | 0.35 | 4.3e-30 | -2.74 | 0.006081 | 0.45 |
| PRR13P5 | sCCA1 | 400 | 21 | enet | 0.013 | 0.024 | 2.81 | 0.004927 | 0.45 |
| GTF2IRD2 | sCCA2 | 103 | 18 | enet | 0.67 | 3.5e-74 | -2.79 | 0.005214 | 0.45 |
| MYL4 | sCCA1 | 337 | 3 | lasso | 0.31 | 1.7e-26 | -2.81 | 0.004971 | 0.45 |
| ZNF525 | sCCA1 | 480 | 15 | lasso | 0.3 | 2.7e-25 | 2.76 | 0.005782 | 0.45 |
| STARD7-AS1 | sCCA2 | 152 | 18 | enet | 0.11 | 8e-10 | 2.78 | 0.0055 | 0.45 |
| HMGN1 | sCCA3 | 668 | 1 | top1 | 0.01 | 0.042 | -2.78 | 0.005444 | 0.45 |
| IGHG2 | sCCA2 | 91 | 1 | top1 | 0.034 | 0.00068 | -2.81 | 0.00491 | 0.45 |
| IGHG1 | sCCA1 | 59 | 1 | top1 | 0.074 | 8.6e-07 | 2.74 | 0.00609 | 0.45 |
| RP11-27I1.4 | sCCA2 | 348 | 15 | enet | 0.042 | 0.00018 | -2.8 | 0.005118 | 0.45 |
| CCDC7 | sCCA3 | 317 | 17 | lasso | 0.36 | 5.7e-31 | -2.78 | 0.00543 | 0.45 |
| RP4-758J18.2 | sCCA1 | 277 | 26 | enet | 0.14 | 9.2e-12 | -2.77 | 0.00559 | 0.45 |
| ARL17B | sCCA1 | 60 | 17 | enet | 0.28 | 7.7e-24 | 2.75 | 0.006003 | 0.45 |
| AC079250.1 | sCCA3 | 360 | 3 | lasso | 0.018 | 0.011 | 2.77 | 0.00554 | 0.45 |
| DLEU2 | sCCA2 | 387 | 19 | enet | 0.06 | 9.2e-06 | 2.82 | 0.00477 | 0.45 |
| C12orf75 | sCCA1 | 548 | 13 | enet | 0.53 | 4.9e-52 | -2.78 | 0.005392 | 0.45 |
| TTN-AS1 | sCCA1 | 457 | 1 | top1 | 0.044 | 0.00013 | -2.76 | 0.00584 | 0.45 |
| RP11-429G19.3 | sCCA2 | 509 | 28 | enet | 0.092 | 4.1e-08 | 2.79 | 0.00532 | 0.45 |
| UBQLN4P1 | sCCA3 | 541 | 1 | top1 | 0.024 | 0.0039 | -2.78 | 0.005416 | 0.45 |
| ARPIN | sCCA1 | 413 | 1 | top1 | 0.51 | 1.3e-48 | -2.79 | 0.0052 | 0.45 |
| EFNA4 | sCCA1 | 373 | 11 | lasso | 0.11 | 2e-09 | 2.75 | 0.00595 | 0.45 |
| RP1-179N16.6 | sCCA1 | 409 | 1 | top1 | 0.71 | 1e-83 | -2.74 | 0.0061 | 0.45 |
| CH17-260O16.1 | sCCA1 | 323 | 30 | enet | 0.035 | 0.00063 | 2.79 | 0.00527 | 0.45 |
| RP11-110I1.13 | sCCA1 | 380 | 1 | top1 | 0.093 | 3.4e-08 | 2.75 | 0.00589 | 0.45 |
| RP3-402G11.27 | sCCA3 | 415 | 14 | enet | 0.038 | 0.00039 | -2.76 | 0.00575 | 0.45 |
| RP11-170N16.3 | sCCA3 | 330 | 5 | lasso | 0.067 | 2.8e-06 | 2.75 | 0.00592 | 0.45 |
| RP11-449P15.2 | sCCA3 | 329 | 7 | lasso | 0.13 | 3.7e-11 | -2.76 | 0.005811 | 0.45 |
| SNORA44 | sCCA2 | 267 | 6 | lasso | 0.32 | 9e-28 | -2.82 | 0.004745 | 0.45 |
| SNORA44 | sCCA1 | 267 | 7 | lasso | 0.26 | 6.6e-22 | 2.79 | 0.00527 | 0.45 |
| AC004076.5 | sCCA1 | 488 | 1 | top1 | 0.55 | 2.1e-55 | -2.77 | 0.005545 | 0.45 |
| AC004076.5 | sCCA2 | 488 | 1 | top1 | 0.49 | 4.7e-47 | 2.77 | 0.005545 | 0.45 |
| AC004076.5 | sCCA3 | 488 | 1 | top1 | 0.4 | 1.6e-35 | -2.77 | 0.00555 | 0.45 |
| PABPC1 | sCCA2 | 452 | 1 | top1 | 0.068 | 2.5e-06 | 2.72 | 0.006455 | 0.46 |
| CFAP20 | sCCA3 | 528 | 4 | lasso | 0.016 | 0.016 | 2.73 | 0.00624 | 0.46 |
| ANKRD13A | sCCA1 | 299 | 32 | enet | 0.048 | 6.3e-05 | -2.73 | 0.006247 | 0.46 |
| MRPL28 | sCCA3 | 377 | 24 | enet | 0.13 | 2.8e-11 | 2.73 | 0.00639 | 0.46 |
| ZFP30 | sCCA1 | 292 | 8 | lasso | 0.38 | 4.3e-33 | -2.73 | 0.006306 | 0.46 |
| ZNF644 | sCCA3 | 418 | 7 | lasso | 0.055 | 1.9e-05 | 2.72 | 0.00644 | 0.46 |
| SMO | sCCA3 | 369 | 8 | lasso | 0.017 | 0.013 | -2.71 | 0.006777 | 0.46 |
| POMP | sCCA2 | 470 | 1 | top1 | 0.065 | 3.6e-06 | 2.73 | 0.00642 | 0.46 |
| CAB39 | sCCA1 | 499 | 17 | enet | 0.083 | 1.7e-07 | 2.73 | 0.00638 | 0.46 |
| CENPC | sCCA3 | 407 | 6 | lasso | 0.44 | 1e-39 | -2.71 | 0.00667 | 0.46 |
| IRAK1BP1 | sCCA1 | 375 | 36 | enet | 0.69 | 2.9e-80 | -2.72 | 0.00662 | 0.46 |
| CEP78 | sCCA2 | 391 | 5 | lasso | 0.1 | 5.5e-09 | -2.71 | 0.006663 | 0.46 |
| NDUFV3 | sCCA1 | 521 | 37 | enet | 0.67 | 2.3e-74 | -2.71 | 0.006636 | 0.46 |
| SHC1 | sCCA2 | 390 | 2 | lasso | 0.029 | 0.0017 | 2.71 | 0.006688 | 0.46 |
| FAM189B | sCCA1 | 300 | 1 | top1 | 0.13 | 1.2e-10 | -2.72 | 0.00647 | 0.46 |
| FAM189B | sCCA2 | 300 | 1 | top1 | 0.038 | 0.00039 | 2.72 | 0.00647 | 0.46 |
| FAM189B | sCCA3 | 300 | 1 | top1 | 0.011 | 0.04 | 2.72 | 0.00647 | 0.46 |
| MAIP1 | sCCA2 | 370 | 1 | top1 | 0.14 | 1e-11 | 2.73 | 0.00643 | 0.46 |
| ZMYM6 | sCCA1 | 299 | 18 | enet | 0.034 | 0.00076 | -2.73 | 0.00633 | 0.46 |
| BORCS5 | sCCA1 | 531 | 52 | enet | 0.4 | 1.3e-35 | -2.72 | 0.006518 | 0.46 |
| XRRA1 | sCCA1 | 456 | 1 | top1 | 0.9 | 2.7e-153 | 2.71 | 0.006716 | 0.46 |
| MAP3K2 | sCCA2 | 436 | 7 | lasso | 0.18 | 3.6e-15 | 2.73 | 0.00633 | 0.46 |
| BOK | sCCA2 | 320 | 8 | lasso | 0.27 | 3.8e-23 | 2.73 | 0.00627 | 0.46 |
| RAP2B | sCCA1 | 367 | 18 | enet | 0.059 | 1e-05 | 2.71 | 0.00672 | 0.46 |
| BTBD6 | sCCA1 | 219 | 5 | lasso | 0.25 | 1.5e-20 | 2.71 | 0.00678 | 0.46 |
| ZNF829 | sCCA2 | 283 | 39 | enet | 0.047 | 8.4e-05 | -2.72 | 0.006443 | 0.46 |
| ZNF749 | sCCA2 | 489 | 8 | lasso | 0.76 | 3.1e-95 | 2.72 | 0.006582 | 0.46 |
| GCNT1 | sCCA3 | 664 | 1 | top1 | 0.086 | 1.1e-07 | 2.71 | 0.0067 | 0.46 |
| RPL37A | sCCA3 | 508 | 4 | lasso | 0.066 | 3.3e-06 | 2.72 | 0.00649 | 0.46 |
| UCKL1 | sCCA2 | 381 | 11 | lasso | 0.13 | 9.6e-11 | -2.72 | 0.006521 | 0.46 |
| ZNF814 | sCCA2 | 467 | 34 | enet | 0.23 | 5.5e-19 | 2.73 | 0.00633 | 0.46 |
| IGLC1 | sCCA1 | 310 | 14 | enet | 0.094 | 2.7e-08 | -2.71 | 0.00671 | 0.46 |
| RP1-257A7.4 | sCCA2 | 523 | 9 | lasso | 0.37 | 1.9e-32 | 2.72 | 0.006543 | 0.46 |
| RP11-147I3.1 | sCCA2 | 454 | 5 | lasso | 0.66 | 1.2e-73 | -2.73 | 0.006381 | 0.46 |
| LILRA6 | sCCA2 | 487 | 22 | enet | 0.085 | 1.2e-07 | -2.72 | 0.006575 | 0.46 |
| RP11-341G23.4 | sCCA2 | 617 | 53 | enet | 0.52 | 9.3e-51 | -2.71 | 0.006768 | 0.46 |
| RP3-337O18.9 | sCCA3 | 515 | 36 | enet | 0.19 | 1e-15 | 2.72 | 0.00658 | 0.46 |
| LINC01578 | sCCA1 | 675 | 4 | lasso | 0.096 | 1.9e-08 | 2.73 | 0.00641 | 0.46 |
| RP11-77K12.9 | sCCA2 | 346 | 21 | enet | 0.047 | 8e-05 | -2.73 | 0.00626 | 0.46 |
| AC142472.6 | sCCA2 | 280 | 9 | lasso | 0.081 | 2.4e-07 | -2.73 | 0.006422 | 0.46 |
| RP11-1099M24.6 | sCCA2 | 507 | 8 | lasso | 0.17 | 4.5e-14 | -2.71 | 0.006742 | 0.46 |
| HHAT | sCCA1 | 544 | 6 | lasso | 0.31 | 1.7e-26 | -2.69 | 0.00719 | 0.47 |
| ACAA1 | sCCA3 | 425 | 39 | enet | 0.031 | 0.0012 | 2.68 | 0.007314 | 0.47 |
| LZTS1 | sCCA1 | 602 | 6 | lasso | 0.19 | 9.1e-16 | 2.69 | 0.00706 | 0.47 |
| KCNAB2 | sCCA1 | 511 | 8 | lasso | 0.11 | 3.7e-09 | -2.69 | 0.00716 | 0.47 |
| TELO2 | sCCA2 | 491 | 24 | enet | 0.59 | 3.4e-61 | -2.7 | 0.00683 | 0.47 |
| NUMBL | sCCA2 | 422 | 17 | enet | 0.066 | 3.4e-06 | -2.68 | 0.007303 | 0.47 |
| POLR2I | sCCA1 | 341 | 23 | enet | 0.34 | 4.7e-29 | -2.68 | 0.007282 | 0.47 |
| MEGF9 | sCCA2 | 341 | 5 | lasso | 0.36 | 6.5e-32 | -2.69 | 0.007139 | 0.47 |
| FNBP4 | sCCA2 | 282 | 1 | top1 | 0.094 | 3e-08 | 2.68 | 0.007262 | 0.47 |
| CYP27B1 | sCCA1 | 346 | 6 | lasso | 0.45 | 6.7e-42 | -2.68 | 0.00745 | 0.47 |
| SRSF3 | sCCA3 | 564 | 1 | top1 | -0.0011 | 0.42 | 2.69 | 0.00717 | 0.47 |
| SMAP1 | sCCA1 | 487 | 1 | top1 | 0.23 | 6.6e-19 | 2.68 | 0.00746 | 0.47 |
| GON4L | sCCA1 | 259 | 7 | lasso | 0.045 | 0.00011 | 2.7 | 0.00693 | 0.47 |
| MAP7D1 | sCCA1 | 322 | 1 | top1 | 0.29 | 1.1e-24 | 2.68 | 0.00744 | 0.47 |
| PQLC1 | sCCA2 | 318 | 6 | lasso | 0.074 | 8e-07 | 2.68 | 0.00734 | 0.47 |
| ACSL3 | sCCA1 | 440 | 1 | top1 | 0.17 | 2.1e-14 | -2.69 | 0.00716 | 0.47 |
| NEURL2 | sCCA1 | 514 | 28 | enet | 0.28 | 5.3e-24 | 2.7 | 0.00688 | 0.47 |
| TTBK2 | sCCA3 | 282 | 11 | lasso | 0.1 | 9.6e-09 | -2.69 | 0.00709 | 0.47 |
| SELENOS | sCCA2 | 614 | 1 | top1 | 0.015 | 0.017 | 2.69 | 0.00724 | 0.47 |
| RRAS2 | sCCA3 | 376 | 15 | lasso | 0.31 | 9.5e-27 | -2.69 | 0.00717 | 0.47 |
| OASL | sCCA3 | 453 | 13 | lasso | -8.50E-05 | 0.32 | 2.69 | 0.00717 | 0.47 |
| KIF13A | sCCA1 | 456 | 23 | enet | 0.25 | 3.8e-21 | 2.7 | 0.00688 | 0.47 |
| COL8A1 | sCCA1 | 401 | 18 | enet | 0.16 | 1.4e-13 | 2.69 | 0.00718 | 0.47 |
| LPP | sCCA3 | 514 | 27 | enet | 0.069 | 2e-06 | 2.68 | 0.007295 | 0.47 |
| IRAK1BP1 | sCCA3 | 375 | 14 | lasso | 0.61 | 1.1e-63 | -2.69 | 0.0072 | 0.47 |
| CCDC3 | sCCA1 | 649 | 31 | enet | 0.27 | 1.2e-22 | 2.68 | 0.00726 | 0.47 |
| SASS6 | sCCA3 | 450 | 14 | lasso | 0.11 | 1.2e-09 | 2.69 | 0.00709 | 0.47 |
| FUBP1 | sCCA2 | 332 | 41 | enet | 0.21 | 1.1e-17 | 2.68 | 0.007271 | 0.47 |
| C1orf74 | sCCA3 | 513 | 1 | top1 | 0.047 | 8.1e-05 | 2.69 | 0.00722 | 0.47 |
| WBP1L | sCCA1 | 358 | 14 | enet | 0.21 | 3.5e-17 | -2.7 | 0.00699 | 0.47 |
| XRRA1 | sCCA2 | 456 | 6 | lasso | 0.87 | 2.3e-135 | -2.69 | 0.00711 | 0.47 |
| CENPV | sCCA1 | 282 | 29 | enet | 0.54 | 9e-53 | 2.68 | 0.00727 | 0.47 |
| P2RY12 | sCCA2 | 501 | 33 | enet | 0.3 | 7.4e-26 | -2.69 | 0.007169 | 0.47 |
| LINC00116 | sCCA1 | 73 | 1 | top1 | 0.054 | 2.4e-05 | 2.68 | 0.00741 | 0.47 |
| LINC00116 | sCCA2 | 73 | 1 | top1 | 0.051 | 4.2e-05 | -2.68 | 0.00741 | 0.47 |
| GGN | sCCA2 | 372 | 17 | lasso | 0.072 | 1.2e-06 | 2.69 | 0.007199 | 0.47 |
| C3orf62 | sCCA3 | 276 | 10 | enet | 0.018 | 0.011 | -2.68 | 0.007266 | 0.47 |
| RP4-530I15.9 | sCCA1 | 449 | 14 | enet | 0.15 | 1.9e-12 | 2.68 | 0.00732 | 0.47 |
| HMBS | sCCA1 | 376 | 52 | enet | 0.6 | 3.2e-62 | -2.69 | 0.007226 | 0.47 |
| RP11-341G23.4 | sCCA3 | 617 | 34 | enet | 0.67 | 8.4e-76 | 2.69 | 0.00713 | 0.47 |
| RP13-890H12.2 | sCCA3 | 276 | 23 | enet | 0.064 | 4.9e-06 | -2.7 | 0.006864 | 0.47 |
| RP11-571M6.17 | sCCA3 | 350 | 7 | lasso | 0.0096 | 0.048 | -2.69 | 0.00724 | 0.47 |
| RP11-521B24.4 | sCCA2 | 183 | 1 | top1 | 0.076 | 6.2e-07 | -2.68 | 0.00728 | 0.47 |
| CTD-2095E4.4 | sCCA1 | 299 | 1 | top1 | 0.057 | 1.5e-05 | -2.69 | 0.00719 | 0.47 |
| PDK4 | sCCA1 | 505 | 1 | top1 | 0.13 | 7.8e-11 | -2.64 | 0.00835 | 0.48 |
| NCAPH2 | sCCA2 | 328 | 16 | enet | 0.038 | 0.00034 | -2.67 | 0.007531 | 0.48 |
| LTBP1 | sCCA1 | 490 | 1 | top1 | 0.29 | 1.3e-24 | -2.67 | 0.00765 | 0.48 |
| TRAF3IP2 | sCCA1 | 437 | 4 | lasso | 0.19 | 1.8e-15 | -2.63 | 0.00849 | 0.48 |
| SEC61A1 | sCCA1 | 397 | 1 | top1 | 0.18 | 8.2e-15 | 2.65 | 0.00811 | 0.48 |
| RFXANK | sCCA3 | 327 | 27 | enet | 0.041 | 0.00022 | 2.63 | 0.00849 | 0.48 |
| KCNAB2 | sCCA3 | 511 | 3 | lasso | 0.11 | 1.4e-09 | 2.63 | 0.00842 | 0.48 |
| NSF | sCCA1 | 146 | 8 | lasso | 0.2 | 6.7e-17 | 2.66 | 0.00779 | 0.48 |
| SLC25A17 | sCCA1 | 277 | 8 | lasso | 0.19 | 3.6e-16 | 2.65 | 0.00817 | 0.48 |
| GOSR2 | sCCA3 | 286 | 15 | enet | 0.37 | 1.7e-32 | -2.63 | 0.008432 | 0.48 |
| TMEM104 | sCCA2 | 449 | 6 | lasso | 0.04 | 0.00027 | -2.64 | 0.008364 | 0.48 |
| SRSF3 | sCCA1 | 564 | 8 | lasso | 0.14 | 8.6e-12 | -2.64 | 0.00822 | 0.48 |
| BACH2 | sCCA2 | 528 | 1 | top1 | 0.015 | 0.019 | -2.66 | 0.007769 | 0.48 |
| SMOC2 | sCCA1 | 665 | 13 | lasso | 0.37 | 2e-32 | -2.65 | 0.00808 | 0.48 |
| PDE8B | sCCA2 | 517 | 10 | lasso | 0.33 | 7.6e-29 | -2.64 | 0.008389 | 0.48 |
| PDGFRB | sCCA1 | 568 | 1 | top1 | 0.02 | 0.0077 | -2.66 | 0.007739 | 0.48 |
| IGFBP5 | sCCA1 | 524 | 42 | enet | 0.073 | 1.1e-06 | 2.64 | 0.00831 | 0.48 |
| CHMP3 | sCCA2 | 334 | 35 | enet | 0.38 | 4.9e-33 | -2.63 | 0.00843 | 0.48 |
| RND3 | sCCA1 | 451 | 15 | lasso | 0.036 | 5e-04 | -2.65 | 0.00801 | 0.48 |
| SCAMP3 | sCCA2 | 299 | 4 | lasso | 0.078 | 4.5e-07 | -2.63 | 0.008566 | 0.48 |
| IRF2BPL | sCCA3 | 548 | 1 | top1 | 0.057 | 1.5e-05 | -2.63 | 0.008423 | 0.48 |
| MXI1 | sCCA2 | 372 | 6 | lasso | 0.071 | 1.4e-06 | -2.67 | 0.00753 | 0.48 |
| TSFM | sCCA1 | 348 | 1 | top1 | 0.68 | 1.5e-76 | -2.65 | 0.007956 | 0.48 |
| METTL21B | sCCA2 | 348 | 3 | lasso | 0.56 | 6.9e-57 | -2.65 | 0.008004 | 0.48 |
| METTL21B | sCCA3 | 348 | 6 | lasso | 0.72 | 5.3e-87 | -2.63 | 0.00844 | 0.48 |
| METTL21B | sCCA1 | 348 | 3 | lasso | 0.79 | 1.2e-103 | 2.63 | 0.008584 | 0.48 |
| AGO2 | sCCA3 | 369 | 8 | lasso | 0.083 | 2e-07 | -2.64 | 0.00818 | 0.48 |
| CPNE5 | sCCA1 | 550 | 10 | lasso | 0.3 | 1.3e-25 | 2.63 | 0.00853 | 0.48 |
| NRN1 | sCCA2 | 703 | 1 | top1 | 0.13 | 5e-11 | -2.66 | 0.007786 | 0.48 |
| SGPP1 | sCCA1 | 385 | 1 | top1 | 0.011 | 0.034 | -2.65 | 0.00801 | 0.48 |
| PRKCSH | sCCA3 | 392 | 16 | enet | 0.12 | 2e-10 | 2.64 | 0.00829 | 0.48 |
| PRKCSH | sCCA2 | 392 | 18 | enet | 0.11 | 1.6e-09 | 2.63 | 0.008633 | 0.48 |
| SLC6A6 | sCCA2 | 589 | 1 | top1 | 0.073 | 1e-06 | -2.65 | 0.008157 | 0.48 |
| SYT11 | sCCA1 | 259 | 1 | top1 | 0.055 | 2e-05 | 2.63 | 0.00857 | 0.48 |
| KIAA1468 | sCCA1 | 508 | 12 | lasso | 0.38 | 3.2e-33 | -2.63 | 0.00862 | 0.48 |
| CEP162 | sCCA1 | 385 | 1 | top1 | 0.045 | 0.00011 | -2.63 | 0.00848 | 0.48 |
| TSPAN31 | sCCA1 | 342 | 1 | top1 | 0.1 | 4.8e-09 | -2.67 | 0.007618 | 0.48 |
| SCEL | sCCA1 | 372 | 29 | enet | 0.091 | 5e-08 | -2.64 | 0.00828 | 0.48 |
| RSL24D1 | sCCA2 | 439 | 23 | lasso | 0.24 | 4.9e-20 | 2.66 | 0.00791 | 0.48 |
| GSTCD | sCCA3 | 403 | 38 | enet | 0.021 | 0.006 | 2.66 | 0.00771 | 0.48 |
| MOB3C | sCCA1 | 362 | 27 | enet | 0.34 | 4.6e-29 | -2.63 | 0.00844 | 0.48 |
| SNRNP200 | sCCA2 | 156 | 10 | lasso | 0.057 | 1.6e-05 | 2.63 | 0.00866 | 0.48 |
| TATDN1 | sCCA3 | 524 | 1 | top1 | 0.049 | 5.9e-05 | -2.66 | 0.00789 | 0.48 |
| ATM | sCCA2 | 345 | 3 | lasso | 0.086 | 1.1e-07 | 2.66 | 0.007912 | 0.48 |
| MPP7 | sCCA2 | 511 | 52 | enet | 0.22 | 3.3e-18 | -2.63 | 0.00862 | 0.48 |
| ITGB1 | sCCA3 | 478 | 13 | lasso | 0.2 | 9e-17 | 2.63 | 0.00851 | 0.48 |
| ABCA6 | sCCA1 | 466 | 3 | lasso | 0.26 | 2.2e-21 | 2.63 | 0.008617 | 0.48 |
| NEIL2 | sCCA1 | 476 | 8 | lasso | 0.53 | 4.3e-52 | 2.64 | 0.0084 | 0.48 |
| ADK | sCCA1 | 236 | 5 | lasso | 0.26 | 1.1e-21 | -2.66 | 0.00774 | 0.48 |
| CCT8 | sCCA2 | 426 | 1 | top1 | 0.57 | 3.6e-58 | 2.64 | 0.00823 | 0.48 |
| CCT8 | sCCA3 | 426 | 1 | top1 | 0.71 | 1e-83 | 2.64 | 0.008231 | 0.48 |
| MAPK13 | sCCA3 | 372 | 5 | lasso | 0.43 | 7.7e-39 | -2.66 | 0.00772 | 0.48 |
| SASS6 | sCCA2 | 450 | 12 | lasso | 0.21 | 2.5e-17 | 2.66 | 0.007704 | 0.48 |
| UBR1 | sCCA1 | 299 | 4 | lasso | 0.1 | 6.4e-09 | -2.65 | 0.00806 | 0.48 |
| GPATCH4 | sCCA3 | 445 | 6 | lasso | 0.079 | 3.4e-07 | 2.63 | 0.00851 | 0.48 |
| TYW5 | sCCA2 | 364 | 41 | enet | 0.11 | 3.1e-09 | 2.66 | 0.00777 | 0.48 |
| PAQR3 | sCCA2 | 349 | 28 | enet | 0.058 | 1.2e-05 | -2.63 | 0.00857 | 0.48 |
| C10orf25 | sCCA2 | 456 | 6 | lasso | 0.098 | 1.3e-08 | -2.65 | 0.00814 | 0.48 |
| FAM227B | sCCA1 | 367 | 27 | enet | 0.33 | 7.5e-29 | -2.67 | 0.00768 | 0.48 |
| MBD6 | sCCA2 | 330 | 15 | enet | 0.052 | 3.5e-05 | -2.64 | 0.008225 | 0.48 |
| PROSER3 | sCCA3 | 426 | 7 | lasso | 0.37 | 1.5e-32 | 2.64 | 0.00839 | 0.48 |
| C8orf31 | sCCA1 | 408 | 5 | lasso | 0.29 | 2.1e-24 | -2.63 | 0.00847 | 0.48 |
| GBA | sCCA2 | 314 | 4 | lasso | 0.11 | 2.7e-09 | -2.67 | 0.007695 | 0.48 |
| HKR1 | sCCA2 | 284 | 1 | top1 | 0.056 | 1.7e-05 | 2.66 | 0.007846 | 0.48 |
| COA4 | sCCA1 | 341 | 17 | enet | 0.13 | 2.7e-11 | -2.63 | 0.008471 | 0.48 |
| CMSS1 | sCCA2 | 403 | 1 | top1 | 0.21 | 9.5e-18 | -2.65 | 0.007979 | 0.48 |
| ZNF395 | sCCA2 | 476 | 1 | top1 | 0.046 | 9.3e-05 | -2.63 | 0.008456 | 0.48 |
| ZNF383 | sCCA1 | 276 | 1 | top1 | 0.031 | 0.0013 | 2.65 | 0.008034 | 0.48 |
| C15orf52 | sCCA1 | 397 | 31 | enet | 0.05 | 5.2e-05 | -2.64 | 0.00841 | 0.48 |
| GTF2IRD2 | sCCA3 | 103 | 14 | enet | 0.61 | 6.2e-65 | 2.67 | 0.007554 | 0.48 |
| GTF2IRD2 | sCCA1 | 103 | 16 | enet | 0.75 | 2.2e-92 | -2.64 | 0.00821 | 0.48 |
| SLC5A3 | sCCA3 | 401 | 24 | enet | 0.039 | 0.00029 | 2.64 | 0.008403 | 0.48 |
| LRRC73 | sCCA2 | 424 | 22 | enet | 0.02 | 0.0073 | 2.62 | 0.008703 | 0.48 |
| HMGN1 | sCCA2 | 668 | 19 | enet | 0.043 | 0.00016 | 2.66 | 0.00784 | 0.48 |
| AC009961.3 | sCCA1 | 469 | 59 | enet | 0.63 | 8.9e-67 | 2.67 | 0.00761 | 0.48 |
| RP1-28O10.1 | sCCA3 | 514 | 17 | enet | 0.054 | 2.4e-05 | -2.66 | 0.0079 | 0.48 |
| AP001469.7 | sCCA3 | 472 | 5 | lasso | 0.059 | 1.1e-05 | 2.67 | 0.007678 | 0.48 |
| RP11-147I3.1 | sCCA3 | 454 | 8 | lasso | 0.76 | 3.6e-96 | -2.66 | 0.00774 | 0.48 |
| CDK11B | sCCA3 | 308 | 27 | enet | 0.26 | 1.2e-21 | 2.65 | 0.00801 | 0.48 |
| CDK11B | sCCA1 | 308 | 25 | enet | 0.32 | 1.2e-27 | -2.63 | 0.00842 | 0.48 |
| RP11-73K9.2 | sCCA1 | 386 | 1 | top1 | 0.045 | 0.00011 | 2.65 | 0.00795 | 0.48 |
| RP11-686D22.4 | sCCA2 | 368 | 28 | enet | 0.34 | 3.3e-29 | 2.63 | 0.008604 | 0.48 |
| RP1-257I20.14 | sCCA1 | 340 | 37 | enet | 0.46 | 2.6e-42 | -2.64 | 0.00833 | 0.48 |
| TYROBP | sCCA1 | 377 | 27 | enet | 0.1 | 6.8e-09 | -2.61 | 0.009116 | 0.49 |
| UNG | sCCA2 | 438 | 5 | lasso | 0.055 | 2e-05 | -2.62 | 0.008819 | 0.49 |
| ASAP3 | sCCA3 | 299 | 11 | enet | 0.17 | 1.5e-14 | 2.62 | 0.00884 | 0.49 |
| TBC1D12 | sCCA3 | 335 | 20 | enet | 0.036 | 5e-04 | -2.62 | 0.00875 | 0.49 |
| CDCA3 | sCCA2 | 223 | 66 | enet | 0.29 | 2.7e-24 | -2.61 | 0.008932 | 0.49 |
| PODXL | sCCA3 | 481 | 1 | top1 | 0.014 | 0.021 | -2.61 | 0.008949 | 0.49 |
| CLIP1 | sCCA2 | 337 | 16 | enet | 0.11 | 1.9e-09 | -2.6 | 0.00919 | 0.49 |
| UBE4B | sCCA2 | 320 | 1 | top1 | 0.078 | 4.7e-07 | 2.61 | 0.009041 | 0.49 |
| TIMM10B | sCCA3 | 654 | 1 | top1 | 0.19 | 3.9e-16 | -2.61 | 0.00912 | 0.49 |
| KIF13A | sCCA3 | 456 | 7 | lasso | 0.14 | 7.8e-12 | -2.62 | 0.00874 | 0.49 |
| NKD2 | sCCA3 | 371 | 20 | enet | 0.088 | 7.4e-08 | -2.61 | 0.009181 | 0.49 |
| GNA12 | sCCA2 | 563 | 7 | lasso | 0.47 | 4e-44 | 2.61 | 0.009087 | 0.49 |
| FSIP1 | sCCA1 | 488 | 1 | top1 | 0.094 | 2.8e-08 | 2.62 | 0.0088 | 0.49 |
| ATXN7 | sCCA1 | 602 | 8 | lasso | 0.17 | 6.6e-14 | 2.61 | 0.00905 | 0.49 |
| ARMC10 | sCCA3 | 240 | 5 | lasso | 0.24 | 4e-20 | -2.62 | 0.008753 | 0.49 |
| PODN | sCCA3 | 479 | 23 | enet | 0.12 | 2.7e-10 | 2.62 | 0.00886 | 0.49 |
| DNHD1 | sCCA3 | 645 | 1 | top1 | 0.06 | 9.7e-06 | 2.61 | 0.00912 | 0.49 |
| CSF1R | sCCA1 | 585 | 34 | enet | 0.13 | 4.3e-11 | -2.62 | 0.008858 | 0.49 |
| CMSS1 | sCCA1 | 403 | 1 | top1 | 0.26 | 6.8e-22 | -2.61 | 0.00911 | 0.49 |
| ZNF420 | sCCA1 | 294 | 1 | top1 | 0.049 | 6e-05 | 2.62 | 0.008816 | 0.49 |
| ZNF461 | sCCA2 | 289 | 1 | top1 | 0.078 | 4.4e-07 | 2.62 | 0.008874 | 0.49 |
| RP11-147I3.1 | sCCA1 | 454 | 20 | enet | 0.76 | 6.4e-96 | -2.61 | 0.009108 | 0.49 |
| CTD-2516F10.2 | sCCA3 | 660 | 18 | lasso | 0.61 | 6.4e-65 | -2.62 | 0.00882 | 0.49 |
| CTD-2555O16.2 | sCCA3 | 494 | 7 | lasso | 0.03 | 0.0014 | -2.62 | 0.008813 | 0.49 |
| GLRX2 | sCCA3 | 348 | 8 | lasso | 0.051 | 4.3e-05 | 2.6 | 0.00935 | 0.5 |
| METTL1 | sCCA1 | 348 | 1 | top1 | 0.23 | 1.8e-19 | -2.6 | 0.009329 | 0.5 |
| MAP3K4 | sCCA2 | 520 | 23 | enet | 0.12 | 1.9e-10 | 2.59 | 0.009641 | 0.5 |
| GLI1 | sCCA1 | 330 | 5 | lasso | 0.12 | 4e-10 | 2.59 | 0.009737 | 0.5 |
| WIPF1 | sCCA1 | 330 | 10 | lasso | 0.29 | 3.3e-24 | 2.59 | 0.00955 | 0.5 |
| TSFM | sCCA2 | 348 | 1 | top1 | 0.47 | 9.2e-44 | -2.6 | 0.009329 | 0.5 |
| TSFM | sCCA3 | 348 | 1 | top1 | 0.4 | 2.9e-35 | -2.6 | 0.00933 | 0.5 |
| KRT17 | sCCA1 | 427 | 5 | lasso | 0.036 | 0.00051 | -2.59 | 0.009593 | 0.5 |
| HIP1R | sCCA2 | 299 | 12 | enet | 0.011 | 0.036 | -2.6 | 0.009399 | 0.5 |
| TCF19 | sCCA2 | 173 | 24 | enet | 0.36 | 4.9e-31 | 2.6 | 0.00937 | 0.5 |
| WDR89 | sCCA2 | 378 | 13 | enet | 0.076 | 5.8e-07 | 2.59 | 0.00947 | 0.5 |
| DUSP10 | sCCA3 | 474 | 8 | lasso | 0.025 | 0.0032 | 2.6 | 0.00927 | 0.5 |
| AP1S3 | sCCA1 | 453 | 37 | enet | 0.29 | 3.7e-24 | 2.59 | 0.00972 | 0.5 |
| GDPD1 | sCCA2 | 297 | 1 | top1 | 0.12 | 1.8e-10 | -2.6 | 0.009309 | 0.5 |
| NEIL2 | sCCA3 | 476 | 7 | lasso | 0.53 | 6e-52 | 2.59 | 0.00963 | 0.5 |
| CABP1 | sCCA3 | 419 | 33 | enet | 0.03 | 0.0014 | 2.59 | 0.00947 | 0.5 |
| NCSTN | sCCA2 | 498 | 25 | enet | 0.15 | 2.5e-12 | 2.59 | 0.009662 | 0.5 |
| PMVK | sCCA3 | 398 | 1 | top1 | 0.0049 | 0.11 | -2.59 | 0.00948 | 0.5 |
| COPS2 | sCCA1 | 334 | 6 | lasso | 0.023 | 0.0046 | 2.6 | 0.00942 | 0.5 |
| C2CD3 | sCCA1 | 407 | 48 | enet | 0.24 | 8.4e-20 | -2.6 | 0.009453 | 0.5 |
| EFNA1 | sCCA3 | 353 | 1 | top1 | 0.052 | 3.4e-05 | 2.59 | 0.0097 | 0.5 |
| EID2B | sCCA2 | 430 | 9 | lasso | 0.039 | 0.00032 | 2.59 | 0.009715 | 0.5 |
| AC025335.1 | sCCA1 | 505 | 9 | lasso | 0.16 | 1.2e-13 | -2.59 | 0.009627 | 0.5 |
| BRF1 | sCCA1 | 193 | 8 | lasso | 0.33 | 5.4e-28 | -2.59 | 0.0097 | 0.5 |
| RP11-574K11.5 | sCCA1 | 239 | 12 | lasso | 0.11 | 2.2e-09 | -2.6 | 0.00925 | 0.5 |
| RP11-159D12.2 | sCCA1 | 481 | 44 | enet | 0.049 | 5.6e-05 | -2.59 | 0.009523 | 0.5 |
| CTC-444N24.8 | sCCA2 | 502 | 5 | lasso | 0.07 | 1.7e-06 | -2.59 | 0.00954 | 0.5 |
| SOCS7 | sCCA1 | 184 | 17 | enet | 0.061 | 7.5e-06 | -2.59 | 0.009511 | 0.5 |
| RP1-198K11.5 | sCCA3 | 392 | 26 | enet | 0.041 | 0.00022 | -2.6 | 0.00928 | 0.5 |
| MASP2 | sCCA3 | 374 | 24 | enet | 0.066 | 3.1e-06 | 2.57 | 0.0103 | 0.51 |
| FYN | sCCA2 | 507 | 18 | enet | -0.0026 | 0.65 | -2.57 | 0.010242 | 0.51 |
| BRD9 | sCCA3 | 372 | 26 | enet | 0.19 | 8.5e-16 | 2.58 | 0.00981 | 0.51 |
| WDR62 | sCCA2 | 337 | 34 | enet | 0.23 | 5.3e-19 | 2.56 | 0.010486 | 0.51 |
| STARD7 | sCCA2 | 152 | 14 | enet | 0.052 | 3.4e-05 | 2.57 | 0.0102 | 0.51 |
| ZC3H7B | sCCA2 | 260 | 1 | top1 | 0.14 | 4.4e-12 | 2.57 | 0.010238 | 0.51 |
| NDRG4 | sCCA1 | 479 | 10 | lasso | 0.21 | 2.1e-17 | -2.58 | 0.00984 | 0.51 |
| SPAG1 | sCCA2 | 370 | 35 | enet | 0.41 | 3e-37 | 2.58 | 0.009996 | 0.51 |
| GOSR2 | sCCA1 | 286 | 15 | enet | 0.39 | 6e-35 | -2.58 | 0.00983 | 0.51 |
| MANSC1 | sCCA1 | 537 | 8 | lasso | 0.085 | 1.3e-07 | 2.56 | 0.010539 | 0.51 |
| MXI1 | sCCA1 | 372 | 26 | enet | 0.24 | 2e-20 | -2.58 | 0.00995 | 0.51 |
| KANSL1 | sCCA1 | 46 | 17 | enet | 0.36 | 2.1e-31 | -2.56 | 0.010483 | 0.51 |
| TIMM10B | sCCA2 | 654 | 1 | top1 | 0.2 | 2.3e-16 | 2.56 | 0.010588 | 0.51 |
| SAA2 | sCCA2 | 430 | 11 | enet | 0.048 | 6.2e-05 | 2.58 | 0.009869 | 0.51 |
| HAUS2 | sCCA2 | 332 | 27 | enet | 0.097 | 1.6e-08 | -2.57 | 0.01005 | 0.51 |
| RSL24D1 | sCCA1 | 439 | 17 | lasso | 0.48 | 1.7e-45 | 2.57 | 0.01014 | 0.51 |
| CIR1 | sCCA1 | 377 | 15 | enet | 0.14 | 1.3e-11 | 2.58 | 0.00994 | 0.51 |
| CASP6 | sCCA1 | 394 | 17 | enet | 0.16 | 1.5e-13 | 2.58 | 0.00995 | 0.51 |
| TMTC3 | sCCA3 | 290 | 4 | lasso | 0.17 | 1.5e-14 | -2.56 | 0.0104 | 0.51 |
| DENR | sCCA1 | 292 | 3 | lasso | 0.05 | 4.8e-05 | 2.58 | 0.009997 | 0.51 |
| SCUBE3 | sCCA2 | 416 | 33 | enet | 0.042 | 0.00018 | -2.58 | 0.009983 | 0.51 |
| COMMD3 | sCCA3 | 279 | 7 | enet | 0.025 | 0.0034 | -2.57 | 0.0101 | 0.51 |
| TOMM70 | sCCA3 | 393 | 6 | lasso | 0.31 | 2.6e-26 | 2.58 | 0.009935 | 0.51 |
| RNF207 | sCCA3 | 450 | 12 | lasso | 0.27 | 2.8e-22 | -2.57 | 0.0101 | 0.51 |
| RUSC1 | sCCA1 | 260 | 1 | top1 | 0.061 | 8.1e-06 | 2.58 | 0.01 | 0.51 |
| C4orf36 | sCCA2 | 320 | 17 | enet | 0.067 | 2.6e-06 | 2.56 | 0.0104 | 0.51 |
| GUCY1A3 | sCCA1 | 438 | 32 | enet | 0.17 | 7.7e-14 | 2.57 | 0.01029 | 0.51 |
| SLC13A4 | sCCA2 | 467 | 39 | enet | 0.17 | 3.6e-14 | -2.56 | 0.010526 | 0.51 |
| C2CD3 | sCCA3 | 407 | 16 | lasso | 0.2 | 1.7e-16 | -2.56 | 0.01052 | 0.51 |
| P2RY12 | sCCA3 | 501 | 35 | enet | 0.52 | 6.9e-50 | 2.57 | 0.010156 | 0.51 |
| ZEB2 | sCCA2 | 264 | 28 | enet | 0.064 | 4.5e-06 | 2.57 | 0.0101 | 0.51 |
| BCL2 | sCCA3 | 499 | 5 | lasso | 0.034 | 0.00072 | 2.58 | 0.00999 | 0.51 |
| ATPAF2 | sCCA3 | 349 | 15 | lasso | 0.46 | 4.7e-42 | -2.57 | 0.010105 | 0.51 |
| UCP3 | sCCA3 | 378 | 19 | enet | 0.057 | 1.6e-05 | -2.56 | 0.01038 | 0.51 |
| C9orf139 | sCCA3 | 363 | 1 | top1 | 0.044 | 0.00013 | -2.57 | 0.01023 | 0.51 |
| PCBP3 | sCCA2 | 487 | 36 | enet | 0.42 | 1.1e-37 | 2.56 | 0.01052 | 0.51 |
| WDR86 | sCCA1 | 523 | 49 | enet | 0.39 | 4.5e-34 | 2.57 | 0.01021 | 0.51 |
| LTN1 | sCCA1 | 426 | 13 | enet | 0.089 | 6.7e-08 | -2.56 | 0.010457 | 0.51 |
| U3 | sCCA2 | 382 | 8 | lasso | 0.081 | 2.7e-07 | 2.57 | 0.0101 | 0.51 |
| ZNF814 | sCCA1 | 467 | 27 | enet | 0.3 | 2.5e-25 | 2.57 | 0.010187 | 0.51 |
| STAG3L1 | sCCA3 | 160 | 53 | enet | 0.42 | 1.9e-37 | 2.56 | 0.010399 | 0.51 |
| RPS7P11 | sCCA1 | 189 | 1 | top1 | 0.047 | 8e-05 | 2.57 | 0.010093 | 0.51 |
| MIR181A2HG | sCCA2 | 340 | 24 | enet | 0.11 | 1.6e-09 | 2.57 | 0.010284 | 0.51 |
| RUSC1-AS1 | sCCA1 | 260 | 23 | enet | 0.35 | 3.4e-30 | -2.58 | 0.00995 | 0.51 |
| AC009961.3 | sCCA2 | 469 | 68 | enet | 0.52 | 1.1e-50 | -2.56 | 0.0105 | 0.51 |
| CTD-2152M20.2 | sCCA2 | 558 | 1 | top1 | 0.017 | 0.012 | -2.57 | 0.010302 | 0.51 |
| RP11-326C3.11 | sCCA1 | 328 | 16 | enet | 0.51 | 6.5e-49 | 2.56 | 0.010479 | 0.51 |
| DCP1A | sCCA2 | 406 | 25 | enet | 0.089 | 6.4e-08 | 2.56 | 0.010584 | 0.51 |
| RP11-1275H24.3 | sCCA1 | 328 | 18 | enet | -0.0027 | 0.67 | 2.56 | 0.01033 | 0.51 |
| RP11-93B14.9 | sCCA3 | 536 | 20 | enet | 0.087 | 8.7e-08 | -2.57 | 0.01013 | 0.51 |
| TTC19 | sCCA3 | 277 | 15 | enet | 0.059 | 1.1e-05 | -2.55 | 0.010743 | 0.52 |
| RTFDC1 | sCCA1 | 583 | 2 | lasso | 0.38 | 4.1e-33 | 2.53 | 0.01128 | 0.52 |
| IP6K2 | sCCA1 | 273 | 7 | lasso | 0.15 | 1.5e-12 | 2.53 | 0.0113 | 0.52 |
| ITPKC | sCCA1 | 429 | 10 | enet | 0.49 | 6.3e-46 | -2.55 | 0.010917 | 0.52 |
| PAPOLA | sCCA3 | 553 | 1 | top1 | 0.12 | 6.1e-10 | 2.55 | 0.010779 | 0.52 |
| MEGF9 | sCCA3 | 341 | 12 | lasso | 0.067 | 2.7e-06 | -2.55 | 0.01075 | 0.52 |
| HLF | sCCA3 | 540 | 5 | lasso | 0.015 | 0.018 | -2.54 | 0.010977 | 0.52 |
| PRRC2C | sCCA3 | 591 | 23 | enet | 0.097 | 1.7e-08 | 2.54 | 0.0111 | 0.52 |
| LDAH | sCCA1 | 530 | 53 | enet | 0.66 | 2.8e-73 | -2.55 | 0.0107 | 0.52 |
| OGFRL1 | sCCA3 | 473 | 37 | enet | 0.094 | 2.6e-08 | 2.54 | 0.01097 | 0.52 |
| KANSL1 | sCCA2 | 46 | 20 | enet | 0.36 | 2e-31 | -2.54 | 0.011134 | 0.52 |
| NCOA3 | sCCA1 | 451 | 15 | enet | 0.33 | 4.1e-28 | -2.54 | 0.01116 | 0.52 |
| HIF3A | sCCA2 | 423 | 32 | enet | 0.025 | 0.0034 | -2.55 | 0.010694 | 0.52 |
| MVB12A | sCCA3 | 441 | 12 | enet | 0.31 | 4.6e-26 | -2.53 | 0.0113 | 0.52 |
| CENPC | sCCA2 | 407 | 33 | enet | 0.45 | 3.6e-41 | 2.54 | 0.01118 | 0.52 |
| ZNF577 | sCCA3 | 616 | 10 | lasso | 0.075 | 7e-07 | 2.54 | 0.0112 | 0.52 |
| NEXN | sCCA2 | 352 | 1 | top1 | 0.067 | 3e-06 | -2.55 | 0.010733 | 0.52 |
| TYW5 | sCCA1 | 364 | 10 | lasso | 0.3 | 1.6e-25 | -2.54 | 0.0112 | 0.52 |
| RNF181 | sCCA1 | 477 | 22 | enet | 0.053 | 2.9e-05 | -2.55 | 0.0109 | 0.52 |
| INO80E | sCCA1 | 202 | 46 | enet | 0.62 | 2.4e-66 | 2.53 | 0.01128 | 0.52 |
| ZNF439 | sCCA1 | 315 | 36 | enet | 0.00021 | 0.3 | 2.55 | 0.010925 | 0.52 |
| PTGER4 | sCCA1 | 435 | 32 | enet | 0.13 | 5.1e-11 | 2.54 | 0.010963 | 0.52 |
| DDX60L | sCCA2 | 524 | 52 | enet | 0.16 | 2.3e-13 | 2.54 | 0.01116 | 0.52 |
| SPDYE12P | sCCA1 | 87 | 31 | enet | 0.36 | 3.2e-31 | -2.55 | 0.01087 | 0.52 |
| PTPN1 | sCCA1 | 447 | 4 | lasso | 0.1 | 4.6e-09 | 2.54 | 0.011 | 0.52 |
| TAS2R15P | sCCA1 | 381 | 8 | lasso | 0.35 | 2.8e-30 | -2.55 | 0.010846 | 0.52 |
| KANSL1-AS1 | sCCA2 | 29 | 11 | lasso | 0.77 | 1.1e-99 | -2.55 | 0.010813 | 0.52 |
| IDI2-AS1 | sCCA3 | 503 | 17 | enet | 0.014 | 0.021 | -2.53 | 0.0113 | 0.52 |
| ASH1L-AS1 | sCCA3 | 223 | 16 | enet | 0.012 | 0.031 | -2.53 | 0.0113 | 0.52 |
| UGDH-AS1 | sCCA2 | 353 | 30 | enet | 0.095 | 2.2e-08 | -2.53 | 0.01126 | 0.52 |
| RP11-326C3.11 | sCCA2 | 328 | 16 | enet | 0.43 | 5.9e-39 | 2.55 | 0.01092 | 0.52 |
| RP11-872J21.3 | sCCA1 | 553 | 1 | top1 | 0.13 | 6.2e-11 | -2.54 | 0.01104 | 0.52 |
| AC002398.13 | sCCA2 | 428 | 19 | enet | 0.14 | 3.6e-12 | -2.54 | 0.010978 | 0.52 |
| RP3-337O18.9 | sCCA1 | 515 | 12 | lasso | 0.27 | 3.8e-23 | 2.53 | 0.01131 | 0.52 |
| RHBDD2 | sCCA3 | 294 | 4 | lasso | 0.045 | 1e-04 | 2.5 | 0.012323 | 0.53 |
| PIAS1 | sCCA3 | 449 | 4 | lasso | 0.29 | 1.6e-24 | 2.52 | 0.01171 | 0.53 |
| CLEC16A | sCCA3 | 560 | 26 | enet | 0.011 | 0.04 | 2.48 | 0.013 | 0.53 |
| IKBKAP | sCCA3 | 609 | 29 | enet | 0.34 | 2.4e-29 | -2.51 | 0.01191 | 0.53 |
| PABPC1 | sCCA1 | 452 | 62 | enet | 0.11 | 1.8e-09 | -2.47 | 0.0136 | 0.53 |
| RAD18 | sCCA1 | 722 | 22 | enet | 0.27 | 5.7e-23 | -2.46 | 0.0139 | 0.53 |
| ATP2A3 | sCCA1 | 494 | 10 | enet | 0.19 | 4.4e-16 | -2.47 | 0.013629 | 0.53 |
| KLHL20 | sCCA3 | 270 | 9 | enet | 0.041 | 0.00022 | -2.46 | 0.0139 | 0.53 |
| PPP2R5C | sCCA1 | 357 | 25 | enet | 0.022 | 0.005 | 2.5 | 0.01239 | 0.53 |
| OSBPL6 | sCCA1 | 424 | 13 | lasso | 0.44 | 3.7e-40 | 2.52 | 0.0118 | 0.53 |
| AP4E1 | sCCA2 | 477 | 12 | lasso | 0.15 | 1.5e-12 | 2.49 | 0.01273 | 0.53 |
| TNPO1 | sCCA2 | 356 | 1 | top1 | 0.082 | 2e-07 | -2.53 | 0.011549 | 0.53 |
| ITPKC | sCCA3 | 429 | 11 | enet | 0.48 | 1.6e-44 | -2.5 | 0.0125 | 0.53 |
| EPB41L4B | sCCA1 | 605 | 10 | lasso | 0.19 | 1.1e-15 | 2.48 | 0.01328 | 0.53 |
| APOL1 | sCCA2 | 499 | 1 | top1 | 0.067 | 3e-06 | 2.5 | 0.01251 | 0.53 |
| SLC25A17 | sCCA3 | 277 | 1 | top1 | 0.12 | 4.8e-10 | 2.52 | 0.01158 | 0.53 |
| CDKN3 | sCCA3 | 452 | 28 | enet | 0.059 | 1.1e-05 | -2.52 | 0.011793 | 0.53 |
| TIMM9 | sCCA3 | 467 | 1 | top1 | 0.02 | 0.0074 | 2.48 | 0.013074 | 0.53 |
| RAE1 | sCCA2 | 687 | 26 | enet | 0.032 | 0.00095 | 2.51 | 0.011972 | 0.53 |
| IDH3B | sCCA2 | 570 | 1 | top1 | 0.14 | 4.5e-12 | 2.52 | 0.011591 | 0.53 |
| SETD6 | sCCA3 | 475 | 7 | lasso | 0.28 | 7.6e-24 | -2.51 | 0.01201 | 0.53 |
| SYT17 | sCCA1 | 443 | 41 | enet | 0.3 | 4.3e-25 | 2.49 | 0.01271 | 0.53 |
| AKT2 | sCCA1 | 390 | 1 | top1 | 0.0016 | 0.23 | 2.52 | 0.011798 | 0.53 |
| TBCB | sCCA3 | 341 | 1 | top1 | 0.048 | 7.1e-05 | -2.48 | 0.0132 | 0.53 |
| PRKAG2 | sCCA2 | 432 | 1 | top1 | 0.11 | 2.1e-09 | -2.48 | 0.01307 | 0.53 |
| NEURL1 | sCCA2 | 367 | 13 | enet | 0.046 | 9.1e-05 | 2.46 | 0.014 | 0.53 |
| GOSR2 | sCCA2 | 286 | 1 | top1 | 0.11 | 3e-09 | -2.46 | 0.013736 | 0.53 |
| ASCC3 | sCCA1 | 306 | 34 | enet | 0.35 | 7.2e-31 | 2.53 | 0.01143 | 0.53 |
| GMDS | sCCA3 | 539 | 24 | enet | 0.0057 | 0.098 | 2.48 | 0.01322 | 0.53 |
| SSR3 | sCCA2 | 397 | 10 | lasso | 0.095 | 2.2e-08 | 2.51 | 0.012186 | 0.53 |
| LRRC42 | sCCA2 | 493 | 50 | enet | 0.049 | 6.2e-05 | 2.52 | 0.011895 | 0.53 |
| OSBPL9 | sCCA2 | 210 | 17 | enet | 0.074 | 8.3e-07 | -2.51 | 0.012018 | 0.53 |
| CTNNAL1 | sCCA2 | 622 | 8 | lasso | 0.34 | 1.8e-29 | -2.52 | 0.011677 | 0.53 |
| ZMYM2 | sCCA2 | 402 | 1 | top1 | 0.057 | 1.4e-05 | -2.52 | 0.01185 | 0.53 |
| GTDC1 | sCCA3 | 263 | 20 | enet | 0.0039 | 0.14 | 2.51 | 0.0122 | 0.53 |
| MRM2 | sCCA2 | 419 | 1 | top1 | 0.43 | 2.4e-39 | -2.49 | 0.012914 | 0.53 |
| PARD6B | sCCA2 | 492 | 20 | enet | 0.049 | 6.1e-05 | 2.47 | 0.013631 | 0.53 |
| SOX4 | sCCA3 | 486 | 24 | enet | 0.076 | 6.4e-07 | -2.48 | 0.01319 | 0.53 |
| RPL23 | sCCA2 | 363 | 21 | enet | 0.017 | 0.013 | -2.5 | 0.012585 | 0.53 |
| OPA3 | sCCA2 | 377 | 6 | lasso | 0.12 | 2.2e-10 | 2.5 | 0.012352 | 0.53 |
| RHOT1 | sCCA1 | 307 | 48 | enet | 0.31 | 3.5e-26 | -2.46 | 0.013823 | 0.53 |
| PRKCSH | sCCA1 | 392 | 6 | lasso | 0.24 | 7.2e-20 | -2.48 | 0.013161 | 0.53 |
| CCDC62 | sCCA2 | 301 | 23 | enet | 0.068 | 2.2e-06 | -2.48 | 0.013121 | 0.53 |
| PDE6B | sCCA2 | 367 | 4 | lasso | 0.042 | 0.00019 | 2.47 | 0.0137 | 0.53 |
| MBD2 | sCCA2 | 387 | 17 | lasso | 0.15 | 3.4e-12 | -2.52 | 0.0116 | 0.53 |
| SPIRE1 | sCCA1 | 375 | 19 | enet | 0.54 | 6.6e-53 | -2.47 | 0.01366 | 0.53 |
| HOOK1 | sCCA2 | 388 | 17 | enet | 0.18 | 5.9e-15 | -2.51 | 0.012008 | 0.53 |
| CDK4 | sCCA1 | 347 | 6 | lasso | 0.043 | 0.00016 | 2.47 | 0.0135 | 0.53 |
| RCBTB2 | sCCA2 | 382 | 1 | top1 | 0.18 | 8.5e-15 | 2.48 | 0.01327 | 0.53 |
| STAM | sCCA1 | 494 | 8 | enet | 0.23 | 2.4e-19 | -2.47 | 0.0133 | 0.53 |
| GGH | sCCA3 | 413 | 51 | enet | 0.67 | 3.7e-75 | 2.5 | 0.01259 | 0.53 |
| MTF2 | sCCA2 | 251 | 23 | enet | 0.024 | 0.0037 | 2.5 | 0.012461 | 0.53 |
| TMCO1 | sCCA3 | 547 | 27 | enet | 0.075 | 7.7e-07 | -2.48 | 0.0131 | 0.53 |
| DCAF1 | sCCA3 | 241 | 6 | lasso | 0.023 | 0.0049 | 2.51 | 0.012229 | 0.53 |
| PPIP5K2 | sCCA3 | 318 | 4 | lasso | 0.24 | 7.4e-20 | -2.52 | 0.011623 | 0.53 |
| PPIP5K2 | sCCA1 | 318 | 13 | lasso | 0.45 | 8.7e-42 | -2.48 | 0.013198 | 0.53 |
| PAM | sCCA2 | 305 | 22 | enet | 0.14 | 8.9e-12 | 2.48 | 0.013208 | 0.53 |
| PHIP | sCCA2 | 410 | 3 | lasso | 0.1 | 8.1e-09 | 2.51 | 0.01206 | 0.53 |
| GNA12 | sCCA1 | 563 | 6 | lasso | 0.46 | 5.7e-43 | 2.47 | 0.01369 | 0.53 |
| SAP18 | sCCA2 | 416 | 4 | lasso | 0.15 | 2.4e-12 | 2.48 | 0.01321 | 0.53 |
| ABCA10 | sCCA3 | 422 | 50 | enet | 0.038 | 0.00039 | 2.47 | 0.013505 | 0.53 |
| CCT8 | sCCA1 | 426 | 13 | lasso | 0.73 | 1.6e-89 | 2.46 | 0.013836 | 0.53 |
| PCDH1 | sCCA1 | 534 | 1 | top1 | 0.053 | 2.9e-05 | -2.46 | 0.013977 | 0.53 |
| CLDN12 | sCCA1 | 479 | 5 | lasso | 0.016 | 0.016 | 2.48 | 0.01323 | 0.53 |
| GATAD1 | sCCA2 | 307 | 18 | enet | 0.39 | 6.1e-35 | 2.52 | 0.011663 | 0.53 |
| DUSP2 | sCCA3 | 142 | 1 | top1 | 0.03 | 0.0015 | -2.49 | 0.0128 | 0.53 |
| RNF207 | sCCA1 | 450 | 9 | lasso | 0.38 | 9.9e-34 | -2.46 | 0.0139 | 0.53 |
| GDPD5 | sCCA3 | 439 | 19 | enet | 0.061 | 8.1e-06 | -2.48 | 0.01322 | 0.53 |
| PSMB4 | sCCA3 | 334 | 7 | lasso | 0.044 | 0.00014 | -2.47 | 0.0135 | 0.53 |
| ZER1 | sCCA2 | 284 | 1 | top1 | 0.05 | 4.5e-05 | 2.49 | 0.012724 | 0.53 |
| FDPS | sCCA2 | 272 | 18 | enet | 0.039 | 0.00029 | -2.53 | 0.01141 | 0.53 |
| ABCF3 | sCCA1 | 484 | 21 | enet | 0.47 | 2e-44 | -2.48 | 0.0132 | 0.53 |
| NEU3 | sCCA2 | 444 | 17 | lasso | 0.18 | 4.7e-15 | -2.52 | 0.011634 | 0.53 |
| PEA15 | sCCA2 | 513 | 24 | enet | 0.016 | 0.015 | 2.52 | 0.011601 | 0.53 |
| SGPP2 | sCCA3 | 457 | 12 | lasso | 0.083 | 1.9e-07 | -2.5 | 0.0124 | 0.53 |
| GPR155 | sCCA2 | 365 | 6 | lasso | 0.062 | 6e-06 | 2.47 | 0.0134 | 0.53 |
| RPN1 | sCCA1 | 355 | 7 | lasso | 0.42 | 9e-38 | 2.49 | 0.0128 | 0.53 |
| RPN1 | sCCA2 | 355 | 1 | top1 | 0.35 | 8e-31 | 2.47 | 0.013649 | 0.53 |
| C16orf46 | sCCA3 | 704 | 19 | enet | 0.023 | 0.0043 | -2.52 | 0.01185 | 0.53 |
| CENPV | sCCA3 | 282 | 31 | enet | 0.35 | 1.5e-30 | -2.51 | 0.011946 | 0.53 |
| ZSWIM1 | sCCA1 | 518 | 1 | top1 | 0.053 | 3.1e-05 | 2.52 | 0.01165 | 0.53 |
| MT1E | sCCA3 | 521 | 31 | enet | 0.28 | 1.3e-23 | -2.47 | 0.01338 | 0.53 |
| TMEM182 | sCCA1 | 418 | 17 | enet | 0.096 | 2e-08 | -2.46 | 0.0139 | 0.53 |
| RNF34 | sCCA1 | 393 | 1 | top1 | 0.067 | 2.8e-06 | -2.49 | 0.012762 | 0.53 |
| RSL1D1 | sCCA1 | 460 | 1 | top1 | 0.25 | 1.1e-20 | 2.5 | 0.01225 | 0.53 |
| SLC25A33 | sCCA1 | 351 | 13 | enet | 0.00038 | 0.29 | 2.51 | 0.0121 | 0.53 |
| ZNF540 | sCCA1 | 290 | 1 | top1 | 0.05 | 4.6e-05 | 2.46 | 0.013955 | 0.53 |
| ATPAF2 | sCCA2 | 349 | 15 | lasso | 0.41 | 1.9e-36 | 2.47 | 0.013651 | 0.53 |
| MYD88 | sCCA3 | 426 | 26 | enet | 0.0023 | 0.19 | 2.51 | 0.011935 | 0.53 |
| MYD88 | sCCA1 | 426 | 5 | lasso | 0.024 | 0.0036 | -2.5 | 0.0123 | 0.53 |
| COMMD1 | sCCA1 | 294 | 5 | lasso | 0.036 | 5e-04 | -2.51 | 0.0121 | 0.53 |
| RNF26 | sCCA1 | 412 | 1 | top1 | 0.68 | 4.7e-78 | -2.47 | 0.01364 | 0.53 |
| RNF26 | sCCA2 | 412 | 1 | top1 | 0.35 | 5.2e-30 | 2.47 | 0.01364 | 0.53 |
| C4orf32 | sCCA2 | 390 | 7 | lasso | 0.37 | 1.1e-32 | -2.51 | 0.01222 | 0.53 |
| C4orf32 | sCCA1 | 390 | 8 | lasso | 0.56 | 1.4e-55 | -2.5 | 0.01247 | 0.53 |
| GOLGA8A | sCCA1 | 418 | 1 | top1 | 0.26 | 7.1e-22 | 2.52 | 0.01164 | 0.53 |
| MYPOP | sCCA2 | 407 | 18 | enet | 0.085 | 1.3e-07 | -2.46 | 0.014005 | 0.53 |
| CRLF3 | sCCA1 | 277 | 38 | enet | 0.56 | 8.9e-56 | -2.47 | 0.013387 | 0.53 |
| SEC24C | sCCA2 | 245 | 1 | top1 | 0.059 | 1.1e-05 | 2.51 | 0.0121 | 0.53 |
| DNAJC28 | sCCA3 | 467 | 5 | lasso | 0.03 | 0.0014 | 2.5 | 0.012268 | 0.53 |
| MLF1 | sCCA1 | 390 | 31 | enet | 0.54 | 2.6e-53 | 2.51 | 0.012 | 0.53 |
| MLF1 | sCCA3 | 390 | 1 | top1 | 0.51 | 2e-48 | 2.49 | 0.0126 | 0.53 |
| DGKZP1 | sCCA1 | 509 | 17 | enet | 0.07 | 1.7e-06 | 2.49 | 0.01295 | 0.53 |
| SERTAD2 | sCCA2 | 453 | 17 | enet | 0.07 | 1.7e-06 | -2.5 | 0.0123 | 0.53 |
| CMTR2 | sCCA1 | 272 | 26 | enet | 0.23 | 2.8e-19 | 2.48 | 0.01301 | 0.53 |
| HIST3H2A | sCCA1 | 341 | 10 | lasso | 0.13 | 1e-10 | 2.46 | 0.0139 | 0.53 |
| BTBD6 | sCCA3 | 219 | 12 | enet | 0.14 | 7.7e-12 | 2.52 | 0.011861 | 0.53 |
| ARL17A | sCCA2 | 142 | 17 | lasso | 0.45 | 7.8e-42 | 2.46 | 0.013934 | 0.53 |
| C7orf61 | sCCA1 | 305 | 16 | enet | 0.03 | 0.0013 | -2.47 | 0.01334 | 0.53 |
| ANKRD46 | sCCA2 | 432 | 1 | top1 | 0.081 | 2.5e-07 | -2.52 | 0.011612 | 0.53 |
| MAPT | sCCA1 | 31 | 1 | top1 | 0.59 | 1e-60 | 2.48 | 0.013323 | 0.53 |
| MAPT | sCCA2 | 31 | 1 | top1 | 0.59 | 2.1e-60 | 2.48 | 0.013323 | 0.53 |
| MAPT | sCCA3 | 31 | 1 | top1 | 0.57 | 1.6e-57 | 2.48 | 0.013323 | 0.53 |
| ZNF383 | sCCA2 | 276 | 4 | lasso | 0.045 | 0.00012 | 2.49 | 0.012752 | 0.53 |
| ALKAL2 | sCCA2 | 390 | 1 | top1 | 0.1 | 7.5e-09 | -2.52 | 0.0118 | 0.53 |
| LINC00176 | sCCA3 | 339 | 43 | enet | 0.26 | 7.3e-22 | 2.47 | 0.01341 | 0.53 |
| ZNF569 | sCCA1 | 278 | 1 | top1 | 0.12 | 3.8e-10 | 2.48 | 0.013302 | 0.53 |
| PLXNB2 | sCCA1 | 396 | 34 | enet | 0.15 | 1.3e-12 | 2.51 | 0.01191 | 0.53 |
| MCMBP | sCCA2 | 499 | 18 | enet | 0.03 | 0.0013 | -2.51 | 0.0121 | 0.53 |
| C1orf228 | sCCA1 | 365 | 11 | lasso | 0.24 | 3.2e-20 | 2.46 | 0.014 | 0.53 |
| DDX39B | sCCA1 | 275 | 24 | enet | 0.16 | 3.1e-13 | 2.5 | 0.01225 | 0.53 |
| ITPRIPL1 | sCCA1 | 162 | 5 | lasso | 0.6 | 2.5e-62 | 2.51 | 0.0121 | 0.53 |
| ITPRIPL1 | sCCA3 | 162 | 5 | lasso | 0.59 | 1.1e-60 | -2.5 | 0.0123 | 0.53 |
| LINC02210 | sCCA1 | 92 | 1 | top1 | 0.92 | 1.5e-172 | -2.48 | 0.013323 | 0.53 |
| LINC02210 | sCCA2 | 92 | 1 | top1 | 0.88 | 7.8e-142 | -2.48 | 0.013323 | 0.53 |
| LINC02210 | sCCA3 | 92 | 1 | top1 | 0.92 | 2.3e-171 | -2.48 | 0.013323 | 0.53 |
| CTD-2270N23.1 | sCCA2 | 463 | 1 | top1 | 0.039 | 3e-04 | -2.48 | 0.01307 | 0.53 |
| KANSL1-AS1 | sCCA1 | 29 | 1 | top1 | 0.8 | 4.3e-107 | 2.48 | 0.013323 | 0.53 |
| KANSL1-AS1 | sCCA3 | 29 | 1 | top1 | 0.8 | 1.7e-107 | -2.48 | 0.013323 | 0.53 |
| LRRC37A4P | sCCA1 | 136 | 22 | enet | 0.92 | 3.5e-165 | 2.51 | 0.011905 | 0.53 |
| LRRC37A4P | sCCA3 | 136 | 24 | enet | 0.91 | 7.6e-160 | -2.51 | 0.01191 | 0.53 |
| LRRC37A4P | sCCA2 | 136 | 37 | enet | 0.87 | 8.2e-137 | 2.5 | 0.012369 | 0.53 |
| RP11-278A23.1 | sCCA1 | 493 | 11 | lasso | 0.54 | 1.5e-52 | 2.52 | 0.01187 | 0.53 |
| RP11-278A23.1 | sCCA3 | 493 | 10 | lasso | 0.53 | 1.3e-51 | 2.5 | 0.01257 | 0.53 |
| CTC-529I10.2 | sCCA3 | 281 | 1 | top1 | 0.067 | 2.9e-06 | -2.53 | 0.011358 | 0.53 |
| CTAGE7P | sCCA2 | 627 | 1 | top1 | 0.0014 | 0.23 | 2.47 | 0.0135 | 0.53 |
| CHCHD2P6 | sCCA1 | 486 | 6 | lasso | 0.049 | 6.2e-05 | 2.46 | 0.0139 | 0.53 |
| MTX1P1 | sCCA1 | 322 | 4 | lasso | 0.16 | 1.3e-13 | 2.48 | 0.0131 | 0.53 |
| LINC00888 | sCCA3 | 424 | 32 | enet | 0.058 | 1.2e-05 | -2.47 | 0.013598 | 0.53 |
| RP11-486O12.2 | sCCA2 | 375 | 16 | enet | 0.05 | 4.8e-05 | 2.46 | 0.013757 | 0.53 |
| ZNF564 | sCCA2 | 286 | 1 | top1 | 0.045 | 0.00011 | 2.49 | 0.012945 | 0.53 |
| RP11-549J18.1 | sCCA3 | 226 | 1 | top1 | 0.025 | 0.0034 | -2.48 | 0.013292 | 0.53 |
| RP11-341G23.4 | sCCA1 | 617 | 22 | enet | 0.75 | 3.4e-92 | -2.49 | 0.012909 | 0.53 |
| PRC1-AS1 | sCCA2 | 488 | 7 | lasso | 0.22 | 6.4e-18 | -2.5 | 0.01254 | 0.53 |
| LINC00622 | sCCA3 | 392 | 4 | lasso | 0.099 | 1.2e-08 | -2.46 | 0.014 | 0.53 |
| RP11-669E14.6 | sCCA1 | 29 | 1 | top1 | 0.14 | 1.2e-11 | 2.48 | 0.013323 | 0.53 |
| ROCK1P1 | sCCA2 | 311 | 16 | enet | 0.62 | 4.1e-65 | -2.47 | 0.0133 | 0.53 |
| MAPK8IP1P2 | sCCA1 | 110 | 1 | top1 | 0.86 | 1.7e-129 | -2.48 | 0.013323 | 0.53 |
| MAPK8IP1P2 | sCCA2 | 110 | 1 | top1 | 0.82 | 6.2e-116 | -2.48 | 0.013323 | 0.53 |
| MAPK8IP1P2 | sCCA3 | 110 | 1 | top1 | 0.84 | 1.1e-124 | 2.48 | 0.013323 | 0.53 |
| DND1P1 | sCCA3 | 118 | 12 | lasso | 0.82 | 1.3e-114 | -2.48 | 0.013289 | 0.53 |
| DND1P1 | sCCA1 | 118 | 12 | lasso | 0.85 | 7.2e-126 | -2.48 | 0.013323 | 0.53 |
| DND1P1 | sCCA2 | 118 | 12 | lasso | 0.74 | 6e-91 | -2.48 | 0.013323 | 0.53 |
| RP11-204L24.2 | sCCA3 | 354 | 27 | enet | 0.044 | 0.00012 | -2.5 | 0.0125 | 0.53 |
| RP11-466A19.1 | sCCA3 | 293 | 18 | enet | 0.074 | 8.9e-07 | -2.47 | 0.013453 | 0.53 |
| ZNF793-AS1 | sCCA2 | 275 | 1 | top1 | 0.15 | 2.9e-12 | 2.52 | 0.011582 | 0.53 |
| ZNF793-AS1 | sCCA1 | 275 | 1 | top1 | 0.34 | 5.8e-29 | -2.49 | 0.012762 | 0.53 |
| ZNF793-AS1 | sCCA3 | 275 | 1 | top1 | 0.082 | 2.3e-07 | 2.48 | 0.0133 | 0.53 |
| AC002398.13 | sCCA1 | 428 | 8 | lasso | 0.36 | 6.2e-31 | 2.51 | 0.011926 | 0.53 |
| RP11-464F9.20 | sCCA2 | 253 | 1 | top1 | 0.041 | 0.00022 | 2.51 | 0.0121 | 0.53 |
| CTC-490E21.10 | sCCA2 | 426 | 1 | top1 | 0.073 | 9.5e-07 | -2.51 | 0.012025 | 0.53 |
| XXbac-BPG299F13.17 | sCCA3 | 173 | 45 | enet | 0.62 | 1.2e-65 | -2.48 | 0.013225 | 0.53 |
| RP11-390E23.6 | sCCA3 | 349 | 1 | top1 | 0.085 | 1.3e-07 | 2.51 | 0.012111 | 0.53 |
| RP5-1159O4.2 | sCCA2 | 713 | 25 | enet | 0.18 | 3.2e-15 | -2.51 | 0.012082 | 0.53 |
| RP11-449P15.2 | sCCA1 | 329 | 3 | lasso | 0.4 | 2.8e-35 | -2.5 | 0.01247 | 0.53 |
| RP11-138A9.2 | sCCA2 | 407 | 28 | enet | 0.035 | 0.00063 | -2.46 | 0.013953 | 0.53 |
| SOCS7 | sCCA3 | 184 | 15 | enet | 0.077 | 5e-07 | -2.48 | 0.01297 | 0.53 |
| RP11-455O6.9 | sCCA3 | 381 | 1 | top1 | 0.061 | 7.4e-06 | 2.48 | 0.012979 | 0.53 |
| RP11-425M5.7 | sCCA2 | 384 | 1 | top1 | 0.019 | 0.0085 | 2.51 | 0.012117 | 0.53 |
| AD001527.4 | sCCA1 | 341 | 6 | lasso | 0.57 | 2.7e-57 | -2.49 | 0.01276 | 0.53 |
| RP13-638C3.5 | sCCA3 | 398 | 26 | enet | 0.064 | 4.9e-06 | 2.52 | 0.011831 | 0.53 |
| RP11-707O23.1 | sCCA1 | 113 | 11 | lasso | 0.78 | 7.9e-103 | -2.48 | 0.013323 | 0.53 |
| RP11-707O23.1 | sCCA3 | 113 | 11 | lasso | 0.76 | 5.8e-95 | -2.48 | 0.013323 | 0.53 |
| MAD1L1 | sCCA2 | 416 | 1 | top1 | 0.092 | 4e-08 | 2.42 | 0.015665 | 0.54 |
| PHTF2 | sCCA3 | 517 | 1 | top1 | 0.076 | 5.6e-07 | 2.43 | 0.015251 | 0.54 |
| AQR | sCCA3 | 399 | 25 | enet | 0.038 | 0.00036 | -2.43 | 0.01511 | 0.54 |
| ACAA1 | sCCA2 | 425 | 1 | top1 | 0.0094 | 0.05 | 2.46 | 0.014075 | 0.54 |
| INTS13 | sCCA3 | 516 | 22 | enet | 0.042 | 0.00017 | 2.43 | 0.0153 | 0.54 |
| SUGP2 | sCCA3 | 331 | 13 | lasso | 0.18 | 1.2e-14 | 2.43 | 0.0152 | 0.54 |
| DHX29 | sCCA1 | 386 | 1 | top1 | 0.12 | 2.5e-10 | 2.42 | 0.015658 | 0.54 |
| DHX29 | sCCA3 | 386 | 1 | top1 | 0.043 | 0.00016 | -2.42 | 0.015658 | 0.54 |
| TP53BP1 | sCCA3 | 266 | 25 | enet | 0.18 | 7.4e-15 | -2.42 | 0.0155 | 0.54 |
| PICALM | sCCA3 | 484 | 15 | enet | 0.17 | 4.1e-14 | -2.45 | 0.01432 | 0.54 |
| USP33 | sCCA3 | 427 | 16 | enet | 0.065 | 3.6e-06 | -2.43 | 0.0151 | 0.54 |
| BCKDHB | sCCA1 | 465 | 15 | lasso | 0.16 | 2.9e-13 | -2.45 | 0.01447 | 0.54 |
| PDS5B | sCCA1 | 498 | 5 | lasso | 0.1 | 4.5e-09 | 2.42 | 0.01536 | 0.54 |
| PSMD5 | sCCA1 | 371 | 16 | enet | 0.51 | 6.7e-49 | 2.46 | 0.01405 | 0.54 |
| PSMD5 | sCCA3 | 371 | 16 | enet | 0.51 | 6.7e-49 | -2.46 | 0.01405 | 0.54 |
| EFHC1 | sCCA2 | 531 | 22 | enet | 0.086 | 1e-07 | -2.44 | 0.014605 | 0.54 |
| SMARCB1 | sCCA3 | 464 | 12 | lasso | 0.46 | 4.5e-42 | -2.44 | 0.0147 | 0.54 |
| PDGFB | sCCA3 | 402 | 34 | enet | 0.021 | 0.0066 | 2.41 | 0.01578 | 0.54 |
| APOL1 | sCCA1 | 499 | 20 | enet | 0.11 | 1.6e-09 | 2.45 | 0.01428 | 0.54 |
| MMP9 | sCCA2 | 498 | 1 | top1 | 0.071 | 1.4e-06 | -2.43 | 0.015286 | 0.54 |
| TRIB3 | sCCA2 | 468 | 1 | top1 | 0.47 | 8.6e-44 | -2.44 | 0.014743 | 0.54 |
| MPG | sCCA1 | 242 | 7 | lasso | 0.09 | 5.5e-08 | -2.45 | 0.01444 | 0.54 |
| LMF1 | sCCA2 | 441 | 9 | lasso | 0.4 | 1.6e-35 | 2.45 | 0.01411 | 0.54 |
| ELL | sCCA3 | 376 | 23 | enet | 0.02 | 0.008 | -2.41 | 0.0161 | 0.54 |
| BET1 | sCCA2 | 395 | 4 | lasso | 0.084 | 1.6e-07 | -2.45 | 0.014462 | 0.54 |
| ASCC3 | sCCA3 | 306 | 37 | enet | 0.35 | 1.4e-30 | -2.41 | 0.01584 | 0.54 |
| FAM162A | sCCA2 | 472 | 7 | lasso | 0.098 | 1.5e-08 | -2.41 | 0.015996 | 0.54 |
| KPNA1 | sCCA2 | 494 | 1 | top1 | 0.068 | 2.2e-06 | -2.44 | 0.014646 | 0.54 |
| KPNA1 | sCCA3 | 494 | 1 | top1 | 0.11 | 1.5e-09 | 2.44 | 0.014646 | 0.54 |
| EIF4G1 | sCCA2 | 522 | 5 | lasso | 0.054 | 2.5e-05 | 2.41 | 0.015778 | 0.54 |
| AGMAT | sCCA2 | 496 | 8 | lasso | 0.13 | 2.2e-11 | -2.44 | 0.014771 | 0.54 |
| PLEKHM2 | sCCA3 | 482 | 1 | top1 | 0.086 | 1e-07 | -2.43 | 0.0152 | 0.54 |
| STMN1 | sCCA2 | 423 | 27 | enet | 0.057 | 1.5e-05 | -2.45 | 0.014415 | 0.54 |
| LDAH | sCCA2 | 530 | 1 | top1 | 0.48 | 1.4e-44 | 2.43 | 0.015 | 0.54 |
| CTNNAL1 | sCCA3 | 622 | 10 | lasso | 0.58 | 1.5e-59 | 2.43 | 0.01522 | 0.54 |
| RBM18 | sCCA1 | 541 | 6 | lasso | 0.28 | 8.1e-24 | -2.43 | 0.01529 | 0.54 |
| MXI1 | sCCA3 | 372 | 34 | enet | 0.023 | 0.0042 | -2.41 | 0.0161 | 0.54 |
| MRM2 | sCCA3 | 419 | 10 | lasso | 0.51 | 5.3e-49 | -2.43 | 0.015279 | 0.54 |
| MRM2 | sCCA1 | 419 | 9 | lasso | 0.52 | 3.8e-50 | -2.42 | 0.01573 | 0.54 |
| PLA2G12A | sCCA3 | 397 | 3 | lasso | 0.22 | 1.8e-18 | 2.41 | 0.01574 | 0.54 |
| NCOA3 | sCCA3 | 451 | 1 | top1 | 0.065 | 4.2e-06 | -2.45 | 0.01437 | 0.54 |
| PPP4R1L | sCCA2 | 652 | 30 | enet | 0.066 | 3.3e-06 | 2.43 | 0.014957 | 0.54 |
| MSTO1 | sCCA2 | 219 | 38 | enet | 0.1 | 8.3e-09 | -2.45 | 0.014353 | 0.54 |
| OPA3 | sCCA1 | 377 | 32 | enet | 0.2 | 1.1e-16 | 2.44 | 0.014585 | 0.54 |
| PRDX5 | sCCA2 | 359 | 20 | enet | 0.11 | 1.1e-09 | -2.43 | 0.014931 | 0.54 |
| BECN1 | sCCA3 | 233 | 14 | lasso | 0.17 | 4.4e-14 | -2.41 | 0.015932 | 0.54 |
| CALU | sCCA3 | 352 | 23 | enet | 0.036 | 0.00053 | -2.42 | 0.015454 | 0.54 |
| CEP85 | sCCA2 | 427 | 30 | enet | 0.26 | 8.2e-22 | -2.41 | 0.015952 | 0.54 |
| LANCL2 | sCCA1 | 406 | 6 | lasso | 0.2 | 1.3e-16 | -2.44 | 0.01454 | 0.54 |
| TSPAN31 | sCCA3 | 342 | 1 | top1 | 0.065 | 4.1e-06 | -2.44 | 0.0149 | 0.54 |
| OS9 | sCCA1 | 349 | 1 | top1 | 0.25 | 1.2e-20 | 2.44 | 0.014657 | 0.54 |
| OS9 | sCCA2 | 349 | 1 | top1 | 0.16 | 3.4e-13 | 2.44 | 0.014657 | 0.54 |
| PLXNC1 | sCCA2 | 621 | 23 | enet | 0.03 | 0.0013 | 2.44 | 0.014715 | 0.54 |
| RCBTB1 | sCCA1 | 380 | 42 | enet | 0.51 | 6.5e-49 | -2.41 | 0.01614 | 0.54 |
| GGH | sCCA1 | 413 | 52 | enet | 0.67 | 5.3e-76 | -2.45 | 0.0141 | 0.54 |
| KYAT3 | sCCA1 | 352 | 37 | enet | 0.67 | 2.4e-74 | 2.44 | 0.0146 | 0.54 |
| RNF185 | sCCA1 | 364 | 1 | top1 | 0.26 | 9.7e-22 | -2.45 | 0.01435 | 0.54 |
| 9-Mar | sCCA1 | 347 | 9 | lasso | 0.12 | 5.2e-10 | 2.43 | 0.015053 | 0.54 |
| ARSG | sCCA3 | 365 | 9 | lasso | 0.094 | 2.9e-08 | 2.45 | 0.014097 | 0.54 |
| PGD | sCCA2 | 373 | 37 | enet | 0.13 | 1.1e-10 | 2.45 | 0.014295 | 0.54 |
| SNAPIN | sCCA2 | 401 | 1 | top1 | 0.099 | 1.2e-08 | -2.41 | 0.015778 | 0.54 |
| SCRN3 | sCCA1 | 377 | 39 | enet | 0.3 | 4.9e-25 | 2.41 | 0.016 | 0.54 |
| FAM193B | sCCA3 | 242 | 4 | lasso | 0.2 | 1.1e-16 | 2.41 | 0.015781 | 0.54 |
| FBXO25 | sCCA1 | 406 | 24 | enet | 0.28 | 3e-23 | 2.44 | 0.0145 | 0.54 |
| TOMM70 | sCCA1 | 393 | 8 | lasso | 0.38 | 1.1e-33 | -2.44 | 0.0147 | 0.54 |
| SLA | sCCA1 | 713 | 1 | top1 | 0.014 | 0.024 | 2.41 | 0.0158 | 0.54 |
| GATAD1 | sCCA3 | 307 | 16 | lasso | 0.49 | 6.1e-46 | -2.41 | 0.016099 | 0.54 |
| ABCF3 | sCCA2 | 484 | 9 | enet | 0.48 | 1.2e-45 | 2.43 | 0.014908 | 0.54 |
| S100A9 | sCCA3 | 466 | 1 | top1 | 0.012 | 0.029 | 2.43 | 0.0152 | 0.54 |
| S100A9 | sCCA1 | 466 | 1 | top1 | 0.1 | 5.3e-09 | -2.41 | 0.0158 | 0.54 |
| TMEM79 | sCCA3 | 391 | 28 | enet | 0.018 | 0.011 | -2.43 | 0.015 | 0.54 |
| STT3B | sCCA1 | 481 | 30 | enet | 0.14 | 7.1e-12 | -2.45 | 0.0142 | 0.54 |
| RPN1 | sCCA3 | 355 | 7 | lasso | 0.41 | 2.5e-37 | 2.44 | 0.014494 | 0.54 |
| TTC8 | sCCA3 | 438 | 3 | lasso | 0.048 | 6.5e-05 | -2.43 | 0.015085 | 0.54 |
| ZNF503 | sCCA2 | 368 | 36 | enet | 0.077 | 5.2e-07 | 2.41 | 0.0158 | 0.54 |
| CENPV | sCCA2 | 282 | 19 | enet | 0.45 | 2.5e-41 | -2.45 | 0.014396 | 0.54 |
| FAM111A | sCCA3 | 339 | 8 | lasso | 0.16 | 9.5e-14 | -2.41 | 0.01597 | 0.54 |
| GPT | sCCA3 | 231 | 22 | enet | 0.052 | 3.3e-05 | 2.41 | 0.01611 | 0.54 |
| TMUB2 | sCCA2 | 351 | 20 | enet | 0.092 | 3.6e-08 | -2.45 | 0.014225 | 0.54 |
| THBS3 | sCCA1 | 333 | 36 | enet | 0.33 | 2.1e-28 | -2.45 | 0.0145 | 0.54 |
| P2RY12 | sCCA1 | 501 | 44 | enet | 0.58 | 6.8e-59 | 2.44 | 0.0146 | 0.54 |
| RNF34 | sCCA3 | 393 | 5 | lasso | 0.00066 | 0.27 | 2.45 | 0.0144 | 0.54 |
| POLH | sCCA2 | 434 | 29 | enet | 0.034 | 0.00065 | -2.42 | 0.015395 | 0.54 |
| MTX1 | sCCA1 | 333 | 2 | lasso | 0.096 | 2.1e-08 | -2.45 | 0.0142 | 0.54 |
| DCTN2 | sCCA3 | 336 | 1 | top1 | 0.022 | 0.0052 | -2.41 | 0.0159 | 0.54 |
| UCP3 | sCCA2 | 378 | 1 | top1 | 0.021 | 0.0067 | 2.41 | 0.0158 | 0.54 |
| ZNF169 | sCCA1 | 426 | 26 | enet | 0.019 | 0.0085 | 2.45 | 0.01439 | 0.54 |
| MYPOP | sCCA1 | 407 | 29 | enet | 0.074 | 8.7e-07 | 2.42 | 0.015562 | 0.54 |
| FAM210A | sCCA1 | 472 | 4 | lasso | 0.048 | 6.5e-05 | -2.45 | 0.01446 | 0.54 |
| RAP2B | sCCA3 | 367 | 15 | enet | 0.041 | 0.00024 | 2.45 | 0.014162 | 0.54 |
| NDUFA6 | sCCA3 | 340 | 63 | enet | 0.65 | 5.4e-72 | 2.43 | 0.01498 | 0.54 |
| BRF1 | sCCA2 | 193 | 1 | top1 | 0.18 | 2.2e-15 | -2.41 | 0.0159 | 0.54 |
| MARC1 | sCCA1 | 408 | 8 | lasso | 0.23 | 3.8e-19 | -2.45 | 0.0142 | 0.54 |
| BLOC1S4 | sCCA1 | 553 | 14 | enet | 0.028 | 0.002 | -2.42 | 0.01549 | 0.54 |
| ZNF140 | sCCA1 | 179 | 17 | enet | 0.11 | 1.7e-09 | -2.41 | 0.016107 | 0.54 |
| PGAP1 | sCCA2 | 349 | 4 | lasso | 0.05 | 4.7e-05 | -2.44 | 0.0145 | 0.54 |
| PAPSS2 | sCCA3 | 323 | 35 | enet | 0.25 | 4.4e-21 | -2.44 | 0.0146 | 0.54 |
| ITPRIPL1 | sCCA2 | 162 | 8 | lasso | 0.37 | 1e-32 | 2.45 | 0.0142 | 0.54 |
| SMIM5 | sCCA2 | 335 | 25 | enet | 0.031 | 0.0011 | -2.43 | 0.014938 | 0.54 |
| IGHG3 | sCCA1 | 43 | 1 | top1 | 0.046 | 9.6e-05 | 2.43 | 0.01501 | 0.54 |
| RP11-465B22.3 | sCCA2 | 205 | 19 | enet | 0.058 | 1.2e-05 | -2.42 | 0.015716 | 0.54 |
| LINC01422 | sCCA1 | 865 | 14 | lasso | 0.48 | 1.1e-45 | 2.42 | 0.01566 | 0.54 |
| LINC01422 | sCCA3 | 865 | 11 | lasso | 0.47 | 4.7e-44 | -2.41 | 0.01614 | 0.54 |
| RUSC1-AS1 | sCCA2 | 260 | 1 | top1 | 0.21 | 1.9e-17 | -2.43 | 0.015297 | 0.54 |
| RUSC1-AS1 | sCCA3 | 260 | 1 | top1 | 0.14 | 1e-11 | 2.43 | 0.0153 | 0.54 |
| ZNF192P1 | sCCA2 | 464 | 31 | enet | 0.21 | 9.1e-18 | -2.42 | 0.01547 | 0.54 |
| ARL17B | sCCA2 | 60 | 19 | enet | 0.34 | 2.3e-29 | -2.45 | 0.014294 | 0.54 |
| AC090587.4 | sCCA1 | 348 | 7 | lasso | 0.12 | 1.6e-10 | 2.45 | 0.01429 | 0.54 |
| DDX50P1 | sCCA2 | 281 | 7 | lasso | 0.045 | 0.00011 | 2.43 | 0.015 | 0.54 |
| HLA-W | sCCA3 | 39 | 28 | enet | 0.12 | 3.7e-10 | -2.45 | 0.014465 | 0.54 |
| TMEM189 | sCCA2 | 436 | 20 | enet | 0.098 | 1.5e-08 | 2.42 | 0.015343 | 0.54 |
| TMEM35B | sCCA1 | 307 | 6 | lasso | -0.0013 | 0.44 | 2.44 | 0.0146 | 0.54 |
| AP000487.5 | sCCA2 | 424 | 5 | lasso | 0.11 | 3.4e-09 | -2.41 | 0.016053 | 0.54 |
| CTD-2516F10.2 | sCCA2 | 660 | 13 | lasso | 0.52 | 4.1e-51 | -2.41 | 0.015851 | 0.54 |
| RP11-843P14.1 | sCCA1 | 311 | 8 | lasso | 0.11 | 1.6e-09 | -2.42 | 0.01569 | 0.54 |
| RP13-317D12.3 | sCCA3 | 395 | 4 | lasso | 0.056 | 1.7e-05 | -2.43 | 0.01506 | 0.54 |
| ZNF10 | sCCA1 | 163 | 10 | enet | 0.44 | 2.3e-40 | 2.44 | 0.014712 | 0.54 |
| RP11-73E17.2 | sCCA3 | 360 | 7 | lasso | 0.065 | 4e-06 | 2.42 | 0.015559 | 0.54 |
| SH3RF3-AS1 | sCCA3 | 384 | 1 | top1 | 0.037 | 0.00043 | 2.41 | 0.0157 | 0.54 |
| ROCK1P1 | sCCA1 | 311 | 20 | enet | 0.67 | 3.4e-74 | 2.43 | 0.01507 | 0.54 |
| RP11-96D1.10 | sCCA1 | 302 | 10 | enet | 0.04 | 0.00025 | 2.45 | 0.01422 | 0.54 |
| RP11-466A19.1 | sCCA2 | 293 | 1 | top1 | 0.065 | 4e-06 | 2.42 | 0.015369 | 0.54 |
| RP11-686D22.4 | sCCA3 | 368 | 8 | lasso | 0.35 | 4.3e-30 | 2.41 | 0.015957 | 0.54 |
| MKNK1-AS1 | sCCA1 | 350 | 1 | top1 | 0.17 | 7.3e-14 | 2.42 | 0.0155 | 0.54 |
| RP11-250B2.6 | sCCA1 | 439 | 24 | enet | 0.41 | 2.9e-36 | 2.41 | 0.01603 | 0.54 |
| RP11-563N4.1 | sCCA3 | 270 | 18 | enet | 0.22 | 5.6e-18 | -2.42 | 0.0155 | 0.54 |
| RP11-493E12.2 | sCCA1 | 303 | 22 | enet | 0.33 | 8.9e-29 | 2.41 | 0.0159 | 0.54 |
| RP11-582E3.6 | sCCA2 | 523 | 41 | enet | 0.44 | 3.7e-40 | 2.44 | 0.014776 | 0.54 |
| SNORA61 | sCCA2 | 267 | 32 | enet | 0.1 | 6.8e-09 | -2.43 | 0.015043 | 0.54 |
| MRM1 | sCCA2 | 321 | 1 | top1 | 0.12 | 3e-10 | -2.43 | 0.014973 | 0.54 |
| AD001527.4 | sCCA2 | 341 | 47 | enet | 0.52 | 1.3e-50 | -2.42 | 0.015383 | 0.54 |
| RP11-122G18.12 | sCCA3 | 393 | 7 | lasso | 0.32 | 1.3e-27 | 2.41 | 0.0159 | 0.54 |
| PDK4 | sCCA2 | 505 | 1 | top1 | 0.077 | 5.1e-07 | -2.37 | 0.017717 | 0.55 |
| RHBDD2 | sCCA2 | 294 | 1 | top1 | 0.049 | 5.5e-05 | -2.36 | 0.018521 | 0.55 |
| IFRD1 | sCCA2 | 403 | 7 | lasso | 0.4 | 4e-36 | -2.35 | 0.018544 | 0.55 |
| ETV7 | sCCA3 | 490 | 4 | lasso | 0.17 | 5.4e-14 | -2.38 | 0.0174 | 0.55 |
| ETV7 | sCCA2 | 490 | 5 | lasso | 0.16 | 9.3e-14 | 2.38 | 0.017519 | 0.55 |
| ISOC1 | sCCA1 | 420 | 16 | lasso | 0.16 | 1.3e-13 | 2.37 | 0.0178 | 0.55 |
| PLPP1 | sCCA2 | 330 | 15 | enet | 0.032 | 0.0011 | 2.34 | 0.019162 | 0.55 |
| TP53BP1 | sCCA1 | 266 | 1 | top1 | 0.32 | 6.7e-28 | -2.35 | 0.01887 | 0.55 |
| ROGDI | sCCA1 | 383 | 1 | top1 | 0.39 | 2.4e-34 | -2.35 | 0.01886 | 0.55 |
| MEF2A | sCCA1 | 513 | 12 | enet | 0.14 | 1.2e-11 | -2.37 | 0.01772 | 0.55 |
| LRP6 | sCCA3 | 549 | 3 | lasso | 0.21 | 1.1e-17 | 2.36 | 0.0183 | 0.55 |
| PICALM | sCCA1 | 484 | 14 | lasso | 0.32 | 1.1e-27 | 2.39 | 0.016804 | 0.55 |
| UBE2K | sCCA2 | 363 | 19 | enet | 0.08 | 3.1e-07 | -2.34 | 0.0192 | 0.55 |
| MKNK1 | sCCA1 | 351 | 6 | lasso | 0.43 | 5.3e-39 | -2.34 | 0.0191 | 0.55 |
| KDM2B | sCCA2 | 395 | 1 | top1 | 0.15 | 1.8e-12 | -2.4 | 0.016388 | 0.55 |
| KDM2B | sCCA1 | 395 | 39 | enet | 0.32 | 3.1e-27 | -2.4 | 0.016516 | 0.55 |
| KDM2B | sCCA3 | 395 | 15 | lasso | 0.21 | 1.3e-17 | 2.39 | 0.017 | 0.55 |
| OAS1 | sCCA2 | 416 | 16 | enet | 0.0032 | 0.16 | -2.39 | 0.016938 | 0.55 |
| PALMD | sCCA3 | 498 | 6 | lasso | 0.029 | 0.0018 | -2.39 | 0.0167 | 0.55 |
| ST13 | sCCA1 | 275 | 9 | enet | 0.13 | 7.6e-11 | 2.38 | 0.01751 | 0.55 |
| TELO2 | sCCA1 | 491 | 58 | enet | 0.68 | 3e-77 | 2.36 | 0.01806 | 0.55 |
| KIAA0391 | sCCA3 | 368 | 27 | enet | 0.27 | 2e-22 | 2.37 | 0.017864 | 0.55 |
| MMP9 | sCCA1 | 498 | 5 | lasso | 0.097 | 1.6e-08 | 2.37 | 0.01793 | 0.55 |
| PSMD7 | sCCA2 | 365 | 22 | enet | 0.025 | 0.0031 | -2.39 | 0.01692 | 0.55 |
| PPP2R1A | sCCA3 | 547 | 1 | top1 | 0.016 | 0.014 | -2.37 | 0.0177 | 0.55 |
| WASL | sCCA1 | 349 | 1 | top1 | 0.13 | 8.9e-11 | -2.38 | 0.01716 | 0.55 |
| RHEB | sCCA3 | 552 | 1 | top1 | 0.025 | 0.0031 | 2.34 | 0.019052 | 0.55 |
| RAPGEF1 | sCCA2 | 460 | 1 | top1 | 0.12 | 1.5e-10 | -2.38 | 0.017468 | 0.55 |
| ERLIN1 | sCCA1 | 392 | 31 | enet | 0.088 | 7.3e-08 | -2.38 | 0.0175 | 0.55 |
| WNT3 | sCCA3 | 255 | 6 | lasso | 0.44 | 2.1e-40 | 2.35 | 0.019011 | 0.55 |
| RANGRF | sCCA2 | 446 | 1 | top1 | 0.039 | 0.00028 | 2.39 | 0.016889 | 0.55 |
| USP46 | sCCA2 | 293 | 1 | top1 | 0.049 | 5.4e-05 | -2.37 | 0.01765 | 0.55 |
| PPP6R3 | sCCA1 | 358 | 7 | lasso | 0.14 | 4.6e-12 | 2.4 | 0.016218 | 0.55 |
| PSMD9 | sCCA2 | 346 | 1 | top1 | 0.11 | 1.1e-09 | 2.35 | 0.018905 | 0.55 |
| LTA4H | sCCA1 | 554 | 5 | lasso | 0.17 | 2.3e-14 | -2.38 | 0.017286 | 0.55 |
| ELK3 | sCCA2 | 529 | 18 | enet | 0.079 | 3.4e-07 | -2.38 | 0.017278 | 0.55 |
| MANSC1 | sCCA3 | 537 | 4 | lasso | 0.13 | 5.4e-11 | 2.4 | 0.0163 | 0.55 |
| OAS3 | sCCA1 | 421 | 22 | enet | 0.37 | 2.6e-32 | -2.39 | 0.016944 | 0.55 |
| OAS3 | sCCA2 | 421 | 1 | top1 | 0.011 | 0.039 | -2.38 | 0.01711 | 0.55 |
| B3GAT2 | sCCA2 | 415 | 17 | enet | 0.3 | 5.8e-26 | 2.35 | 0.018725 | 0.55 |
| MEF2D | sCCA1 | 430 | 1 | top1 | 0.18 | 5.5e-15 | 2.38 | 0.0173 | 0.55 |
| DARS2 | sCCA1 | 275 | 1 | top1 | 0.42 | 2.2e-38 | 2.39 | 0.017 | 0.55 |
| DARS2 | sCCA3 | 275 | 1 | top1 | 0.2 | 1.1e-16 | -2.39 | 0.017 | 0.55 |
| NENF | sCCA2 | 472 | 29 | enet | 0.034 | 0.00077 | 2.4 | 0.016264 | 0.55 |
| ESYT2 | sCCA1 | 407 | 54 | enet | 0.41 | 4.8e-37 | -2.36 | 0.01808 | 0.55 |
| SPCS2 | sCCA1 | 455 | 32 | enet | 0.063 | 5.1e-06 | 2.35 | 0.018752 | 0.55 |
| SLC17A5 | sCCA1 | 382 | 46 | enet | 0.34 | 2e-29 | 2.36 | 0.01827 | 0.55 |
| SLC17A5 | sCCA3 | 382 | 47 | enet | 0.079 | 3.6e-07 | -2.36 | 0.01847 | 0.55 |
| WBP4 | sCCA3 | 305 | 12 | enet | 0.15 | 2.9e-12 | 2.36 | 0.0182 | 0.55 |
| IFT81 | sCCA3 | 261 | 12 | enet | 0.21 | 1.1e-17 | 2.37 | 0.0178 | 0.55 |
| SPRYD7 | sCCA2 | 403 | 6 | lasso | 0.071 | 1.5e-06 | 2.36 | 0.01827 | 0.55 |
| BATF3 | sCCA2 | 434 | 28 | enet | 0.047 | 8.4e-05 | -2.36 | 0.018115 | 0.55 |
| PMS2P5 | sCCA2 | 97 | 24 | enet | 0.52 | 1.5e-50 | 2.38 | 0.017526 | 0.55 |
| RUNX2 | sCCA2 | 354 | 1 | top1 | 0.013 | 0.028 | 2.37 | 0.017884 | 0.55 |
| ZNF780B | sCCA2 | 392 | 28 | enet | 0.031 | 0.0012 | -2.35 | 0.018782 | 0.55 |
| LRFN1 | sCCA3 | 440 | 1 | top1 | 0.067 | 2.6e-06 | 2.38 | 0.0175 | 0.55 |
| GFPT2 | sCCA1 | 424 | 26 | enet | 0.037 | 4e-04 | -2.38 | 0.017217 | 0.55 |
| CTNNBL1 | sCCA3 | 402 | 8 | lasso | 0.13 | 2.1e-11 | -2.36 | 0.01828 | 0.55 |
| GPALPP1 | sCCA1 | 348 | 1 | top1 | 0.025 | 0.0031 | -2.38 | 0.01727 | 0.55 |
| FLNB | sCCA3 | 400 | 57 | enet | 0.23 | 3.3e-19 | 2.4 | 0.016509 | 0.55 |
| LRRC8A | sCCA1 | 320 | 1 | top1 | 0.061 | 7.3e-06 | -2.38 | 0.01738 | 0.55 |
| ALDOB | sCCA2 | 548 | 14 | lasso | 0.17 | 2.8e-14 | 2.38 | 0.017325 | 0.55 |
| KIF13A | sCCA2 | 456 | 28 | enet | 0.076 | 5.9e-07 | -2.35 | 0.01886 | 0.55 |
| GGH | sCCA2 | 413 | 21 | lasso | 0.49 | 9.8e-47 | -2.37 | 0.017729 | 0.55 |
| KYAT3 | sCCA2 | 352 | 23 | enet | 0.54 | 6.3e-53 | 2.38 | 0.017278 | 0.55 |
| ARL3 | sCCA3 | 365 | 6 | lasso | 0.22 | 3.7e-18 | -2.38 | 0.0171 | 0.55 |
| ANXA7 | sCCA2 | 232 | 8 | lasso | 0.033 | 0.00078 | -2.39 | 0.017 | 0.55 |
| CLSTN3 | sCCA2 | 74 | 4 | lasso | 0.012 | 0.029 | -2.35 | 0.018828 | 0.55 |
| MVB12A | sCCA1 | 441 | 21 | enet | 0.47 | 2.7e-43 | 2.38 | 0.017087 | 0.55 |
| ZNF687 | sCCA3 | 311 | 34 | enet | 0.011 | 0.039 | 2.36 | 0.0183 | 0.55 |
| DUSP10 | sCCA1 | 474 | 3 | lasso | 0.13 | 5.7e-11 | -2.39 | 0.0166 | 0.55 |
| IQSEC1 | sCCA2 | 490 | 16 | enet | 0.097 | 1.6e-08 | 2.39 | 0.016698 | 0.55 |
| FAM105A | sCCA3 | 494 | 23 | enet | 0.035 | 0.00061 | 2.36 | 0.018078 | 0.55 |
| GIN1 | sCCA1 | 318 | 7 | lasso | 0.31 | 2.2e-26 | 2.36 | 0.018249 | 0.55 |
| PPIP5K2 | sCCA2 | 318 | 18 | enet | 0.23 | 3e-19 | 2.38 | 0.017356 | 0.55 |
| PAM | sCCA1 | 305 | 37 | enet | 0.43 | 5.3e-39 | 2.38 | 0.017165 | 0.55 |
| FAM193B | sCCA1 | 242 | 1 | top1 | 0.22 | 2.2e-18 | 2.39 | 0.01669 | 0.55 |
| FAM193B | sCCA2 | 242 | 1 | top1 | 0.08 | 3.1e-07 | 2.39 | 0.01669 | 0.55 |
| ZNF92 | sCCA1 | 160 | 5 | lasso | 0.27 | 4.4e-23 | -2.35 | 0.01862 | 0.55 |
| CSGALNACT1 | sCCA3 | 673 | 46 | enet | 0.45 | 5.3e-41 | 2.38 | 0.01728 | 0.55 |
| PTPRJ | sCCA1 | 237 | 1 | top1 | 0.27 | 4.7e-23 | -2.39 | 0.016871 | 0.55 |
| PTPRJ | sCCA2 | 237 | 1 | top1 | 0.16 | 4e-13 | -2.37 | 0.017568 | 0.55 |
| TRIM11 | sCCA2 | 338 | 8 | lasso | 0.092 | 3.6e-08 | 2.37 | 0.017599 | 0.55 |
| DPY19L4 | sCCA3 | 444 | 31 | enet | 0.13 | 7.7e-11 | -2.38 | 0.01755 | 0.55 |
| N6AMT1 | sCCA3 | 470 | 21 | enet | 0.38 | 5.1e-34 | 2.39 | 0.016683 | 0.55 |
| UBE2L6 | sCCA2 | 377 | 28 | enet | 0.038 | 0.00035 | -2.35 | 0.018807 | 0.55 |
| SAMD8 | sCCA1 | 311 | 32 | enet | 0.028 | 0.002 | -2.35 | 0.0187 | 0.55 |
| CNNM4 | sCCA3 | 184 | 5 | lasso | 0.1 | 4.8e-09 | 2.4 | 0.0165 | 0.55 |
| NDUFV3 | sCCA3 | 521 | 11 | lasso | 0.54 | 3.6e-53 | -2.39 | 0.017029 | 0.55 |
| ZDHHC12 | sCCA2 | 264 | 1 | top1 | 0.05 | 5e-05 | -2.38 | 0.017117 | 0.55 |
| GPATCH4 | sCCA1 | 445 | 8 | lasso | 0.31 | 2e-26 | -2.35 | 0.0186 | 0.55 |
| PARS2 | sCCA3 | 536 | 7 | lasso | 0.098 | 1.3e-08 | -2.4 | 0.0166 | 0.55 |
| MAIP1 | sCCA1 | 370 | 6 | lasso | 0.18 | 2.3e-15 | 2.38 | 0.0172 | 0.55 |
| PMVK | sCCA2 | 398 | 14 | enet | 0.044 | 0.00013 | -2.4 | 0.016472 | 0.55 |
| CHCHD4 | sCCA2 | 574 | 47 | enet | 0.019 | 0.0088 | 2.39 | 0.016907 | 0.55 |
| U2SURP | sCCA1 | 511 | 1 | top1 | 0.11 | 2.3e-09 | -2.36 | 0.0182 | 0.55 |
| SUN1 | sCCA1 | 314 | 5 | lasso | 0.2 | 1.1e-16 | 2.36 | 0.01844 | 0.55 |
| TMEM67 | sCCA2 | 437 | 8 | enet | 0.0071 | 0.075 | -2.36 | 0.018091 | 0.55 |
| BORCS5 | sCCA3 | 531 | 16 | lasso | 0.31 | 9e-27 | -2.38 | 0.0175 | 0.55 |
| FRS2 | sCCA1 | 561 | 31 | enet | 0.36 | 2.5e-31 | 2.36 | 0.018188 | 0.55 |
| WBP1L | sCCA3 | 358 | 1 | top1 | 0.22 | 4.8e-18 | -2.39 | 0.0167 | 0.55 |
| WBP1L | sCCA2 | 358 | 23 | enet | 0.2 | 5e-17 | 2.36 | 0.0185 | 0.55 |
| C2CD3 | sCCA2 | 407 | 11 | lasso | 0.13 | 4.4e-11 | 2.35 | 0.018886 | 0.55 |
| HEXIM2 | sCCA3 | 279 | 4 | lasso | 0.075 | 7.6e-07 | -2.38 | 0.017298 | 0.55 |
| ZBTB43 | sCCA1 | 401 | 4 | lasso | 0.024 | 0.0038 | -2.37 | 0.01788 | 0.55 |
| THBS3 | sCCA3 | 333 | 29 | enet | 0.15 | 3.1e-12 | -2.4 | 0.0165 | 0.55 |
| INO80E | sCCA3 | 202 | 32 | enet | 0.58 | 8.5e-60 | 2.36 | 0.01806 | 0.55 |
| ARMC10 | sCCA1 | 240 | 10 | lasso | 0.33 | 3e-28 | 2.4 | 0.01623 | 0.55 |
| NUDT6 | sCCA2 | 335 | 40 | enet | 0.28 | 5.2e-24 | 2.38 | 0.01727 | 0.55 |
| TEFM | sCCA1 | 272 | 1 | top1 | 0.24 | 1.2e-19 | -2.37 | 0.017876 | 0.55 |
| MYOZ2 | sCCA1 | 436 | 15 | enet | 0.13 | 3.7e-11 | 2.34 | 0.01907 | 0.55 |
| SMPDL3A | sCCA3 | 418 | 37 | enet | 0.25 | 4.9e-21 | 2.35 | 0.01885 | 0.55 |
| C4orf32 | sCCA3 | 390 | 4 | lasso | 0.34 | 2.5e-29 | -2.36 | 0.01812 | 0.55 |
| UBE2C | sCCA3 | 531 | 1 | top1 | 0.09 | 5.4e-08 | 2.36 | 0.01814 | 0.55 |
| ARL10 | sCCA2 | 311 | 23 | enet | 0.23 | 5.1e-19 | 2.4 | 0.016364 | 0.55 |
| TTLL11 | sCCA1 | 527 | 8 | lasso | 0.38 | 9.4e-34 | 2.38 | 0.01733 | 0.55 |
| ATAD5 | sCCA1 | 279 | 1 | top1 | 0.28 | 9e-24 | 2.37 | 0.017876 | 0.55 |
| CRLF3 | sCCA2 | 277 | 25 | lasso | 0.42 | 2.1e-37 | 2.4 | 0.016356 | 0.55 |
| LACC1 | sCCA1 | 527 | 7 | lasso | 0.45 | 1.8e-41 | -2.38 | 0.0173 | 0.55 |
| SLC36A4 | sCCA2 | 444 | 10 | lasso | 0.15 | 7.3e-13 | -2.36 | 0.018522 | 0.55 |
| LRRC57 | sCCA3 | 332 | 36 | enet | 0.27 | 4.8e-23 | 2.35 | 0.01861 | 0.55 |
| LSMEM1 | sCCA2 | 407 | 9 | lasso | 0.31 | 1.9e-26 | -2.35 | 0.018863 | 0.55 |
| C3orf58 | sCCA2 | 435 | 29 | enet | 0.012 | 0.034 | -2.34 | 0.019175 | 0.55 |
| DDX41 | sCCA1 | 260 | 23 | enet | 0.013 | 0.025 | 2.39 | 0.017063 | 0.55 |
| TRAIP | sCCA1 | 336 | 14 | enet | 0.039 | 0.00029 | 2.39 | 0.0168 | 0.55 |
| ADAP2 | sCCA1 | 274 | 1 | top1 | 0.17 | 7.6e-14 | -2.39 | 0.016854 | 0.55 |
| NDUFA6 | sCCA2 | 340 | 51 | enet | 0.61 | 4.3e-64 | -2.4 | 0.016606 | 0.55 |
| NDUFA6 | sCCA1 | 340 | 56 | enet | 0.66 | 1.4e-72 | -2.37 | 0.01761 | 0.55 |
| ZNF793 | sCCA3 | 275 | 14 | lasso | 0.44 | 1.7e-40 | 2.35 | 0.0185 | 0.55 |
| FAM83G | sCCA3 | 176 | 21 | enet | 0.031 | 0.0012 | 2.36 | 0.018313 | 0.55 |
| NKAPL | sCCA3 | 424 | 6 | lasso | 0.034 | 0.00071 | 2.39 | 0.016999 | 0.55 |
| KIAA0408 | sCCA3 | 337 | 9 | lasso | 0.24 | 1.2e-19 | -2.36 | 0.01808 | 0.55 |
| TMEM63A | sCCA3 | 375 | 9 | lasso | 0.29 | 3.1e-24 | 2.36 | 0.0183 | 0.55 |
| TMEM63A | sCCA1 | 375 | 1 | top1 | 0.3 | 1.6e-25 | -2.35 | 0.0187 | 0.55 |
| ANAPC7 | sCCA3 | 226 | 10 | lasso | 0.004 | 0.14 | 2.38 | 0.0171 | 0.55 |
| MVB12B | sCCA1 | 489 | 22 | enet | 0.39 | 1.2e-34 | 2.36 | 0.01804 | 0.55 |
| ZNF585A | sCCA3 | 273 | 40 | enet | 0.22 | 1.5e-18 | -2.36 | 0.0183 | 0.55 |
| UAP1L1 | sCCA2 | 363 | 1 | top1 | 0.06 | 8.4e-06 | -2.36 | 0.018458 | 0.55 |
| HNRNPAB | sCCA1 | 420 | 1 | top1 | 0.11 | 2.1e-09 | -2.34 | 0.019082 | 0.55 |
| ZNF44 | sCCA1 | 272 | 1 | top1 | 0.02 | 0.0083 | 2.35 | 0.018996 | 0.55 |
| ZNF568 | sCCA1 | 283 | 33 | enet | 0.47 | 2.5e-43 | 2.36 | 0.018173 | 0.55 |
| ZNF587 | sCCA3 | 470 | 1 | top1 | 0.23 | 1.4e-19 | -2.39 | 0.0168 | 0.55 |
| C1orf228 | sCCA2 | 365 | 1 | top1 | 0.2 | 6.3e-17 | -2.38 | 0.017136 | 0.55 |
| PAPSS2 | sCCA1 | 323 | 4 | lasso | 0.52 | 5.6e-51 | -2.39 | 0.0169 | 0.55 |
| FAM169A | sCCA3 | 453 | 33 | enet | 0.017 | 0.014 | 2.39 | 0.01669 | 0.55 |
| RP11-296O14.3 | sCCA1 | 320 | 1 | top1 | 0.059 | 1.1e-05 | -2.39 | 0.017 | 0.55 |
| STARD7-AS1 | sCCA1 | 152 | 20 | enet | 0.12 | 1.7e-10 | 2.38 | 0.0174 | 0.55 |
| CFI | sCCA1 | 405 | 27 | enet | 0.086 | 1.2e-07 | 2.39 | 0.01673 | 0.55 |
| IGHG3 | sCCA2 | 43 | 24 | enet | 0.038 | 0.00038 | 2.39 | 0.0167 | 0.55 |
| CRIP1 | sCCA3 | 154 | 1 | top1 | 0.055 | 2.2e-05 | -2.35 | 0.0187 | 0.55 |
| RP11-563J2.2 | sCCA3 | 744 | 68 | enet | 0.16 | 3.9e-13 | 2.37 | 0.0179 | 0.55 |
| VPS16 | sCCA2 | 526 | 42 | enet | 0.12 | 3.9e-10 | -2.37 | 0.017855 | 0.55 |
| RPL21P28 | sCCA3 | 428 | 1 | top1 | 0.036 | 0.00049 | 2.35 | 0.0187 | 0.55 |
| MIR181A2HG | sCCA1 | 340 | 1 | top1 | 0.25 | 4.1e-21 | -2.35 | 0.01899 | 0.55 |
| RPSAP18 | sCCA3 | 517 | 1 | top1 | 0.042 | 0.00019 | -2.37 | 0.0177 | 0.55 |
| PSMD5-AS1 | sCCA1 | 357 | 19 | enet | 0.92 | 7.5e-167 | 2.39 | 0.01699 | 0.55 |
| PSMD5-AS1 | sCCA3 | 357 | 20 | enet | 0.88 | 1.4e-140 | -2.35 | 0.01897 | 0.55 |
| AC079807.2 | sCCA2 | 343 | 1 | top1 | 0.071 | 1.4e-06 | 2.4 | 0.0165 | 0.55 |
| RP4-756G23.5 | sCCA3 | 253 | 26 | enet | 0.22 | 1.4e-18 | -2.35 | 0.01893 | 0.55 |
| ASH1L-AS1 | sCCA1 | 223 | 16 | enet | 0.094 | 3e-08 | -2.35 | 0.0188 | 0.55 |
| FAM200B | sCCA1 | 558 | 29 | enet | 0.16 | 1.6e-13 | 2.35 | 0.01887 | 0.55 |
| BX470102.3 | sCCA1 | 427 | 1 | top1 | 0.061 | 8e-06 | 2.38 | 0.0174 | 0.55 |
| RPL7AP11 | sCCA3 | 326 | 1 | top1 | 0.032 | 0.001 | 2.38 | 0.017451 | 0.55 |
| ETV5 | sCCA1 | 437 | 38 | enet | 0.073 | 1e-06 | -2.35 | 0.0186 | 0.55 |
| LINC01024 | sCCA1 | 283 | 1 | top1 | 0.1 | 6.1e-09 | 2.39 | 0.017013 | 0.55 |
| LINC01094 | sCCA1 | 450 | 1 | top1 | 0.18 | 3.2e-15 | 2.4 | 0.01629 | 0.55 |
| RP11-809N8.5 | sCCA3 | 331 | 23 | enet | 0.034 | 0.00065 | 2.35 | 0.01902 | 0.55 |
| RP11-620J15.3 | sCCA1 | 406 | 23 | enet | 0.32 | 2.2e-27 | -2.39 | 0.016826 | 0.55 |
| RP11-588K22.2 | sCCA2 | 455 | 32 | enet | 0.068 | 2.2e-06 | 2.34 | 0.0191 | 0.55 |
| SNAI3-AS1 | sCCA1 | 382 | 19 | enet | 0.59 | 1.7e-60 | -2.4 | 0.01641 | 0.55 |
| RP11-669E14.6 | sCCA2 | 29 | 15 | enet | 0.14 | 4e-12 | 2.39 | 0.016973 | 0.55 |
| RP11-259G18.3 | sCCA1 | 65 | 24 | enet | 0.87 | 1.9e-137 | -2.35 | 0.018698 | 0.55 |
| ROCK1P1 | sCCA3 | 311 | 17 | enet | 0.6 | 1.8e-62 | -2.39 | 0.01704 | 0.55 |
| RP11-848P1.2 | sCCA3 | 288 | 1 | top1 | 0.049 | 6e-05 | -2.37 | 0.017715 | 0.55 |
| RP11-973H7.3 | sCCA1 | 372 | 13 | lasso | 0.22 | 1.1e-18 | -2.38 | 0.01722 | 0.55 |
| CTD-2342J14.6 | sCCA1 | 397 | 18 | enet | 0.035 | 0.00064 | 2.38 | 0.017331 | 0.55 |
| RP11-635N19.1 | sCCA1 | 506 | 6 | lasso | 0.46 | 4.3e-43 | 2.38 | 0.01751 | 0.55 |
| KCNQ1OT1 | sCCA3 | 513 | 14 | enet | 0.015 | 0.019 | 2.35 | 0.0189 | 0.55 |
| RP1-257I20.14 | sCCA2 | 340 | 9 | lasso | 0.26 | 5.7e-22 | -2.39 | 0.016867 | 0.55 |
| CAHM | sCCA2 | 567 | 26 | enet | 0.025 | 0.0032 | 2.35 | 0.01894 | 0.55 |
| RP11-307C12.12 | sCCA2 | 395 | 24 | enet | 0.0075 | 0.071 | 2.35 | 0.018836 | 0.55 |
| CTB-119C2.1 | sCCA3 | 525 | 9 | lasso | 0.062 | 6.8e-06 | -2.37 | 0.017935 | 0.55 |
| RP11-493E12.2 | sCCA3 | 303 | 23 | enet | 0.33 | 3e-28 | -2.34 | 0.0192 | 0.55 |
| ORAI1 | sCCA1 | 375 | 16 | enet | 0.29 | 3.4e-24 | -2.37 | 0.017555 | 0.55 |
| RP11-872J21.5 | sCCA1 | 572 | 4 | lasso | 0.061 | 7.3e-06 | 2.4 | 0.0166 | 0.55 |
| BAD | sCCA2 | 352 | 1 | top1 | 0.1 | 9e-09 | 2.32 | 0.020319 | 0.56 |
| PIAS1 | sCCA1 | 449 | 64 | enet | 0.49 | 3.1e-46 | -2.33 | 0.01975 | 0.56 |
| GNB1 | sCCA3 | 317 | 2 | lasso | 0.045 | 0.00011 | 2.32 | 0.0202 | 0.56 |
| DNAJA1 | sCCA2 | 478 | 10 | lasso | 0.043 | 0.00016 | -2.32 | 0.020227 | 0.56 |
| TIMP3 | sCCA1 | 685 | 10 | lasso | 0.14 | 1.3e-11 | -2.32 | 0.0205 | 0.56 |
| SBF1 | sCCA2 | 337 | 9 | enet | 0.0053 | 0.11 | -2.33 | 0.019854 | 0.56 |
| CEP76 | sCCA1 | 388 | 7 | lasso | 0.081 | 2.7e-07 | -2.34 | 0.01954 | 0.56 |
| VWA8 | sCCA2 | 512 | 29 | enet | 0.15 | 2.4e-12 | -2.33 | 0.0197 | 0.56 |
| SETD6 | sCCA2 | 475 | 8 | lasso | 0.29 | 1.1e-24 | -2.34 | 0.01931 | 0.56 |
| SPAG1 | sCCA1 | 370 | 32 | enet | 0.61 | 7.2e-64 | -2.34 | 0.0195 | 0.56 |
| GAR1 | sCCA2 | 405 | 7 | lasso | 0.017 | 0.012 | 2.34 | 0.01943 | 0.56 |
| PROC | sCCA2 | 418 | 4 | lasso | 0.29 | 7.6e-25 | 2.32 | 0.0205 | 0.56 |
| TXNDC12 | sCCA3 | 200 | 1 | top1 | 0.066 | 3.3e-06 | -2.33 | 0.0198 | 0.56 |
| FBXL5 | sCCA3 | 558 | 1 | top1 | 0.15 | 1.2e-12 | -2.33 | 0.01993 | 0.56 |
| RCL1 | sCCA3 | 605 | 26 | enet | 0.021 | 0.0066 | -2.33 | 0.02006 | 0.56 |
| TARDBP | sCCA1 | 380 | 32 | enet | 0.044 | 0.00014 | 2.32 | 0.0202 | 0.56 |
| SH2D3A | sCCA2 | 474 | 4 | lasso | 0.63 | 7.2e-68 | 2.33 | 0.019773 | 0.56 |
| ZNF304 | sCCA2 | 507 | 9 | lasso | 0.57 | 1.9e-58 | 2.32 | 0.020279 | 0.56 |
| EEFSEC | sCCA1 | 378 | 1 | top1 | 0.3 | 2.2e-25 | -2.32 | 0.0205 | 0.56 |
| SPIRE1 | sCCA3 | 375 | 37 | enet | 0.28 | 1.7e-23 | 2.34 | 0.01927 | 0.56 |
| BTF3L4 | sCCA3 | 200 | 1 | top1 | 0.043 | 0.00014 | 2.33 | 0.0198 | 0.56 |
| PHF11 | sCCA1 | 396 | 49 | enet | 0.28 | 1.4e-23 | -2.33 | 0.01956 | 0.56 |
| SETDB2 | sCCA3 | 403 | 40 | enet | 0.038 | 0.00034 | -2.33 | 0.0199 | 0.56 |
| CDK5RAP2 | sCCA2 | 340 | 17 | enet | 0.032 | 0.0011 | -2.32 | 0.020365 | 0.56 |
| TUBGCP4 | sCCA1 | 269 | 7 | lasso | 0.11 | 3.7e-09 | 2.33 | 0.02002 | 0.56 |
| MVB12A | sCCA2 | 441 | 1 | top1 | 0.15 | 2.7e-12 | -2.34 | 0.01927 | 0.56 |
| CYP4B1 | sCCA2 | 382 | 6 | lasso | 0.21 | 2.3e-17 | -2.33 | 0.019599 | 0.56 |
| CALM2 | sCCA2 | 551 | 15 | enet | 0.092 | 3.7e-08 | -2.33 | 0.0198 | 0.56 |
| POMGNT2 | sCCA3 | 371 | 1 | top1 | 0.059 | 1.1e-05 | -2.32 | 0.020371 | 0.56 |
| TERF1 | sCCA1 | 573 | 36 | enet | 0.034 | 0.00066 | -2.32 | 0.0204 | 0.56 |
| MAPK13 | sCCA2 | 372 | 40 | enet | 0.4 | 1e-35 | -2.33 | 0.019885 | 0.56 |
| GPR153 | sCCA3 | 447 | 15 | enet | 0.04 | 0.00026 | -2.32 | 0.0205 | 0.56 |
| ZNF577 | sCCA2 | 616 | 1 | top1 | 0.19 | 1.9e-15 | 2.34 | 0.019471 | 0.56 |
| DCD | sCCA1 | 420 | 17 | enet | 0.029 | 0.0017 | 2.33 | 0.020071 | 0.56 |
| NEU3 | sCCA1 | 444 | 8 | lasso | 0.27 | 9.8e-23 | 2.34 | 0.019363 | 0.56 |
| ERCC3 | sCCA3 | 454 | 10 | lasso | 0.56 | 1.8e-55 | 2.34 | 0.0193 | 0.56 |
| LRRC2 | sCCA3 | 372 | 37 | enet | 0.14 | 7.9e-12 | 2.32 | 0.020482 | 0.56 |
| ZMYM6 | sCCA3 | 299 | 21 | enet | 0.011 | 0.04 | -2.32 | 0.0203 | 0.56 |
| INO80E | sCCA2 | 202 | 26 | enet | 0.41 | 1.5e-36 | 2.33 | 0.02001 | 0.56 |
| SMPDL3A | sCCA1 | 418 | 29 | enet | 0.41 | 6.8e-37 | -2.33 | 0.01972 | 0.56 |
| DAG1 | sCCA2 | 288 | 1 | top1 | 0.17 | 6.5e-14 | -2.33 | 0.019756 | 0.56 |
| DAG1 | sCCA1 | 288 | 1 | top1 | 0.19 | 3.8e-16 | -2.33 | 0.0198 | 0.56 |
| CDC42EP4 | sCCA1 | 585 | 14 | enet | 0.16 | 2.7e-13 | -2.32 | 0.020154 | 0.56 |
| PCBP3 | sCCA3 | 487 | 9 | lasso | 0.48 | 1.4e-45 | -2.33 | 0.01955 | 0.56 |
| OLFML1 | sCCA2 | 661 | 26 | enet | 0.04 | 0.00025 | 2.32 | 0.020222 | 0.56 |
| ARL17A | sCCA1 | 142 | 21 | enet | 0.56 | 5.6e-56 | -2.33 | 0.019826 | 0.56 |
| BLOC1S4 | sCCA3 | 553 | 4 | lasso | 0.02 | 0.0073 | 2.33 | 0.01958 | 0.56 |
| PRR13P5 | sCCA3 | 400 | 22 | enet | 0.027 | 0.0021 | 2.33 | 0.0197 | 0.56 |
| ADAT2 | sCCA3 | 513 | 38 | enet | 0.59 | 1.6e-60 | 2.32 | 0.02012 | 0.56 |
| KIAA0408 | sCCA2 | 337 | 12 | lasso | 0.059 | 1.1e-05 | -2.34 | 0.019323 | 0.56 |
| ZNF585A | sCCA1 | 273 | 41 | enet | 0.22 | 1.6e-18 | 2.34 | 0.019532 | 0.56 |
| STAG3L1 | sCCA1 | 160 | 78 | enet | 0.52 | 1.7e-50 | -2.33 | 0.01992 | 0.56 |
| PSMD5-AS1 | sCCA2 | 357 | 14 | enet | 0.9 | 5.2e-152 | -2.32 | 0.020127 | 0.56 |
| RPS4XP11 | sCCA2 | 368 | 16 | enet | 0.013 | 0.028 | -2.33 | 0.0196 | 0.56 |
| RPS14P8 | sCCA2 | 589 | 16 | enet | 0.061 | 7.9e-06 | -2.33 | 0.020024 | 0.56 |
| RP11-30L15.4 | sCCA1 | 423 | 1 | top1 | 0.074 | 8.7e-07 | 2.32 | 0.0204 | 0.56 |
| SNAI3-AS1 | sCCA3 | 382 | 1 | top1 | 0.24 | 4.8e-20 | -2.33 | 0.01994 | 0.56 |
| RP5-882C2.2 | sCCA1 | 342 | 1 | top1 | 0.12 | 2.7e-10 | 2.33 | 0.019998 | 0.56 |
| RP5-882C2.2 | sCCA3 | 342 | 1 | top1 | 0.041 | 0.00021 | -2.33 | 0.019998 | 0.56 |
| RP11-259G18.3 | sCCA3 | 65 | 16 | lasso | 0.79 | 1.3e-105 | -2.32 | 0.020395 | 0.56 |
| AP001462.6 | sCCA2 | 344 | 1 | top1 | 0.062 | 6.6e-06 | 2.33 | 0.019582 | 0.56 |
| CTA-384D8.36 | sCCA2 | 321 | 8 | lasso | 0.19 | 1.4e-15 | 2.33 | 0.019982 | 0.56 |
| CTD-2267D19.1 | sCCA2 | 348 | 11 | lasso | 0.0062 | 0.09 | 2.32 | 0.020236 | 0.56 |
| QPCTL | sCCA1 | 370 | 28 | enet | 0.12 | 4.6e-10 | 2.31 | 0.020631 | 0.57 |
| DDX17 | sCCA3 | 366 | 4 | lasso | 0.017 | 0.014 | 2.3 | 0.02124 | 0.57 |
| CD83 | sCCA3 | 489 | 1 | top1 | 0.12 | 4e-10 | -2.31 | 0.02106 | 0.57 |
| PPP2R5D | sCCA3 | 368 | 6 | lasso | 0.073 | 9.4e-07 | -2.31 | 0.02105 | 0.57 |
| MAN2A1 | sCCA2 | 427 | 29 | enet | 0.062 | 6.9e-06 | -2.31 | 0.020911 | 0.57 |
| SLC4A3 | sCCA3 | 487 | 1 | top1 | 0.068 | 2.2e-06 | -2.31 | 0.021 | 0.57 |
| IGFBP2 | sCCA1 | 532 | 25 | enet | 0.29 | 6.4e-25 | 2.31 | 0.0212 | 0.57 |
| KANSL1 | sCCA3 | 46 | 19 | enet | 0.3 | 1.6e-25 | -2.3 | 0.021394 | 0.57 |
| MTHFS | sCCA3 | 573 | 61 | enet | 0.68 | 4e-77 | -2.32 | 0.0206 | 0.57 |
| RDX | sCCA3 | 388 | 1 | top1 | 0.073 | 9.9e-07 | -2.31 | 0.02081 | 0.57 |
| SEC24D | sCCA2 | 377 | 27 | enet | 0.053 | 3e-05 | 2.3 | 0.02129 | 0.57 |
| FARP1 | sCCA1 | 579 | 1 | top1 | 0.25 | 7e-21 | 2.3 | 0.02123 | 0.57 |
| ACSS1 | sCCA1 | 481 | 13 | lasso | 0.17 | 4.7e-14 | -2.31 | 0.02097 | 0.57 |
| ALG8 | sCCA3 | 341 | 45 | enet | 0.32 | 3.1e-27 | -2.3 | 0.02128 | 0.57 |
| TKT | sCCA1 | 404 | 6 | lasso | 0.33 | 1.8e-28 | 2.31 | 0.0209 | 0.57 |
| WASHC5 | sCCA3 | 489 | 11 | lasso | 0.0062 | 0.09 | 2.31 | 0.0211 | 0.57 |
| SMPDL3A | sCCA2 | 418 | 28 | enet | 0.29 | 4.1e-24 | -2.3 | 0.021356 | 0.57 |
| C11orf45 | sCCA2 | 551 | 7 | lasso | 0.2 | 1e-16 | 2.32 | 0.020566 | 0.57 |
| RPLP2 | sCCA2 | 423 | 8 | lasso | 0.11 | 2.2e-09 | 2.3 | 0.02122 | 0.57 |
| MYOZ1 | sCCA1 | 248 | 11 | lasso | 0.21 | 7.7e-18 | 2.32 | 0.0206 | 0.57 |
| ZNF527 | sCCA1 | 292 | 1 | top1 | 0.041 | 0.00024 | 2.3 | 0.021207 | 0.57 |
| KIAA0408 | sCCA1 | 337 | 8 | lasso | 0.25 | 2.8e-21 | 2.31 | 0.02114 | 0.57 |
| GRK6 | sCCA3 | 267 | 20 | enet | 0.049 | 5.3e-05 | -2.31 | 0.020754 | 0.57 |
| TCEA3 | sCCA1 | 313 | 7 | lasso | 0.3 | 7.4e-26 | 2.31 | 0.0208 | 0.57 |
| CNEP1R1 | sCCA3 | 383 | 8 | lasso | 0.011 | 0.036 | -2.31 | 0.02096 | 0.57 |
| STAG3L1 | sCCA2 | 160 | 86 | enet | 0.54 | 2.5e-53 | -2.31 | 0.021018 | 0.57 |
| LINC01422 | sCCA2 | 865 | 17 | lasso | 0.41 | 1.3e-36 | -2.31 | 0.020703 | 0.57 |
| RPSAP9 | sCCA3 | 663 | 1 | top1 | 0.057 | 1.6e-05 | -2.31 | 0.02115 | 0.57 |
| RP11-1280N14.3 | sCCA2 | 72 | 1 | top1 | 0.029 | 0.0018 | 2.31 | 0.021128 | 0.57 |
| ZNF10 | sCCA3 | 163 | 5 | lasso | 0.44 | 6.9e-40 | -2.31 | 0.0207 | 0.57 |
| MMP25-AS1 | sCCA3 | 370 | 1 | top1 | 0.072 | 1.2e-06 | -2.31 | 0.02082 | 0.57 |
| RP11-355B11.2 | sCCA2 | 299 | 15 | lasso | 0.2 | 2.2e-16 | -2.31 | 0.0208 | 0.57 |
| RP11-464F9.22 | sCCA2 | 252 | 4 | lasso | 0.028 | 0.0021 | -2.3 | 0.0213 | 0.57 |
| RP11-640N20.4 | sCCA2 | 288 | 7 | lasso | 0.069 | 2e-06 | 2.3 | 0.021343 | 0.57 |
| RP11-893F2.18 | sCCA2 | 487 | 4 | lasso | 0.063 | 5.2e-06 | -2.31 | 0.021094 | 0.57 |
| ENPP4 | sCCA3 | 503 | 22 | enet | 0.25 | 3.5e-21 | -2.3 | 0.02173 | 0.58 |
| RNF19A | sCCA3 | 395 | 41 | enet | 0.1 | 6.4e-09 | -2.27 | 0.02317 | 0.58 |
| LTBP1 | sCCA2 | 490 | 25 | enet | 0.16 | 1.3e-13 | -2.29 | 0.0218 | 0.58 |
| TRNT1 | sCCA1 | 811 | 5 | lasso | 0.44 | 1.4e-40 | -2.28 | 0.0225 | 0.58 |
| RAB21 | sCCA2 | 402 | 5 | lasso | 0.044 | 0.00013 | 2.28 | 0.022687 | 0.58 |
| PAPOLA | sCCA1 | 553 | 8 | lasso | 0.25 | 6.3e-21 | 2.27 | 0.02322 | 0.58 |
| RAB11FIP3 | sCCA2 | 397 | 19 | enet | 0.05 | 4.6e-05 | 2.28 | 0.02276 | 0.58 |
| SMARCB1 | sCCA1 | 464 | 11 | lasso | 0.61 | 5.1e-64 | 2.27 | 0.02302 | 0.58 |
| DDT | sCCA1 | 416 | 38 | enet | 0.77 | 1.6e-98 | 2.28 | 0.02257 | 0.58 |
| HSCB | sCCA2 | 307 | 1 | top1 | 0.12 | 4.2e-10 | -2.29 | 0.021772 | 0.58 |
| STK4 | sCCA2 | 422 | 24 | enet | 0.086 | 1e-07 | -2.29 | 0.022161 | 0.58 |
| SIRPB1 | sCCA3 | 627 | 10 | lasso | 0.78 | 3.1e-103 | 2.29 | 0.02182 | 0.58 |
| SIRPB1 | sCCA1 | 627 | 16 | lasso | 0.82 | 1.1e-114 | -2.28 | 0.02255 | 0.58 |
| SMG9 | sCCA2 | 427 | 1 | top1 | 0.12 | 4.6e-10 | -2.29 | 0.022304 | 0.58 |
| GLCCI1 | sCCA3 | 709 | 23 | enet | 0.048 | 7.3e-05 | -2.29 | 0.02224 | 0.58 |
| RHEB | sCCA1 | 552 | 20 | enet | 0.26 | 5.2e-22 | 2.3 | 0.02146 | 0.58 |
| EIF3A | sCCA2 | 488 | 11 | enet | 0.0018 | 0.21 | -2.29 | 0.022 | 0.58 |
| PSMD11 | sCCA2 | 260 | 7 | enet | 0.013 | 0.024 | -2.3 | 0.021723 | 0.58 |
| RNF141 | sCCA2 | 525 | 29 | enet | 0.017 | 0.013 | -2.29 | 0.022102 | 0.58 |
| PTP4A1 | sCCA3 | 275 | 3 | lasso | 0.057 | 1.4e-05 | 2.29 | 0.02205 | 0.58 |
| ENPP5 | sCCA3 | 514 | 49 | enet | 0.066 | 3.1e-06 | 2.27 | 0.02303 | 0.58 |
| FHL2 | sCCA1 | 594 | 1 | top1 | 0.14 | 1e-11 | 2.29 | 0.0221 | 0.58 |
| PPP1R7 | sCCA2 | 417 | 1 | top1 | 0.04 | 0.00026 | -2.28 | 0.0229 | 0.58 |
| DARS2 | sCCA2 | 275 | 8 | lasso | 0.18 | 2.2e-15 | -2.28 | 0.022785 | 0.58 |
| FBXL5 | sCCA1 | 558 | 1 | top1 | 0.18 | 3.9e-15 | -2.29 | 0.02211 | 0.58 |
| LYPLA1 | sCCA1 | 420 | 1 | top1 | 0.089 | 6.3e-08 | 2.27 | 0.0231 | 0.58 |
| SEPT7 | sCCA2 | 579 | 22 | enet | 0.043 | 0.00016 | 2.28 | 0.022425 | 0.58 |
| EFNB2 | sCCA2 | 511 | 1 | top1 | 0.087 | 9.1e-08 | 2.3 | 0.0216 | 0.58 |
| DTD2 | sCCA1 | 322 | 10 | lasso | 0.45 | 6.8e-42 | -2.28 | 0.02241 | 0.58 |
| ECSIT | sCCA3 | 384 | 29 | enet | 0.091 | 4.4e-08 | -2.28 | 0.0227 | 0.58 |
| ANKHD1 | sCCA2 | 311 | 2 | lasso | 0.022 | 0.0054 | -2.29 | 0.022157 | 0.58 |
| C1orf159 | sCCA1 | 218 | 12 | enet | 0.46 | 3.3e-43 | -2.29 | 0.0218 | 0.58 |
| CHSY1 | sCCA3 | 623 | 1 | top1 | 0.018 | 0.012 | -2.28 | 0.02276 | 0.58 |
| PRMT7 | sCCA3 | 316 | 29 | enet | 0.47 | 8e-44 | 2.29 | 0.02204 | 0.58 |
| CD63 | sCCA3 | 308 | 25 | enet | 0.056 | 1.6e-05 | -2.28 | 0.0228 | 0.58 |
| STX11 | sCCA1 | 400 | 1 | top1 | 0.11 | 1.6e-09 | 2.28 | 0.02261 | 0.58 |
| MRPL44 | sCCA1 | 475 | 4 | lasso | 0.19 | 1.3e-15 | 2.3 | 0.0215 | 0.58 |
| MTHFS | sCCA2 | 573 | 35 | enet | 0.68 | 2.7e-77 | -2.29 | 0.02181 | 0.58 |
| RIPK1 | sCCA3 | 659 | 19 | enet | 0.4 | 1.5e-35 | 2.29 | 0.02174 | 0.58 |
| RIPK1 | sCCA1 | 659 | 19 | enet | 0.54 | 1.2e-52 | -2.29 | 0.02183 | 0.58 |
| SYTL2 | sCCA3 | 376 | 15 | enet | 0.1 | 7.6e-09 | -2.27 | 0.02308 | 0.58 |
| ARL3 | sCCA1 | 365 | 15 | enet | 0.33 | 1.2e-28 | 2.28 | 0.0226 | 0.58 |
| USO1 | sCCA1 | 459 | 1 | top1 | 0.18 | 1e-14 | 2.28 | 0.0229 | 0.58 |
| RNF185 | sCCA2 | 364 | 5 | lasso | 0.13 | 2.4e-11 | -2.27 | 0.023195 | 0.58 |
| ACVRL1 | sCCA1 | 535 | 1 | top1 | 0.13 | 8e-11 | -2.29 | 0.022139 | 0.58 |
| SLC12A6 | sCCA2 | 450 | 47 | enet | 0.14 | 6.4e-12 | -2.29 | 0.02194 | 0.58 |
| RAB5A | sCCA2 | 380 | 43 | enet | 0.15 | 8.8e-13 | -2.3 | 0.021514 | 0.58 |
| ZNF92 | sCCA3 | 160 | 5 | lasso | 0.24 | 3.2e-20 | 2.3 | 0.021699 | 0.58 |
| CSGALNACT1 | sCCA2 | 673 | 48 | enet | 0.42 | 2e-38 | 2.27 | 0.023162 | 0.58 |
| NFIB | sCCA3 | 753 | 11 | lasso | 0.0083 | 0.06 | -2.28 | 0.02252 | 0.58 |
| ITGB1 | sCCA1 | 478 | 1 | top1 | 0.28 | 2.2e-23 | 2.28 | 0.0224 | 0.58 |
| ABCB9 | sCCA3 | 312 | 12 | lasso | 0.18 | 1.3e-14 | -2.29 | 0.022 | 0.58 |
| N6AMT1 | sCCA2 | 470 | 3 | lasso | 0.19 | 5.2e-16 | -2.29 | 0.02218 | 0.58 |
| USP16 | sCCA1 | 425 | 21 | lasso | 0.11 | 1.1e-09 | 2.29 | 0.021887 | 0.58 |
| ARHGAP27 | sCCA1 | 183 | 8 | lasso | 0.42 | 6.2e-38 | 2.28 | 0.022894 | 0.58 |
| RECQL4 | sCCA2 | 237 | 9 | lasso | 0.03 | 0.0014 | 2.28 | 0.0225 | 0.58 |
| FBXO27 | sCCA1 | 402 | 45 | enet | 0.8 | 2.1e-107 | 2.28 | 0.022358 | 0.58 |
| ZNF382 | sCCA2 | 300 | 18 | enet | 0.092 | 4e-08 | -2.29 | 0.021823 | 0.58 |
| GPR155 | sCCA1 | 365 | 9 | lasso | 0.26 | 3.6e-22 | -2.27 | 0.0232 | 0.58 |
| ITGA2 | sCCA3 | 577 | 19 | lasso | 0.37 | 1.9e-32 | 2.29 | 0.021947 | 0.58 |
| MAP1A | sCCA2 | 266 | 3 | lasso | 0.066 | 3.3e-06 | 2.29 | 0.0222 | 0.58 |
| MAP1A | sCCA1 | 266 | 3 | lasso | 0.082 | 2.2e-07 | 2.27 | 0.02317 | 0.58 |
| MGAT2 | sCCA3 | 382 | 24 | enet | 0.016 | 0.016 | -2.3 | 0.021707 | 0.58 |
| CHD3 | sCCA3 | 506 | 1 | top1 | -0.00085 | 0.39 | 2.29 | 0.022148 | 0.58 |
| TRIAP1 | sCCA2 | 390 | 9 | enet | 0.057 | 1.6e-05 | -2.27 | 0.02328 | 0.58 |
| ARL10 | sCCA1 | 311 | 5 | lasso | 0.25 | 8e-21 | -2.27 | 0.022962 | 0.58 |
| TTLL11 | sCCA3 | 527 | 9 | lasso | 0.38 | 9.2e-34 | 2.27 | 0.02313 | 0.58 |
| TGIF1 | sCCA1 | 500 | 18 | enet | 0.019 | 0.0095 | 2.29 | 0.02195 | 0.58 |
| ZNF518A | sCCA1 | 463 | 22 | enet | 0.32 | 3.3e-27 | -2.29 | 0.0219 | 0.58 |
| DPM3 | sCCA3 | 349 | 4 | lasso | 0.04 | 0.00026 | -2.27 | 0.023 | 0.58 |
| NRIP1 | sCCA3 | 511 | 20 | enet | 0.13 | 8.2e-11 | -2.27 | 0.023053 | 0.58 |
| PSMG4 | sCCA2 | 612 | 38 | enet | 0.56 | 1.5e-55 | 2.27 | 0.023356 | 0.58 |
| LSMEM1 | sCCA3 | 407 | 19 | enet | 0.33 | 2.8e-28 | 2.27 | 0.023034 | 0.58 |
| COL18A1 | sCCA3 | 479 | 22 | enet | 0.13 | 3.9e-11 | -2.27 | 0.023109 | 0.58 |
| PCBP3 | sCCA1 | 487 | 12 | lasso | 0.55 | 5.3e-55 | 2.29 | 0.022279 | 0.58 |
| KNTC1 | sCCA3 | 311 | 4 | lasso | 0.13 | 3.6e-11 | 2.3 | 0.0216 | 0.58 |
| LPAR5 | sCCA2 | 297 | 14 | enet | 0.12 | 2.7e-10 | 2.28 | 0.022465 | 0.58 |
| ZNF793 | sCCA1 | 275 | 11 | lasso | 0.45 | 3e-41 | 2.3 | 0.021577 | 0.58 |
| SUPT3H | sCCA2 | 362 | 9 | lasso | 0.19 | 9e-16 | -2.28 | 0.022465 | 0.58 |
| ARID5A | sCCA3 | 212 | 1 | top1 | 0.043 | 0.00017 | 2.28 | 0.0224 | 0.58 |
| PDLIM7 | sCCA2 | 264 | 22 | enet | 0.23 | 1.9e-19 | 2.29 | 0.022 | 0.58 |
| ZNF607 | sCCA1 | 298 | 34 | enet | 0.45 | 4.6e-41 | -2.28 | 0.022406 | 0.58 |
| ANKRD39 | sCCA3 | 169 | 1 | top1 | 0.021 | 0.0069 | 2.3 | 0.0217 | 0.58 |
| HNRNPA1P10 | sCCA2 | 349 | 30 | enet | 0.037 | 0.00041 | -2.29 | 0.021804 | 0.58 |
| PPIAP31 | sCCA2 | 358 | 1 | top1 | 0.018 | 0.011 | 2.3 | 0.0215 | 0.58 |
| C6orf226 | sCCA3 | 356 | 30 | enet | 0.25 | 1.2e-20 | -2.27 | 0.02296 | 0.58 |
| PLEKHM1 | sCCA1 | 160 | 2 | lasso | 0.36 | 5.4e-31 | -2.3 | 0.021537 | 0.58 |
| GPX1 | sCCA3 | 276 | 21 | enet | 0.31 | 1.7e-26 | -2.29 | 0.02217 | 0.58 |
| GPX1 | sCCA1 | 276 | 19 | enet | 0.3 | 5.9e-26 | -2.27 | 0.0231 | 0.58 |
| PCDHGC3 | sCCA1 | 499 | 22 | enet | 0.036 | 0.00052 | -2.27 | 0.023171 | 0.58 |
| RP1-179N16.6 | sCCA2 | 409 | 11 | lasso | 0.57 | 2.9e-58 | -2.29 | 0.02174 | 0.58 |
| RP11-933H2.4 | sCCA2 | 423 | 1 | top1 | 0.0038 | 0.14 | 2.29 | 0.022289 | 0.58 |
| AP000487.6 | sCCA1 | 427 | 1 | top1 | 0.11 | 9.7e-10 | -2.3 | 0.021614 | 0.58 |
| CTSO | sCCA1 | 470 | 5 | lasso | 0.35 | 1.9e-30 | -2.28 | 0.02245 | 0.58 |
| ZNF625 | sCCA2 | 295 | 15 | enet | 0.038 | 0.00035 | 2.27 | 0.022974 | 0.58 |
| RP11-259G18.3 | sCCA2 | 65 | 19 | lasso | 0.84 | 8.7e-123 | 2.3 | 0.021728 | 0.58 |
| CTD-2349P21.9 | sCCA1 | 268 | 34 | enet | 0.38 | 1.6e-33 | 2.28 | 0.022748 | 0.58 |
| RP11-635N19.1 | sCCA3 | 506 | 5 | lasso | 0.29 | 2e-24 | -2.28 | 0.02289 | 0.58 |
| RP11-686D22.4 | sCCA1 | 368 | 9 | lasso | 0.46 | 4.2e-42 | 2.29 | 0.021857 | 0.58 |
| RP11-563N4.1 | sCCA2 | 270 | 24 | enet | 0.099 | 1.1e-08 | -2.29 | 0.0218 | 0.58 |
| HIST1H3H | sCCA2 | 504 | 1 | top1 | 0.042 | 0.00019 | 2.28 | 0.0229 | 0.58 |
| RP11-707O23.1 | sCCA2 | 113 | 35 | enet | 0.72 | 4.5e-86 | -2.3 | 0.021661 | 0.58 |
| HSPB6 | sCCA2 | 426 | 52 | enet | 0.028 | 0.002 | -2.24 | 0.024981 | 0.59 |
| IPO5 | sCCA2 | 549 | 3 | lasso | 0.023 | 0.0046 | -2.25 | 0.02449 | 0.59 |
| IP6K2 | sCCA3 | 273 | 11 | lasso | 0.17 | 3.3e-14 | 2.26 | 0.023736 | 0.59 |
| TP73 | sCCA3 | 541 | 16 | enet | 0.068 | 2.4e-06 | -2.24 | 0.0251 | 0.59 |
| COL9A3 | sCCA2 | 523 | 51 | enet | 0.005 | 0.11 | -2.26 | 0.024065 | 0.59 |
| DERL3 | sCCA2 | 446 | 7 | lasso | 0.099 | 1.2e-08 | 2.26 | 0.023613 | 0.59 |
| TAB1 | sCCA3 | 386 | 15 | enet | 0.024 | 0.0038 | -2.26 | 0.02413 | 0.59 |
| PLTP | sCCA2 | 515 | 55 | enet | 0.29 | 2.1e-24 | -2.24 | 0.024827 | 0.59 |
| PRELID3B | sCCA2 | 561 | 19 | enet | 0.26 | 6.9e-22 | 2.26 | 0.023689 | 0.59 |
| MYL12A | sCCA3 | 489 | 7 | lasso | 0.14 | 5.5e-12 | -2.25 | 0.02472 | 0.59 |
| SLC7A6 | sCCA1 | 307 | 8 | lasso | 0.35 | 1.3e-30 | 2.25 | 0.02447 | 0.59 |
| PPP2R1A | sCCA2 | 547 | 1 | top1 | 0.074 | 7.8e-07 | 2.26 | 0.023807 | 0.59 |
| SMG9 | sCCA3 | 427 | 6 | lasso | 0.21 | 4.7e-17 | 2.26 | 0.024 | 0.59 |
| LSM5 | sCCA2 | 487 | 44 | enet | 0.09 | 5.8e-08 | -2.26 | 0.023522 | 0.59 |
| PRUNE2 | sCCA1 | 533 | 5 | lasso | 0.59 | 4.3e-61 | 2.24 | 0.02495 | 0.59 |
| AMBP | sCCA3 | 674 | 16 | enet | 0.024 | 0.0041 | 2.26 | 0.02362 | 0.59 |
| CUEDC2 | sCCA3 | 341 | 1 | top1 | 0.075 | 7.3e-07 | -2.26 | 0.0238 | 0.59 |
| STN1 | sCCA2 | 428 | 4 | lasso | 0.0018 | 0.21 | -2.26 | 0.024 | 0.59 |
| WNT3 | sCCA1 | 255 | 7 | lasso | 0.58 | 1.9e-59 | -2.24 | 0.024993 | 0.59 |
| PROC | sCCA1 | 418 | 1 | top1 | 0.32 | 3.9e-27 | -2.25 | 0.0247 | 0.59 |
| RPS6KA1 | sCCA2 | 352 | 8 | lasso | 0.079 | 3.4e-07 | -2.25 | 0.024181 | 0.59 |
| NENF | sCCA1 | 472 | 7 | lasso | 0.23 | 4.7e-19 | 2.24 | 0.0249 | 0.59 |
| RPL21 | sCCA2 | 604 | 1 | top1 | 0.028 | 0.0021 | -2.26 | 0.02391 | 0.59 |
| CHST3 | sCCA2 | 563 | 1 | top1 | 0.0036 | 0.15 | -2.25 | 0.0242 | 0.59 |
| PMS2P5 | sCCA1 | 97 | 13 | enet | 0.57 | 3.3e-57 | -2.26 | 0.02358 | 0.59 |
| TOX2 | sCCA3 | 526 | 30 | enet | 0.4 | 4.2e-36 | 2.27 | 0.02345 | 0.59 |
| HECTD3 | sCCA1 | 316 | 4 | lasso | 0.064 | 4.6e-06 | 2.25 | 0.0241 | 0.59 |
| CAPNS1 | sCCA2 | 332 | 60 | enet | 0.025 | 0.0035 | 2.25 | 0.024465 | 0.59 |
| FLRT1 | sCCA1 | 319 | 10 | lasso | 0.25 | 4.6e-21 | 2.25 | 0.024749 | 0.59 |
| PTPN12 | sCCA3 | 372 | 56 | enet | 0.37 | 1.7e-32 | 2.25 | 0.024315 | 0.59 |
| SIGLEC9 | sCCA3 | 622 | 6 | lasso | 0.044 | 0.00013 | -2.24 | 0.025 | 0.59 |
| CYP2E1 | sCCA3 | 288 | 34 | enet | 0.27 | 1.1e-22 | 2.27 | 0.0235 | 0.59 |
| C1QL1 | sCCA1 | 348 | 1 | top1 | 0.11 | 2.7e-09 | -2.26 | 0.023746 | 0.59 |
| MAP1B | sCCA2 | 451 | 1 | top1 | 0.49 | 3.2e-46 | -2.25 | 0.02429 | 0.59 |
| ZNF304 | sCCA1 | 507 | 36 | enet | 0.72 | 1.8e-86 | 2.26 | 0.02409 | 0.59 |
| EEFSEC | sCCA2 | 378 | 7 | lasso | 0.21 | 2.6e-17 | 2.26 | 0.02399 | 0.59 |
| KCNH3 | sCCA1 | 314 | 1 | top1 | 0.1 | 5.2e-09 | -2.25 | 0.024695 | 0.59 |
| FLNB | sCCA2 | 400 | 9 | lasso | 0.21 | 2.3e-17 | 2.25 | 0.024634 | 0.59 |
| RBM26 | sCCA3 | 395 | 21 | enet | 0.086 | 1.1e-07 | -2.25 | 0.0243 | 0.59 |
| LRRC46 | sCCA1 | 382 | 24 | enet | 0.56 | 7.8e-56 | -2.25 | 0.024526 | 0.59 |
| RPRD1A | sCCA2 | 460 | 8 | lasso | 0.16 | 3.8e-13 | 2.25 | 0.0242 | 0.59 |
| PELP1 | sCCA3 | 435 | 20 | enet | 0.01 | 0.041 | 2.25 | 0.024687 | 0.59 |
| CYP4B1 | sCCA1 | 382 | 38 | enet | 0.53 | 1.8e-52 | -2.26 | 0.0241 | 0.59 |
| CSGALNACT1 | sCCA1 | 673 | 65 | enet | 0.61 | 2.1e-63 | -2.25 | 0.0243 | 0.59 |
| PPRC1 | sCCA1 | 277 | 28 | enet | 0.014 | 0.024 | 2.24 | 0.0248 | 0.59 |
| SAP18 | sCCA3 | 416 | 1 | top1 | 0.13 | 6.7e-11 | 2.26 | 0.0235 | 0.59 |
| SAP18 | sCCA1 | 416 | 1 | top1 | 0.23 | 2.6e-19 | -2.26 | 0.02352 | 0.59 |
| RANBP2 | sCCA1 | 370 | 31 | enet | 0.086 | 1e-07 | -2.26 | 0.024 | 0.59 |
| SH3RF1 | sCCA2 | 379 | 1 | top1 | 0.053 | 2.8e-05 | -2.24 | 0.02494 | 0.59 |
| FTCD | sCCA1 | 495 | 57 | enet | 0.43 | 5e-39 | -2.25 | 0.024152 | 0.59 |
| ZNF577 | sCCA1 | 616 | 9 | lasso | 0.28 | 6.3e-24 | 2.25 | 0.024149 | 0.59 |
| ERCC3 | sCCA1 | 454 | 7 | lasso | 0.62 | 5.1e-65 | 2.24 | 0.0249 | 0.59 |
| PPM1K | sCCA3 | 460 | 24 | enet | 0.053 | 2.8e-05 | -2.26 | 0.02394 | 0.59 |
| PLB1 | sCCA1 | 427 | 14 | enet | 0.24 | 8.4e-20 | -2.25 | 0.0244 | 0.59 |
| TRUB2 | sCCA1 | 288 | 1 | top1 | 0.23 | 3.6e-19 | 2.24 | 0.02506 | 0.59 |
| MT1E | sCCA1 | 521 | 45 | enet | 0.49 | 1.8e-46 | 2.26 | 0.02385 | 0.59 |
| TLR1 | sCCA2 | 525 | 1 | top1 | 0.064 | 4.7e-06 | -2.25 | 0.02439 | 0.59 |
| FZD4 | sCCA1 | 588 | 6 | lasso | 0.11 | 1.3e-09 | -2.24 | 0.024885 | 0.59 |
| MYOZ1 | sCCA2 | 248 | 8 | lasso | 0.21 | 2.5e-17 | -2.26 | 0.0237 | 0.59 |
| ZNF552 | sCCA1 | 487 | 9 | lasso | 0.22 | 2.3e-18 | -2.25 | 0.024409 | 0.59 |
| PCED1B | sCCA2 | 442 | 8 | lasso | 0.06 | 9.5e-06 | -2.24 | 0.024971 | 0.59 |
| PRR14L | sCCA2 | 372 | 3 | lasso | 0.099 | 1.3e-08 | -2.26 | 0.023591 | 0.59 |
| ZNF546 | sCCA1 | 399 | 1 | top1 | 0.034 | 0.00074 | 2.27 | 0.023426 | 0.59 |
| MYO5A | sCCA2 | 437 | 1 | top1 | 0.19 | 1.6e-15 | 2.26 | 0.02386 | 0.59 |
| CEP290 | sCCA1 | 290 | 11 | lasso | 0.45 | 9.2e-42 | -2.25 | 0.024345 | 0.59 |
| TRIQK | sCCA2 | 364 | 24 | enet | 0.036 | 0.00051 | 2.26 | 0.023619 | 0.59 |
| MAP10 | sCCA1 | 619 | 11 | lasso | 0.22 | 2e-18 | -2.26 | 0.0241 | 0.59 |
| ANKRD39 | sCCA1 | 169 | 27 | enet | 0.074 | 7.9e-07 | -2.24 | 0.025 | 0.59 |
| PEX26 | sCCA1 | 338 | 4 | lasso | 0.13 | 3.8e-11 | 2.26 | 0.02413 | 0.59 |
| MTMR9LP | sCCA2 | 202 | 1 | top1 | -0.0013 | 0.43 | -2.25 | 0.02429 | 0.59 |
| BTBD19 | sCCA1 | 338 | 50 | enet | 0.3 | 3.2e-25 | 2.26 | 0.0236 | 0.59 |
| BTBD19 | sCCA3 | 338 | 3 | lasso | 0.086 | 1.2e-07 | 2.26 | 0.0239 | 0.59 |
| RP5-1142A6.2 | sCCA1 | 401 | 26 | enet | 0.052 | 3.2e-05 | -2.26 | 0.02371 | 0.59 |
| WASH4P | sCCA1 | 219 | 1 | top1 | 0.073 | 1e-06 | -2.26 | 0.02395 | 0.59 |
| RP11-288I21.1 | sCCA3 | 444 | 33 | enet | 0.13 | 9.3e-11 | -2.24 | 0.0249 | 0.59 |
| SEC24B-AS1 | sCCA3 | 381 | 23 | enet | -0.0019 | 0.52 | -2.24 | 0.02485 | 0.59 |
| NAIP | sCCA2 | 38 | 4 | lasso | 0.053 | 2.8e-05 | -2.25 | 0.024674 | 0.59 |
| FXYD1 | sCCA3 | 489 | 25 | enet | 0.027 | 0.0023 | -2.24 | 0.0248 | 0.59 |
| RP11-973H7.3 | sCCA3 | 372 | 12 | lasso | 0.23 | 5.7e-19 | 2.24 | 0.02501 | 0.59 |
| CTC-471F3.5 | sCCA2 | 393 | 1 | top1 | 0.078 | 4e-07 | -2.27 | 0.023426 | 0.59 |
| RP11-362K14.6 | sCCA3 | 416 | 1 | top1 | 0.066 | 3.4e-06 | -2.26 | 0.023517 | 0.59 |
| RP11-124D2.7 | sCCA3 | 482 | 1 | top1 | 0.024 | 0.0042 | 2.26 | 0.023871 | 0.59 |
| RP11-390E23.6 | sCCA1 | 349 | 10 | lasso | 0.41 | 3.6e-37 | 2.24 | 0.02494 | 0.59 |
| ORAI1 | sCCA2 | 375 | 1 | top1 | 0.14 | 3.5e-12 | 2.25 | 0.024671 | 0.59 |
| RP11-143K11.7 | sCCA2 | 558 | 9 | lasso | 0.0084 | 0.059 | 2.27 | 0.023454 | 0.59 |
| CTC-205M6.1 | sCCA3 | 377 | 18 | lasso | 0.45 | 4.6e-41 | 2.25 | 0.024148 | 0.59 |
| PNKP | sCCA2 | 345 | 5 | lasso | 0.3 | 4.2e-25 | -2.19 | 0.028528 | 0.6 |
| USP28 | sCCA2 | 583 | 43 | enet | 0.058 | 1.3e-05 | 2.21 | 0.027353 | 0.6 |
| CYBA | sCCA1 | 379 | 5 | lasso | 0.37 | 1.1e-32 | -2.2 | 0.02814 | 0.6 |
| AKR7A2 | sCCA1 | 488 | 12 | lasso | 0.35 | 7.2e-31 | -2.2 | 0.0278 | 0.6 |
| ROGDI | sCCA2 | 383 | 7 | enet | 0.019 | 0.0091 | 2.2 | 0.02799 | 0.6 |
| LIMS2 | sCCA1 | 364 | 32 | enet | 0.24 | 1.9e-20 | -2.2 | 0.0279 | 0.6 |
| NFATC3 | sCCA1 | 276 | 9 | lasso | 0.42 | 3.1e-38 | 2.19 | 0.02845 | 0.6 |
| ANO8 | sCCA1 | 437 | 33 | enet | 0.13 | 1.2e-10 | -2.2 | 0.027869 | 0.6 |
| UBE2K | sCCA3 | 363 | 1 | top1 | 0.14 | 7.8e-12 | -2.22 | 0.02636 | 0.6 |
| REXO1 | sCCA3 | 382 | 5 | lasso | 0.065 | 3.6e-06 | 2.2 | 0.0277 | 0.6 |
| GSK3B | sCCA2 | 453 | 1 | top1 | 0.13 | 6e-11 | -2.2 | 0.02754 | 0.6 |
| GSK3B | sCCA3 | 453 | 1 | top1 | 0.076 | 5.8e-07 | 2.2 | 0.02754 | 0.6 |
| CTTN | sCCA2 | 424 | 12 | lasso | 0.14 | 9.1e-12 | -2.21 | 0.027324 | 0.6 |
| ZC3HC1 | sCCA2 | 388 | 3 | lasso | 0.015 | 0.018 | -2.23 | 0.025739 | 0.6 |
| COMT | sCCA3 | 565 | 55 | enet | 0.18 | 1e-14 | 2.24 | 0.02527 | 0.6 |
| SRRD | sCCA3 | 779 | 6 | lasso | 0.066 | 3.3e-06 | -2.23 | 0.02594 | 0.6 |
| RAB36 | sCCA2 | 393 | 9 | enet | 0.22 | 2.7e-18 | 2.2 | 0.027899 | 0.6 |
| LMF2 | sCCA1 | 329 | 7 | lasso | 0.25 | 2.9e-21 | -2.23 | 0.02589 | 0.6 |
| CHADL | sCCA3 | 249 | 7 | enet | 0.057 | 1.4e-05 | -2.21 | 0.02726 | 0.6 |
| TRIB3 | sCCA1 | 468 | 6 | lasso | 0.51 | 1.7e-49 | 2.2 | 0.02777 | 0.6 |
| SIRPB1 | sCCA2 | 627 | 28 | enet | 0.84 | 1.5e-122 | -2.23 | 0.025694 | 0.6 |
| CAB39L | sCCA2 | 403 | 38 | enet | 0.16 | 3.1e-13 | -2.19 | 0.0282 | 0.6 |
| ACD | sCCA2 | 235 | 12 | enet | 0.05 | 4.9e-05 | -2.21 | 0.02701 | 0.6 |
| SLC7A6OS | sCCA1 | 316 | 1 | top1 | 0.1 | 6.6e-09 | -2.23 | 0.02564 | 0.6 |
| ACSBG1 | sCCA3 | 407 | 8 | lasso | 0.0096 | 0.048 | 2.22 | 0.02632 | 0.6 |
| TUBB4A | sCCA1 | 435 | 1 | top1 | 0.055 | 2.2e-05 | -2.22 | 0.026728 | 0.6 |
| FAM32A | sCCA2 | 478 | 25 | enet | 0.03 | 0.0014 | 2.23 | 0.026078 | 0.6 |
| TFPI2 | sCCA1 | 376 | 20 | enet | 0.051 | 4.3e-05 | 2.19 | 0.02852 | 0.6 |
| ACTR3C | sCCA3 | 350 | 15 | enet | 0.17 | 2.7e-14 | -2.2 | 0.027763 | 0.6 |
| ABCA2 | sCCA1 | 364 | 3 | lasso | 0.16 | 4.6e-13 | -2.24 | 0.02534 | 0.6 |
| ACTA2 | sCCA2 | 571 | 11 | enet | 0.045 | 0.00011 | -2.23 | 0.0255 | 0.6 |
| GBF1 | sCCA2 | 298 | 25 | enet | 0.017 | 0.014 | 2.21 | 0.027 | 0.6 |
| BLMH | sCCA2 | 301 | 1 | top1 | 0.034 | 0.00076 | 2.23 | 0.025768 | 0.6 |
| ELF2 | sCCA1 | 380 | 1 | top1 | 0.11 | 4.1e-09 | -2.2 | 0.02813 | 0.6 |
| EHD1 | sCCA1 | 331 | 19 | enet | 0.072 | 1.2e-06 | -2.2 | 0.028145 | 0.6 |
| SLC35F2 | sCCA3 | 401 | 34 | enet | 0.15 | 6.9e-13 | 2.21 | 0.0269 | 0.6 |
| NDUFS8 | sCCA1 | 269 | 1 | top1 | 0.49 | 2.8e-46 | 2.22 | 0.026355 | 0.6 |
| CD83 | sCCA1 | 489 | 1 | top1 | 0.28 | 2.9e-23 | 2.2 | 0.02753 | 0.6 |
| GOLPH3 | sCCA3 | 547 | 4 | lasso | 0.011 | 0.035 | 2.2 | 0.027821 | 0.6 |
| HMGXB3 | sCCA3 | 571 | 1 | top1 | 0.18 | 3.8e-15 | -2.21 | 0.026972 | 0.6 |
| ACVR2B | sCCA2 | 452 | 45 | enet | 0.041 | 0.00021 | 2.2 | 0.027732 | 0.6 |
| EIF4G1 | sCCA1 | 522 | 7 | lasso | 0.063 | 5.6e-06 | 2.2 | 0.0279 | 0.6 |
| STAT1 | sCCA2 | 355 | 31 | enet | 0.0033 | 0.16 | -2.2 | 0.0275 | 0.6 |
| CHST10 | sCCA1 | 485 | 1 | top1 | 0.35 | 1.1e-30 | -2.24 | 0.0253 | 0.6 |
| STK16 | sCCA2 | 393 | 34 | enet | 0.033 | 0.00079 | -2.21 | 0.0272 | 0.6 |
| WIPF1 | sCCA3 | 330 | 14 | lasso | 0.15 | 1.8e-12 | -2.21 | 0.027 | 0.6 |
| FARSB | sCCA2 | 444 | 1 | top1 | 0.047 | 7.8e-05 | 2.23 | 0.0258 | 0.6 |
| DNAJC6 | sCCA2 | 438 | 39 | enet | 0.14 | 5.3e-12 | 2.2 | 0.027904 | 0.6 |
| RGS2 | sCCA1 | 358 | 27 | enet | 0.28 | 2.3e-23 | 2.2 | 0.0276 | 0.6 |
| YIPF4 | sCCA3 | 317 | 4 | lasso | 0.15 | 3.4e-12 | 2.23 | 0.0257 | 0.6 |
| YIPF4 | sCCA1 | 317 | 9 | lasso | 0.15 | 2.3e-12 | 2.22 | 0.0263 | 0.6 |
| WBP4 | sCCA1 | 305 | 9 | lasso | 0.46 | 2.5e-42 | 2.19 | 0.02834 | 0.6 |
| ELF1 | sCCA1 | 305 | 12 | enet | 0.05 | 5.2e-05 | 2.2 | 0.02805 | 0.6 |
| IFT81 | sCCA1 | 261 | 1 | top1 | 0.23 | 6.1e-19 | -2.2 | 0.027682 | 0.6 |
| DDX54 | sCCA2 | 487 | 15 | enet | 0.064 | 4.6e-06 | 2.22 | 0.026398 | 0.6 |
| PLA2G12A | sCCA1 | 397 | 1 | top1 | 0.5 | 5.7e-48 | -2.22 | 0.02619 | 0.6 |
| PLA2G12A | sCCA2 | 397 | 1 | top1 | 0.32 | 3e-27 | 2.22 | 0.02619 | 0.6 |
| RIOK1 | sCCA1 | 604 | 9 | lasso | 0.46 | 3.6e-42 | 2.23 | 0.02575 | 0.6 |
| CNOT1 | sCCA2 | 469 | 22 | enet | 0.11 | 2.3e-09 | 2.23 | 0.02553 | 0.6 |
| TPST2 | sCCA3 | 848 | 1 | top1 | 0.11 | 3.4e-09 | 2.23 | 0.02595 | 0.6 |
| BCL2L2 | sCCA1 | 475 | 11 | lasso | 0.21 | 2.4e-17 | 2.21 | 0.02722 | 0.6 |
| DTD2 | sCCA2 | 322 | 8 | lasso | 0.35 | 1.5e-30 | -2.2 | 0.0276 | 0.6 |
| ZCCHC9 | sCCA3 | 465 | 35 | enet | 0.11 | 1.7e-09 | -2.2 | 0.028033 | 0.6 |
| RAF1 | sCCA2 | 510 | 1 | top1 | 0.14 | 8e-12 | -2.23 | 0.025812 | 0.6 |
| RFC3 | sCCA1 | 474 | 23 | enet | 0.093 | 3.1e-08 | 2.21 | 0.02699 | 0.6 |
| MRPS36 | sCCA2 | 290 | 34 | enet | 0.096 | 1.8e-08 | 2.24 | 0.025277 | 0.6 |
| PDGFRA | sCCA1 | 409 | 23 | enet | 0.064 | 4.6e-06 | 2.22 | 0.0266 | 0.6 |
| RAB11FIP5 | sCCA3 | 267 | 21 | enet | 0.047 | 8e-05 | 2.19 | 0.0283 | 0.6 |
| TGFBRAP1 | sCCA1 | 572 | 6 | lasso | 0.16 | 2e-13 | 2.22 | 0.0261 | 0.6 |
| GTF2A2 | sCCA2 | 521 | 3 | lasso | 0.069 | 2.1e-06 | 2.2 | 0.02807 | 0.6 |
| CLTC | sCCA2 | 312 | 9 | enet | 0.021 | 0.0068 | 2.23 | 0.025463 | 0.6 |
| CELA2A | sCCA1 | 513 | 1 | top1 | 0.068 | 2.4e-06 | 2.23 | 0.026 | 0.6 |
| LYST | sCCA2 | 448 | 15 | enet | 0.094 | 2.8e-08 | 2.23 | 0.025925 | 0.6 |
| ASXL2 | sCCA2 | 283 | 19 | lasso | 0.042 | 0.00019 | -2.24 | 0.0252 | 0.6 |
| SLC26A1 | sCCA2 | 399 | 27 | enet | 0.76 | 3.5e-96 | -2.22 | 0.02673 | 0.6 |
| LHFPL2 | sCCA1 | 449 | 18 | enet | 0.16 | 3e-13 | 2.19 | 0.028373 | 0.6 |
| SYBU | sCCA2 | 343 | 12 | enet | 0.048 | 6.8e-05 | -2.22 | 0.02631 | 0.6 |
| C10orf11 | sCCA1 | 381 | 1 | top1 | 0.65 | 4.5e-71 | 2.21 | 0.0271 | 0.6 |
| PIP4K2A | sCCA3 | 345 | 2 | lasso | 0.23 | 5.6e-19 | -2.21 | 0.0274 | 0.6 |
| ME3 | sCCA3 | 583 | 34 | enet | 0.21 | 7.9e-18 | -2.23 | 0.02576 | 0.6 |
| PSTPIP2 | sCCA3 | 511 | 1 | top1 | 0.048 | 6.3e-05 | -2.21 | 0.0273 | 0.6 |
| UHMK1 | sCCA1 | 657 | 2 | lasso | 0.2 | 6.4e-17 | 2.22 | 0.0266 | 0.6 |
| TMEM251 | sCCA1 | 383 | 3 | lasso | 0.051 | 4.1e-05 | 2.2 | 0.02758 | 0.6 |
| TTC39B | sCCA2 | 465 | 1 | top1 | 0.052 | 3.7e-05 | 2.2 | 0.028151 | 0.6 |
| N6AMT1 | sCCA1 | 470 | 15 | lasso | 0.4 | 3.5e-36 | -2.23 | 0.025445 | 0.6 |
| GATAD1 | sCCA1 | 307 | 44 | enet | 0.53 | 8.4e-52 | -2.22 | 0.02645 | 0.6 |
| ARHGAP27 | sCCA3 | 183 | 4 | lasso | 0.048 | 6.6e-05 | -2.24 | 0.025146 | 0.6 |
| VPS11 | sCCA3 | 380 | 20 | enet | 0.51 | 1e-49 | -2.21 | 0.0271 | 0.6 |
| FBXO27 | sCCA2 | 402 | 21 | enet | 0.4 | 9.3e-36 | -2.24 | 0.025347 | 0.6 |
| BPNT1 | sCCA1 | 400 | 1 | top1 | 0.063 | 5.3e-06 | -2.2 | 0.0277 | 0.6 |
| C1orf115 | sCCA1 | 402 | 20 | enet | 0.4 | 6.4e-36 | -2.21 | 0.0274 | 0.6 |
| KIAA1841 | sCCA1 | 322 | 1 | top1 | 0.027 | 0.0023 | -2.24 | 0.0254 | 0.6 |
| ERCC3 | sCCA2 | 454 | 6 | lasso | 0.61 | 8.5e-64 | -2.23 | 0.0255 | 0.6 |
| BSN | sCCA2 | 302 | 18 | enet | 0.036 | 0.00047 | 2.21 | 0.027368 | 0.6 |
| ANAPC10 | sCCA3 | 250 | 21 | enet | 0.036 | 5e-04 | -2.24 | 0.0253 | 0.6 |
| PSIP1 | sCCA1 | 432 | 1 | top1 | 0.046 | 1e-04 | 2.2 | 0.02807 | 0.6 |
| NIPSNAP3B | sCCA1 | 605 | 30 | enet | 0.2 | 1.1e-16 | -2.24 | 0.02537 | 0.6 |
| ILK | sCCA2 | 632 | 1 | top1 | 0.26 | 6.4e-22 | -2.21 | 0.027432 | 0.6 |
| CATSPER2 | sCCA2 | 257 | 38 | enet | 0.13 | 2.6e-11 | 2.22 | 0.0264 | 0.6 |
| FAM111A | sCCA1 | 339 | 1 | top1 | 0.29 | 2.4e-24 | -2.21 | 0.027362 | 0.6 |
| FAM111A | sCCA2 | 339 | 1 | top1 | 0.21 | 3e-17 | -2.21 | 0.027362 | 0.6 |
| PIP4K2C | sCCA2 | 337 | 1 | top1 | 0.097 | 1.5e-08 | 2.22 | 0.026144 | 0.6 |
| GATAD2A | sCCA1 | 342 | 20 | enet | 0.18 | 4.1e-15 | 2.22 | 0.026685 | 0.6 |
| SETD5 | sCCA2 | 534 | 5 | lasso | 0.083 | 1.8e-07 | -2.21 | 0.02745 | 0.6 |
| PPIP5K1 | sCCA3 | 256 | 31 | enet | 0.019 | 0.0089 | -2.22 | 0.02624 | 0.6 |
| IL12A | sCCA2 | 347 | 15 | lasso | 0.049 | 5.8e-05 | -2.22 | 0.026481 | 0.6 |
| MFSD7 | sCCA3 | 383 | 24 | enet | 0.2 | 2.9e-16 | -2.23 | 0.02557 | 0.6 |
| NPIPB3 | sCCA1 | 205 | 22 | enet | 0.14 | 9.8e-12 | -2.21 | 0.02732 | 0.6 |
| MT1E | sCCA2 | 521 | 40 | enet | 0.38 | 4.2e-33 | 2.2 | 0.02758 | 0.6 |
| ZNF570 | sCCA1 | 279 | 1 | top1 | 0.048 | 6.3e-05 | 2.22 | 0.026692 | 0.6 |
| QARS | sCCA3 | 254 | 6 | lasso | 0.047 | 7.9e-05 | 2.21 | 0.027026 | 0.6 |
| MCRIP2 | sCCA3 | 461 | 17 | enet | 0.087 | 8.5e-08 | -2.21 | 0.02694 | 0.6 |
| MYOZ2 | sCCA2 | 436 | 14 | enet | 0.15 | 5.8e-13 | -2.24 | 0.02529 | 0.6 |
| LCLAT1 | sCCA3 | 486 | 8 | lasso | 0.46 | 1.1e-42 | -2.23 | 0.0257 | 0.6 |
| LCLAT1 | sCCA2 | 486 | 30 | enet | 0.34 | 2.4e-29 | 2.2 | 0.0281 | 0.6 |
| RAPH1 | sCCA1 | 353 | 6 | lasso | 0.025 | 0.003 | 2.21 | 0.027 | 0.6 |
| LIPT2 | sCCA3 | 413 | 7 | lasso | 0.049 | 5.9e-05 | 2.23 | 0.02603 | 0.6 |
| BAIAP2 | sCCA2 | 445 | 5 | lasso | 0.13 | 2.4e-11 | -2.2 | 0.028077 | 0.6 |
| SLC35A4 | sCCA1 | 328 | 22 | enet | 0.089 | 6.8e-08 | 2.22 | 0.026099 | 0.6 |
| YES1 | sCCA1 | 598 | 13 | lasso | 0.13 | 1e-10 | 2.23 | 0.02543 | 0.6 |
| GPX2 | sCCA1 | 485 | 21 | enet | 0.21 | 9.1e-18 | -2.2 | 0.02807 | 0.6 |
| PAWR | sCCA3 | 278 | 1 | top1 | 0.05 | 4.9e-05 | -2.2 | 0.0277 | 0.6 |
| ZC3H12D | sCCA3 | 439 | 24 | enet | 0.066 | 3.5e-06 | -2.22 | 0.02611 | 0.6 |
| DTX3 | sCCA2 | 332 | 20 | enet | 0.013 | 0.024 | -2.21 | 0.027204 | 0.6 |
| TMEM52 | sCCA2 | 328 | 13 | lasso | 0.13 | 1.1e-10 | -2.19 | 0.028278 | 0.6 |
| ZFP3 | sCCA1 | 441 | 8 | lasso | 0.047 | 7.9e-05 | -2.22 | 0.026275 | 0.6 |
| ZNF443 | sCCA3 | 280 | 4 | lasso | 0.23 | 1.9e-19 | -2.2 | 0.0276 | 0.6 |
| CELF2-AS1 | sCCA1 | 585 | 13 | enet | 0.033 | 0.00079 | 2.22 | 0.0261 | 0.6 |
| ZNRF3 | sCCA2 | 340 | 1 | top1 | 0.057 | 1.4e-05 | -2.2 | 0.02806 | 0.6 |
| RP5-821D11.7 | sCCA2 | 320 | 10 | lasso | 0.17 | 2.3e-14 | -2.21 | 0.027116 | 0.6 |
| KNTC1 | sCCA1 | 311 | 6 | lasso | 0.22 | 2.8e-18 | 2.22 | 0.026132 | 0.6 |
| SPDYE12P | sCCA2 | 87 | 32 | enet | 0.36 | 4.4e-31 | -2.22 | 0.026737 | 0.6 |
| SNAI3 | sCCA1 | 387 | 13 | enet | 0.31 | 7.1e-27 | 2.21 | 0.02694 | 0.6 |
| NCCRP1 | sCCA2 | 419 | 1 | top1 | 0.076 | 5.9e-07 | 2.24 | 0.025315 | 0.6 |
| ARID2 | sCCA3 | 389 | 1 | top1 | 0.053 | 2.9e-05 | 2.23 | 0.0258 | 0.6 |
| ZNF573 | sCCA2 | 325 | 10 | lasso | 0.27 | 2.4e-22 | -2.23 | 0.02562 | 0.6 |
| SUPT3H | sCCA1 | 362 | 69 | enet | 0.44 | 2.9e-40 | 2.21 | 0.02706 | 0.6 |
| PPTC7 | sCCA2 | 210 | 19 | enet | 0.037 | 4e-04 | 2.2 | 0.028117 | 0.6 |
| ZNF615 | sCCA1 | 600 | 1 | top1 | 0.16 | 2e-13 | 2.19 | 0.028285 | 0.6 |
| PSAP | sCCA1 | 580 | 14 | lasso | 0.15 | 1.1e-12 | -2.24 | 0.0252 | 0.6 |
| ENTPD7 | sCCA2 | 445 | 44 | enet | 0.056 | 1.9e-05 | 2.23 | 0.0259 | 0.6 |
| CARD11 | sCCA3 | 599 | 47 | enet | 0.091 | 4.7e-08 | 2.23 | 0.02561 | 0.6 |
| MYL4 | sCCA3 | 337 | 1 | top1 | 0.05 | 4.6e-05 | -2.21 | 0.027155 | 0.6 |
| WWP2 | sCCA1 | 253 | 21 | enet | 0.33 | 4.3e-28 | 2.21 | 0.02701 | 0.6 |
| ZNF568 | sCCA3 | 283 | 39 | enet | 0.29 | 3e-24 | 2.19 | 0.0285 | 0.6 |
| TCEA3 | sCCA2 | 313 | 41 | enet | 0.14 | 8.9e-12 | 2.22 | 0.026528 | 0.6 |
| LINC01291 | sCCA2 | 456 | 89 | enet | 0.71 | 1.9e-84 | -2.22 | 0.0263 | 0.6 |
| ZNF316 | sCCA2 | 318 | 32 | enet | 0.12 | 1.8e-10 | 2.2 | 0.027989 | 0.6 |
| RPS7P11 | sCCA2 | 189 | 1 | top1 | 0.032 | 0.00094 | 2.21 | 0.027422 | 0.6 |
| RPSAP54 | sCCA3 | 445 | 1 | top1 | 0.032 | 0.00099 | -2.21 | 0.0274 | 0.6 |
| RPL17P50 | sCCA1 | 237 | 11 | enet | 0.068 | 2.2e-06 | -2.24 | 0.0252 | 0.6 |
| RP11-472B18.1 | sCCA2 | 366 | 1 | top1 | 0.22 | 1e-18 | -2.23 | 0.02549 | 0.6 |
| SP2-AS1 | sCCA1 | 374 | 16 | lasso | 0.048 | 6.9e-05 | 2.22 | 0.026691 | 0.6 |
| HLA-W | sCCA2 | 39 | 34 | enet | 0.13 | 6.4e-11 | -2.22 | 0.02625 | 0.6 |
| RP4-756G23.5 | sCCA1 | 253 | 21 | enet | 0.41 | 4.5e-37 | -2.21 | 0.02705 | 0.6 |
| FAM21FP | sCCA1 | 222 | 35 | enet | 0.47 | 4e-44 | -2.24 | 0.0253 | 0.6 |
| COX19 | sCCA2 | 350 | 34 | enet | 0.12 | 5.3e-10 | -2.21 | 0.027194 | 0.6 |
| H2AFJ | sCCA3 | 367 | 1 | top1 | 0.083 | 1.7e-07 | -2.23 | 0.0256 | 0.6 |
| CDK11B | sCCA2 | 308 | 19 | enet | 0.31 | 2.4e-26 | 2.22 | 0.026571 | 0.6 |
| LRRC37BP1 | sCCA3 | 292 | 17 | enet | 0.097 | 1.8e-08 | -2.23 | 0.025678 | 0.6 |
| NTAN1P2 | sCCA1 | 445 | 15 | lasso | 0.26 | 3.3e-22 | -2.22 | 0.0264 | 0.6 |
| CTD-2284J15.1 | sCCA1 | 403 | 1 | top1 | 0.11 | 3.2e-09 | -2.23 | 0.0257 | 0.6 |
| RP5-901A4.1 | sCCA1 | 269 | 1 | top1 | 0.53 | 6.9e-52 | 2.22 | 0.026355 | 0.6 |
| TRAPPC2B | sCCA2 | 505 | 11 | lasso | 0.14 | 4.9e-12 | 2.21 | 0.027035 | 0.6 |
| RP11-16E12.1 | sCCA1 | 447 | 1 | top1 | 0.073 | 1.1e-06 | 2.22 | 0.02614 | 0.6 |
| RP11-174G6.5 | sCCA1 | 370 | 20 | enet | 0.12 | 3e-10 | 2.19 | 0.028502 | 0.6 |
| PNLIPRP2 | sCCA1 | 469 | 11 | enet | 0.49 | 3.2e-46 | -2.22 | 0.0267 | 0.6 |
| CBX3P2 | sCCA2 | 515 | 1 | top1 | 0.072 | 1.1e-06 | -2.22 | 0.0262 | 0.6 |
| RP11-35G9.3 | sCCA2 | 546 | 40 | enet | 0.056 | 1.7e-05 | -2.23 | 0.0255 | 0.6 |
| AC137932.4 | sCCA3 | 393 | 22 | enet | 0.074 | 8e-07 | -2.22 | 0.02613 | 0.6 |
| GAS5-AS1 | sCCA3 | 268 | 5 | lasso | 0.1 | 8e-09 | 2.23 | 0.026 | 0.6 |
| RP11-245J9.5 | sCCA1 | 576 | 29 | enet | 0.12 | 1.5e-10 | -2.22 | 0.0266 | 0.6 |
| RP4-635E18.8 | sCCA3 | 374 | 1 | top1 | 0.038 | 0.00038 | -2.22 | 0.0261 | 0.6 |
| RP11-574K11.29 | sCCA3 | 254 | 1 | top1 | 0.034 | 0.00074 | 2.2 | 0.0281 | 0.6 |
| RP1-286D6.5 | sCCA2 | 544 | 3 | lasso | 0.071 | 1.5e-06 | 2.2 | 0.027889 | 0.6 |
| RP11-390E23.6 | sCCA2 | 349 | 47 | enet | 0.36 | 1.7e-31 | 2.21 | 0.026771 | 0.6 |
| RP11-563N4.1 | sCCA1 | 270 | 18 | enet | 0.27 | 5e-23 | 2.19 | 0.0283 | 0.6 |
| CTB-119C2.1 | sCCA1 | 525 | 6 | lasso | 0.25 | 1.5e-20 | -2.23 | 0.02593 | 0.6 |
| SNORA44 | sCCA3 | 267 | 9 | lasso | 0.17 | 9.1e-14 | 2.21 | 0.0272 | 0.6 |
| RP5-908M14.9 | sCCA1 | 507 | 8 | lasso | 0.66 | 1.2e-72 | -2.23 | 0.02592 | 0.6 |
| RP5-908M14.9 | sCCA3 | 507 | 5 | lasso | 0.48 | 4.5e-45 | 2.23 | 0.02595 | 0.6 |
| RP5-908M14.9 | sCCA2 | 507 | 6 | lasso | 0.61 | 7.4e-65 | -2.22 | 0.026444 | 0.6 |
| RP5-908M14.10 | sCCA2 | 522 | 10 | lasso | 0.13 | 2.7e-11 | 2.21 | 0.027437 | 0.6 |
| RP11-582E3.6 | sCCA3 | 523 | 36 | enet | 0.62 | 1.7e-65 | -2.23 | 0.0259 | 0.6 |
| RP11-582E3.6 | sCCA1 | 523 | 34 | enet | 0.61 | 1e-63 | 2.2 | 0.027781 | 0.6 |
| AL133243.2 | sCCA1 | 380 | 7 | lasso | 0.15 | 2e-12 | -2.2 | 0.0278 | 0.6 |
| RP11-380B4.3 | sCCA1 | 558 | 34 | enet | 0.22 | 4.5e-18 | -2.21 | 0.02686 | 0.6 |
| RP11-45M22.2 | sCCA1 | 371 | 11 | lasso | 0.064 | 4.8e-06 | -2.24 | 0.025287 | 0.6 |
| AL022393.9 | sCCA1 | 461 | 66 | enet | 0.52 | 6.8e-51 | 2.2 | 0.02779 | 0.6 |
| IFRD1 | sCCA1 | 403 | 5 | lasso | 0.55 | 2.6e-54 | 2.13 | 0.03335 | 0.61 |
| CELSR3 | sCCA3 | 255 | 1 | top1 | 0.043 | 0.00016 | 2.18 | 0.02941 | 0.61 |
| MMP25 | sCCA1 | 369 | 7 | lasso | 0.24 | 1.1e-19 | -2.12 | 0.03394 | 0.61 |
| VTA1 | sCCA3 | 336 | 9 | lasso | 0.05 | 4.9e-05 | -2.14 | 0.03242 | 0.61 |
| LTF | sCCA1 | 396 | 1 | top1 | 0.18 | 1.2e-14 | -2.17 | 0.0302 | 0.61 |
| ALOX5 | sCCA3 | 346 | 17 | lasso | 0.17 | 3.3e-14 | -2.12 | 0.0336 | 0.61 |
| SLC25A39 | sCCA3 | 340 | 47 | enet | 0.52 | 6.7e-50 | -2.16 | 0.031117 | 0.61 |
| RFC1 | sCCA1 | 419 | 1 | top1 | 0.19 | 8.5e-16 | 2.13 | 0.03336 | 0.61 |
| AKR7A2 | sCCA2 | 488 | 4 | lasso | 0.28 | 2.8e-23 | -2.11 | 0.034767 | 0.61 |
| THRAP3 | sCCA3 | 343 | 1 | top1 | 0.02 | 0.0076 | -2.11 | 0.0352 | 0.61 |
| PPP1R12A | sCCA3 | 288 | 1 | top1 | 0.017 | 0.013 | 2.13 | 0.0328 | 0.61 |
| PPP2R5A | sCCA2 | 445 | 10 | lasso | 0.057 | 1.5e-05 | -2.16 | 0.031 | 0.61 |
| DHX29 | sCCA2 | 386 | 14 | enet | 0.052 | 3.2e-05 | -2.17 | 0.030237 | 0.61 |
| PRR11 | sCCA1 | 291 | 1 | top1 | 0.17 | 5.9e-14 | -2.13 | 0.033321 | 0.61 |
| SRBD1 | sCCA1 | 673 | 45 | enet | 0.43 | 1.9e-39 | 2.16 | 0.0308 | 0.61 |
| IFT80 | sCCA1 | 374 | 9 | lasso | 0.18 | 2.4e-15 | 2.11 | 0.0345 | 0.61 |
| IKBKAP | sCCA2 | 609 | 9 | lasso | 0.34 | 2.4e-29 | 2.1 | 0.035388 | 0.61 |
| NFATC3 | sCCA3 | 276 | 8 | lasso | 0.41 | 1.5e-36 | -2.16 | 0.03084 | 0.61 |
| TRNT1 | sCCA3 | 811 | 6 | lasso | 0.25 | 2.4e-21 | -2.18 | 0.029345 | 0.61 |
| TMEM38A | sCCA2 | 379 | 9 | lasso | 0.17 | 1.6e-14 | -2.19 | 0.028817 | 0.61 |
| ACAT1 | sCCA2 | 361 | 4 | lasso | 0.42 | 1.2e-37 | 2.1 | 0.035501 | 0.61 |
| KLHL42 | sCCA3 | 527 | 37 | enet | 0.017 | 0.013 | -2.15 | 0.0312 | 0.61 |
| OAS1 | sCCA3 | 416 | 1 | top1 | 0.056 | 1.8e-05 | 2.17 | 0.03 | 0.61 |
| LTBP4 | sCCA2 | 413 | 1 | top1 | 0.013 | 0.024 | -2.12 | 0.033786 | 0.61 |
| ALKBH5 | sCCA3 | 304 | 40 | enet | 0.44 | 1.4e-40 | 2.12 | 0.033985 | 0.61 |
| PSME1 | sCCA1 | 511 | 21 | enet | 0.033 | 0.00081 | -2.17 | 0.02967 | 0.61 |
| MYH7 | sCCA2 | 457 | 14 | enet | 0.0068 | 0.08 | 2.18 | 0.0292 | 0.61 |
| TRPM7 | sCCA1 | 347 | 1 | top1 | 0.07 | 1.7e-06 | 2.19 | 0.02882 | 0.61 |
| XYLB | sCCA1 | 438 | 10 | lasso | 0.14 | 2e-11 | 2.17 | 0.0297 | 0.61 |
| RAB18 | sCCA1 | 441 | 1 | top1 | 0.19 | 1.3e-15 | 2.11 | 0.035 | 0.61 |
| CEP170B | sCCA3 | 341 | 25 | enet | 0.37 | 5.9e-33 | 2.12 | 0.033743 | 0.61 |
| RASSF7 | sCCA3 | 435 | 7 | lasso | 0.072 | 1.1e-06 | 2.16 | 0.03081 | 0.61 |
| SMARCB1 | sCCA2 | 464 | 7 | lasso | 0.54 | 5.9e-53 | 2.11 | 0.034848 | 0.61 |
| DDT | sCCA2 | 416 | 7 | lasso | 0.54 | 1.1e-53 | 2.13 | 0.033165 | 0.61 |
| RAB36 | sCCA1 | 393 | 15 | enet | 0.31 | 1.6e-26 | 2.18 | 0.02953 | 0.61 |
| RAB36 | sCCA3 | 393 | 17 | enet | 0.29 | 2.9e-24 | -2.16 | 0.03116 | 0.61 |
| LMF2 | sCCA2 | 329 | 42 | enet | 0.22 | 2.2e-18 | -2.11 | 0.034693 | 0.61 |
| PDGFB | sCCA2 | 402 | 1 | top1 | 0.038 | 0.00037 | 2.14 | 0.032679 | 0.61 |
| TXN2 | sCCA3 | 623 | 53 | enet | 0.17 | 5.2e-14 | 2.15 | 0.0317 | 0.61 |
| RBX1 | sCCA1 | 273 | 1 | top1 | 0.083 | 1.8e-07 | 2.14 | 0.03276 | 0.61 |
| CHADL | sCCA1 | 249 | 1 | top1 | 0.058 | 1.3e-05 | 2.14 | 0.03276 | 0.61 |
| TRPC4AP | sCCA3 | 341 | 13 | lasso | 0.26 | 1.5e-21 | -2.11 | 0.03482 | 0.61 |
| PRPF6 | sCCA2 | 372 | 62 | enet | 0.24 | 2.9e-20 | -2.11 | 0.035015 | 0.61 |
| RBFA | sCCA2 | 281 | 6 | lasso | 0.34 | 4.1e-29 | -2.17 | 0.0297 | 0.61 |
| KPNA3 | sCCA2 | 406 | 1 | top1 | 0.063 | 5.8e-06 | 2.17 | 0.03033 | 0.61 |
| CD276 | sCCA3 | 361 | 13 | enet | 0.025 | 0.0031 | -2.18 | 0.02936 | 0.61 |
| FZD3 | sCCA1 | 443 | 12 | lasso | 0.3 | 2.5e-25 | 2.12 | 0.0341 | 0.61 |
| CPQ | sCCA1 | 404 | 9 | lasso | 0.26 | 2e-21 | 2.17 | 0.0302 | 0.61 |
| CPQ | sCCA3 | 404 | 10 | lasso | 0.23 | 1.9e-19 | 2.11 | 0.03502 | 0.61 |
| PLAT | sCCA3 | 344 | 1 | top1 | 0.051 | 4.3e-05 | 2.15 | 0.03189 | 0.61 |
| SPAG1 | sCCA3 | 370 | 9 | lasso | 0.23 | 8.4e-19 | 2.18 | 0.02955 | 0.61 |
| KLHDC4 | sCCA2 | 468 | 9 | lasso | 0.25 | 3.6e-21 | 2.15 | 0.03154 | 0.61 |
| ARHGEF18 | sCCA3 | 473 | 23 | enet | 0.048 | 6.7e-05 | 2.13 | 0.0332 | 0.61 |
| FAM32A | sCCA1 | 478 | 19 | enet | 0.04 | 0.00025 | 2.15 | 0.031766 | 0.61 |
| MYH14 | sCCA3 | 358 | 6 | enet | 0.078 | 4.6e-07 | -2.15 | 0.0315 | 0.61 |
| RAB3D | sCCA2 | 389 | 33 | enet | 0.031 | 0.0013 | -2.16 | 0.031144 | 0.61 |
| PPP2R1A | sCCA1 | 547 | 1 | top1 | 0.28 | 9e-24 | 2.17 | 0.030209 | 0.61 |
| TNPO2 | sCCA2 | 295 | 37 | enet | 0.29 | 3.9e-24 | 2.11 | 0.035065 | 0.61 |
| ARRDC2 | sCCA2 | 427 | 11 | enet | 0.017 | 0.012 | -2.16 | 0.030813 | 0.61 |
| ELL | sCCA1 | 376 | 9 | lasso | 0.3 | 1.2e-25 | -2.18 | 0.029174 | 0.61 |
| SMG9 | sCCA1 | 427 | 5 | lasso | 0.57 | 9.3e-58 | -2.17 | 0.030171 | 0.61 |
| ADAP1 | sCCA1 | 348 | 40 | enet | 0.41 | 9.8e-37 | -2.17 | 0.03019 | 0.61 |
| ADAP1 | sCCA2 | 348 | 44 | enet | 0.43 | 1.5e-38 | -2.16 | 0.03047 | 0.61 |
| PRKAG2 | sCCA1 | 432 | 56 | enet | 0.05 | 4.8e-05 | 2.14 | 0.03261 | 0.61 |
| PPP3CB | sCCA2 | 246 | 1 | top1 | 0.048 | 6.9e-05 | 2.16 | 0.0311 | 0.61 |
| CPEB3 | sCCA1 | 305 | 1 | top1 | 0.1 | 7.8e-09 | -2.18 | 0.0293 | 0.61 |
| CUEDC2 | sCCA1 | 341 | 5 | lasso | 0.089 | 6.2e-08 | 2.17 | 0.0301 | 0.61 |
| SUFU | sCCA2 | 333 | 1 | top1 | 0.11 | 2.2e-09 | 2.18 | 0.0291 | 0.61 |
| GTPBP4 | sCCA3 | 498 | 9 | lasso | 0.074 | 9.1e-07 | -2.13 | 0.0334 | 0.61 |
| SHOC2 | sCCA2 | 509 | 39 | enet | 0.025 | 0.003 | 2.12 | 0.0341 | 0.61 |
| RAPGEFL1 | sCCA3 | 294 | 5 | lasso | 0.54 | 9.5e-53 | -2.11 | 0.034829 | 0.61 |
| C17orf75 | sCCA2 | 253 | 10 | enet | 0.032 | 0.001 | 2.1 | 0.035551 | 0.61 |
| WFS1 | sCCA1 | 673 | 1 | top1 | 0.53 | 2.1e-51 | -2.17 | 0.03032 | 0.61 |
| WFS1 | sCCA2 | 673 | 1 | top1 | 0.2 | 1.6e-16 | -2.17 | 0.03032 | 0.61 |
| TRIM3 | sCCA3 | 651 | 26 | enet | 0.17 | 7.2e-14 | 2.17 | 0.03037 | 0.61 |
| PPFIBP1 | sCCA2 | 497 | 8 | lasso | 0.11 | 1.1e-09 | 2.16 | 0.030524 | 0.61 |
| RSRC2 | sCCA1 | 312 | 1 | top1 | 0.13 | 2.7e-11 | -2.13 | 0.033132 | 0.61 |
| CYP27B1 | sCCA2 | 346 | 15 | enet | 0.01 | 0.043 | -2.15 | 0.031666 | 0.61 |
| CDK2AP1 | sCCA3 | 340 | 8 | lasso | 0.57 | 3.7e-57 | -2.12 | 0.034 | 0.61 |
| CDK2AP1 | sCCA2 | 340 | 18 | lasso | 0.39 | 6.9e-35 | -2.11 | 0.03527 | 0.61 |
| CD83 | sCCA2 | 489 | 7 | lasso | 0.16 | 2.7e-13 | 2.16 | 0.030671 | 0.61 |
| PHACTR2 | sCCA3 | 505 | 11 | lasso | 0.11 | 1.1e-09 | 2.17 | 0.03034 | 0.61 |
| GUCA1B | sCCA2 | 410 | 14 | enet | 0.32 | 2.1e-27 | -2.17 | 0.030225 | 0.61 |
| CUL9 | sCCA1 | 364 | 4 | lasso | 0.1 | 9.1e-09 | 2.16 | 0.03071 | 0.61 |
| HMGXB3 | sCCA1 | 571 | 35 | enet | 0.37 | 4.3e-32 | 2.17 | 0.029859 | 0.61 |
| SMC4 | sCCA2 | 374 | 17 | enet | 0.16 | 1.1e-13 | -2.11 | 0.035052 | 0.61 |
| KPNA1 | sCCA1 | 494 | 9 | lasso | 0.18 | 3.1e-15 | -2.13 | 0.0335 | 0.61 |
| SSR3 | sCCA1 | 397 | 26 | enet | 0.12 | 6.4e-10 | 2.16 | 0.0308 | 0.61 |
| PDCL3 | sCCA1 | 536 | 5 | lasso | 0.35 | 2.4e-30 | -2.17 | 0.03 | 0.61 |
| PLEKHB2 | sCCA2 | 197 | 6 | lasso | 0.03 | 0.0013 | -2.14 | 0.0327 | 0.61 |
| THADA | sCCA1 | 476 | 11 | lasso | 0.11 | 2.7e-09 | -2.15 | 0.0316 | 0.61 |
| MSH6 | sCCA2 | 360 | 7 | lasso | 0.33 | 9.9e-29 | -2.14 | 0.0322 | 0.61 |
| SCAMP3 | sCCA1 | 299 | 1 | top1 | 0.044 | 0.00013 | -2.18 | 0.0295 | 0.61 |
| MEF2D | sCCA3 | 430 | 18 | enet | 0.023 | 0.0048 | -2.16 | 0.0305 | 0.61 |
| RGS2 | sCCA3 | 358 | 38 | enet | 0.16 | 2.6e-13 | -2.12 | 0.0343 | 0.61 |
| PLEKHM2 | sCCA2 | 482 | 10 | lasso | 0.12 | 1.6e-10 | 2.13 | 0.032891 | 0.61 |
| TFAP2E | sCCA3 | 220 | 27 | enet | 0.065 | 3.8e-06 | 2.14 | 0.0326 | 0.61 |
| TMED5 | sCCA2 | 265 | 15 | enet | 0.065 | 4.2e-06 | -2.16 | 0.030763 | 0.61 |
| MARC2 | sCCA1 | 404 | 3 | lasso | 0.38 | 4.2e-33 | 2.11 | 0.0351 | 0.61 |
| MFSD1 | sCCA3 | 398 | 11 | lasso | 0.28 | 1.1e-23 | -2.18 | 0.029393 | 0.61 |
| MFSD1 | sCCA1 | 398 | 8 | lasso | 0.4 | 3e-35 | -2.16 | 0.0308 | 0.61 |
| LDAH | sCCA3 | 530 | 7 | enet | 0.47 | 1.7e-44 | 2.13 | 0.0331 | 0.61 |
| CNTRL | sCCA1 | 379 | 15 | lasso | 0.11 | 9.9e-10 | 2.12 | 0.03372 | 0.61 |
| PHF19 | sCCA1 | 356 | 5 | lasso | 0.073 | 1e-06 | -2.15 | 0.03134 | 0.61 |
| NEK6 | sCCA2 | 404 | 1 | top1 | 0.013 | 0.028 | -2.11 | 0.034485 | 0.61 |
| RBM18 | sCCA2 | 541 | 6 | lasso | 0.29 | 6.5e-25 | -2.18 | 0.029332 | 0.61 |
| AREL1 | sCCA1 | 342 | 16 | enet | 0.16 | 1.5e-13 | -2.13 | 0.03301 | 0.61 |
| ZNF706 | sCCA1 | 503 | 1 | top1 | 0.073 | 1.1e-06 | -2.17 | 0.0303 | 0.61 |
| POPDC2 | sCCA2 | 516 | 1 | top1 | 0.15 | 7.4e-13 | 2.17 | 0.030339 | 0.61 |
| HERPUD2 | sCCA1 | 542 | 4 | lasso | 0.34 | 3e-29 | -2.16 | 0.03094 | 0.61 |
| LRP1 | sCCA2 | 330 | 1 | top1 | 0.016 | 0.015 | 2.15 | 0.0316 | 0.61 |
| ATPAF1 | sCCA1 | 367 | 1 | top1 | 0.071 | 1.5e-06 | -2.11 | 0.0353 | 0.61 |
| G0S2 | sCCA3 | 496 | 1 | top1 | 0.02 | 0.0075 | -2.14 | 0.032 | 0.61 |
| CKS2 | sCCA3 | 497 | 42 | enet | 0.33 | 1.6e-28 | -2.1 | 0.03558 | 0.61 |
| SDC4 | sCCA1 | 491 | 26 | enet | 0.28 | 1.1e-23 | -2.1 | 0.03552 | 0.61 |
| NEURL2 | sCCA2 | 514 | 39 | enet | 0.071 | 1.4e-06 | -2.19 | 0.028754 | 0.61 |
| NEURL2 | sCCA3 | 514 | 41 | enet | 0.16 | 2.7e-13 | 2.14 | 0.03261 | 0.61 |
| RIOK1 | sCCA3 | 604 | 5 | lasso | 0.24 | 1.8e-20 | 2.14 | 0.03239 | 0.61 |
| RPP40 | sCCA3 | 636 | 24 | enet | 0.05 | 5e-05 | 2.17 | 0.03029 | 0.61 |
| C17orf53 | sCCA1 | 361 | 16 | enet | 0.014 | 0.022 | -2.15 | 0.031525 | 0.61 |
| OPRL1 | sCCA3 | 312 | 27 | enet | 0.58 | 8.1e-59 | 2.16 | 0.03097 | 0.61 |
| GZF1 | sCCA2 | 564 | 31 | enet | 0.1 | 6.9e-09 | -2.18 | 0.028989 | 0.61 |
| TICAM1 | sCCA3 | 427 | 54 | enet | 0.14 | 2e-11 | 2.18 | 0.0294 | 0.61 |
| GNG11 | sCCA1 | 395 | 3 | lasso | 0.22 | 3.1e-18 | -2.15 | 0.03119 | 0.61 |
| GNAI1 | sCCA1 | 424 | 8 | lasso | 0.17 | 1.5e-14 | 2.13 | 0.03346 | 0.61 |
| PEX1 | sCCA3 | 313 | 1 | top1 | 0.066 | 3.2e-06 | -2.14 | 0.032556 | 0.61 |
| SPECC1 | sCCA1 | 275 | 29 | enet | 0.18 | 1.2e-14 | -2.16 | 0.030895 | 0.61 |
| PSMG2 | sCCA1 | 375 | 13 | lasso | 0.38 | 5.3e-33 | 2.11 | 0.0345 | 0.61 |
| PSMG2 | sCCA3 | 375 | 14 | enet | 0.078 | 4.2e-07 | 2.1 | 0.0357 | 0.61 |
| LOXL1 | sCCA1 | 402 | 4 | lasso | 0.22 | 6.3e-18 | -2.11 | 0.03485 | 0.61 |
| MAP1S | sCCA2 | 429 | 1 | top1 | 0.044 | 0.00013 | 2.11 | 0.03517 | 0.61 |
| SAMD10 | sCCA3 | 372 | 20 | enet | 0.011 | 0.035 | -2.17 | 0.03032 | 0.61 |
| LSP1 | sCCA2 | 424 | 1 | top1 | 0.092 | 4e-08 | 2.18 | 0.029465 | 0.61 |
| PAK4 | sCCA3 | 412 | 43 | enet | 0.51 | 5.4e-49 | -2.13 | 0.0332 | 0.61 |
| C1orf159 | sCCA3 | 218 | 31 | enet | 0.4 | 3.1e-35 | 2.15 | 0.0319 | 0.61 |
| MAP1B | sCCA3 | 451 | 14 | enet | 0.41 | 1.4e-36 | 2.18 | 0.029422 | 0.61 |
| MAP1B | sCCA1 | 451 | 7 | lasso | 0.72 | 8.9e-87 | -2.12 | 0.033606 | 0.61 |
| ZNF304 | sCCA3 | 507 | 9 | lasso | 0.72 | 3.7e-86 | 2.17 | 0.0299 | 0.61 |
| RPA1 | sCCA2 | 439 | 34 | enet | 0.26 | 9.5e-22 | -2.12 | 0.034154 | 0.61 |
| PRMT7 | sCCA1 | 316 | 53 | enet | 0.7 | 4.5e-81 | -2.13 | 0.03305 | 0.61 |
| TERF2 | sCCA3 | 304 | 1 | top1 | -0.0017 | 0.48 | 2.13 | 0.03351 | 0.61 |
| NES | sCCA1 | 445 | 1 | top1 | 0.002 | 0.21 | 2.18 | 0.0292 | 0.61 |
| ARHGEF11 | sCCA3 | 503 | 10 | enet | 0.027 | 0.0022 | 2.16 | 0.0305 | 0.61 |
| LPIN3 | sCCA2 | 361 | 24 | enet | 0.22 | 4.1e-18 | 2.15 | 0.031489 | 0.61 |
| MYH10 | sCCA1 | 426 | 6 | lasso | 0.13 | 5.9e-11 | 2.16 | 0.030638 | 0.61 |
| KATNBL1 | sCCA2 | 451 | 15 | enet | 0.051 | 4.1e-05 | -2.19 | 0.02869 | 0.61 |
| NAV1 | sCCA1 | 617 | 3 | lasso | 0.26 | 7.9e-22 | 2.14 | 0.0322 | 0.61 |
| DSC2 | sCCA2 | 490 | 23 | enet | 0.022 | 0.0051 | 2.15 | 0.0312 | 0.61 |
| CCDC102A | sCCA1 | 553 | 19 | enet | 0.42 | 2.1e-37 | -2.16 | 0.03082 | 0.61 |
| RAPGEF5 | sCCA1 | 665 | 1 | top1 | 0.15 | 8.5e-13 | 2.11 | 0.03461 | 0.61 |
| MTHFS | sCCA1 | 573 | 38 | enet | 0.78 | 3.2e-102 | 2.16 | 0.03072 | 0.61 |
| FOXP4 | sCCA1 | 507 | 6 | lasso | 0.21 | 7.2e-18 | -2.11 | 0.03504 | 0.61 |
| GMPR | sCCA2 | 620 | 32 | enet | 0.14 | 2e-11 | 2.15 | 0.031389 | 0.61 |
| RIPK1 | sCCA2 | 659 | 11 | lasso | 0.35 | 2.3e-30 | 2.14 | 0.032194 | 0.61 |
| TAF8 | sCCA3 | 446 | 40 | enet | 0.094 | 2.9e-08 | -2.11 | 0.03445 | 0.61 |
| THBS1 | sCCA2 | 528 | 1 | top1 | 0.042 | 0.00019 | 2.13 | 0.03291 | 0.61 |
| TMEM62 | sCCA2 | 297 | 1 | top1 | 0.016 | 0.015 | -2.15 | 0.03152 | 0.61 |
| BCAR3 | sCCA1 | 433 | 34 | enet | 0.21 | 2.4e-17 | 2.14 | 0.0324 | 0.61 |
| KYAT3 | sCCA3 | 352 | 60 | enet | 0.49 | 1.4e-46 | -2.14 | 0.0322 | 0.61 |
| SEMA7A | sCCA2 | 374 | 2 | lasso | 0.049 | 5.8e-05 | -2.13 | 0.03338 | 0.61 |
| LARP1B | sCCA1 | 267 | 5 | lasso | 0.051 | 4e-05 | 2.11 | 0.03483 | 0.61 |
| ETNK1 | sCCA3 | 446 | 10 | enet | -0.001 | 0.41 | -2.17 | 0.0297 | 0.61 |
| GTF2A2 | sCCA1 | 521 | 16 | enet | 0.37 | 3.2e-32 | -2.13 | 0.0334 | 0.61 |
| BCL2A1 | sCCA3 | 579 | 14 | enet | 0.0075 | 0.071 | -2.14 | 0.03225 | 0.61 |
| WDR61 | sCCA3 | 395 | 1 | top1 | 0.083 | 1.7e-07 | 2.18 | 0.02939 | 0.61 |
| TOB1 | sCCA1 | 408 | 8 | lasso | 0.18 | 7e-15 | -2.12 | 0.033695 | 0.61 |
| LRRC46 | sCCA3 | 382 | 35 | enet | 0.52 | 5.2e-50 | 2.18 | 0.029463 | 0.61 |
| CELA2A | sCCA2 | 513 | 50 | enet | 0.0099 | 0.045 | 2.12 | 0.033588 | 0.61 |
| CELSR2 | sCCA2 | 421 | 1 | top1 | 0.39 | 2.5e-34 | -2.11 | 0.034675 | 0.61 |
| CRABP2 | sCCA1 | 442 | 31 | enet | 0.061 | 7.9e-06 | -2.11 | 0.0346 | 0.61 |
| RGL1 | sCCA2 | 518 | 8 | lasso | 0.045 | 0.00012 | -2.14 | 0.032085 | 0.61 |
| ZNF687 | sCCA1 | 311 | 1 | top1 | 0.12 | 3.3e-10 | -2.11 | 0.0346 | 0.61 |
| MCL1 | sCCA3 | 289 | 19 | enet | 0.018 | 0.012 | -2.1 | 0.0356 | 0.61 |
| ADAM15 | sCCA2 | 377 | 45 | enet | 0.12 | 4.5e-10 | -2.18 | 0.028986 | 0.61 |
| UBAP2L | sCCA1 | 334 | 1 | top1 | 0.071 | 1.5e-06 | -2.18 | 0.029 | 0.61 |
| HAX1 | sCCA1 | 352 | 41 | enet | 0.35 | 1.9e-30 | 2.16 | 0.0306 | 0.61 |
| VPS54 | sCCA3 | 374 | 11 | lasso | 0.053 | 2.7e-05 | 2.14 | 0.0325 | 0.61 |
| SCRN3 | sCCA3 | 377 | 9 | lasso | 0.036 | 0.00055 | 2.15 | 0.0316 | 0.61 |
| MANF | sCCA3 | 246 | 6 | lasso | 0.12 | 2.8e-10 | 2.18 | 0.029137 | 0.61 |
| SLC26A1 | sCCA3 | 399 | 7 | lasso | 0.85 | 2.5e-126 | 2.14 | 0.0325 | 0.61 |
| TBCK | sCCA3 | 312 | 3 | lasso | 0.025 | 0.0032 | -2.18 | 0.02889 | 0.61 |
| PIK3R1 | sCCA2 | 474 | 37 | enet | 0.063 | 5.5e-06 | 2.1 | 0.035605 | 0.61 |
| GIN1 | sCCA2 | 318 | 1 | top1 | 0.13 | 8.5e-11 | -2.17 | 0.030095 | 0.61 |
| PAM | sCCA3 | 305 | 7 | lasso | 0.15 | 1.7e-12 | 2.11 | 0.03466 | 0.61 |
| TMEM181 | sCCA3 | 502 | 27 | enet | 0.16 | 5.1e-13 | -2.11 | 0.03474 | 0.61 |
| SYBU | sCCA3 | 343 | 1 | top1 | 0.065 | 3.6e-06 | 2.14 | 0.03206 | 0.61 |
| AASDHPPT | sCCA2 | 368 | 1 | top1 | 0.055 | 2e-05 | 2.11 | 0.035208 | 0.61 |
| TM7SF2 | sCCA1 | 328 | 21 | enet | 0.045 | 0.00011 | 2.12 | 0.03397 | 0.61 |
| HIRIP3 | sCCA1 | 203 | 1 | top1 | 0.041 | 0.00021 | 2.14 | 0.03267 | 0.61 |
| CCDC122 | sCCA1 | 527 | 64 | enet | 0.74 | 5.2e-90 | 2.14 | 0.03243 | 0.61 |
| CCDC122 | sCCA2 | 527 | 11 | lasso | 0.63 | 7.7e-68 | -2.12 | 0.0338 | 0.61 |
| CCDC122 | sCCA3 | 527 | 13 | lasso | 0.6 | 3e-62 | 2.12 | 0.0343 | 0.61 |
| PDK1 | sCCA2 | 495 | 1 | top1 | 0.038 | 0.00035 | -2.13 | 0.0335 | 0.61 |
| ZNF256 | sCCA1 | 468 | 9 | lasso | 0.051 | 4.4e-05 | 2.18 | 0.029555 | 0.61 |
| ZFP36L2 | sCCA1 | 495 | 24 | enet | 0.071 | 1.4e-06 | -2.12 | 0.0343 | 0.61 |
| TMEM251 | sCCA3 | 383 | 5 | lasso | 0.031 | 0.0013 | -2.13 | 0.033264 | 0.61 |
| USP25 | sCCA1 | 366 | 7 | lasso | 0.13 | 5e-11 | -2.11 | 0.035075 | 0.61 |
| USP16 | sCCA3 | 425 | 3 | lasso | 0.017 | 0.014 | 2.15 | 0.031805 | 0.61 |
| BACH1 | sCCA2 | 450 | 17 | enet | 0.031 | 0.0012 | -2.18 | 0.0289 | 0.61 |
| PCGF6 | sCCA1 | 352 | 5 | lasso | 0.043 | 0.00015 | 2.14 | 0.0322 | 0.61 |
| PCGF6 | sCCA3 | 352 | 1 | top1 | 0.044 | 0.00014 | 2.12 | 0.0344 | 0.61 |
| EIF5B | sCCA2 | 320 | 1 | top1 | 0.06 | 8.9e-06 | 2.12 | 0.0344 | 0.61 |
| STARD9 | sCCA3 | 321 | 22 | enet | 0.036 | 0.00054 | 2.14 | 0.03261 | 0.61 |
| NDUFV3 | sCCA2 | 521 | 30 | lasso | 0.75 | 1e-92 | -2.14 | 0.03227 | 0.61 |
| FTCD | sCCA3 | 495 | 60 | enet | 0.41 | 4.3e-37 | -2.11 | 0.034614 | 0.61 |
| FDPS | sCCA3 | 272 | 1 | top1 | 0.035 | 0.00058 | 2.13 | 0.0331 | 0.61 |
| GBAP1 | sCCA2 | 324 | 33 | enet | 0.49 | 7.2e-46 | 2.15 | 0.031317 | 0.61 |
| GBAP1 | sCCA1 | 324 | 21 | enet | 0.65 | 6e-71 | 2.15 | 0.0317 | 0.61 |
| GBAP1 | sCCA3 | 324 | 13 | enet | 0.44 | 2e-40 | -2.13 | 0.0329 | 0.61 |
| PLXDC1 | sCCA1 | 352 | 6 | lasso | 0.36 | 2.3e-31 | 2.18 | 0.02944 | 0.61 |
| MED11 | sCCA2 | 440 | 1 | top1 | 0.076 | 6.2e-07 | -2.13 | 0.033463 | 0.61 |
| EXTL2 | sCCA1 | 438 | 4 | lasso | 0.3 | 1.1e-25 | -2.14 | 0.032 | 0.61 |
| TYW5 | sCCA3 | 364 | 1 | top1 | 0.3 | 4.1e-25 | 2.14 | 0.0323 | 0.61 |
| IGFBP7 | sCCA2 | 551 | 47 | enet | 0.073 | 1e-06 | -2.18 | 0.02962 | 0.61 |
| ADORA1 | sCCA2 | 512 | 3 | lasso | 0.12 | 1.8e-10 | -2.16 | 0.03091 | 0.61 |
| CCNL1 | sCCA2 | 419 | 5 | lasso | 0.022 | 0.0058 | -2.16 | 0.031105 | 0.61 |
| WDR43 | sCCA2 | 592 | 26 | enet | 0.1 | 9.9e-09 | -2.1 | 0.0354 | 0.61 |
| SMIM12 | sCCA2 | 348 | 53 | enet | 0.38 | 2e-33 | -2.13 | 0.033412 | 0.61 |
| MEAF6 | sCCA2 | 452 | 6 | lasso | 0.36 | 1.5e-31 | -2.17 | 0.029774 | 0.61 |
| MEAF6 | sCCA3 | 452 | 10 | lasso | 0.5 | 3.4e-48 | 2.16 | 0.0306 | 0.61 |
| MEAF6 | sCCA1 | 452 | 23 | enet | 0.58 | 6.8e-60 | 2.12 | 0.0344 | 0.61 |
| METAP1 | sCCA1 | 503 | 16 | enet | 0.22 | 1.2e-18 | 2.11 | 0.03483 | 0.61 |
| MON1A | sCCA2 | 335 | 3 | lasso | 0.024 | 0.0036 | -2.13 | 0.033325 | 0.61 |
| TEX264 | sCCA1 | 233 | 22 | enet | 0.52 | 5.4e-51 | -2.11 | 0.035 | 0.61 |
| OTUD4 | sCCA2 | 253 | 1 | top1 | 0.073 | 9.7e-07 | 2.15 | 0.03148 | 0.61 |
| MICALL2 | sCCA2 | 388 | 5 | lasso | 0.067 | 2.9e-06 | 2.1 | 0.035461 | 0.61 |
| TMEM67 | sCCA3 | 437 | 1 | top1 | 0.044 | 0.00014 | 2.14 | 0.03223 | 0.61 |
| KDM1B | sCCA3 | 495 | 1 | top1 | 0.042 | 0.00019 | -2.18 | 0.02952 | 0.61 |
| NDUFB6 | sCCA1 | 513 | 24 | enet | 0.12 | 1.8e-10 | 2.14 | 0.03265 | 0.61 |
| NSD1 | sCCA2 | 385 | 26 | enet | 0.036 | 0.00049 | 2.17 | 0.030251 | 0.61 |
| ZNF219 | sCCA3 | 529 | 25 | enet | 0.024 | 0.0038 | 2.16 | 0.030472 | 0.61 |
| TRUB1 | sCCA1 | 324 | 7 | lasso | 0.5 | 2.5e-47 | 2.12 | 0.0338 | 0.61 |
| DPCD | sCCA3 | 307 | 9 | lasso | 0.014 | 0.021 | 2.11 | 0.0352 | 0.61 |
| FRS2 | sCCA2 | 561 | 36 | enet | 0.11 | 1.8e-09 | -2.11 | 0.034613 | 0.61 |
| ATF7IP2 | sCCA2 | 611 | 8 | lasso | 0.21 | 1.4e-17 | -2.12 | 0.03361 | 0.61 |
| NAV2 | sCCA1 | 573 | 22 | enet | 0.00024 | 0.3 | -2.19 | 0.028655 | 0.61 |
| ZNF91 | sCCA3 | 237 | 1 | top1 | 0.023 | 0.005 | -2.1 | 0.0356 | 0.61 |
| POP5 | sCCA1 | 410 | 5 | lasso | 0.32 | 5.2e-27 | -2.1 | 0.035427 | 0.61 |
| IRGQ | sCCA3 | 388 | 14 | enet | 0.052 | 3.4e-05 | 2.19 | 0.0287 | 0.61 |
| GATAD2A | sCCA2 | 342 | 24 | enet | 0.071 | 1.5e-06 | -2.1 | 0.035424 | 0.61 |
| SERTAD3 | sCCA3 | 372 | 12 | lasso | 0.23 | 1.6e-19 | 2.14 | 0.0323 | 0.61 |
| EVPL | sCCA3 | 395 | 35 | enet | 0.017 | 0.013 | -2.14 | 0.032409 | 0.61 |
| ECI1 | sCCA2 | 342 | 37 | enet | 0.44 | 9.8e-40 | -2.14 | 0.03231 | 0.61 |
| PCMTD1 | sCCA1 | 426 | 8 | lasso | 0.32 | 6.9e-28 | 2.14 | 0.0322 | 0.61 |
| CNNM3 | sCCA3 | 173 | 1 | top1 | 0.069 | 1.8e-06 | -2.13 | 0.0328 | 0.61 |
| ADAL | sCCA3 | 265 | 8 | lasso | 0.61 | 2.9e-64 | -2.13 | 0.03326 | 0.61 |
| LCMT2 | sCCA2 | 265 | 30 | enet | 0.23 | 6.2e-19 | 2.14 | 0.03236 | 0.61 |
| ZBTB49 | sCCA2 | 380 | 27 | enet | 0.31 | 1.7e-26 | 2.14 | 0.03268 | 0.61 |
| FAM110B | sCCA3 | 479 | 8 | lasso | 0.051 | 4.4e-05 | -2.14 | 0.03232 | 0.61 |
| THBS3 | sCCA2 | 333 | 29 | enet | 0.21 | 7e-18 | 2.16 | 0.030623 | 0.61 |
| BOLA2B | sCCA1 | 211 | 1 | top1 | 0.029 | 0.0017 | 2.14 | 0.03267 | 0.61 |
| UBB | sCCA1 | 291 | 19 | enet | 0.062 | 6.3e-06 | -2.11 | 0.034934 | 0.61 |
| METTL7B | sCCA1 | 310 | 45 | enet | 0.28 | 3.2e-23 | 2.18 | 0.029007 | 0.61 |
| ATPAF2 | sCCA1 | 349 | 10 | lasso | 0.47 | 2e-44 | 2.14 | 0.032608 | 0.61 |
| QARS | sCCA1 | 254 | 7 | lasso | 0.05 | 4.8e-05 | 2.14 | 0.0325 | 0.61 |
| SLFN12 | sCCA1 | 378 | 32 | enet | 0.35 | 6.6e-30 | 2.11 | 0.034542 | 0.61 |
| MBOAT1 | sCCA2 | 529 | 7 | lasso | 0.31 | 1.5e-26 | 2.11 | 0.034824 | 0.61 |
| COPRS | sCCA2 | 321 | 3 | lasso | 0.22 | 3.3e-18 | -2.12 | 0.034242 | 0.61 |
| AGAP5 | sCCA1 | 252 | 15 | enet | 0.21 | 8.8e-18 | 2.18 | 0.0293 | 0.61 |
| DAG1 | sCCA3 | 288 | 20 | enet | 0.14 | 1.4e-11 | 2.14 | 0.032543 | 0.61 |
| ATP2A2 | sCCA1 | 235 | 7 | lasso | 0.17 | 2.6e-14 | -2.16 | 0.030985 | 0.61 |
| METTL15P1 | sCCA2 | 427 | 38 | enet | 0.23 | 9.6e-19 | -2.19 | 0.028797 | 0.61 |
| METTL15P1 | sCCA3 | 427 | 2 | lasso | 0.14 | 1.1e-11 | 2.11 | 0.035116 | 0.61 |
| PCCA | sCCA1 | 406 | 10 | enet | 0.078 | 4.2e-07 | 2.1 | 0.03554 | 0.61 |
| ARL10 | sCCA3 | 311 | 19 | enet | 0.1 | 6.9e-09 | 2.11 | 0.03506 | 0.61 |
| SHMT1 | sCCA2 | 262 | 33 | enet | 0.56 | 1.4e-56 | 2.12 | 0.034191 | 0.61 |
| C19orf18 | sCCA1 | 454 | 58 | enet | 0.28 | 1.8e-23 | -2.18 | 0.029428 | 0.61 |
| C8orf31 | sCCA3 | 408 | 7 | lasso | 0.085 | 1.3e-07 | -2.18 | 0.02893 | 0.61 |
| CCDC71 | sCCA2 | 258 | 19 | enet | 0.16 | 3.3e-13 | 2.13 | 0.033158 | 0.61 |
| PNPLA2 | sCCA3 | 425 | 1 | top1 | 0.061 | 7.3e-06 | 2.15 | 0.0316 | 0.61 |
| ZNF518A | sCCA3 | 463 | 1 | top1 | 0.22 | 2.1e-18 | -2.14 | 0.0324 | 0.61 |
| ODF3B | sCCA1 | 316 | 2 | lasso | 0.23 | 2.6e-19 | -2.14 | 0.0322 | 0.61 |
| ZNF354B | sCCA3 | 580 | 8 | lasso | 0.073 | 9.6e-07 | -2.12 | 0.034168 | 0.61 |
| SUZ12 | sCCA2 | 307 | 1 | top1 | 0.01 | 0.044 | 2.11 | 0.035117 | 0.61 |
| LGALS7B | sCCA1 | 399 | 8 | lasso | 0.75 | 2.8e-92 | -2.11 | 0.034592 | 0.61 |
| LGALS7B | sCCA3 | 399 | 10 | lasso | 0.55 | 1.7e-54 | -2.11 | 0.0351 | 0.61 |
| CTC1 | sCCA1 | 451 | 8 | lasso | 0.34 | 5e-29 | 2.1 | 0.03548 | 0.61 |
| DCTPP1 | sCCA1 | 191 | 1 | top1 | 0.087 | 9.5e-08 | 2.14 | 0.03267 | 0.61 |
| PAK2 | sCCA1 | 406 | 6 | lasso | 0.35 | 1.6e-30 | 2.1 | 0.0353 | 0.61 |
| LRRC57 | sCCA2 | 332 | 3 | lasso | 0.42 | 1.8e-37 | 2.18 | 0.02923 | 0.61 |
| BBS12 | sCCA2 | 293 | 9 | enet | 0.078 | 4.3e-07 | -2.11 | 0.03475 | 0.61 |
| AEN | sCCA2 | 473 | 24 | enet | 0.11 | 3.8e-09 | -2.12 | 0.03402 | 0.61 |
| NOP10 | sCCA2 | 445 | 10 | lasso | 0.34 | 1.2e-29 | 2.1 | 0.03572 | 0.61 |
| SHMT2 | sCCA1 | 339 | 10 | lasso | 0.053 | 3.1e-05 | -2.13 | 0.03295 | 0.61 |
| PRR14L | sCCA1 | 372 | 12 | lasso | 0.13 | 3.5e-11 | 2.18 | 0.02947 | 0.61 |
| LIN9 | sCCA3 | 396 | 2 | lasso | 0.081 | 2.6e-07 | -2.18 | 0.029 | 0.61 |
| DIABLO | sCCA3 | 332 | 1 | top1 | 0.043 | 0.00016 | -2.11 | 0.0344 | 0.61 |
| VPS33B | sCCA1 | 496 | 22 | enet | 0.36 | 1.6e-31 | -2.17 | 0.03036 | 0.61 |
| SPDYE12P | sCCA3 | 87 | 35 | enet | 0.38 | 1.5e-33 | 2.18 | 0.028903 | 0.61 |
| CA13 | sCCA3 | 243 | 33 | enet | 0.082 | 2.1e-07 | 2.11 | 0.03525 | 0.61 |
| IFITM2 | sCCA3 | 322 | 13 | lasso | 0.16 | 2.2e-13 | 2.11 | 0.03524 | 0.61 |
| IFITM1 | sCCA3 | 325 | 7 | lasso | 0.26 | 5.4e-22 | -2.18 | 0.02898 | 0.61 |
| ZNF267 | sCCA1 | 157 | 2 | lasso | 0.069 | 2.1e-06 | -2.14 | 0.03224 | 0.61 |
| CCDC84 | sCCA1 | 378 | 1 | top1 | 0.21 | 1.4e-17 | 2.13 | 0.033165 | 0.61 |
| HEXIM1 | sCCA2 | 280 | 1 | top1 | 0.1 | 6.7e-09 | -2.15 | 0.031621 | 0.61 |
| ZNF286A | sCCA2 | 353 | 30 | enet | 0.06 | 9.1e-06 | 2.16 | 0.03103 | 0.61 |
| SEMA4D | sCCA2 | 471 | 28 | enet | 0.3 | 5.9e-26 | -2.12 | 0.034336 | 0.61 |
| RILPL1 | sCCA1 | 369 | 28 | enet | 0.062 | 6.7e-06 | -2.16 | 0.030396 | 0.61 |
| LAMTOR4 | sCCA2 | 274 | 17 | lasso | 0.35 | 5.7e-30 | 2.14 | 0.032247 | 0.61 |
| HES4 | sCCA2 | 186 | 23 | enet | 0.14 | 6e-12 | -2.16 | 0.030914 | 0.61 |
| ADAT2 | sCCA1 | 513 | 9 | lasso | 0.64 | 4e-69 | -2.13 | 0.03331 | 0.61 |
| TDRD7 | sCCA3 | 345 | 1 | top1 | 0.063 | 5.5e-06 | -2.17 | 0.02999 | 0.61 |
| XPNPEP3 | sCCA2 | 275 | 1 | top1 | 0.4 | 3.1e-35 | 2.15 | 0.031538 | 0.61 |
| XPNPEP3 | sCCA1 | 275 | 1 | top1 | 0.46 | 5.7e-43 | 2.15 | 0.03154 | 0.61 |
| XPNPEP3 | sCCA3 | 275 | 1 | top1 | 0.31 | 1.1e-26 | 2.15 | 0.03154 | 0.61 |
| TRAPPC4 | sCCA1 | 382 | 1 | top1 | 0.81 | 1.1e-112 | 2.11 | 0.035237 | 0.61 |
| TRAPPC4 | sCCA2 | 382 | 1 | top1 | 0.73 | 3.5e-87 | 2.11 | 0.035237 | 0.61 |
| TRAPPC4 | sCCA3 | 382 | 1 | top1 | 0.81 | 2.8e-110 | 2.11 | 0.03524 | 0.61 |
| ZNF431 | sCCA1 | 283 | 14 | lasso | 0.12 | 2e-10 | -2.13 | 0.033585 | 0.61 |
| LAMB3 | sCCA3 | 497 | 4 | lasso | 0.028 | 0.0021 | -2.18 | 0.0294 | 0.61 |
| PIGN | sCCA1 | 508 | 16 | lasso | 0.65 | 7.5e-71 | -2.14 | 0.03228 | 0.61 |
| FAR1 | sCCA2 | 496 | 10 | lasso | 0.0095 | 0.049 | 2.12 | 0.033954 | 0.61 |
| 5-Mar | sCCA1 | 305 | 1 | top1 | 0.21 | 1.3e-17 | 2.17 | 0.0297 | 0.61 |
| ZNF770 | sCCA2 | 405 | 8 | lasso | 0.021 | 0.0067 | 2.13 | 0.03357 | 0.61 |
| RPS6KL1 | sCCA2 | 379 | 1 | top1 | 0.32 | 2.6e-27 | -2.17 | 0.0303 | 0.61 |
| RPS6KL1 | sCCA1 | 379 | 1 | top1 | 0.48 | 4e-45 | 2.17 | 0.03033 | 0.61 |
| ZKSCAN8 | sCCA3 | 465 | 32 | enet | 0.095 | 2.5e-08 | -2.15 | 0.03194 | 0.61 |
| PIM3 | sCCA2 | 440 | 30 | enet | 0.15 | 6.7e-13 | 2.19 | 0.02874 | 0.61 |
| CTNND1 | sCCA1 | 392 | 4 | lasso | 0.065 | 3.6e-06 | -2.12 | 0.033625 | 0.61 |
| PAPSS2 | sCCA2 | 323 | 34 | enet | 0.32 | 5.5e-27 | -2.14 | 0.0324 | 0.61 |
| OSTC | sCCA1 | 431 | 1 | top1 | 0.064 | 4.3e-06 | -2.13 | 0.03356 | 0.61 |
| GRK5 | sCCA3 | 496 | 5 | lasso | 0.014 | 0.022 | 2.17 | 0.03 | 0.61 |
| CIPC | sCCA1 | 539 | 1 | top1 | 0.14 | 4.5e-12 | 2.13 | 0.03289 | 0.61 |
| SGMS1 | sCCA1 | 427 | 14 | lasso | 0.096 | 2e-08 | -2.14 | 0.032 | 0.61 |
| C1orf53 | sCCA1 | 358 | 32 | enet | 0.17 | 2.7e-14 | -2.16 | 0.031 | 0.61 |
| MAFB | sCCA2 | 430 | 25 | enet | 0.025 | 0.0034 | -2.13 | 0.033045 | 0.61 |
| ZNRD1ASP | sCCA3 | 39 | 29 | enet | 0.31 | 5e-26 | -2.13 | 0.033398 | 0.61 |
| HACD2 | sCCA2 | 367 | 47 | enet | 0.19 | 6.7e-16 | 2.11 | 0.034587 | 0.61 |
| IGHG3 | sCCA3 | 43 | 5 | enet | 0.032 | 0.00098 | -2.13 | 0.032762 | 0.61 |
| FAM209B | sCCA1 | 603 | 1 | top1 | 0.12 | 4.6e-10 | -2.11 | 0.03505 | 0.61 |
| PPIAP29 | sCCA3 | 640 | 1 | top1 | 0.029 | 0.0017 | -2.14 | 0.03208 | 0.61 |
| PPP3CB-AS1 | sCCA2 | 246 | 5 | lasso | 0.049 | 6e-05 | -2.13 | 0.033 | 0.61 |
| RP11-134G8.5 | sCCA3 | 671 | 6 | lasso | 0.16 | 2.3e-13 | -2.11 | 0.0347 | 0.61 |
| AC005076.5 | sCCA3 | 462 | 34 | enet | 0.03 | 0.0014 | -2.16 | 0.030876 | 0.61 |
| RPS4XP16 | sCCA2 | 491 | 30 | enet | 0.074 | 9.1e-07 | 2.11 | 0.03444 | 0.61 |
| ENTPD1-AS1 | sCCA2 | 466 | 14 | lasso | 0.14 | 8.5e-12 | 2.11 | 0.0349 | 0.61 |
| BACH1-IT2 | sCCA1 | 429 | 1 | top1 | 0.084 | 1.5e-07 | 2.13 | 0.033347 | 0.61 |
| PA2G4P4 | sCCA3 | 450 | 23 | enet | 0.064 | 4.3e-06 | -2.14 | 0.03201 | 0.61 |
| HCG4P7 | sCCA3 | 40 | 32 | enet | 0.27 | 6.6e-23 | -2.11 | 0.034716 | 0.61 |
| AF129075.5 | sCCA3 | 427 | 21 | enet | 0.02 | 0.0077 | -2.15 | 0.03127 | 0.61 |
| FLJ27354 | sCCA3 | 363 | 23 | enet | 0.031 | 0.0012 | -2.14 | 0.032 | 0.61 |
| AC078883.3 | sCCA2 | 467 | 1 | top1 | 0.047 | 8.4e-05 | -2.13 | 0.0335 | 0.61 |
| RPS2P32 | sCCA3 | 409 | 40 | enet | 0.067 | 3e-06 | -2.16 | 0.030461 | 0.61 |
| SMG7-AS1 | sCCA2 | 511 | 66 | enet | 0.051 | 4.3e-05 | 2.1 | 0.035373 | 0.61 |
| FAM133B | sCCA2 | 295 | 1 | top1 | 0.055 | 1.9e-05 | 2.15 | 0.031911 | 0.61 |
| WASH4P | sCCA2 | 219 | 21 | enet | 0.036 | 0.00055 | -2.14 | 0.03199 | 0.61 |
| SNHG20 | sCCA2 | 575 | 1 | top1 | 0.2 | 9.5e-17 | -2.12 | 0.033984 | 0.61 |
| SNRPGP10 | sCCA1 | 585 | 35 | enet | 0.061 | 7.2e-06 | 2.12 | 0.0342 | 0.61 |
| KRR1P1 | sCCA1 | 437 | 20 | enet | 0.022 | 0.0056 | -2.16 | 0.03103 | 0.61 |
| FAM21FP | sCCA2 | 222 | 6 | lasso | 0.32 | 1.3e-27 | 2.12 | 0.0336 | 0.61 |
| RPL23AP64 | sCCA3 | 378 | 52 | enet | 0.2 | 5.2e-17 | 2.13 | 0.03329 | 0.61 |
| PISD | sCCA1 | 333 | 9 | lasso | 0.22 | 1.6e-18 | -2.15 | 0.03175 | 0.61 |
| RP11-466H18.1 | sCCA2 | 402 | 3 | lasso | 0.036 | 0.00049 | -2.11 | 0.034709 | 0.61 |
| RP11-7F17.5 | sCCA1 | 545 | 14 | enet | 0.33 | 1.6e-28 | 2.18 | 0.02912 | 0.61 |
| PGAM5 | sCCA3 | 256 | 20 | enet | 0.061 | 7.5e-06 | 2.17 | 0.0303 | 0.61 |
| RP11-932O9.7 | sCCA3 | 192 | 1 | top1 | 0.069 | 2e-06 | -2.1 | 0.03558 | 0.61 |
| NRAV | sCCA2 | 397 | 7 | lasso | 0.1 | 1e-08 | 2.1 | 0.035626 | 0.61 |
| CCDC153 | sCCA2 | 376 | 6 | lasso | 0.42 | 1.3e-37 | 2.14 | 0.032171 | 0.61 |
| UGDH-AS1 | sCCA1 | 353 | 16 | enet | 0.2 | 1.2e-16 | 2.15 | 0.03184 | 0.61 |
| LRRC37BP1 | sCCA1 | 292 | 13 | lasso | 0.24 | 8.2e-20 | 2.11 | 0.034738 | 0.61 |
| CTD-2152M20.2 | sCCA1 | 558 | 1 | top1 | 0.018 | 0.011 | 2.15 | 0.03135 | 0.61 |
| METTL21EP | sCCA1 | 710 | 37 | enet | 0.47 | 1.3e-43 | -2.14 | 0.03256 | 0.61 |
| SHANK3 | sCCA1 | 235 | 1 | top1 | 0.26 | 3.8e-22 | 2.12 | 0.0337 | 0.61 |
| SHANK3 | sCCA3 | 235 | 1 | top1 | 0.089 | 6.9e-08 | 2.12 | 0.0337 | 0.61 |
| RP11-521B24.3 | sCCA3 | 167 | 14 | enet | 0.085 | 1.4e-07 | 2.18 | 0.029232 | 0.61 |
| RP11-326C3.11 | sCCA3 | 328 | 26 | enet | 0.31 | 1.7e-26 | -2.17 | 0.03021 | 0.61 |
| RP11-110I1.5 | sCCA1 | 378 | 1 | top1 | 0.15 | 5.9e-13 | 2.11 | 0.035237 | 0.61 |
| CTSO | sCCA3 | 470 | 23 | enet | 0.36 | 2e-31 | -2.11 | 0.03454 | 0.61 |
| HP | sCCA2 | 358 | 33 | enet | 0.23 | 1.9e-19 | -2.12 | 0.03367 | 0.61 |
| CHURC1 | sCCA2 | 492 | 1 | top1 | 0.87 | 3.6e-136 | 2.13 | 0.0331 | 0.61 |
| CHURC1 | sCCA1 | 492 | 1 | top1 | 0.92 | 3.5e-171 | 2.13 | 0.03314 | 0.61 |
| CHURC1 | sCCA3 | 492 | 1 | top1 | 0.92 | 1.2e-169 | 2.13 | 0.033143 | 0.61 |
| ARHGAP5-AS1 | sCCA3 | 485 | 1 | top1 | 0.056 | 1.7e-05 | -2.11 | 0.034467 | 0.61 |
| RP11-395I6.3 | sCCA2 | 432 | 1 | top1 | 0.066 | 3.2e-06 | -2.18 | 0.02924 | 0.61 |
| RP11-352G18.2 | sCCA2 | 507 | 10 | lasso | 0.087 | 8.8e-08 | -2.11 | 0.0352 | 0.61 |
| LINC00622 | sCCA1 | 392 | 30 | enet | 0.046 | 9.2e-05 | -2.13 | 0.0328 | 0.61 |
| RP11-16E18.3 | sCCA3 | 389 | 1 | top1 | 0.069 | 2.1e-06 | -2.13 | 0.03333 | 0.61 |
| CTD-2349P21.9 | sCCA2 | 268 | 47 | enet | 0.24 | 2.1e-20 | -2.15 | 0.031774 | 0.61 |
| RP11-258F1.1 | sCCA2 | 304 | 9 | lasso | 0.068 | 2.2e-06 | 2.17 | 0.030137 | 0.61 |
| SH3GL1P1 | sCCA1 | 288 | 1 | top1 | 0.081 | 2.5e-07 | 2.11 | 0.035117 | 0.61 |
| ASB16-AS1 | sCCA3 | 351 | 30 | enet | 0.36 | 3.1e-31 | 2.15 | 0.031673 | 0.61 |
| ASB16-AS1 | sCCA1 | 351 | 54 | enet | 0.43 | 2.7e-39 | 2.12 | 0.03442 | 0.61 |
| CTD-2020K17.1 | sCCA2 | 265 | 42 | enet | 0.019 | 0.0085 | -2.11 | 0.034769 | 0.61 |
| SNHG22 | sCCA2 | 566 | 39 | enet | 0.13 | 6.1e-11 | -2.11 | 0.0346 | 0.61 |
| RP11-635N19.1 | sCCA2 | 506 | 11 | lasso | 0.21 | 2.3e-17 | -2.16 | 0.0311 | 0.61 |
| CTC-429P9.2 | sCCA1 | 383 | 9 | lasso | 0.24 | 7.4e-20 | -2.12 | 0.034418 | 0.61 |
| RP11-158H5.7 | sCCA1 | 405 | 17 | enet | 0.082 | 2.3e-07 | 2.14 | 0.03252 | 0.61 |
| CTD-3131K8.2 | sCCA3 | 422 | 1 | top1 | 0.066 | 3.1e-06 | 2.18 | 0.0289 | 0.61 |
| RP11-248J18.2 | sCCA1 | 466 | 4 | lasso | 0.12 | 3.9e-10 | 2.15 | 0.03193 | 0.61 |
| RP11-324I22.3 | sCCA2 | 313 | 1 | top1 | 0.049 | 5.4e-05 | 2.14 | 0.032 | 0.61 |
| HMGN3-AS1 | sCCA3 | 430 | 7 | lasso | 0.23 | 2.9e-19 | -2.15 | 0.03167 | 0.61 |
| RP5-1042K10.13 | sCCA2 | 292 | 4 | lasso | 0.043 | 0.00016 | 2.15 | 0.031432 | 0.61 |
| AP000240.9 | sCCA2 | 431 | 1 | top1 | 0.069 | 2e-06 | 2.1 | 0.03564 | 0.61 |
| RP11-416N2.4 | sCCA1 | 371 | 6 | lasso | 0.074 | 7.8e-07 | 2.11 | 0.0352 | 0.61 |
| RP11-731C17.2 | sCCA3 | 336 | 9 | lasso | 0.085 | 1.2e-07 | -2.16 | 0.03077 | 0.61 |
| RP11-45A17.4 | sCCA1 | 516 | 23 | enet | 0.053 | 3.2e-05 | -2.12 | 0.0341 | 0.61 |
| RP11-7F17.8 | sCCA2 | 548 | 7 | lasso | 0.034 | 0.00077 | 2.15 | 0.0314 | 0.61 |
| RP11-227G15.10 | sCCA3 | 248 | 17 | enet | -0.0031 | 0.79 | 2.15 | 0.031284 | 0.61 |
| HIST1H3E | sCCA2 | 645 | 26 | enet | 0.54 | 7.3e-53 | -2.15 | 0.03142 | 0.61 |
| FCGBP | sCCA3 | 400 | 1 | top1 | 0.082 | 2.1e-07 | -2.16 | 0.0305 | 0.61 |
| RP11-872J21.5 | sCCA2 | 572 | 24 | enet | 0.0083 | 0.061 | 2.18 | 0.0291 | 0.61 |
| RP11-481J2.4 | sCCA2 | 472 | 35 | enet | 0.011 | 0.038 | -2.13 | 0.03354 | 0.61 |
| AL133243.1 | sCCA2 | 376 | 1 | top1 | 0.26 | 1.9e-21 | 2.15 | 0.0313 | 0.61 |
| AL133243.1 | sCCA3 | 376 | 28 | enet | 0.48 | 7.9e-45 | 2.11 | 0.0352 | 0.61 |
| CTD-2537I9.18 | sCCA2 | 572 | 8 | lasso | 0.038 | 0.00035 | -2.1 | 0.035513 | 0.61 |
| GTF2IP1 | sCCA3 | 115 | 13 | enet | 0.015 | 0.019 | 2.14 | 0.032658 | 0.61 |
| RP11-81A1.6 | sCCA3 | 574 | 6 | lasso | 0.5 | 4e-48 | -2.1 | 0.0355 | 0.61 |
| AC007191.4 | sCCA2 | 369 | 27 | enet | 0.021 | 0.007 | -2.11 | 0.034652 | 0.61 |
| RP11-44F14.6 | sCCA2 | 387 | 7 | lasso | 0.48 | 6.5e-45 | -2.14 | 0.03243 | 0.61 |
| RP11-1099M24.6 | sCCA3 | 507 | 22 | enet | 0.22 | 2.6e-18 | 2.11 | 0.03461 | 0.61 |
| OSBPL7 | sCCA1 | 385 | 5 | lasso | 0.44 | 1e-40 | 2.07 | 0.038576 | 0.62 |
| TTC19 | sCCA2 | 277 | 7 | lasso | 0.42 | 1.8e-38 | 2.06 | 0.039322 | 0.62 |
| UFL1 | sCCA1 | 422 | 1 | top1 | 0.12 | 3.9e-10 | 2.06 | 0.03954 | 0.62 |
| PIAS1 | sCCA2 | 449 | 9 | lasso | 0.28 | 6.4e-24 | 2.07 | 0.0389 | 0.62 |
| CYFIP2 | sCCA1 | 473 | 1 | top1 | 0.32 | 1.1e-27 | 2.07 | 0.038908 | 0.62 |
| TAB2 | sCCA2 | 480 | 11 | lasso | 0.037 | 0.00042 | 2.07 | 0.0387 | 0.62 |
| TM7SF3 | sCCA2 | 525 | 16 | lasso | 0.54 | 4.4e-53 | 2.07 | 0.038288 | 0.62 |
| TM7SF3 | sCCA1 | 525 | 16 | lasso | 0.67 | 6.4e-76 | 2.06 | 0.039401 | 0.62 |
| PRKCQ | sCCA3 | 783 | 37 | enet | 0.067 | 2.9e-06 | -2.1 | 0.0359 | 0.62 |
| YBX1 | sCCA1 | 375 | 15 | enet | 0.073 | 1e-06 | -2.08 | 0.0378 | 0.62 |
| PRKCZ | sCCA3 | 366 | 1 | top1 | 0.032 | 0.00095 | 2.09 | 0.0366 | 0.62 |
| PRR11 | sCCA2 | 291 | 1 | top1 | 0.14 | 4.4e-12 | -2.06 | 0.039372 | 0.62 |
| TGFBR3 | sCCA3 | 499 | 19 | enet | 0.01 | 0.043 | -2.09 | 0.0369 | 0.62 |
| MAPK6 | sCCA2 | 372 | 1 | top1 | 0.074 | 7.8e-07 | 2.08 | 0.03716 | 0.62 |
| VASH1 | sCCA2 | 564 | 19 | enet | 0.017 | 0.013 | 2.07 | 0.0384 | 0.62 |
| NFATC3 | sCCA2 | 276 | 30 | enet | 0.34 | 2e-29 | 2.09 | 0.03685 | 0.62 |
| SNRPA | sCCA1 | 431 | 1 | top1 | 0.055 | 2.2e-05 | 2.07 | 0.038604 | 0.62 |
| SLC1A3 | sCCA2 | 420 | 36 | enet | 0.12 | 1.9e-10 | -2.06 | 0.039087 | 0.62 |
| EPB41L2 | sCCA3 | 439 | 27 | enet | 0.023 | 0.0048 | 2.08 | 0.03774 | 0.62 |
| MRPL22 | sCCA1 | 422 | 18 | enet | 0.12 | 1.3e-10 | -2.08 | 0.037641 | 0.62 |
| GSK3B | sCCA1 | 453 | 10 | lasso | 0.34 | 3.2e-29 | 2.06 | 0.0397 | 0.62 |
| KAT6A | sCCA3 | 370 | 18 | enet | 0.055 | 2.1e-05 | -2.07 | 0.03878 | 0.62 |
| REST | sCCA1 | 529 | 11 | lasso | 0.14 | 1.8e-11 | 2.09 | 0.03675 | 0.62 |
| ASAP3 | sCCA2 | 299 | 27 | enet | 0.12 | 1.8e-10 | -2.07 | 0.038544 | 0.62 |
| ASAP3 | sCCA1 | 299 | 30 | enet | 0.48 | 7e-45 | 2.06 | 0.0393 | 0.62 |
| RGS1 | sCCA2 | 363 | 13 | lasso | 0.051 | 3.9e-05 | 2.06 | 0.039218 | 0.62 |
| ALKBH5 | sCCA1 | 304 | 28 | enet | 0.59 | 1.4e-61 | 2.06 | 0.039337 | 0.62 |
| ALKBH5 | sCCA2 | 304 | 35 | enet | 0.6 | 8.3e-62 | -2.06 | 0.039538 | 0.62 |
| HNRNPC | sCCA3 | 491 | 43 | enet | 0.034 | 0.00068 | 2.08 | 0.037297 | 0.62 |
| GRK3 | sCCA3 | 572 | 33 | enet | 0.39 | 2.6e-34 | -2.08 | 0.03786 | 0.62 |
| CYP2D6 | sCCA2 | 348 | 8 | lasso | 0.5 | 4.1e-47 | 2.07 | 0.038818 | 0.62 |
| CBX7 | sCCA3 | 409 | 5 | lasso | 0.026 | 0.0027 | 2.06 | 0.03987 | 0.62 |
| SLC25A17 | sCCA2 | 277 | 7 | lasso | 0.12 | 6.5e-10 | -2.06 | 0.039573 | 0.62 |
| TRIB3 | sCCA3 | 468 | 5 | lasso | 0.37 | 4.8e-32 | 2.07 | 0.03807 | 0.62 |
| LMF1 | sCCA1 | 441 | 8 | lasso | 0.44 | 6.4e-40 | -2.08 | 0.03725 | 0.62 |
| MAZ | sCCA1 | 157 | 1 | top1 | 0.026 | 0.0026 | -2.1 | 0.03616 | 0.62 |
| LEPROTL1 | sCCA3 | 377 | 15 | enet | 0.23 | 2e-19 | 2.1 | 0.03605 | 0.62 |
| RASAL3 | sCCA3 | 549 | 1 | top1 | 0.038 | 0.00036 | 2.07 | 0.0387 | 0.62 |
| RAB3A | sCCA1 | 420 | 1 | top1 | 0.15 | 2.2e-12 | 2.07 | 0.038778 | 0.62 |
| ATP13A1 | sCCA2 | 330 | 6 | lasso | 0.085 | 1.3e-07 | 2.09 | 0.036794 | 0.62 |
| CDK6 | sCCA2 | 319 | 6 | lasso | 0.049 | 6.1e-05 | -2.07 | 0.038876 | 0.62 |
| CCNJ | sCCA1 | 489 | 53 | enet | 0.11 | 1.6e-09 | -2.08 | 0.0372 | 0.62 |
| RAPGEFL1 | sCCA2 | 294 | 5 | lasso | 0.53 | 5.6e-52 | -2.06 | 0.039369 | 0.62 |
| PIGL | sCCA2 | 299 | 8 | lasso | 0.15 | 1.3e-12 | -2.05 | 0.039902 | 0.62 |
| MED31 | sCCA3 | 566 | 7 | lasso | 0.17 | 3.7e-14 | -2.08 | 0.037853 | 0.62 |
| WFS1 | sCCA3 | 673 | 4 | lasso | 0.24 | 2.1e-20 | 2.08 | 0.03753 | 0.62 |
| CCDC34 | sCCA2 | 391 | 7 | lasso | 0.046 | 8.9e-05 | 2.08 | 0.037303 | 0.62 |
| PTPMT1 | sCCA3 | 276 | 11 | enet | 0.08 | 3e-07 | -2.08 | 0.03773 | 0.62 |
| CYP27B1 | sCCA3 | 346 | 1 | top1 | 0.064 | 4.9e-06 | 2.07 | 0.038 | 0.62 |
| CDK2AP1 | sCCA1 | 340 | 30 | enet | 0.72 | 1.1e-85 | -2.09 | 0.036927 | 0.62 |
| HCFC2 | sCCA3 | 704 | 12 | lasso | 0.0039 | 0.14 | -2.06 | 0.0393 | 0.62 |
| FAM162A | sCCA1 | 472 | 16 | lasso | 0.31 | 1.6e-26 | -2.1 | 0.036 | 0.62 |
| USP4 | sCCA3 | 277 | 1 | top1 | 0.031 | 0.0012 | 2.07 | 0.038587 | 0.62 |
| KANSL3 | sCCA1 | 203 | 1 | top1 | 0.086 | 1e-07 | -2.09 | 0.037 | 0.62 |
| TANC1 | sCCA2 | 522 | 39 | enet | 0.12 | 5.3e-10 | 2.06 | 0.0395 | 0.62 |
| LRRC42 | sCCA3 | 493 | 1 | top1 | 0.078 | 4.5e-07 | -2.07 | 0.0381 | 0.62 |
| TRIM62 | sCCA2 | 375 | 3 | lasso | 0.016 | 0.016 | -2.07 | 0.038701 | 0.62 |
| MAP7D1 | sCCA3 | 322 | 1 | top1 | 0.017 | 0.013 | -2.06 | 0.0397 | 0.62 |
| PTCH2 | sCCA2 | 322 | 1 | top1 | 0.064 | 4.8e-06 | 2.1 | 0.035815 | 0.62 |
| MARC2 | sCCA3 | 404 | 3 | lasso | 0.33 | 8.1e-29 | 2.1 | 0.0361 | 0.62 |
| RARRES1 | sCCA1 | 400 | 1 | top1 | 0.12 | 4.1e-10 | 2.08 | 0.0378 | 0.62 |
| RBM18 | sCCA3 | 541 | 2 | lasso | 0.16 | 1.8e-13 | 2.05 | 0.03989 | 0.62 |
| PLXDC2 | sCCA3 | 590 | 1 | top1 | 0.019 | 0.0095 | -2.07 | 0.0385 | 0.62 |
| FAM117A | sCCA3 | 403 | 1 | top1 | 0.055 | 2.1e-05 | -2.07 | 0.038289 | 0.62 |
| POPDC2 | sCCA1 | 516 | 16 | enet | 0.4 | 3.5e-36 | -2.09 | 0.0364 | 0.62 |
| PKN1 | sCCA2 | 371 | 12 | enet | 0.027 | 0.0024 | 2.09 | 0.036174 | 0.62 |
| OPRL1 | sCCA1 | 312 | 27 | enet | 0.61 | 1e-64 | 2.09 | 0.03625 | 0.62 |
| ZNF436 | sCCA1 | 310 | 1 | top1 | 0.73 | 3.1e-89 | -2.07 | 0.0383 | 0.62 |
| ZNF436 | sCCA3 | 310 | 1 | top1 | 0.72 | 1.4e-86 | 2.07 | 0.0383 | 0.62 |
| ZNF436 | sCCA2 | 310 | 1 | top1 | 0.52 | 6.3e-50 | -2.07 | 0.038339 | 0.62 |
| PSMB2 | sCCA1 | 237 | 1 | top1 | 0.037 | 0.00041 | 2.06 | 0.0391 | 0.62 |
| HSPA2 | sCCA1 | 497 | 17 | enet | 0.26 | 9.2e-22 | 2.08 | 0.03788 | 0.62 |
| RHOT1 | sCCA2 | 307 | 27 | enet | 0.17 | 5.3e-14 | -2.1 | 0.036123 | 0.62 |
| CCDC136 | sCCA1 | 340 | 7 | lasso | 0.15 | 1.1e-12 | -2.07 | 0.03819 | 0.62 |
| TTBK2 | sCCA2 | 282 | 11 | lasso | 0.08 | 3.1e-07 | -2.08 | 0.03799 | 0.62 |
| CAMSAP1 | sCCA3 | 430 | 1 | top1 | 0.047 | 7.8e-05 | 2.06 | 0.03924 | 0.62 |
| MED18 | sCCA3 | 264 | 26 | enet | 0.057 | 1.6e-05 | 2.06 | 0.0394 | 0.62 |
| TRIM47 | sCCA1 | 382 | 24 | enet | 0.27 | 1.8e-22 | -2.07 | 0.038888 | 0.62 |
| NIP7 | sCCA3 | 300 | 23 | enet | 0.031 | 0.0011 | 2.09 | 0.03686 | 0.62 |
| MUTYH | sCCA3 | 283 | 4 | lasso | 0.045 | 0.00011 | -2.1 | 0.036 | 0.62 |
| RBM38 | sCCA3 | 671 | 1 | top1 | 0.052 | 3.3e-05 | -2.1 | 0.03599 | 0.62 |
| PIK3C2B | sCCA3 | 614 | 45 | enet | 0.12 | 2e-10 | 2.09 | 0.0367 | 0.62 |
| MBD2 | sCCA1 | 387 | 13 | lasso | 0.12 | 5.7e-10 | 2.08 | 0.03756 | 0.62 |
| CD63 | sCCA2 | 308 | 12 | lasso | 0.037 | 0.00042 | 2.08 | 0.037248 | 0.62 |
| NOV | sCCA3 | 474 | 28 | enet | 0.089 | 6.8e-08 | -2.08 | 0.03729 | 0.62 |
| TCF19 | sCCA1 | 173 | 32 | enet | 0.41 | 2.9e-36 | -2.07 | 0.03853 | 0.62 |
| FAM8A1 | sCCA3 | 355 | 46 | enet | 0.15 | 2.9e-12 | 2.1 | 0.03581 | 0.62 |
| RDX | sCCA2 | 388 | 13 | enet | 0.029 | 0.0016 | -2.08 | 0.037546 | 0.62 |
| FNBP1L | sCCA1 | 318 | 1 | top1 | 0.13 | 4.2e-11 | -2.08 | 0.0373 | 0.62 |
| PARP9 | sCCA2 | 503 | 1 | top1 | 0.086 | 1e-07 | -2.07 | 0.038242 | 0.62 |
| INHBE | sCCA1 | 334 | 4 | lasso | 0.045 | 0.00011 | 2.06 | 0.039115 | 0.62 |
| WDR89 | sCCA3 | 378 | 30 | enet | 0.087 | 8.8e-08 | 2.07 | 0.038616 | 0.62 |
| C18orf21 | sCCA1 | 437 | 5 | lasso | 0.14 | 4.4e-12 | 2.08 | 0.03761 | 0.62 |
| RPS11 | sCCA1 | 369 | 7 | lasso | 0.46 | 1.1e-42 | 2.07 | 0.038049 | 0.62 |
| RPS11 | sCCA3 | 369 | 9 | lasso | 0.36 | 2e-31 | 2.06 | 0.0391 | 0.62 |
| PGD | sCCA1 | 373 | 1 | top1 | 0.6 | 1.1e-62 | -2.06 | 0.0394 | 0.62 |
| ECM1 | sCCA3 | 279 | 8 | lasso | 0.051 | 3.8e-05 | 2.08 | 0.0379 | 0.62 |
| HAX1 | sCCA3 | 352 | 39 | enet | 0.37 | 2.8e-32 | -2.07 | 0.0381 | 0.62 |
| GUK1 | sCCA1 | 328 | 6 | lasso | 0.13 | 5.4e-11 | -2.07 | 0.0381 | 0.62 |
| DCAF1 | sCCA1 | 241 | 18 | lasso | 0.036 | 0.00054 | 2.08 | 0.0372 | 0.62 |
| ECE2 | sCCA1 | 508 | 1 | top1 | 0.23 | 1.9e-19 | -2.1 | 0.0361 | 0.62 |
| SLC26A1 | sCCA1 | 399 | 26 | enet | 0.85 | 2.7e-129 | -2.1 | 0.03581 | 0.62 |
| CDKN2A | sCCA3 | 446 | 23 | enet | 0.032 | 0.00095 | 2.09 | 0.03653 | 0.62 |
| TCF7L2 | sCCA2 | 492 | 20 | enet | 0.025 | 0.0035 | -2.06 | 0.0391 | 0.62 |
| MTG1 | sCCA3 | 341 | 29 | enet | 0.14 | 3.5e-12 | -2.07 | 0.0384 | 0.62 |
| SREK1IP1 | sCCA1 | 363 | 9 | lasso | 0.14 | 1.1e-11 | -2.07 | 0.038891 | 0.62 |
| TRIM11 | sCCA1 | 338 | 23 | enet | 0.14 | 1.1e-11 | 2.06 | 0.0396 | 0.62 |
| KIF5A | sCCA1 | 332 | 1 | top1 | 0.089 | 7e-08 | 2.09 | 0.036815 | 0.62 |
| EEF1A1 | sCCA1 | 390 | 57 | enet | 0.036 | 5e-04 | -2.07 | 0.03825 | 0.62 |
| WIPI2 | sCCA2 | 361 | 1 | top1 | 0.16 | 1.3e-13 | 2.08 | 0.037608 | 0.62 |
| GPR153 | sCCA1 | 447 | 37 | enet | 0.053 | 3.2e-05 | -2.09 | 0.0369 | 0.62 |
| DEDD | sCCA3 | 388 | 27 | enet | 0.0093 | 0.051 | -2.07 | 0.0386 | 0.62 |
| EFCAB14 | sCCA1 | 376 | 11 | lasso | 0.4 | 3.7e-35 | 2.1 | 0.036 | 0.62 |
| FBXL13 | sCCA3 | 240 | 19 | enet | 0.01 | 0.042 | 2.06 | 0.039191 | 0.62 |
| BDH1 | sCCA1 | 383 | 11 | lasso | 0.31 | 7.4e-27 | 2.1 | 0.0361 | 0.62 |
| FCMR | sCCA3 | 454 | 25 | enet | 0.038 | 0.00039 | -2.09 | 0.0365 | 0.62 |
| MAIP1 | sCCA3 | 370 | 1 | top1 | 0.17 | 4.3e-14 | -2.09 | 0.0367 | 0.62 |
| YY1AP1 | sCCA3 | 220 | 4 | lasso | 0.012 | 0.033 | 2.09 | 0.0363 | 0.62 |
| PIGX | sCCA3 | 403 | 26 | enet | 0.38 | 1.2e-33 | 2.07 | 0.03885 | 0.62 |
| TEX264 | sCCA3 | 233 | 8 | lasso | 0.52 | 6.7e-50 | 2.07 | 0.038437 | 0.62 |
| TMEM65 | sCCA1 | 529 | 42 | enet | 0.48 | 1.5e-44 | 2.09 | 0.0367 | 0.62 |
| AMOTL1 | sCCA2 | 480 | 1 | top1 | 0.067 | 2.5e-06 | 2.06 | 0.039401 | 0.62 |
| POLL | sCCA1 | 297 | 1 | top1 | 0.14 | 2.1e-11 | -2.09 | 0.0364 | 0.62 |
| CUL5 | sCCA2 | 369 | 1 | top1 | 0.023 | 0.0043 | -2.08 | 0.037623 | 0.62 |
| ATF7IP2 | sCCA1 | 611 | 35 | enet | 0.44 | 1.1e-39 | -2.09 | 0.03626 | 0.62 |
| AP1G1 | sCCA1 | 429 | 1 | top1 | 0.22 | 4.3e-18 | 2.09 | 0.03669 | 0.62 |
| AP1G1 | sCCA3 | 429 | 1 | top1 | 0.22 | 2.7e-18 | 2.09 | 0.03669 | 0.62 |
| ZNF641 | sCCA1 | 473 | 10 | lasso | 0.55 | 3.6e-54 | -2.07 | 0.038559 | 0.62 |
| SDHAF2 | sCCA2 | 368 | 1 | top1 | -0.0016 | 0.48 | 2.08 | 0.037086 | 0.62 |
| SDHAF2 | sCCA3 | 368 | 1 | top1 | 0.077 | 5.4e-07 | -2.08 | 0.03709 | 0.62 |
| SCARA3 | sCCA1 | 611 | 9 | lasso | 0.3 | 1.5e-25 | -2.1 | 0.0361 | 0.62 |
| NUDT16L1 | sCCA3 | 390 | 33 | enet | 0.0065 | 0.084 | 2.07 | 0.03883 | 0.62 |
| IRS1 | sCCA3 | 456 | 6 | lasso | 0.1 | 9.2e-09 | -2.06 | 0.0398 | 0.62 |
| UBB | sCCA2 | 291 | 1 | top1 | 0.015 | 0.019 | 2.09 | 0.03684 | 0.62 |
| NFXL1 | sCCA2 | 412 | 1 | top1 | 0.097 | 1.7e-08 | -2.07 | 0.0386 | 0.62 |
| NPAS2 | sCCA3 | 506 | 7 | enet | 0.014 | 0.021 | -2.08 | 0.0378 | 0.62 |
| LGALS4 | sCCA3 | 395 | 48 | enet | 0.17 | 2.3e-14 | -2.06 | 0.0391 | 0.62 |
| RPS21 | sCCA1 | 507 | 14 | enet | 0.094 | 2.6e-08 | -2.07 | 0.0382 | 0.62 |
| MBOAT1 | sCCA1 | 529 | 17 | lasso | 0.43 | 3.3e-39 | -2.1 | 0.03584 | 0.62 |
| PLK3 | sCCA2 | 347 | 1 | top1 | 0.07 | 1.6e-06 | 2.07 | 0.038058 | 0.62 |
| C11orf45 | sCCA3 | 551 | 15 | enet | 0.04 | 0.00024 | 2.09 | 0.03679 | 0.62 |
| TMEM167A | sCCA1 | 412 | 31 | enet | 0.086 | 1.1e-07 | 2.09 | 0.036883 | 0.62 |
| DCTN2 | sCCA2 | 336 | 1 | top1 | 0.041 | 0.00022 | 2.07 | 0.038025 | 0.62 |
| TBC1D10C | sCCA2 | 254 | 1 | top1 | 0.055 | 2e-05 | -2.1 | 0.03603 | 0.62 |
| TTLL11 | sCCA2 | 527 | 8 | lasso | 0.29 | 1.9e-24 | 2.1 | 0.035878 | 0.62 |
| C2orf69 | sCCA1 | 349 | 8 | lasso | 0.3 | 8.3e-26 | 2.09 | 0.0362 | 0.62 |
| C2orf69 | sCCA3 | 349 | 1 | top1 | 0.28 | 4.7e-24 | -2.09 | 0.0367 | 0.62 |
| EPM2AIP1 | sCCA1 | 407 | 8 | lasso | 0.19 | 6.5e-16 | -2.09 | 0.0365 | 0.62 |
| LGALS7B | sCCA2 | 399 | 8 | lasso | 0.56 | 4.9e-56 | 2.06 | 0.039801 | 0.62 |
| ELMOD2 | sCCA3 | 413 | 25 | enet | 0.029 | 0.0018 | -2.09 | 0.0362 | 0.62 |
| LACC1 | sCCA3 | 527 | 40 | enet | 0.21 | 2.2e-17 | -2.07 | 0.0389 | 0.62 |
| ACTA2-AS1 | sCCA2 | 566 | 1 | top1 | 0.045 | 0.00012 | 2.08 | 0.0379 | 0.62 |
| ZNF443 | sCCA2 | 280 | 1 | top1 | 0.26 | 1.9e-21 | 2.09 | 0.036498 | 0.62 |
| LSMEM1 | sCCA1 | 407 | 27 | enet | 0.42 | 1.9e-38 | -2.06 | 0.03898 | 0.62 |
| KMT5A | sCCA3 | 339 | 40 | enet | 0.19 | 3.4e-16 | -2.06 | 0.0396 | 0.62 |
| SOCS3 | sCCA3 | 483 | 1 | top1 | -0.0016 | 0.47 | 2.07 | 0.038462 | 0.62 |
| ATL3 | sCCA3 | 303 | 35 | enet | 0.15 | 1.5e-12 | 2.06 | 0.03932 | 0.62 |
| UBOX5 | sCCA1 | 421 | 8 | lasso | 0.43 | 2.1e-39 | -2.07 | 0.0382 | 0.62 |
| TARSL2 | sCCA2 | 388 | 5 | lasso | 0.26 | 1.3e-21 | 2.09 | 0.03669 | 0.62 |
| C19orf68 | sCCA2 | 510 | 32 | enet | 0.075 | 6.6e-07 | 2.08 | 0.037518 | 0.62 |
| IRF7 | sCCA2 | 463 | 5 | lasso | 0.15 | 1.4e-12 | 2.07 | 0.038249 | 0.62 |
| IFITM1 | sCCA1 | 325 | 6 | lasso | 0.34 | 2.5e-29 | -2.09 | 0.036514 | 0.62 |
| KRT10 | sCCA2 | 423 | 1 | top1 | 0.07 | 1.7e-06 | -2.06 | 0.039011 | 0.62 |
| NAP1L1 | sCCA1 | 493 | 14 | enet | 0.13 | 5.8e-11 | -2.06 | 0.03964 | 0.62 |
| WDR86 | sCCA2 | 523 | 41 | enet | 0.21 | 1e-17 | 2.07 | 0.038161 | 0.62 |
| ADAT2 | sCCA2 | 513 | 5 | lasso | 0.34 | 1.8e-29 | -2.09 | 0.036964 | 0.62 |
| MAN2A2 | sCCA1 | 494 | 14 | enet | 0.17 | 5.2e-14 | -2.06 | 0.03967 | 0.62 |
| ANXA6 | sCCA2 | 621 | 1 | top1 | 0.15 | 2.8e-12 | -2.07 | 0.038801 | 0.62 |
| DDI2 | sCCA2 | 478 | 13 | lasso | 0.12 | 4.6e-10 | 2.09 | 0.03705 | 0.62 |
| ZNF628 | sCCA1 | 579 | 5 | lasso | 0.071 | 1.4e-06 | 2.08 | 0.037349 | 0.62 |
| LDB1 | sCCA1 | 272 | 15 | enet | 0.11 | 2.2e-09 | 2.06 | 0.0395 | 0.62 |
| SELENOM | sCCA3 | 373 | 27 | enet | 0.18 | 3.5e-15 | 2.08 | 0.03713 | 0.62 |
| OPA1 | sCCA2 | 473 | 36 | enet | 0.18 | 1.1e-14 | -2.08 | 0.037554 | 0.62 |
| MICB | sCCA2 | 275 | 18 | lasso | 0.65 | 2.1e-70 | -2.09 | 0.03625 | 0.62 |
| ZNRD1ASP | sCCA2 | 39 | 31 | enet | 0.25 | 8.4e-21 | 2.09 | 0.03631 | 0.62 |
| ZNRD1ASP | sCCA1 | 39 | 25 | enet | 0.31 | 2.6e-26 | -2.08 | 0.03717 | 0.62 |
| HLA-G | sCCA2 | 40 | 30 | enet | 0.06 | 9.8e-06 | -2.08 | 0.03754 | 0.62 |
| RP11-563J2.3 | sCCA1 | 770 | 53 | enet | 0.3 | 1.4e-25 | 2.06 | 0.0391 | 0.62 |
| PPM1N | sCCA2 | 369 | 5 | lasso | 0.23 | 5.9e-19 | 2.08 | 0.03781 | 0.62 |
| TOMM6 | sCCA2 | 505 | 11 | lasso | 0.016 | 0.015 | 2.06 | 0.039627 | 0.62 |
| AC141586.5 | sCCA1 | 295 | 7 | lasso | 0.42 | 1.2e-37 | 2.09 | 0.03704 | 0.62 |
| FNIP1 | sCCA2 | 287 | 1 | top1 | 0.059 | 1.1e-05 | -2.09 | 0.036873 | 0.62 |
| NUTM2A-AS1 | sCCA1 | 274 | 15 | enet | 0.027 | 0.0023 | -2.09 | 0.0365 | 0.62 |
| ATXN1L | sCCA3 | 412 | 4 | lasso | 0.049 | 5.9e-05 | -2.09 | 0.03699 | 0.62 |
| DHFR | sCCA3 | 480 | 49 | enet | 0.65 | 2.5e-71 | -2.09 | 0.036812 | 0.62 |
| RP11-314N13.3 | sCCA3 | 677 | 6 | lasso | 0.056 | 1.8e-05 | 2.07 | 0.03838 | 0.62 |
| LINC00342 | sCCA2 | 107 | 10 | enet | 0.062 | 5.9e-06 | -2.08 | 0.0379 | 0.62 |
| RP4-756G23.5 | sCCA2 | 253 | 19 | enet | 0.37 | 5.6e-33 | -2.07 | 0.038264 | 0.62 |
| MTX1P1 | sCCA2 | 322 | 1 | top1 | 0.024 | 0.0036 | -2.06 | 0.03906 | 0.62 |
| RGL2 | sCCA2 | 489 | 6 | lasso | 0.014 | 0.022 | -2.07 | 0.03877 | 0.62 |
| RP4-728D4.2 | sCCA3 | 220 | 1 | top1 | 0.016 | 0.017 | -2.06 | 0.0397 | 0.62 |
| MIF | sCCA1 | 429 | 35 | enet | 0.84 | 4.5e-122 | 2.07 | 0.03806 | 0.62 |
| OR2A1-AS1 | sCCA1 | 338 | 29 | enet | 0.18 | 4.9e-15 | 2.09 | 0.03692 | 0.62 |
| RP3-330M21.5 | sCCA1 | 358 | 33 | enet | 0.1 | 8.2e-09 | -2.06 | 0.03949 | 0.62 |
| AP001258.4 | sCCA1 | 339 | 8 | lasso | 0.027 | 0.0022 | 2.09 | 0.036725 | 0.62 |
| NAIP | sCCA3 | 38 | 1 | top1 | 0.053 | 2.8e-05 | -2.06 | 0.039842 | 0.62 |
| CDK3 | sCCA1 | 402 | 7 | lasso | 0.63 | 3.9e-68 | -2.08 | 0.037491 | 0.62 |
| CDK3 | sCCA3 | 402 | 8 | lasso | 0.42 | 1.2e-37 | 2.06 | 0.039361 | 0.62 |
| AC005363.9 | sCCA1 | 444 | 28 | enet | 0.77 | 6.4e-99 | -2.08 | 0.03763 | 0.62 |
| CTD-2341M24.1 | sCCA1 | 496 | 21 | enet | 0.16 | 2.2e-13 | 2.07 | 0.03806 | 0.62 |
| KB-1460A1.5 | sCCA2 | 507 | 9 | enet | 0.02 | 0.0079 | -2.08 | 0.037511 | 0.62 |
| TIMM23 | sCCA3 | 159 | 21 | enet | 0.11 | 3e-09 | 2.1 | 0.0359 | 0.62 |
| RP3-467L1.6 | sCCA2 | 458 | 9 | lasso | 0.13 | 2.2e-11 | 2.06 | 0.0391 | 0.62 |
| RP11-250B2.6 | sCCA2 | 439 | 32 | enet | 0.25 | 4.1e-21 | -2.06 | 0.039087 | 0.62 |
| RP11-422P24.11 | sCCA2 | 327 | 23 | enet | 0.041 | 0.00021 | -2.08 | 0.037949 | 0.62 |
| RP11-47A8.5 | sCCA2 | 362 | 16 | enet | 0.08 | 3.3e-07 | -2.07 | 0.0386 | 0.62 |
| RP11-416N2.4 | sCCA3 | 371 | 1 | top1 | 0.065 | 4.1e-06 | 2.07 | 0.0383 | 0.62 |
| CWC25 | sCCA2 | 364 | 4 | lasso | 0.16 | 2e-13 | -2.09 | 0.036322 | 0.62 |
| CWC25 | sCCA1 | 364 | 4 | lasso | 0.28 | 1.1e-23 | -2.09 | 0.036981 | 0.62 |
| RP11-158H5.8 | sCCA1 | 399 | 1 | top1 | 0.34 | 2e-29 | -2.09 | 0.03669 | 0.62 |
| RP11-227G15.10 | sCCA2 | 248 | 4 | enet | 0.067 | 2.9e-06 | -2.09 | 0.036196 | 0.62 |
| RP11-61K9.3 | sCCA3 | 351 | 5 | lasso | 0.0034 | 0.15 | -2.08 | 0.0374 | 0.62 |
| ORAI1 | sCCA3 | 375 | 6 | lasso | 0.15 | 8.2e-13 | -2.09 | 0.0362 | 0.62 |
| AL133243.1 | sCCA1 | 376 | 10 | lasso | 0.47 | 8.9e-44 | 2.07 | 0.0381 | 0.62 |
| N4BP2L2-IT2 | sCCA3 | 476 | 1 | top1 | 0.11 | 1.1e-09 | -2.07 | 0.0384 | 0.62 |
| N4BP2L2-IT2 | sCCA1 | 476 | 9 | lasso | 0.23 | 3.8e-19 | -2.06 | 0.03967 | 0.62 |
| ARRDC3-AS1 | sCCA3 | 334 | 1 | top1 | 0.056 | 1.9e-05 | 2.08 | 0.037523 | 0.62 |
| IFRD1 | sCCA3 | 403 | 7 | lasso | 0.52 | 3.3e-50 | 2.05 | 0.04059 | 0.63 |
| RPUSD1 | sCCA3 | 453 | 6 | lasso | 0.12 | 2.8e-10 | -2.05 | 0.04039 | 0.63 |
| PPP2R5B | sCCA3 | 335 | 1 | top1 | 0.051 | 4.1e-05 | -2.05 | 0.0404 | 0.63 |
| LIMS2 | sCCA2 | 364 | 9 | lasso | 0.19 | 9e-16 | -2.05 | 0.0401 | 0.63 |
| MCM2 | sCCA2 | 464 | 7 | lasso | 0.011 | 0.039 | 2.04 | 0.041569 | 0.63 |
| FLT3LG | sCCA1 | 363 | 12 | enet | 0.043 | 0.00015 | -2.05 | 0.040564 | 0.63 |
| SEMA6A | sCCA1 | 586 | 15 | lasso | 0.11 | 2e-09 | -2.05 | 0.040398 | 0.63 |
| TRMT2A | sCCA3 | 557 | 38 | enet | 0.2 | 1e-16 | -2.04 | 0.04119 | 0.63 |
| RPAP1 | sCCA3 | 362 | 15 | lasso | 0.43 | 2.3e-39 | -2.04 | 0.04121 | 0.63 |
| CLIP3 | sCCA2 | 337 | 44 | enet | 0.24 | 7.3e-20 | 2.04 | 0.041526 | 0.63 |
| SUFU | sCCA3 | 333 | 4 | lasso | 0.061 | 8e-06 | -2.04 | 0.0412 | 0.63 |
| C1QBP | sCCA1 | 535 | 1 | top1 | 0.23 | 1.6e-19 | -2.05 | 0.040472 | 0.63 |
| BCL7A | sCCA2 | 336 | 1 | top1 | 0.14 | 8.6e-12 | -2.04 | 0.04148 | 0.63 |
| NT5DC3 | sCCA3 | 611 | 30 | enet | 0.44 | 3.6e-40 | -2.04 | 0.041 | 0.63 |
| HMGXB3 | sCCA2 | 571 | 6 | lasso | 0.23 | 4.8e-19 | 2.05 | 0.040509 | 0.63 |
| DNAJC16 | sCCA3 | 505 | 1 | top1 | 0.062 | 6.1e-06 | -2.05 | 0.0402 | 0.63 |
| MARC2 | sCCA2 | 404 | 1 | top1 | 0.13 | 1.1e-10 | -2.05 | 0.04022 | 0.63 |
| PPL | sCCA1 | 402 | 24 | enet | 0.072 | 1.2e-06 | -2.05 | 0.04067 | 0.63 |
| SRGN | sCCA2 | 473 | 9 | lasso | 0.015 | 0.018 | 2.05 | 0.0403 | 0.63 |
| CKS2 | sCCA1 | 497 | 54 | enet | 0.54 | 1.2e-53 | 2.04 | 0.04156 | 0.63 |
| ZBTB1 | sCCA2 | 491 | 29 | enet | 0.2 | 1.3e-16 | -2.05 | 0.0407 | 0.63 |
| RHOT1 | sCCA3 | 307 | 51 | enet | 0.22 | 1.2e-18 | -2.04 | 0.041071 | 0.63 |
| RPAIN | sCCA1 | 528 | 1 | top1 | 0.46 | 7.4e-43 | 2.05 | 0.040472 | 0.63 |
| RPAIN | sCCA3 | 528 | 1 | top1 | 0.15 | 8.5e-13 | 2.05 | 0.040472 | 0.63 |
| CCDC62 | sCCA1 | 301 | 26 | enet | 0.08 | 2.9e-07 | 2.05 | 0.040174 | 0.63 |
| KATNBL1 | sCCA3 | 451 | 1 | top1 | 0.09 | 5.9e-08 | 2.05 | 0.03998 | 0.63 |
| AVIL | sCCA2 | 354 | 1 | top1 | 0.028 | 0.0019 | -2.05 | 0.040631 | 0.63 |
| TSPAN31 | sCCA2 | 342 | 1 | top1 | 0.048 | 6.6e-05 | 2.04 | 0.041398 | 0.63 |
| PARP9 | sCCA1 | 503 | 1 | top1 | 0.13 | 2.6e-11 | 2.04 | 0.0414 | 0.63 |
| C1orf216 | sCCA2 | 243 | 5 | lasso | 0.18 | 4.9e-15 | -2.05 | 0.040376 | 0.63 |
| ZNF300 | sCCA3 | 534 | 9 | lasso | 0.0025 | 0.18 | 2.04 | 0.041487 | 0.63 |
| SPC24 | sCCA1 | 378 | 30 | enet | 0.41 | 7e-37 | -2.05 | 0.04022 | 0.63 |
| JAK1 | sCCA1 | 437 | 11 | lasso | 0.17 | 4.5e-14 | 2.05 | 0.0408 | 0.63 |
| MEMO1 | sCCA3 | 267 | 1 | top1 | 0.11 | 8.5e-10 | -2.05 | 0.0401 | 0.63 |
| ATP23 | sCCA1 | 408 | 16 | lasso | 0.92 | 2.4e-166 | -2.05 | 0.040464 | 0.63 |
| NYAP1 | sCCA2 | 303 | 1 | top1 | 0.21 | 3.6e-17 | 2.05 | 0.040236 | 0.63 |
| C11orf84 | sCCA1 | 318 | 1 | top1 | 0.096 | 2.1e-08 | -2.05 | 0.040552 | 0.63 |
| BMI1 | sCCA2 | 286 | 1 | top1 | 0.042 | 0.00019 | 2.05 | 0.0407 | 0.63 |
| FAM161A | sCCA1 | 287 | 14 | enet | 0.29 | 5.1e-25 | 2.05 | 0.04 | 0.63 |
| KRT15 | sCCA2 | 439 | 29 | enet | 0.071 | 1.3e-06 | 2.05 | 0.040724 | 0.63 |
| ZFAND4 | sCCA1 | 239 | 15 | lasso | 0.34 | 1.5e-29 | 2.04 | 0.0413 | 0.63 |
| ERCC4 | sCCA3 | 511 | 68 | enet | 0.052 | 3.2e-05 | 2.05 | 0.04031 | 0.63 |
| VSIG10 | sCCA3 | 507 | 33 | enet | 0.2 | 1.9e-16 | 2.04 | 0.0409 | 0.63 |
| C2orf88 | sCCA3 | 339 | 34 | enet | 0.0028 | 0.17 | -2.05 | 0.0405 | 0.63 |
| FAM217B | sCCA1 | 620 | 48 | enet | 4.5e-05 | 0.31 | 2.05 | 0.04064 | 0.63 |
| ZGPAT | sCCA2 | 437 | 15 | enet | 0.091 | 4.4e-08 | -2.05 | 0.040436 | 0.63 |
| PIGN | sCCA2 | 508 | 7 | lasso | 0.3 | 7.8e-26 | -2.05 | 0.0402 | 0.63 |
| TMEM229B | sCCA3 | 383 | 1 | top1 | 0.2 | 9.9e-17 | -2.05 | 0.040298 | 0.63 |
| HMGN2 | sCCA1 | 368 | 25 | enet | 0.067 | 3e-06 | 2.04 | 0.0414 | 0.63 |
| C17orf67 | sCCA3 | 457 | 22 | enet | 0.15 | 3.5e-12 | 2.04 | 0.041597 | 0.63 |
| MIF-AS1 | sCCA2 | 429 | 42 | enet | 0.84 | 1.7e-122 | -2.05 | 0.040337 | 0.63 |
| RP3-475N16.1 | sCCA1 | 362 | 1 | top1 | 0.13 | 5.8e-11 | 2.04 | 0.04149 | 0.63 |
| RP11-263K19.6 | sCCA1 | 332 | 1 | top1 | 0.18 | 1.4e-14 | -2.05 | 0.0408 | 0.63 |
| MIF | sCCA2 | 429 | 41 | enet | 0.8 | 5.1e-108 | 2.05 | 0.040105 | 0.63 |
| CCDC153 | sCCA3 | 376 | 42 | enet | 0.44 | 3e-40 | -2.05 | 0.04073 | 0.63 |
| RAC1P2 | sCCA1 | 293 | 25 | enet | 0.024 | 0.0036 | -2.05 | 0.04074 | 0.63 |
| LINC01094 | sCCA3 | 450 | 30 | enet | 0.084 | 1.7e-07 | -2.05 | 0.04018 | 0.63 |
| RP11-244O19.1 | sCCA1 | 469 | 31 | enet | 0.37 | 9.8e-33 | 2.04 | 0.04131 | 0.63 |
| TMEM249 | sCCA2 | 238 | 20 | enet | 0.26 | 3e-22 | -2.05 | 0.040447 | 0.63 |
| CTC-524C5.2 | sCCA1 | 529 | 1 | top1 | 0.36 | 8.7e-32 | 2.05 | 0.040472 | 0.63 |
| CTC-524C5.2 | sCCA3 | 529 | 1 | top1 | 0.34 | 5e-29 | 2.05 | 0.040472 | 0.63 |
| KB-226F1.2 | sCCA3 | 423 | 1 | top1 | 0.11 | 2.2e-09 | -2.05 | 0.04002 | 0.63 |
| AP000350.5 | sCCA3 | 427 | 1 | top1 | 0.073 | 1.1e-06 | 2.05 | 0.04002 | 0.63 |
| RP11-81A1.6 | sCCA1 | 574 | 6 | lasso | 0.56 | 1.8e-55 | 2.05 | 0.04011 | 0.63 |
| RP11-498C9.4 | sCCA2 | 286 | 21 | enet | 0.026 | 0.0028 | 2.04 | 0.041654 | 0.63 |
| TTC19 | sCCA1 | 277 | 1 | top1 | 0.57 | 2.7e-58 | -2.01 | 0.044895 | 0.64 |
| TYMP | sCCA1 | 318 | 9 | lasso | 0.45 | 2.9e-41 | -2.03 | 0.04187 | 0.64 |
| ZCCHC8 | sCCA2 | 321 | 19 | enet | 0.041 | 0.00022 | 2.03 | 0.042044 | 0.64 |
| MRPS10 | sCCA3 | 412 | 22 | enet | 0.62 | 5.5e-65 | 2.03 | 0.04198 | 0.64 |
| NFE2L3 | sCCA2 | 526 | 31 | enet | 0.081 | 2.4e-07 | 2.02 | 0.043315 | 0.64 |
| TAB2 | sCCA3 | 480 | 7 | lasso | 0.041 | 0.00022 | 2.03 | 0.04218 | 0.64 |
| INTS13 | sCCA2 | 516 | 1 | top1 | 0.034 | 0.00073 | -2.01 | 0.044919 | 0.64 |
| TM7SF3 | sCCA3 | 525 | 16 | lasso | 0.64 | 2.8e-69 | 2.02 | 0.0432 | 0.64 |
| PDIA5 | sCCA3 | 484 | 7 | lasso | 0.034 | 0.00073 | -2 | 0.045151 | 0.64 |
| TP53BP1 | sCCA2 | 266 | 1 | top1 | 0.21 | 3.8e-17 | -2.01 | 0.04479 | 0.64 |
| KDM5A | sCCA3 | 390 | 1 | top1 | 0.054 | 2.4e-05 | 2.03 | 0.0423 | 0.64 |
| ZNF638 | sCCA3 | 482 | 37 | enet | 0.001 | 0.25 | 2.03 | 0.0424 | 0.64 |
| TP53INP2 | sCCA3 | 323 | 1 | top1 | 0.064 | 4.4e-06 | 2.01 | 0.04399 | 0.64 |
| KHSRP | sCCA3 | 412 | 55 | enet | 0.056 | 1.6e-05 | 2.03 | 0.0426 | 0.64 |
| KHSRP | sCCA2 | 412 | 9 | lasso | 0.011 | 0.04 | 2.01 | 0.044783 | 0.64 |
| MUL1 | sCCA2 | 512 | 33 | enet | 0.45 | 3.3e-41 | -2.02 | 0.043563 | 0.64 |
| RAPGEF4 | sCCA3 | 493 | 37 | enet | 0.35 | 5.2e-30 | 2.03 | 0.0426 | 0.64 |
| MYO15A | sCCA1 | 323 | 1 | top1 | 0.37 | 3.7e-32 | -2 | 0.045029 | 0.64 |
| AGO1 | sCCA3 | 257 | 6 | enet | 0.039 | 0.00028 | 2.01 | 0.044 | 0.64 |
| LZTR1 | sCCA3 | 372 | 14 | lasso | 0.43 | 1.3e-39 | 2 | 0.04515 | 0.64 |
| CABIN1 | sCCA2 | 411 | 5 | lasso | 0.14 | 1.4e-11 | -2.02 | 0.043119 | 0.64 |
| TCF20 | sCCA1 | 389 | 1 | top1 | 0.017 | 0.013 | -2.02 | 0.04372 | 0.64 |
| KIAA0391 | sCCA1 | 368 | 29 | enet | 0.48 | 5.6e-45 | -2.02 | 0.04333 | 0.64 |
| STK4 | sCCA1 | 422 | 21 | enet | 0.22 | 4.3e-18 | -2.02 | 0.04348 | 0.64 |
| PLA2G15 | sCCA1 | 309 | 7 | lasso | 0.25 | 6.7e-21 | -2.02 | 0.04336 | 0.64 |
| RAB11A | sCCA2 | 386 | 3 | lasso | 0.012 | 0.03 | 2 | 0.04529 | 0.64 |
| ETFB | sCCA2 | 648 | 8 | lasso | 0.097 | 1.7e-08 | -2.02 | 0.043761 | 0.64 |
| PSMA2 | sCCA2 | 454 | 9 | enet | 0.023 | 0.0043 | 2.01 | 0.044558 | 0.64 |
| PRUNE2 | sCCA2 | 533 | 3 | lasso | -0.0016 | 0.48 | 2.01 | 0.043964 | 0.64 |
| RAPGEFL1 | sCCA1 | 294 | 5 | lasso | 0.64 | 1.2e-69 | -2.03 | 0.04242 | 0.64 |
| MED13 | sCCA3 | 247 | 24 | enet | 0.0052 | 0.11 | -2.02 | 0.04339 | 0.64 |
| PRKAR1A | sCCA2 | 445 | 1 | top1 | 0.042 | 0.00019 | 2.01 | 0.044034 | 0.64 |
| WSB1 | sCCA1 | 266 | 11 | lasso | 0.081 | 2.6e-07 | 2.02 | 0.043351 | 0.64 |
| SLC35F2 | sCCA1 | 401 | 37 | enet | 0.28 | 6.5e-24 | -2.03 | 0.042344 | 0.64 |
| FOXM1 | sCCA2 | 595 | 37 | enet | 0.026 | 0.0027 | -2.01 | 0.044304 | 0.64 |
| ARPC3 | sCCA3 | 224 | 6 | lasso | 0.11 | 7.4e-10 | -2.02 | 0.0434 | 0.64 |
| PAK1IP1 | sCCA1 | 469 | 3 | lasso | 0.095 | 2.2e-08 | -2 | 0.0452 | 0.64 |
| APBB3 | sCCA1 | 328 | 4 | lasso | 0.025 | 0.003 | -2.03 | 0.0423 | 0.64 |
| DBN1 | sCCA1 | 254 | 13 | enet | 0.18 | 1e-14 | 2.03 | 0.042014 | 0.64 |
| COMMD2 | sCCA1 | 477 | 1 | top1 | 0.57 | 2.6e-57 | -2.02 | 0.0433 | 0.64 |
| COMMD2 | sCCA2 | 477 | 1 | top1 | 0.29 | 1.6e-24 | -2.02 | 0.043349 | 0.64 |
| COMMD2 | sCCA3 | 477 | 1 | top1 | 0.52 | 6.2e-50 | -2.02 | 0.043349 | 0.64 |
| IGFBP2 | sCCA2 | 532 | 26 | enet | 0.14 | 6.7e-12 | -2 | 0.045 | 0.64 |
| ARHGEF2 | sCCA1 | 292 | 8 | lasso | 0.053 | 3.1e-05 | -2.02 | 0.0435 | 0.64 |
| TNFSF4 | sCCA3 | 325 | 30 | enet | 0.053 | 2.7e-05 | -2.03 | 0.0425 | 0.64 |
| IRF6 | sCCA3 | 521 | 4 | lasso | 0.067 | 2.8e-06 | 2.04 | 0.0418 | 0.64 |
| HMGN3 | sCCA2 | 423 | 8 | lasso | 0.15 | 3.1e-12 | 2.03 | 0.042194 | 0.64 |
| PROX2 | sCCA1 | 373 | 6 | lasso | 0.2 | 1.7e-16 | -2.02 | 0.04318 | 0.64 |
| ZC2HC1C | sCCA3 | 420 | 4 | lasso | 0.062 | 7e-06 | -2.03 | 0.042523 | 0.64 |
| GPSM2 | sCCA3 | 369 | 27 | enet | 0.16 | 3e-13 | -2.03 | 0.0421 | 0.64 |
| CISD1 | sCCA3 | 429 | 23 | lasso | 0.4 | 6.1e-36 | -2.02 | 0.0439 | 0.64 |
| HVCN1 | sCCA3 | 204 | 7 | enet | 0.061 | 7.3e-06 | 2.03 | 0.0419 | 0.64 |
| PEX6 | sCCA3 | 370 | 27 | enet | 0.86 | 4e-132 | 2.01 | 0.04482 | 0.64 |
| GFAP | sCCA1 | 362 | 34 | enet | 0.07 | 1.6e-06 | -2.02 | 0.043138 | 0.64 |
| LPIN3 | sCCA3 | 361 | 28 | enet | 0.46 | 3.2e-42 | -2.02 | 0.04338 | 0.64 |
| SPINK5 | sCCA2 | 442 | 39 | enet | 0.5 | 3.1e-47 | -2.02 | 0.043001 | 0.64 |
| CDC73 | sCCA2 | 343 | 12 | lasso | 0.025 | 0.0032 | 2.01 | 0.044674 | 0.64 |
| KDELC1 | sCCA1 | 689 | 4 | lasso | 0.028 | 0.0021 | -2.03 | 0.04256 | 0.64 |
| PSAT1 | sCCA1 | 423 | 7 | lasso | 0.24 | 3.9e-20 | 2.03 | 0.04284 | 0.64 |
| SRPK2 | sCCA1 | 395 | 57 | enet | 0.32 | 3e-27 | -2.01 | 0.04469 | 0.64 |
| AVIL | sCCA1 | 354 | 1 | top1 | 0.086 | 1.1e-07 | 2.02 | 0.043153 | 0.64 |
| MED4 | sCCA1 | 356 | 7 | lasso | 0.33 | 2.7e-28 | -2.03 | 0.04188 | 0.64 |
| RABEPK | sCCA2 | 322 | 11 | enet | 0.045 | 1e-04 | 2.01 | 0.044016 | 0.64 |
| RPS6 | sCCA2 | 550 | 33 | enet | 0.19 | 5.9e-16 | 2.03 | 0.04187 | 0.64 |
| FOXP4 | sCCA3 | 507 | 27 | enet | 0.05 | 5.1e-05 | -2.01 | 0.04431 | 0.64 |
| STAT4 | sCCA1 | 384 | 19 | enet | 0.5 | 3.7e-48 | -2.03 | 0.0428 | 0.64 |
| B4GALNT3 | sCCA2 | 414 | 7 | lasso | 0.19 | 6.8e-16 | 2.02 | 0.04347 | 0.64 |
| ETV6 | sCCA1 | 506 | 4 | lasso | 0.11 | 3.5e-09 | 2.01 | 0.044339 | 0.64 |
| 9-Mar | sCCA3 | 347 | 11 | enet | 0.02 | 0.0081 | -2.01 | 0.0441 | 0.64 |
| INHBE | sCCA3 | 334 | 1 | top1 | 0.03 | 0.0014 | -2.02 | 0.0429 | 0.64 |
| SLC7A1 | sCCA2 | 607 | 4 | lasso | 0.15 | 2e-12 | 2.01 | 0.04495 | 0.64 |
| RPS2 | sCCA3 | 443 | 28 | enet | 0.2 | 1.6e-16 | -2.01 | 0.04483 | 0.64 |
| ECE2 | sCCA3 | 508 | 20 | enet | 0.059 | 1.1e-05 | 2.03 | 0.04266 | 0.64 |
| HAUS6 | sCCA2 | 647 | 6 | lasso | 0.2 | 2.6e-16 | -2.02 | 0.043843 | 0.64 |
| GSN | sCCA1 | 417 | 34 | enet | 0.063 | 5.4e-06 | -2.03 | 0.04201 | 0.64 |
| INTS4 | sCCA1 | 349 | 17 | lasso | 0.48 | 1.9e-45 | 2.02 | 0.043903 | 0.64 |
| ATM | sCCA1 | 345 | 44 | enet | 0.31 | 1.2e-26 | 2.02 | 0.043833 | 0.64 |
| QDPR | sCCA1 | 458 | 1 | top1 | 0.49 | 1.1e-46 | -2.03 | 0.04237 | 0.64 |
| FAM151B | sCCA1 | 439 | 34 | enet | 0.17 | 2.6e-14 | 2.03 | 0.042467 | 0.64 |
| ZNF837 | sCCA3 | 312 | 6 | lasso | 0.13 | 1.2e-10 | -2.01 | 0.0444 | 0.64 |
| DAB2 | sCCA3 | 432 | 1 | top1 | 0.053 | 3.2e-05 | 2 | 0.045294 | 0.64 |
| PDLIM3 | sCCA1 | 540 | 47 | enet | 0.21 | 1.2e-17 | -2.02 | 0.04387 | 0.64 |
| ZNF18 | sCCA3 | 461 | 39 | enet | 0.047 | 8.5e-05 | -2.01 | 0.044514 | 0.64 |
| SUPV3L1 | sCCA3 | 530 | 14 | enet | 0.11 | 8.2e-10 | -2.01 | 0.044 | 0.64 |
| KAT6B | sCCA3 | 275 | 23 | enet | 0.059 | 1.1e-05 | -2.04 | 0.0418 | 0.64 |
| RAB11FIP1 | sCCA1 | 215 | 23 | enet | 0.11 | 2.3e-09 | 2.01 | 0.0442 | 0.64 |
| DUSP23 | sCCA2 | 513 | 9 | lasso | 0.022 | 0.0056 | 2.02 | 0.042956 | 0.64 |
| CDC42SE2 | sCCA3 | 270 | 17 | enet | 0.15 | 6.7e-13 | -2.01 | 0.044728 | 0.64 |
| C1QC | sCCA1 | 485 | 27 | enet | 0.034 | 0.00075 | 2 | 0.0453 | 0.64 |
| ABR | sCCA3 | 444 | 32 | enet | 0.27 | 1.1e-22 | -2.03 | 0.042107 | 0.64 |
| WDR4 | sCCA1 | 521 | 4 | lasso | 0.2 | 1.1e-16 | -2.01 | 0.04462 | 0.64 |
| SPTBN4 | sCCA3 | 377 | 25 | enet | 0.076 | 6.4e-07 | -2.04 | 0.0418 | 0.64 |
| ASB16 | sCCA3 | 355 | 1 | top1 | 0.17 | 6e-14 | 2.02 | 0.043861 | 0.64 |
| JAK1 | sCCA2 | 437 | 1 | top1 | 0.037 | 0.00044 | 2 | 0.044993 | 0.64 |
| C1orf74 | sCCA1 | 513 | 4 | lasso | 0.056 | 1.7e-05 | 2.03 | 0.0421 | 0.64 |
| FRS2 | sCCA3 | 561 | 27 | enet | 0.17 | 8.9e-14 | -2.02 | 0.0429 | 0.64 |
| RNF169 | sCCA2 | 459 | 1 | top1 | 0.037 | 0.00046 | 2.03 | 0.04267 | 0.64 |
| RPL27A | sCCA3 | 494 | 28 | enet | 0.058 | 1.3e-05 | 2.02 | 0.04366 | 0.64 |
| ATP23 | sCCA2 | 408 | 13 | lasso | 0.87 | 1.7e-135 | -2.02 | 0.043349 | 0.64 |
| MTMR10 | sCCA3 | 375 | 11 | lasso | 0.25 | 7e-21 | 2.01 | 0.04406 | 0.64 |
| RAB4B | sCCA1 | 429 | 51 | enet | 0.015 | 0.019 | 2.02 | 0.043107 | 0.64 |
| ATG4B | sCCA2 | 277 | 1 | top1 | 0.32 | 1.7e-27 | -2.02 | 0.043 | 0.64 |
| RAB24 | sCCA1 | 315 | 9 | lasso | 0.37 | 2.9e-32 | -2 | 0.045301 | 0.64 |
| B3GNT2 | sCCA2 | 357 | 24 | enet | -0.0016 | 0.48 | -2.03 | 0.0424 | 0.64 |
| SLC30A1 | sCCA3 | 417 | 23 | enet | 0.033 | 0.00091 | 2.01 | 0.0449 | 0.64 |
| RCAN2 | sCCA2 | 508 | 8 | lasso | 0.052 | 3.7e-05 | -2.01 | 0.044632 | 0.64 |
| AGAP5 | sCCA2 | 252 | 1 | top1 | 0.15 | 9.7e-13 | 2.02 | 0.0434 | 0.64 |
| ESRRA | sCCA2 | 354 | 1 | top1 | 0.035 | 6e-04 | 2.03 | 0.042209 | 0.64 |
| GMPPB | sCCA2 | 318 | 1 | top1 | 0.7 | 2.6e-81 | -2.03 | 0.042674 | 0.64 |
| GMPPB | sCCA3 | 318 | 1 | top1 | 0.75 | 2.1e-92 | 2.03 | 0.042674 | 0.64 |
| GMPPB | sCCA1 | 318 | 1 | top1 | 0.74 | 7.8e-91 | -2.03 | 0.0427 | 0.64 |
| CCS | sCCA3 | 257 | 7 | lasso | 0.042 | 2e-04 | -2.02 | 0.04326 | 0.64 |
| ASPHD1 | sCCA1 | 176 | 8 | lasso | 0.11 | 7.8e-10 | -2.03 | 0.0428 | 0.64 |
| MYO1D | sCCA1 | 483 | 18 | enet | 0.13 | 3.6e-11 | 2.03 | 0.042381 | 0.64 |
| SUZ12 | sCCA1 | 307 | 1 | top1 | 0.071 | 1.5e-06 | -2.02 | 0.043351 | 0.64 |
| R3HDM2 | sCCA1 | 332 | 31 | enet | 0.061 | 7.9e-06 | 2.04 | 0.04182 | 0.64 |
| PSMG4 | sCCA1 | 612 | 4 | lasso | 0.69 | 1.2e-78 | 2.03 | 0.04199 | 0.64 |
| ZNF572 | sCCA2 | 507 | 1 | top1 | 0.051 | 4.2e-05 | -2.01 | 0.044707 | 0.64 |
| C12orf60 | sCCA3 | 365 | 41 | enet | 0.47 | 2.5e-43 | -2.03 | 0.0428 | 0.64 |
| TNFAIP8L3 | sCCA1 | 431 | 22 | enet | 0.11 | 2.2e-09 | -2.02 | 0.04292 | 0.64 |
| KREMEN1 | sCCA1 | 387 | 4 | lasso | 0.52 | 3.1e-50 | 2.02 | 0.04326 | 0.64 |
| TM2D3 | sCCA2 | 437 | 1 | top1 | 0.16 | 4e-13 | -2.02 | 0.04334 | 0.64 |
| TMEM173 | sCCA2 | 267 | 18 | enet | 0.22 | 1.5e-18 | 2.02 | 0.043189 | 0.64 |
| RBM33 | sCCA3 | 577 | 8 | enet | -0.0031 | 0.83 | -2.03 | 0.042434 | 0.64 |
| LRRC37B | sCCA1 | 296 | 1 | top1 | 0.12 | 3.1e-10 | 2.02 | 0.043351 | 0.64 |
| NRBP2 | sCCA2 | 283 | 1 | top1 | 0.067 | 2.6e-06 | -2.03 | 0.042276 | 0.64 |
| RBM11 | sCCA2 | 447 | 10 | lasso | 0.081 | 2.4e-07 | 2.03 | 0.04227 | 0.64 |
| UBE2H | sCCA1 | 402 | 1 | top1 | 0.12 | 4.3e-10 | -2.03 | 0.04213 | 0.64 |
| PDZD7 | sCCA3 | 414 | 3 | lasso | 0.0065 | 0.085 | 2.03 | 0.0425 | 0.64 |
| GCNT1 | sCCA1 | 664 | 68 | enet | 0.06 | 8.7e-06 | 2.02 | 0.04358 | 0.64 |
| HDAC2 | sCCA1 | 369 | 23 | enet | 0.063 | 5.4e-06 | -2 | 0.04534 | 0.64 |
| ZNF418 | sCCA3 | 471 | 6 | lasso | 0.085 | 1.2e-07 | 2.01 | 0.0443 | 0.64 |
| PDLIM7 | sCCA1 | 264 | 1 | top1 | 0.47 | 2.1e-43 | -2.02 | 0.043847 | 0.64 |
| ZNF790 | sCCA2 | 283 | 17 | enet | 0.05 | 4.5e-05 | 2.01 | 0.044257 | 0.64 |
| ADH5 | sCCA2 | 499 | 33 | enet | 0.22 | 3.1e-18 | 2.01 | 0.04462 | 0.64 |
| GJC2 | sCCA1 | 327 | 1 | top1 | 0.074 | 8.3e-07 | 2.02 | 0.0435 | 0.64 |
| RN7SKP80 | sCCA1 | 416 | 27 | enet | 0.21 | 7.7e-18 | 2.02 | 0.04331 | 0.64 |
| COL5A2 | sCCA1 | 323 | 1 | top1 | 0.19 | 1.9e-15 | 2.03 | 0.0426 | 0.64 |
| DXO | sCCA1 | 220 | 19 | enet | 0.36 | 1.6e-31 | -2 | 0.04519 | 0.64 |
| ITPRIPL2 | sCCA2 | 421 | 1 | top1 | 0.063 | 5.3e-06 | -2.02 | 0.04377 | 0.64 |
| ALG3 | sCCA1 | 508 | 1 | top1 | 0.1 | 8.7e-09 | -2.01 | 0.0442 | 0.64 |
| MIF-AS1 | sCCA1 | 429 | 52 | enet | 0.89 | 1.8e-147 | 2.02 | 0.04338 | 0.64 |
| AC093818.1 | sCCA3 | 496 | 5 | lasso | 0.024 | 0.0036 | -2.02 | 0.0438 | 0.64 |
| MEMO1P1 | sCCA2 | 497 | 1 | top1 | -3.80E-05 | 0.32 | 2 | 0.04507 | 0.64 |
| LTA | sCCA2 | 288 | 42 | enet | 0.089 | 7e-08 | 2.01 | 0.04432 | 0.64 |
| PTGES3P3 | sCCA3 | 353 | 5 | lasso | -0.0012 | 0.42 | -2.02 | 0.04332 | 0.64 |
| AC079250.1 | sCCA2 | 360 | 12 | enet | 0.0027 | 0.18 | 2 | 0.0451 | 0.64 |
| CTD-2666L21.1 | sCCA1 | 291 | 3 | lasso | 0.14 | 9.9e-12 | -2.03 | 0.042744 | 0.64 |
| RPS15AP38 | sCCA2 | 577 | 1 | top1 | 0.017 | 0.013 | -2.02 | 0.043086 | 0.64 |
| FAM200B | sCCA3 | 558 | 26 | enet | 0.085 | 1.3e-07 | 2.03 | 0.04244 | 0.64 |
| GATS | sCCA1 | 286 | 4 | lasso | 0.074 | 8.5e-07 | -2.01 | 0.04485 | 0.64 |
| MIF | sCCA3 | 429 | 28 | enet | 0.75 | 2.3e-93 | 2.03 | 0.04217 | 0.64 |
| FAM13A-AS1 | sCCA1 | 411 | 1 | top1 | 0.21 | 2.4e-17 | 2.03 | 0.04201 | 0.64 |
| AP001372.2 | sCCA2 | 413 | 71 | enet | 0.073 | 1.1e-06 | 2.01 | 0.044791 | 0.64 |
| CTSO | sCCA2 | 470 | 3 | lasso | 0.2 | 6.8e-17 | 2 | 0.04516 | 0.64 |
| ZNF625 | sCCA1 | 295 | 3 | lasso | 0.075 | 7.7e-07 | -2 | 0.045253 | 0.64 |
| AC068831.6 | sCCA3 | 487 | 34 | enet | 0.049 | 6e-05 | -2.02 | 0.04357 | 0.64 |
| RP11-192M23.1 | sCCA1 | 585 | 34 | enet | 0.13 | 2.2e-11 | 2.01 | 0.0442 | 0.64 |
| RP11-89K11.1 | sCCA1 | 372 | 5 | lasso | 0.38 | 4.1e-33 | 2 | 0.04532 | 0.64 |
| RP3-467N11.1 | sCCA3 | 306 | 3 | lasso | 0.059 | 1e-05 | -2.03 | 0.0423 | 0.64 |
| SNAI3-AS1 | sCCA2 | 382 | 17 | enet | 0.53 | 1.9e-52 | -2.02 | 0.04303 | 0.64 |
| CTC-559E9.5 | sCCA3 | 295 | 11 | enet | 0.052 | 3.6e-05 | 2.02 | 0.0438 | 0.64 |
| RP11-686D22.3 | sCCA1 | 369 | 7 | lasso | 0.36 | 8.2e-32 | 2.01 | 0.044577 | 0.64 |
| AC005387.3 | sCCA1 | 379 | 1 | top1 | 0.025 | 0.0034 | -2.04 | 0.041831 | 0.64 |
| RP11-124D2.7 | sCCA2 | 482 | 49 | enet | 0.063 | 5.5e-06 | 2 | 0.0451 | 0.64 |
| RP11-245J9.6 | sCCA1 | 569 | 6 | lasso | 0.13 | 3.5e-11 | 2.03 | 0.042 | 0.64 |
| CTD-2555O16.4 | sCCA1 | 492 | 1 | top1 | 0.05 | 4.7e-05 | -2.01 | 0.0446 | 0.64 |
| RP11-731C17.2 | sCCA1 | 336 | 29 | enet | 0.27 | 6.7e-23 | -2.02 | 0.043 | 0.64 |
| CTD-2036P10.6 | sCCA2 | 275 | 27 | enet | 0.057 | 1.5e-05 | -2.03 | 0.04252 | 0.64 |
| LLNLR-268E12.1 | sCCA1 | 402 | 1 | top1 | 0.14 | 1.4e-11 | 2.02 | 0.043848 | 0.64 |
| RP11-348N5.9 | sCCA1 | 513 | 12 | enet | 0.0023 | 0.19 | -2.01 | 0.0447 | 0.64 |
| RP3-424M6.4 | sCCA1 | 230 | 1 | top1 | 0.11 | 2.9e-09 | -2.01 | 0.044341 | 0.64 |
| RBM12B-AS1 | sCCA3 | 436 | 1 | top1 | 0.054 | 2.5e-05 | 2.02 | 0.04287 | 0.64 |
| CTC-444N24.7 | sCCA3 | 502 | 6 | lasso | 0.049 | 5.3e-05 | 2.03 | 0.0426 | 0.64 |
| RP11-96D1.8 | sCCA3 | 312 | 5 | enet | 0.056 | 1.6e-05 | 2.03 | 0.04216 | 0.64 |
| LLNLR-245B6.1 | sCCA3 | 329 | 5 | lasso | 0.05 | 4.8e-05 | 2.01 | 0.0446 | 0.64 |
| PHTF2 | sCCA1 | 517 | 1 | top1 | 0.043 | 0.00016 | -1.99 | 0.04661 | 0.65 |
| PNKP | sCCA3 | 345 | 1 | top1 | 0.2 | 6.3e-17 | 1.97 | 0.0483 | 0.65 |
| MRPS10 | sCCA1 | 412 | 25 | enet | 0.79 | 4.7e-106 | -1.99 | 0.04626 | 0.65 |
| NFE2L3 | sCCA1 | 526 | 1 | top1 | 0.24 | 6.8e-20 | -1.99 | 0.04672 | 0.65 |
| BCAR1 | sCCA3 | 377 | 19 | enet | 0.07 | 1.5e-06 | 1.99 | 0.04688 | 0.65 |
| WDR3 | sCCA3 | 403 | 10 | lasso | 0.035 | 6e-04 | -1.98 | 0.0481 | 0.65 |
| ELOVL1 | sCCA3 | 452 | 26 | enet | 0.062 | 6.3e-06 | -1.98 | 0.0479 | 0.65 |
| GOLGA5 | sCCA2 | 525 | 7 | lasso | 0.13 | 3e-11 | 1.97 | 0.0486 | 0.65 |
| PICALM | sCCA2 | 484 | 8 | lasso | 0.18 | 1.1e-14 | -1.98 | 0.047695 | 0.65 |
| BCKDHB | sCCA3 | 465 | 1 | top1 | 0.072 | 1.2e-06 | -1.99 | 0.04681 | 0.65 |
| WDFY1 | sCCA1 | 474 | 30 | enet | 0.071 | 1.4e-06 | -1.97 | 0.0487 | 0.65 |
| POMGNT1 | sCCA2 | 343 | 30 | enet | 0.073 | 1.1e-06 | 1.99 | 0.046957 | 0.65 |
| HECTD1 | sCCA1 | 316 | 35 | enet | 0.063 | 5.4e-06 | 1.99 | 0.04691 | 0.65 |
| PGC | sCCA3 | 514 | 43 | enet | 0.00033 | 0.3 | -1.98 | 0.04754 | 0.65 |
| CRKL | sCCA3 | 405 | 14 | enet | 0.048 | 6.6e-05 | 1.98 | 0.04819 | 0.65 |
| DDTL | sCCA2 | 424 | 36 | enet | 0.54 | 6e-54 | 1.97 | 0.048581 | 0.65 |
| TOMM22 | sCCA2 | 373 | 5 | lasso | 0.041 | 0.00022 | 2 | 0.046005 | 0.65 |
| L3MBTL2 | sCCA1 | 259 | 1 | top1 | 0.17 | 3e-14 | -2 | 0.04581 | 0.65 |
| L3MBTL2 | sCCA3 | 259 | 1 | top1 | 0.075 | 6.6e-07 | -2 | 0.04581 | 0.65 |
| POLR3H | sCCA2 | 297 | 1 | top1 | 0.14 | 1.4e-11 | 1.99 | 0.046365 | 0.65 |
| TRIP11 | sCCA3 | 500 | 23 | lasso | 0.22 | 3.5e-18 | 2 | 0.046031 | 0.65 |
| HM13 | sCCA1 | 245 | 1 | top1 | 0.28 | 9.5e-24 | -1.99 | 0.04648 | 0.65 |
| TANGO6 | sCCA1 | 308 | 10 | lasso | 0.19 | 1.1e-15 | -1.99 | 0.04664 | 0.65 |
| LMF1 | sCCA3 | 441 | 4 | lasso | 0.38 | 5.4e-34 | -1.97 | 0.04848 | 0.65 |
| STUB1 | sCCA1 | 447 | 38 | enet | 0.14 | 9.2e-12 | -1.99 | 0.04616 | 0.65 |
| C16orf62 | sCCA2 | 463 | 8 | lasso | 0.15 | 7.4e-13 | -2 | 0.04604 | 0.65 |
| C16orf62 | sCCA1 | 463 | 13 | lasso | 0.21 | 3.2e-17 | 1.97 | 0.04902 | 0.65 |
| ZNF106 | sCCA1 | 366 | 11 | lasso | 0.059 | 1.1e-05 | 1.98 | 0.04793 | 0.65 |
| KLHDC4 | sCCA3 | 468 | 21 | enet | 0.6 | 4e-62 | 1.99 | 0.04703 | 0.65 |
| CNTD2 | sCCA1 | 397 | 1 | top1 | 0.056 | 1.7e-05 | -2 | 0.045913 | 0.65 |
| GRB10 | sCCA2 | 532 | 1 | top1 | 0.067 | 2.6e-06 | -1.97 | 0.04858 | 0.65 |
| SH3PXD2A | sCCA1 | 423 | 1 | top1 | 0.13 | 4.9e-11 | 1.97 | 0.0485 | 0.65 |
| SH3D19 | sCCA3 | 279 | 5 | lasso | 0.086 | 1.1e-07 | 1.98 | 0.04815 | 0.65 |
| CCDC34 | sCCA1 | 391 | 10 | lasso | 0.072 | 1.1e-06 | 1.99 | 0.046564 | 0.65 |
| EHD1 | sCCA2 | 331 | 1 | top1 | 0.1 | 7.6e-09 | 2 | 0.045754 | 0.65 |
| CAMKK2 | sCCA3 | 397 | 47 | enet | 0.12 | 1.3e-10 | 1.97 | 0.0486 | 0.65 |
| GAPDH | sCCA1 | 356 | 1 | top1 | 0.18 | 7.3e-15 | -2 | 0.045575 | 0.65 |
| BYSL | sCCA2 | 472 | 21 | enet | 0.084 | 1.6e-07 | -1.99 | 0.047115 | 0.65 |
| TTL | sCCA1 | 413 | 7 | lasso | 0.14 | 6.5e-12 | 1.99 | 0.0469 | 0.65 |
| IL18R1 | sCCA2 | 568 | 34 | enet | 0.2 | 7.6e-17 | 1.99 | 0.0463 | 0.65 |
| MSH6 | sCCA3 | 360 | 27 | enet | 0.49 | 7.7e-46 | -1.97 | 0.049 | 0.65 |
| UCHL5 | sCCA3 | 374 | 7 | lasso | 0.05 | 4.5e-05 | 1.98 | 0.0481 | 0.65 |
| RAB3GAP2 | sCCA2 | 378 | 16 | enet | 0.091 | 5e-08 | 1.98 | 0.048018 | 0.65 |
| CISD1 | sCCA1 | 429 | 9 | lasso | 0.61 | 6.9e-64 | 2 | 0.046 | 0.65 |
| SLC25A35 | sCCA2 | 449 | 24 | enet | 0.06 | 9.4e-06 | -1.99 | 0.046044 | 0.65 |
| GPCPD1 | sCCA2 | 619 | 1 | top1 | 0.011 | 0.036 | -1.99 | 0.04634 | 0.65 |
| TSPAN8 | sCCA3 | 431 | 24 | enet | 0.03 | 0.0014 | 1.99 | 0.0469 | 0.65 |
| PTPN12 | sCCA1 | 372 | 68 | enet | 0.52 | 2.4e-50 | 1.99 | 0.0469 | 0.65 |
| PTPN12 | sCCA2 | 372 | 64 | enet | 0.51 | 4.3e-49 | -1.98 | 0.047506 | 0.65 |
| YWHAH | sCCA2 | 463 | 10 | lasso | 0.15 | 2.8e-12 | 1.99 | 0.047136 | 0.65 |
| CCDC136 | sCCA2 | 340 | 1 | top1 | 0.17 | 6.8e-14 | 1.98 | 0.047397 | 0.65 |
| ATP1B2 | sCCA3 | 494 | 23 | enet | 0.2 | 1.3e-16 | 1.98 | 0.047429 | 0.65 |
| LPIN3 | sCCA1 | 361 | 8 | lasso | 0.44 | 1.3e-40 | -1.98 | 0.04765 | 0.65 |
| CASP9 | sCCA3 | 505 | 9 | lasso | 0.43 | 9.5e-39 | 2 | 0.046 | 0.65 |
| RTN3 | sCCA2 | 303 | 17 | enet | 0.0052 | 0.11 | -1.98 | 0.04716 | 0.65 |
| TSPAN2 | sCCA3 | 485 | 23 | enet | 0.13 | 9.4e-11 | -1.97 | 0.0488 | 0.65 |
| PSRC1 | sCCA2 | 441 | 12 | lasso | 0.27 | 2.9e-22 | 1.97 | 0.048661 | 0.65 |
| OS9 | sCCA3 | 349 | 1 | top1 | 0.098 | 1.5e-08 | -1.99 | 0.0463 | 0.65 |
| PPIL1 | sCCA3 | 538 | 37 | enet | 0.36 | 1.2e-31 | -1.98 | 0.04823 | 0.65 |
| RSL24D1 | sCCA3 | 439 | 79 | enet | 0.44 | 2.9e-40 | -1.99 | 0.04638 | 0.65 |
| EXOC6 | sCCA2 | 457 | 7 | lasso | 0.029 | 0.0015 | 1.98 | 0.048 | 0.65 |
| AOX1 | sCCA1 | 477 | 37 | enet | 0.07 | 1.6e-06 | -2 | 0.0454 | 0.65 |
| ANXA3 | sCCA3 | 466 | 17 | enet | 0.46 | 2.5e-42 | -1.99 | 0.04713 | 0.65 |
| ZSCAN29 | sCCA2 | 268 | 8 | lasso | 0.35 | 6.7e-30 | 1.97 | 0.04841 | 0.65 |
| FTO | sCCA2 | 463 | 9 | lasso | 0.14 | 3.8e-12 | -1.99 | 0.04664 | 0.65 |
| TRIM65 | sCCA3 | 388 | 5 | lasso | 0.2 | 6.6e-17 | 2 | 0.046015 | 0.65 |
| MYO1F | sCCA1 | 424 | 6 | lasso | 0.35 | 3.7e-30 | -1.98 | 0.048117 | 0.65 |
| RPS11 | sCCA2 | 369 | 1 | top1 | 0.29 | 1.7e-24 | 1.98 | 0.047666 | 0.65 |
| TUFT1 | sCCA2 | 337 | 44 | enet | 0.099 | 1.2e-08 | 2 | 0.046003 | 0.65 |
| HAX1 | sCCA2 | 352 | 29 | enet | 0.27 | 5.1e-23 | -1.98 | 0.048093 | 0.65 |
| IQSEC1 | sCCA1 | 490 | 5 | lasso | 0.11 | 1.1e-09 | -1.99 | 0.047 | 0.65 |
| TMF1 | sCCA3 | 452 | 6 | lasso | 0.18 | 3.2e-15 | -1.99 | 0.046326 | 0.65 |
| PIK3R1 | sCCA1 | 474 | 54 | enet | 0.034 | 0.00065 | 1.99 | 0.046102 | 0.65 |
| GIN1 | sCCA3 | 318 | 22 | enet | 0.049 | 6.2e-05 | 1.97 | 0.048425 | 0.65 |
| RMND5B | sCCA1 | 392 | 1 | top1 | 0.045 | 0.00012 | -1.99 | 0.04607 | 0.65 |
| BOD1 | sCCA1 | 572 | 8 | lasso | 0.22 | 1e-18 | 1.99 | 0.046843 | 0.65 |
| GSN | sCCA2 | 417 | 5 | lasso | 0.06 | 9.1e-06 | -2 | 0.045975 | 0.65 |
| NPAT | sCCA1 | 351 | 1 | top1 | 0.31 | 2.6e-26 | -1.97 | 0.048796 | 0.65 |
| NPAT | sCCA2 | 351 | 1 | top1 | 0.1 | 4.9e-09 | -1.97 | 0.048796 | 0.65 |
| HYOU1 | sCCA2 | 378 | 9 | lasso | 0.055 | 2e-05 | 1.97 | 0.04833 | 0.65 |
| EI24 | sCCA1 | 545 | 47 | enet | 0.29 | 3.7e-24 | 1.97 | 0.048849 | 0.65 |
| IPMK | sCCA2 | 429 | 12 | lasso | 0.021 | 0.0061 | -1.98 | 0.0473 | 0.65 |
| AP1S3 | sCCA2 | 453 | 12 | enet | 0.063 | 5.1e-06 | 1.97 | 0.0491 | 0.65 |
| UBP1 | sCCA3 | 362 | 5 | lasso | 0.0015 | 0.23 | -2 | 0.045497 | 0.65 |
| ABCA6 | sCCA3 | 466 | 28 | enet | 0.016 | 0.017 | -1.98 | 0.047535 | 0.65 |
| MIA3 | sCCA1 | 316 | 42 | enet | 0.27 | 4.2e-23 | 2 | 0.0455 | 0.65 |
| RBM45 | sCCA2 | 474 | 1 | top1 | 0.12 | 6.2e-10 | 1.99 | 0.0461 | 0.65 |
| DYRK1A | sCCA1 | 498 | 7 | lasso | 0.16 | 9.7e-14 | -2 | 0.045728 | 0.65 |
| AP3S2 | sCCA1 | 415 | 6 | lasso | 0.8 | 1.5e-107 | -1.99 | 0.04632 | 0.65 |
| AP3S2 | sCCA2 | 415 | 6 | lasso | 0.8 | 2.5e-108 | -1.98 | 0.04769 | 0.65 |
| CCDC58 | sCCA1 | 472 | 29 | enet | 0.26 | 1e-21 | 1.99 | 0.0466 | 0.65 |
| CCNF | sCCA2 | 301 | 1 | top1 | 0.073 | 1e-06 | -1.99 | 0.04706 | 0.65 |
| DHRS3 | sCCA3 | 274 | 22 | enet | 0.074 | 8.6e-07 | -1.97 | 0.0486 | 0.65 |
| TMCO4 | sCCA1 | 559 | 42 | enet | 0.19 | 1.3e-15 | -1.97 | 0.0487 | 0.65 |
| KRTCAP2 | sCCA1 | 341 | 1 | top1 | 0.028 | 0.002 | 1.99 | 0.0465 | 0.65 |
| SGO2 | sCCA2 | 485 | 1 | top1 | 0.026 | 0.0028 | -1.98 | 0.0482 | 0.65 |
| INPPL1 | sCCA2 | 300 | 19 | enet | 0.053 | 2.8e-05 | -1.99 | 0.046742 | 0.65 |
| BORCS5 | sCCA2 | 531 | 36 | enet | 0.26 | 1.2e-21 | -2 | 0.045899 | 0.65 |
| ATP23 | sCCA3 | 408 | 1 | top1 | 0.8 | 2.2e-108 | 1.99 | 0.0463 | 0.65 |
| CCNDBP1 | sCCA2 | 290 | 9 | lasso | 0.29 | 6.3e-25 | -1.98 | 0.04732 | 0.65 |
| CCNDBP1 | sCCA1 | 290 | 11 | lasso | 0.29 | 5.8e-25 | -1.97 | 0.04904 | 0.65 |
| KATNAL2 | sCCA1 | 484 | 13 | lasso | 0.38 | 1.9e-33 | -1.99 | 0.04654 | 0.65 |
| DUS2 | sCCA2 | 256 | 5 | lasso | 0.3 | 3.3e-25 | 1.98 | 0.04721 | 0.65 |
| ZNF610 | sCCA2 | 488 | 4 | lasso | 0.027 | 0.0024 | -2 | 0.04553 | 0.65 |
| EVPL | sCCA1 | 395 | 71 | enet | 0.098 | 1.3e-08 | 1.98 | 0.047319 | 0.65 |
| SFTPB | sCCA1 | 477 | 1 | top1 | 0.12 | 4.8e-10 | 1.98 | 0.0477 | 0.65 |
| MFSD7 | sCCA1 | 383 | 1 | top1 | 0.37 | 4.2e-32 | -1.99 | 0.04662 | 0.65 |
| MFSD7 | sCCA2 | 383 | 1 | top1 | 0.19 | 1.5e-15 | -1.99 | 0.04662 | 0.65 |
| FOS | sCCA1 | 433 | 1 | top1 | 0.0038 | 0.14 | -1.97 | 0.04851 | 0.65 |
| CD14 | sCCA3 | 344 | 14 | enet | 0.12 | 5.7e-10 | 1.99 | 0.047092 | 0.65 |
| ATP6V0E2 | sCCA2 | 262 | 4 | lasso | 0.49 | 1.7e-46 | -1.98 | 0.047532 | 0.65 |
| HOPX | sCCA1 | 503 | 16 | lasso | 0.34 | 5.8e-29 | 1.98 | 0.04784 | 0.65 |
| MBOAT1 | sCCA3 | 529 | 28 | enet | 0.33 | 3.5e-28 | 2 | 0.04558 | 0.65 |
| C1QA | sCCA3 | 481 | 18 | enet | 0.031 | 0.0012 | -1.98 | 0.0475 | 0.65 |
| RMDN1 | sCCA1 | 439 | 12 | lasso | 0.67 | 7.2e-76 | -1.98 | 0.0478 | 0.65 |
| ZNF543 | sCCA3 | 504 | 1 | top1 | 0.15 | 1.5e-12 | -2 | 0.046 | 0.65 |
| SAMD4B | sCCA2 | 443 | 22 | enet | 0.1 | 7e-09 | 1.99 | 0.046534 | 0.65 |
| ARID3B | sCCA1 | 348 | 1 | top1 | 0.29 | 1.9e-24 | -1.98 | 0.04808 | 0.65 |
| ARID3B | sCCA2 | 348 | 1 | top1 | 0.17 | 2.4e-14 | -1.98 | 0.04808 | 0.65 |
| ARID3B | sCCA3 | 348 | 1 | top1 | 0.15 | 8.9e-13 | 1.98 | 0.04808 | 0.65 |
| EPHB3 | sCCA3 | 439 | 12 | enet | 0.074 | 8.1e-07 | 1.98 | 0.047683 | 0.65 |
| CEND1 | sCCA2 | 429 | 1 | top1 | 0.037 | 0.00046 | 1.98 | 0.047601 | 0.65 |
| TARSL2 | sCCA3 | 388 | 19 | enet | 0.23 | 4e-19 | 2 | 0.04588 | 0.65 |
| IFITM1 | sCCA2 | 325 | 6 | lasso | 0.2 | 1.1e-16 | 1.98 | 0.047644 | 0.65 |
| C15orf41 | sCCA1 | 560 | 15 | enet | 0.23 | 1.5e-19 | -1.98 | 0.0478 | 0.65 |
| PDE2A | sCCA3 | 307 | 1 | top1 | 0.029 | 0.0016 | -1.98 | 0.048 | 0.65 |
| FAM83G | sCCA1 | 176 | 4 | lasso | 0.068 | 2.3e-06 | -1.97 | 0.048915 | 0.65 |
| ZNF799 | sCCA2 | 275 | 19 | enet | 0.042 | 0.00019 | 1.97 | 0.048797 | 0.65 |
| 5-Mar | sCCA2 | 305 | 1 | top1 | 0.18 | 4.1e-15 | 1.99 | 0.0464 | 0.65 |
| 5-Mar | sCCA3 | 305 | 1 | top1 | 0.22 | 4.4e-18 | -1.99 | 0.0464 | 0.65 |
| ZNF876P | sCCA2 | 179 | 35 | enet | 0.034 | 0.00074 | 2 | 0.0455 | 0.65 |
| RPS6KL1 | sCCA3 | 379 | 1 | top1 | 0.035 | 0.00064 | 1.97 | 0.04851 | 0.65 |
| ZNF568 | sCCA2 | 283 | 22 | enet | 0.39 | 1.8e-34 | -1.99 | 0.046272 | 0.65 |
| CCDC152 | sCCA2 | 315 | 10 | lasso | 0.048 | 6.8e-05 | 1.97 | 0.048349 | 0.65 |
| MICB | sCCA1 | 275 | 39 | enet | 0.74 | 8.3e-91 | 1.98 | 0.04775 | 0.65 |
| LINC01291 | sCCA3 | 456 | 90 | enet | 0.72 | 2.5e-86 | 1.98 | 0.0477 | 0.65 |
| TMEM240 | sCCA2 | 283 | 15 | enet | 0.028 | 0.002 | -2 | 0.045528 | 0.65 |
| PPM1N | sCCA1 | 369 | 6 | lasso | 0.56 | 2.2e-56 | -1.98 | 0.048228 | 0.65 |
| NUDT19 | sCCA2 | 399 | 1 | top1 | 0.31 | 7.3e-27 | 1.98 | 0.047722 | 0.65 |
| RP11-1023L17.1 | sCCA3 | 417 | 1 | top1 | 0.03 | 0.0014 | -1.98 | 0.047961 | 0.65 |
| RP11-278A23.1 | sCCA2 | 493 | 6 | lasso | 0.42 | 9.8e-38 | 2 | 0.04593 | 0.65 |
| DHFR | sCCA1 | 480 | 37 | enet | 0.79 | 1.4e-103 | 2 | 0.045559 | 0.65 |
| DOC2GP | sCCA2 | 257 | 40 | enet | 0.2 | 2.9e-16 | 1.99 | 0.046969 | 0.65 |
| RP11-196G11.6 | sCCA3 | 240 | 3 | lasso | 0.049 | 5.8e-05 | 1.98 | 0.04781 | 0.65 |
| RPS2P32 | sCCA1 | 409 | 6 | lasso | 0.1 | 7.7e-09 | 1.99 | 0.04641 | 0.65 |
| FAM200B | sCCA2 | 558 | 1 | top1 | 0.19 | 4.8e-16 | 1.98 | 0.04715 | 0.65 |
| NCK1-AS1 | sCCA1 | 334 | 81 | enet | 0.23 | 3.9e-19 | -1.99 | 0.0463 | 0.65 |
| RP11-285F7.2 | sCCA1 | 388 | 10 | lasso | 0.18 | 2.7e-15 | -2 | 0.0455 | 0.65 |
| N4BP2L2 | sCCA3 | 484 | 1 | top1 | 0.063 | 5.3e-06 | -1.98 | 0.0473 | 0.65 |
| RAD51-AS1 | sCCA1 | 307 | 1 | top1 | 0.062 | 6.3e-06 | -1.99 | 0.04688 | 0.65 |
| RP11-727A23.5 | sCCA2 | 403 | 41 | enet | 0.0075 | 0.071 | 1.97 | 0.048639 | 0.65 |
| TMEM161B-AS1 | sCCA3 | 258 | 10 | lasso | 0.52 | 2e-50 | 2 | 0.045866 | 0.65 |
| ZNF436-AS1 | sCCA3 | 310 | 15 | lasso | 0.69 | 1.3e-79 | 1.97 | 0.049 | 0.65 |
| LINC02021 | sCCA3 | 325 | 26 | enet | 0.77 | 2.8e-98 | -1.99 | 0.046406 | 0.65 |
| RP11-933H2.4 | sCCA3 | 423 | 1 | top1 | 0.062 | 6.6e-06 | -1.98 | 0.047551 | 0.65 |
| CCDC150P1 | sCCA1 | 344 | 1 | top1 | 0.1 | 5.6e-09 | -2 | 0.04563 | 0.65 |
| PRC1-AS1 | sCCA3 | 488 | 8 | lasso | 0.33 | 2.8e-28 | -1.98 | 0.04717 | 0.65 |
| PRC1-AS1 | sCCA1 | 488 | 6 | lasso | 0.44 | 6e-40 | 1.97 | 0.04831 | 0.65 |
| RP11-89K11.1 | sCCA3 | 372 | 4 | lasso | 0.38 | 5.1e-34 | 1.97 | 0.04899 | 0.65 |
| RBM15B | sCCA1 | 245 | 1 | top1 | 0.3 | 4.2e-25 | 1.97 | 0.0484 | 0.65 |
| NSFP1 | sCCA2 | 64 | 31 | enet | 0.74 | 1.3e-91 | 1.98 | 0.047872 | 0.65 |
| LINC00562 | sCCA1 | 382 | 1 | top1 | 0.42 | 4.1e-38 | -2 | 0.04591 | 0.65 |
| ZNF587B | sCCA1 | 483 | 44 | enet | 0.27 | 1.9e-22 | 1.98 | 0.047786 | 0.65 |
| PTOV1-AS2 | sCCA3 | 348 | 1 | top1 | 0.38 | 3.1e-33 | -1.97 | 0.0483 | 0.65 |
| RP1-151F17.2 | sCCA1 | 551 | 7 | lasso | 0.35 | 7e-31 | 1.98 | 0.04817 | 0.65 |
| RP11-339B21.13 | sCCA1 | 285 | 13 | enet | 0.12 | 4.6e-10 | -1.97 | 0.04908 | 0.65 |
| RP5-1159O4.2 | sCCA1 | 713 | 12 | lasso | 0.49 | 3.2e-46 | -1.98 | 0.04789 | 0.65 |
| RP11-449P15.2 | sCCA2 | 329 | 11 | lasso | 0.15 | 9.2e-13 | 1.99 | 0.046708 | 0.65 |
| AP000350.5 | sCCA1 | 427 | 44 | enet | 0.91 | 3.2e-158 | 2 | 0.04589 | 0.65 |
| RP11-486I11.2 | sCCA1 | 467 | 4 | lasso | 0.066 | 3.2e-06 | 1.98 | 0.04786 | 0.65 |
| RP11-348N5.9 | sCCA2 | 513 | 27 | enet | 0.023 | 0.0045 | -1.99 | 0.0465 | 0.65 |
| FLJ38576 | sCCA3 | 584 | 6 | lasso | 0.24 | 3e-20 | 2 | 0.04578 | 0.65 |
| RP11-546D6.3 | sCCA1 | 323 | 20 | enet | 0.14 | 1.3e-11 | -1.99 | 0.046048 | 0.65 |
| CTC-205M6.1 | sCCA1 | 377 | 11 | lasso | 0.52 | 4.1e-50 | -1.98 | 0.047533 | 0.65 |
| RP11-384M20.1 | sCCA2 | 352 | 1 | top1 | 0.077 | 5.3e-07 | -1.98 | 0.047902 | 0.65 |
| LA16c-313F4.1 | sCCA2 | 481 | 5 | lasso | 0.023 | 0.0043 | 1.97 | 0.04841 | 0.65 |
| CYBA | sCCA2 | 379 | 1 | top1 | 0.24 | 2.4e-20 | 1.96 | 0.04962 | 0.66 |
| EIF2B3 | sCCA3 | 313 | 1 | top1 | 0.12 | 2.8e-10 | -1.96 | 0.0498 | 0.66 |
| STUB1 | sCCA2 | 447 | 21 | enet | 0.026 | 0.0028 | -1.97 | 0.04941 | 0.66 |
| KLHDC4 | sCCA1 | 468 | 24 | enet | 0.62 | 3e-65 | 1.97 | 0.04931 | 0.66 |
| UBE2R2 | sCCA1 | 306 | 14 | lasso | 0.1 | 4.8e-09 | -1.96 | 0.04966 | 0.66 |
| ZBTB17 | sCCA1 | 403 | 32 | enet | 0.025 | 0.0031 | 1.96 | 0.0499 | 0.66 |
| RPL5 | sCCA1 | 272 | 17 | enet | 0.1 | 4.3e-09 | 1.97 | 0.0494 | 0.66 |
| PEX6 | sCCA1 | 370 | 36 | enet | 0.91 | 4.7e-161 | 1.97 | 0.0493 | 0.66 |
| LRRFIP1 | sCCA1 | 517 | 10 | lasso | 0.22 | 3.2e-18 | 1.96 | 0.0498 | 0.66 |
| OPRL1 | sCCA2 | 312 | 17 | enet | 0.32 | 2.1e-27 | 1.97 | 0.049261 | 0.66 |
| PAK4 | sCCA1 | 412 | 33 | enet | 0.61 | 1.5e-64 | 1.97 | 0.049237 | 0.66 |
| RAB25 | sCCA1 | 306 | 21 | enet | 0.11 | 7.4e-10 | 1.96 | 0.0498 | 0.66 |
| USO1 | sCCA2 | 459 | 11 | lasso | 0.2 | 2.9e-16 | 1.96 | 0.04951 | 0.66 |
| MYO1F | sCCA2 | 424 | 4 | lasso | 0.23 | 2.2e-19 | 1.97 | 0.049209 | 0.66 |
| ACP1 | sCCA1 | 373 | 33 | enet | 0.16 | 1.3e-13 | 1.96 | 0.0495 | 0.66 |
| AMMECR1L | sCCA1 | 382 | 1 | top1 | 0.22 | 6.4e-18 | 1.97 | 0.0492 | 0.66 |
| SFRP2 | sCCA2 | 435 | 1 | top1 | 0.094 | 2.8e-08 | -1.96 | 0.04959 | 0.66 |
| RPL7L1 | sCCA3 | 351 | 32 | enet | 0.55 | 3.3e-54 | 1.96 | 0.04968 | 0.66 |
| LAMTOR1 | sCCA1 | 245 | 20 | enet | 0.062 | 6e-06 | 1.97 | 0.049142 | 0.66 |
| SPTBN4 | sCCA1 | 377 | 11 | lasso | 0.098 | 1.3e-08 | -1.96 | 0.049556 | 0.66 |
| FGFR4 | sCCA1 | 384 | 14 | lasso | 0.6 | 4.1e-63 | 1.96 | 0.049639 | 0.66 |
| CLP1 | sCCA1 | 390 | 1 | top1 | 0.074 | 8.6e-07 | 1.96 | 0.049618 | 0.66 |
| CEP19 | sCCA1 | 403 | 13 | lasso | 0.56 | 8.4e-57 | 1.96 | 0.0499 | 0.66 |
| C16orf91 | sCCA1 | 486 | 1 | top1 | 0.035 | 0.00061 | -1.96 | 0.04975 | 0.66 |
| DEAF1 | sCCA3 | 470 | 1 | top1 | 0.0061 | 0.091 | -1.97 | 0.04926 | 0.66 |
| PTRF | sCCA1 | 255 | 1 | top1 | 0.089 | 6.1e-08 | 1.97 | 0.049205 | 0.66 |
| CCDC84 | sCCA2 | 378 | 27 | enet | 0.059 | 9.9e-06 | -1.97 | 0.049363 | 0.66 |
| SKOR1 | sCCA2 | 349 | 13 | lasso | 0.56 | 6.6e-57 | -1.97 | 0.04935 | 0.66 |
| LRRK2 | sCCA2 | 540 | 13 | lasso | 0.44 | 1.5e-40 | -1.96 | 0.049609 | 0.66 |
| FUT11 | sCCA2 | 242 | 35 | enet | 0.29 | 3.5e-24 | -1.96 | 0.0498 | 0.66 |
| SNHG14 | sCCA3 | 398 | 10 | enet | 0.11 | 7.5e-10 | -1.96 | 0.04982 | 0.66 |
| FNDC10 | sCCA3 | 281 | 5 | lasso | 0.017 | 0.014 | -1.97 | 0.0494 | 0.66 |
| LINC00886 | sCCA3 | 446 | 42 | enet | 0.68 | 7.3e-78 | -1.96 | 0.04975 | 0.66 |
| RP11-440L14.1 | sCCA3 | 389 | 17 | enet | 0.03 | 0.0014 | 1.96 | 0.04991 | 0.66 |
| AC005363.9 | sCCA3 | 444 | 43 | enet | 0.72 | 3.3e-86 | -1.96 | 0.04953 | 0.66 |
| HNRNPLP2 | sCCA1 | 406 | 6 | lasso | 0.25 | 2.8e-21 | 1.96 | 0.0498 | 0.66 |
| RP11-686D22.7 | sCCA3 | 376 | 7 | lasso | 0.2 | 2.6e-16 | 1.96 | 0.049807 | 0.66 |
| RP11-95D17.1 | sCCA2 | 536 | 10 | lasso | 0.64 | 5.7e-69 | -1.96 | 0.0495 | 0.66 |
| RP11-95D17.1 | sCCA3 | 536 | 15 | enet | 0.49 | 7.8e-47 | 1.96 | 0.0495 | 0.66 |
| CWC25 | sCCA3 | 364 | 4 | lasso | 0.2 | 1.2e-16 | -1.96 | 0.049629 | 0.66 |
| RP5-1142A6.10 | sCCA1 | 391 | 36 | enet | 0.51 | 8.1e-49 | -1.96 | 0.04949 | 0.66 |
| CTD-2013N17.6 | sCCA2 | 407 | 5 | lasso | 0.12 | 6.4e-10 | 1.97 | 0.049369 | 0.66 |
