# Supplementary material for: Genomic Structural Equation Modeling Combined With Post‐GWAS Analysis Identifies Two Risk Gene Loci and Functionally Sensitive Genes Associated With Cardiac Conduction Block
Source: Genet Res (Camb). 2026 Jan 14;2026:1063531. doi: 10.1155/genr/1063531 (PMC12801132; doi:10.1155/genr/1063531)
Supplement: Supplementary file 1 — Supporting Information Additional supporting information can be found online in the Supporting Information section. [file GENR-2026-1063531-s001.zip › Table S3.docx]

| ens_gene_id | eqtlfile | pip | in_cred_set | ID |
| --- | --- | --- | --- | --- |
| ENSG00000073331 | sCCA1 | 0.00679 | 0 | ALPK1 |
| ENSG00000073331 | sCCA2 | 0.00715 | 0 | ALPK1 |
| ENSG00000073331 | sCCA3 | 0.00799 | 0 | ALPK1 |
| ENSG00000079150 | sCCA1 | 0.952 | 1 | FKBP7 |
| ENSG00000079150 | sCCA2 | 0.995 | 1 | FKBP7 |
| ENSG00000079150 | sCCA3 | 0.996 | 1 | FKBP7 |
| ENSG00000079156 | sCCA1 | 0.0707 | 1 | OSBPL6 |
| ENSG00000079156 | sCCA2 | 0.0102 | 0 | OSBPL6 |
| ENSG00000079156 | sCCA3 | 0.0152 | 0 | OSBPL6 |
| ENSG00000080345 | sCCA1 | 0.0862 | 1 | RIF1 |
| ENSG00000080345 | sCCA2 | 0.0866 | 1 | RIF1 |
| ENSG00000080345 | sCCA3 | 0.0774 | 1 | RIF1 |
| ENSG00000115963 | sCCA2 | 0.0792 | 1 | RND3 |
| ENSG00000116095 | sCCA1 | 0.0172 | 0 | PLEKHA3 |
| ENSG00000116095 | sCCA2 | 0.0087 | 0 | PLEKHA3 |
| ENSG00000116095 | sCCA3 | 0.00867 | 0 | PLEKHA3 |
| ENSG00000123609 | sCCA1 | 0.0807 | 1 | NMI |
| ENSG00000123609 | sCCA2 | 0.0857 | 1 | NMI |
| ENSG00000123609 | sCCA3 | 0.062 | 0 | NMI |
| ENSG00000123610 | sCCA1 | 0.0922 | 1 | TNFAIP6 |
| ENSG00000123610 | sCCA2 | 0.0768 | 1 | TNFAIP6 |
| ENSG00000123610 | sCCA3 | 0.103 | 1 | TNFAIP6 |
| ENSG00000130396 | sCCA3 | 0.0328 | 0 | AFDN |
| ENSG00000138658 | sCCA1 | 0.00645 | 0 | ZGRF1 |
| ENSG00000138658 | sCCA2 | 0.00735 | 0 | ZGRF1 |
| ENSG00000138658 | sCCA3 | 0.00678 | 0 | ZGRF1 |
| ENSG00000138660 | sCCA1 | 0.0179 | 0 | AP1AR |
| ENSG00000138660 | sCCA2 | 0.0234 | 0 | AP1AR |
| ENSG00000138660 | sCCA3 | 0.0148 | 0 | AP1AR |
| ENSG00000145365 | sCCA1 | 0.0145 | 0 | TIFA |
| ENSG00000145365 | sCCA2 | 0.0181 | 0 | TIFA |
| ENSG00000145365 | sCCA3 | 0.00644 | 0 | TIFA |
| ENSG00000155636 | sCCA1 | 0.0306 | 1 | RBM45 |
| ENSG00000155636 | sCCA2 | 0.0119 | 0 | RBM45 |
| ENSG00000155636 | sCCA3 | 0.00856 | 0 | RBM45 |
| ENSG00000163510 | sCCA1 | 0.0123 | 0 | CWC22 |
| ENSG00000163510 | sCCA2 | 0.00782 | 0 | CWC22 |
| ENSG00000163510 | sCCA3 | 0.0108 | 0 | CWC22 |
| ENSG00000170522 | sCCA1 | 0.0179 | 0 | ELOVL6 |
| ENSG00000170522 | sCCA2 | 0.02 | 0 | ELOVL6 |
| ENSG00000174720 | sCCA1 | 0.00671 | 0 | LARP7 |
| ENSG00000174720 | sCCA2 | 0.00682 | 0 | LARP7 |
| ENSG00000174720 | sCCA3 | 0.00651 | 0 | LARP7 |
| ENSG00000174749 | sCCA1 | 0.0671 | 1 | C4orf32 |
| ENSG00000174749 | sCCA2 | 0.0662 | 1 | C4orf32 |
| ENSG00000174749 | sCCA3 | 0.0635 | 1 | C4orf32 |
| ENSG00000180228 | sCCA1 | 0.0125 | 0 | PRKRA |
| ENSG00000180228 | sCCA2 | 0.0212 | 1 | PRKRA |
| ENSG00000180228 | sCCA3 | 0.0394 | 1 | PRKRA |
| ENSG00000183091 | sCCA1 | 0.0795 | 0 | NEB |
| ENSG00000183091 | sCCA2 | 0.0766 | 0 | NEB |
| ENSG00000184898 | sCCA1 | 0.0824 | 1 | RBM43 |
| ENSG00000184898 | sCCA2 | 0.0967 | 1 | RBM43 |
| ENSG00000184898 | sCCA3 | 0.0744 | 1 | RBM43 |
| ENSG00000187231 | sCCA1 | 0.015 | 0 | SESTD1 |
| ENSG00000187231 | sCCA2 | 0.013 | 0 | SESTD1 |
| ENSG00000187231 | sCCA3 | 0.013 | 0 | SESTD1 |
| ENSG00000196659 | sCCA2 | 0.0162 | 0 | TTC30B |
| ENSG00000196659 | sCCA3 | 0.00891 | 0 | TTC30B |
| ENSG00000197557 | sCCA1 | 0.00979 | 0 | TTC30A |
| ENSG00000197557 | sCCA3 | 0.0106 | 0 | TTC30A |
| ENSG00000204311 | sCCA1 | 0.0135 | 0 | DFNB59 |
| ENSG00000204311 | sCCA2 | 0.0203 | 0 | DFNB59 |
| ENSG00000204311 | sCCA3 | 0.0096 | 0 | DFNB59 |
| ENSG00000213066 | sCCA1 | 0.0443 | 0 | FGFR1OP |
| ENSG00000213066 | sCCA2 | 0.0452 | 0 | FGFR1OP |
| ENSG00000213066 | sCCA3 | 0.081 | 1 | FGFR1OP |
| ENSG00000223960 | sCCA1 | 0.0132 | 0 | AC009948.5 |
| ENSG00000223960 | sCCA2 | 0.0114 | 0 | AC009948.5 |
| ENSG00000223960 | sCCA3 | 0.0206 | 1 | AC009948.5 |
| ENSG00000225808 | sCCA3 | 0.0223 | 1 | DNAJC19P5 |
| ENSG00000237298 | sCCA1 | 0.0683 | 1 | TTN-AS1 |
| ENSG00000237298 | sCCA3 | 0.0136 | 0 | TTN-AS1 |
| ENSG00000241741 | sCCA1 | 0.00819 | 0 | RPL7AP30 |
| ENSG00000241741 | sCCA3 | 0.00837 | 0 | RPL7AP30 |
| ENSG00000260526 | sCCA1 | 0.0733 | 1 | RP11-73K9.2 |
| ENSG00000260526 | sCCA2 | 0.00684 | 0 | RP11-73K9.2 |
| ENSG00000260526 | sCCA3 | 0.00743 | 0 | RP11-73K9.2 |
