# Supplementary material for: Genomic Structural Equation Modeling Combined With Post‐GWAS Analysis Identifies Two Risk Gene Loci and Functionally Sensitive Genes Associated With Cardiac Conduction Block
Source: Genet Res (Camb). 2026 Jan 14;2026:1063531. doi: 10.1155/genr/1063531 (PMC12801132; doi:10.1155/genr/1063531)
Supplement: Supplementary file 1 — Supporting Information Additional supporting information can be found online in the Supporting Information section. [file GENR-2026-1063531-s001.zip › Table S4.docx]

| trait | mean_chisq | lambda_gc | intercept | intercept_se | ratio | ratio_se | h2_observed | h2_observed_se | h2_Z | h2_p |
| --- | --- | --- | --- | --- | --- | --- | --- | --- | --- | --- |
| SSS | 1.079 | 1.07 | 1.03 | 0.007 | 0.453 | 0.0937 | 0.0046 | 0.001 | 4.051 | 5.089e-05 |
| FKBP7 | 2.107 | 1.91 | 1.5 | 0.281 | 0.459 | 0.254 | 1.013 | 0.381 | 2.658 | 0.007 |
| CCDC141 | 4.37 | 2.13 | 0.10 | 0.519 | -0.266 | 0.153 | 7.450 | 1.540 | 4.837 | 1.315e-06 |
| ESR2 | 9.84 | 3.68 | -1.67 | 1.049 | -0.303 | 0.118 | 16.754 | 2.503 | 6.693 | 2.182e-11 |
| SH3PXD2A | 14.05 | 10.98 | 12.20 | 2.684 | 0.858 | 0.205 | 0.360 | 3.941 | 0.091 | 0.927 |
| IAVB | 1.08 | 1.07 | 1.03 | 0.0089 | 0.454 | 0.104 | 0.005 | 0.001 | 4.35 | 1.359e-05 |
| IIAVB | 1.03 | 1.02 | 1.00 | 0.0058 | 0.242 | 0.192 | 0.002 | 0.0009 | 2.420 | 0.0155 |
| IIIAVB | 1.06 | 1.06 | 1.00 | 0.0072 | 0.0982 | 0.117 | 0.0058 | 0.001 | 5.82 | 5.631e-09 |
| LBBB | 1.07 | 1.05 | 1.02 | 0.0092 | 0.337 | 0.130 | 0.005 | 0.001 | 4.346 | 1.383e-05 |
| RBBB | 1.02 | 1.02 | 1.00 | 0.0078 | 0.345 | 0.333 | 0.001 | 0.001 | 1.432 | 0.151 |
