# Supplementary material for: Genomic Structural Equation Modeling Combined With Post‐GWAS Analysis Identifies Two Risk Gene Loci and Functionally Sensitive Genes Associated With Cardiac Conduction Block
Source: Genet Res (Camb). 2026 Jan 14;2026:1063531. doi: 10.1155/genr/1063531 (PMC12801132; doi:10.1155/genr/1063531)
Supplement: Supplementary file 1 — Supporting Information Additional supporting information can be found online in the Supporting Information section. [file GENR-2026-1063531-s001.zip › Table S5.docx]

| outcome | exposure | egger_intercept | se | pval |
| --- | --- | --- | --- | --- |
| LBBB | ESR2 | 0.014637817 | 0.010785759 | 0.179429815 |
| F1 | ESR2 | 0.004199818 | 0.00413241 | 0.313430478 |
| RBBB | ESR2 | -0.032446189 | 0.018879241 | 0.090444032 |
| SSS | ESR2 | 0.01119721 | 0.009119821 | 0.223822489 |
| IIIAVB | ESR2 | 0.00478209 | 0.008593397 | 0.579733825 |
| IAVB | ESR2 | 0.019163566 | 0.010966905 | 0.085149894 |
| IIAVB | ESR2 | 0.035027143 | 0.014998198 | 0.02252423 |
| LBBB | CCDC141 | 0.036313635 | 0.019587846 | 0.072993332 |
| F1 | CCDC141 | -0.012545577 | 0.008047203 | 0.129484976 |
| RBBB | CCDC141 | 0.014290592 | 0.033745467 | 0.674776659 |
| SSS | CCDC141 | -0.054710691 | 0.014242304 | 0.000508899 |
| IIIAVB | CCDC141 | -0.02703691 | 0.012447697 | 0.036919773 |
| IAVB | CCDC141 | -0.049421963 | 0.014655571 | 0.001872601 |
| IIAVB | CCDC141 | -0.049907844 | 0.028640988 | 0.090455208 |
| LBBB | SH3PXD2A | 0.006118801 | 0.006876482 | 0.375028759 |
| F1 | SH3PXD2A | -0.006360173 | 0.003269235 | 0.053813059 |
| RBBB | SH3PXD2A | -0.012282953 | 0.012885275 | 0.342036417 |
| SSS | SH3PXD2A | -0.004188267 | 0.005294923 | 0.430170397 |
| IIIAVB | SH3PXD2A | -0.011385562 | 0.006657667 | 0.08926651 |
| IAVB | SH3PXD2A | -0.026845085 | 0.014143644 | 0.059575203 |
| IIAVB | SH3PXD2A | -0.019765159 | 0.018413769 | 0.284786111 |

**A.**

| exposure | outcome | snp_r2.exposure | snp_r2.outcome | correct_causal_direction | steiger_pval |
| --- | --- | --- | --- | --- | --- |
| SH3PXD2A | LBBB | 0.65 | 0.0002 | TRUE | 0 |
| SH3PXD2A | F1 | 0.58 | 0.0090 | TRUE | 0 |
| SH3PXD2A | RBBB | 0.65 | 0.00073 | TRUE | 0 |
| SH3PXD2A | SSS | 0.65 | 0.00036 | TRUE | 0 |
| SH3PXD2A | IIIAVB | 0.65 | 0.00056 | TRUE | 0 |
| SH3PXD2A | IAVB | 0.65 | 0.0025 | TRUE | 0 |
| SH3PXD2A | IIAVB | 0.65 | 0.0014 | TRUE | 0 |
| FKBP7 | LBBB | 0.0011 | 1.27e-07 | TRUE | 1.25e-08 |
| FKBP7 | F1 | 0.0011 | 0.00017 | TRUE | 0.0025 |
| FKBP7 | RBBB | 0.0011 | 2.095e-05 | TRUE | 6.36e-07 |
| FKBP7 | SSS | 0.0011 | 8.74e-06 | TRUE | 1.26e-07 |
| FKBP7 | IIIAVB | 0.0011 | 3.048e-06 | TRUE | 4.003e-08 |
| FKBP7 | IAVB | 0.0011 | 1.382e-05 | TRUE | 2.55e-07 |
| FKBP7 | IIAVB | 0.0011 | 1.96e-05 | TRUE | 4.75e-07 |
| CCDC141 | LBBB | 0.090 | 9.05e-05 | TRUE | 0 |
| CCDC141 | F1 | 0.076 | 0.001634 | TRUE | 4.34e-271 |
| CCDC141 | RBBB | 0.090 | 0.00021 | TRUE | 0 |
| CCDC141 | SSS | 0.093 | 0.00019 | TRUE | 0 |
| CCDC141 | IIIAVB | 0.093 | 0.00010 | TRUE | 0 |
| CCDC141 | IAVB | 0.093 | 0.00013 | TRUE | 0 |
| CCDC141 | IIAVB | 0.093 | 0.00014 | TRUE | 0 |
| ESR2 | LBBB | 0.24 | 0.00017 | TRUE | 0 |
| ESR2 | F1 | 0.21 | 0.00481 | TRUE | 0 |
| ESR2 | RBBB | 0.24 | 0.00037 | TRUE | 0 |
| ESR2 | SSS | 0.24 | 0.00039 | TRUE | 0 |
| ESR2 | IIIAVB | 0.24 | 0.00028 | TRUE | 0 |
| ESR2 | IAVB | 0.24 | 0.00044 | TRUE | 0 |
| ESR2 | IIAVB | 0.24 | 0.00030 | TRUE | 0 |

**B.**
