# Supplementary material for: Genomic Structural Equation Modeling Combined With Post‐GWAS Analysis Identifies Two Risk Gene Loci and Functionally Sensitive Genes Associated With Cardiac Conduction Block
Source: Genet Res (Camb). 2026 Jan 14;2026:1063531. doi: 10.1155/genr/1063531 (PMC12801132; doi:10.1155/genr/1063531)
Supplement: Supplementary file 1 — Supporting Information Additional supporting information can be found online in the Supporting Information section. [file GENR-2026-1063531-s001.zip › Table S6.docx]

**A.**

| outcome | exposure | egger_intercept | se | pval |
| --- | --- | --- | --- | --- |
| IIIAVB | APOL1 | 0.092 | 0.05 | 0.08 |
| IAVB | APOL1 | -0.021 | 0.03 | 0.52 |
| LBBB | APOL1 | 0.008 | 0.04 | 0.83 |
| RBBB | APOL1 | 0.012 | 0.04 | 0.79 |
| IIAVB | APOL1 | -0.049 | 0.05 | 0.38 |
| SSS | APOL1 | 0.063 | 0.04 | 0.18 |

**B.**

| exposure | outcome | snp_r2.exposure | snp_r2.outcome | correct_causal_direction | steiger_pval |
| --- | --- | --- | --- | --- | --- |
| APOL1 | IIIAVB | 0.11 | 0.00051 | TRUE | 0 |
| APOL1 | IAVB | 0.11 | 0.00011 | TRUE | 0 |
| APOL1 | LBBB | 0.11 | 0.00011 | TRUE | 0 |
| APOL1 | RBBB | 0.11 | 0.00029 | TRUE | 0 |
| APOL1 | IIAVB | 0.11 | 0.00021 | TRUE | 0 |
| APOL1 | SSS | 0.11 | 0.00015 | TRUE | 0 |
