# Supplementary material for: Genomic Structural Equation Modeling Combined With Post‐GWAS Analysis Identifies Two Risk Gene Loci and Functionally Sensitive Genes Associated With Cardiac Conduction Block
Source: Genet Res (Camb). 2026 Jan 14;2026:1063531. doi: 10.1155/genr/1063531 (PMC12801132; doi:10.1155/genr/1063531)
Supplement: Supplementary file 1 — Supporting Information Additional supporting information can be found online in the Supporting Information section. [file GENR-2026-1063531-s001.zip › Table S7.docx]

**A**

| outcome | exposure | method | nsnp | b | se | pval |
| --- | --- | --- | --- | --- | --- | --- |
| CKD stage V | APOL1 | Inverse variance weighted | 23 | 0.47 | 0.201 | 0.018 |
| CKD stage III | APOL1 | Inverse variance weighted | 27 | 0.20 | 0.085 | 0.014 |
| CKD stage IV | APOL1 | Inverse variance weighted | 26 | 0.21 | 0.150 | 0.143 |
| CKD stage I | APOL1 | Inverse variance weighted | 30 | -0.01 | 0.070 | 0.782 |

**B**

| id.outcome | outcome | exposure | egger_intercept | se | pval |
| --- | --- | --- | --- | --- | --- |
| GCST90476125 | CKD stage V | APOL1 | 0.017 | 0.086 | 0.842 |
| GCST90476127 | CKD stage III | APOL1 | 0.025 | 0.027 | 0.363 |
| GCST90476129 | CKD stage IV | APOL1 | 0.028 | 0.050 | 0.577 |
| GCST90478531 | CKD stage I | APOL1 | 0.044 | 0.024 | 0.077 |

| id.outcome | exposure | outcome | snp_r2.exposure | snp_r2.outcome | correct_causal_direction | steiger_pval |
| --- | --- | --- | --- | --- | --- | --- |
| GCST90476125 | APOL1 | CKD stage V | 0.115 | 0.002 | TRUE | 0 |
| GCST90476127 | APOL1 | CKD stage III | 0.122 | 0.001 | TRUE | 0 |
| GCST90476129 | APOL1 | CKD stage IV | 0.120 | 0.001 | TRUE | 0 |
| GCST90478531 | APOL1 | CKD stage I | 0.145 | 0.0006 | TRUE | 0 |

**C**
