# Supplementary material for: Genomic Structural Equation Modeling Combined With Post‐GWAS Analysis Identifies Two Risk Gene Loci and Functionally Sensitive Genes Associated With Cardiac Conduction Block
Source: Genet Res (Camb). 2026 Jan 14;2026:1063531. doi: 10.1155/genr/1063531 (PMC12801132; doi:10.1155/genr/1063531)
Supplement: Supplementary file 1 — Supporting Information Additional supporting information can be found online in the Supporting Information section. [file GENR-2026-1063531-s001.zip › Table S8.docx]

**A**

| id.exposure | outcome | exposure | method | nsnp | b | se | pval |
| --- | --- | --- | --- | --- | --- | --- | --- |
| GCST90476125 | LBBB | Stage V CKD | Inverse variance weighted | 93 | 0.086 | 0.029 | 0.002 |
| GCST90476125 | IAVB | Stage V CKD | Inverse variance weighted | 93 | -0.017 | 0.023 | 0.440 |
| GCST90476125 | IIIAVB | Stage V CKD | Inverse variance weighted | 93 | -0.005 | 0.031 | 0.859 |
| GCST90476125 | SSS | Stage V CKD | Inverse variance weighted | 93 | -0.191 | 0.035 | 5.992e-08 |
| GCST90476125 | IIAVB | Stage V CKD | Inverse variance weighted | 93 | -0.195 | 0.037 | 1.373e-07 |
| GCST90476125 | RBBB | Stage V CKD | Inverse variance weighted | 93 | 0.189 | 0.026 | 2.105e-12 |
| GCST90476127 | LBBB | Stage 3 CKD | Inverse variance weighted | 31 | 0.267 | 0.103 | 0.009 |
| GCST90476127 | IAVB | Stage 3 CKD | Inverse variance weighted | 31 | 0.0726 | 0.082 | 0.377 |
| GCST90476127 | IIIAVB | Stage 3 CKD | Inverse variance weighted | 31 | 0.233 | 0.105 | 0.027 |
| GCST90476127 | SSS | Stage 3 CKD | Inverse variance weighted | 31 | -0.158 | 0.116 | 0.173 |
| GCST90476127 | IIAVB | Stage 3 CKD | Inverse variance weighted | 31 | -0.153 | 0.132 | 0.246 |
| GCST90476127 | RBBB | Stage 3 CKD | Inverse variance weighted | 31 | 0.494 | 0.096 | 3.087e-07 |

**B**

| outcome | exposure | egger_intercept | se | pval |
| --- | --- | --- | --- | --- |
| LBBB | Stage V CKD | -0.008 | 0.023 | 0.703 |
| IAVB | Stage V CKD | -0.023 | 0.018 | 0.210 |
| IIIAVB | Stage V CKD | -0.035 | 0.025 | 0.164 |
| SSS | Stage V CKD | -0.018 | 0.028 | 0.519 |
| IIAVB | Stage V CKD | -0.017 | 0.029 | 0.558 |
| RBBB | Stage V CKD | -0.024 | 0.021 | 0.252 |
| LBBB | Stage 3 CKD | 0.106 | 0.036 | 0.006 |
| IAVB | Stage 3 CKD | -0.051 | 0.028 | 0.081 |
| IIIAVB | Stage 3 CKD | 0.063 | 0.037 | 0.097 |
| SSS | Stage 3 CKD | -0.150 | 0.040 | 0.00089 |
| IIAVB | Stage 3 CKD | -0.088 | 0.046 | 0.065 |
| RBBB | Stage 3 CKD | 0.120 | 0.033 | 0.001 |

**C**

| exposure | outcome | snp_r2.exposure | snp_r2.outcome | correct_causal_direction | steiger_pval |
| --- | --- | --- | --- | --- | --- |
| Stage V CKD | LBBB | 0.05397 | 0.00058 | TRUE | 0 |
| Stage V CKD | IAVB | 0.05397 | 0.00090 | TRUE | 0 |
| Stage V CKD | IIIAVB | 0.05397 | 0.00114 | TRUE | 0 |
| Stage V CKD | SSS | 0.05397 | 0.00152 | TRUE | 0 |
| Stage V CKD | IIAVB | 0.05397 | 0.00094 | TRUE | 0 |
| Stage V CKD | RBBB | 0.05397 | 0.00109 | TRUE | 0 |
| Stage 3 CKD | LBBB | 0.01374 | 0.00029 | TRUE | 1.37e-133 |
| Stage 3 CKD | IAVB | 0.01374 | 0.00032 | TRUE | 1.28e-130 |
| Stage 3 CKD | IIIAVB | 0.01374 | 0.00029 | TRUE | 7.84e-134 |
| Stage 3 CKD | SSS | 0.013745 | 0.00030 | TRUE | 7.50e-133 |
| Stage 3 CKD | IIAVB | 0.013745 | 0.00034 | TRUE | 2.84e-130 |
| Stage 3 CKD | RBBB | 0.013745 | 0.00041 | TRUE | 7.97e-125 |
